# A 2,2′-diphosphinotolane as a versatile precursor for the synthesis of P-ylidic mesoionic carbenes *via* reversible C–P bond formation†

**DOI:** 10.1039/d0sc06128j

**Published:** 2021-01-29

**Authors:** Hannah K. Wagner, Hubert Wadepohl, Joachim Ballmann

**Affiliations:** Anorganisch-Chemisches Institut, Universität Heidelberg Im Neuenheimer Feld 276 D-69120 Heidelberg Germany joachim.ballmann@uni-heidelberg.de

## Abstract

A metal-templated synthetic route to cyclic (aryl)(ylidic) mesoionic carbenes (**CArY-MIC**s) featuring an endocyclic P-ylide is presented. This approach, which requires metal templates with two *cis*-positioned open coordination sites, is based on the controlled cyclisation of a *P*,*P*′-diisopropyl-substituted 2,2′-diphosphinotolane (**1**) and leads to chelate complexes coordinated by a phosphine donor and the **CArY-MIC** carbon atom. The C–P bond formation involved in the former partial cyclisation of **1** proceeds under mild conditions and was shown to be applicable all over the d-block. In the presence of a third *fac*-positioned open coordination site, the P–C bond formation was found to be reversible, as shown for a series of molybdenum complexes. DFT modelling studies are in line with an interpretation of the target compounds as **CArY-MIC**s.

## Abstract 2

A metal-templated synthesis of cyclic (aryl)(ylidic)mesoionic carbene complexes (**CArY-MICs**) is presented. In the case of molybdenum carbonyls, the crucial P–C bond formation, which occurs during **CArY-MIC** formation, was found to be reversible.

## Graphical abstract

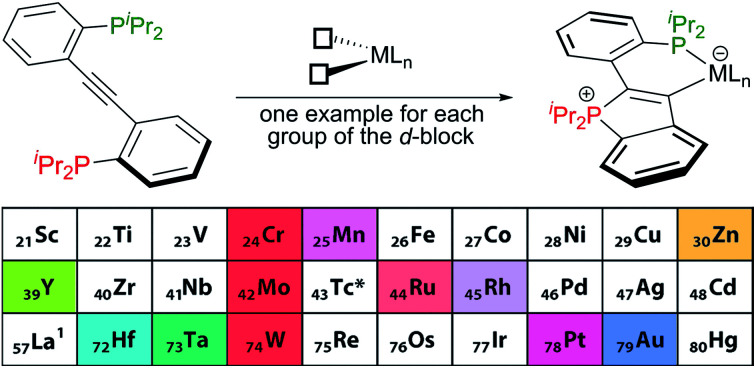

## Introduction

**Scheme 1:**
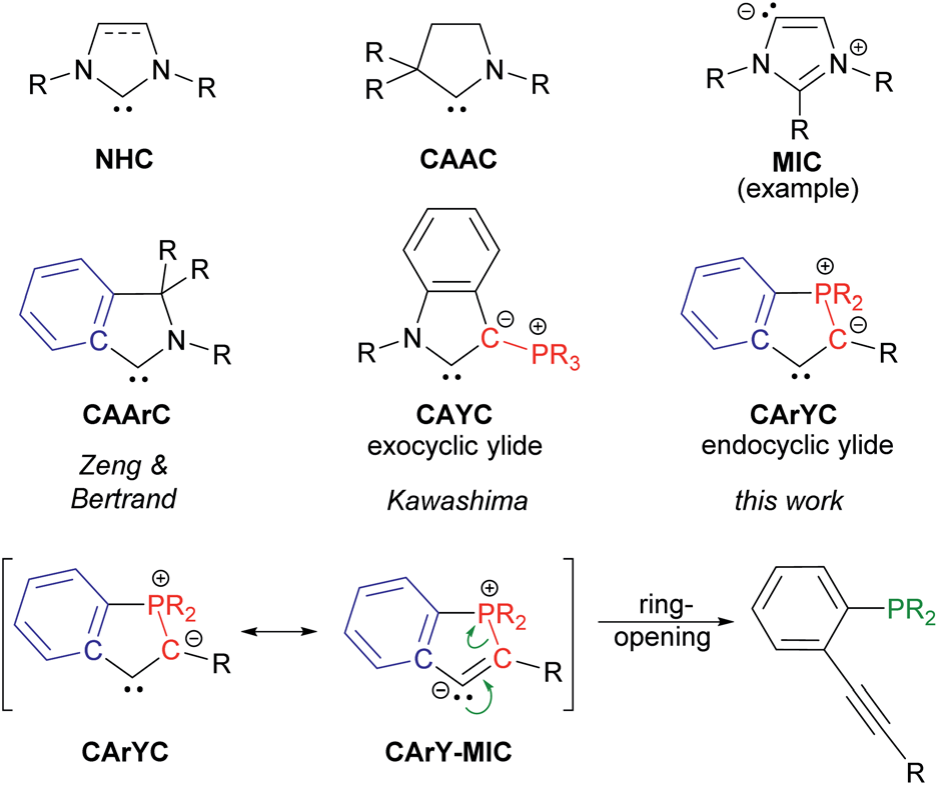
Selected carbene ligands together with the ring-opening of **CArY-MIC**s.

N-Heterocyclic carbenes (**NHC**),^1^ mesoionic carbenes (**MIC**)^2^ and cyclic (alkyl)(amino) carbenes (**CAAC**)^3^ are among the most widely used carbene ligands. Their stability mainly arises from the presence of one or more endocyclic nitrogen atoms stabilising the formally divalent carbon atom *via* donation into its empty p-orbital.^4^ Although spatial protection of the carbene centres certainly plays a role for isolating the free carbenes, the introduction of steric bulk is occasionally not required as metal-templated synthetic routes and *in situ* trapping protocols are well-established nowadays.^5^ The emergence of these procedures opened-up new possibilities to modulate the electronic properties of N-containing carbene ligands, for example by replacing the alkyl groups in **CAAC**s for aryl moieties or by introducing an exocyclic ylide as a replacement for one of the nitrogens in **NHC**s. This approach led to cyclic (amino)(aryl) carbenes (**CAArC**)^6^ and cyclic (amino)(ylide) carbenes (**CAYC**)^7^ (see Scheme 1), respectively, which have been generated *in situ* and trapped as their metal complexes. By formally combining **CAArC**s and P-ylidic **CAYC**s, a new motif in carbene chemistry comes into reach, namely cyclic (aryl)(ylidic) carbenes (**CArYC**s), which do not contain an endocyclic nitrogen atom. In these **CArYC** (see Scheme 1), the quaternary R_2_C-carbon in Zeng's and Bertrand's **CAArC**^6*a*^ is replaced by an ylidic R_2_P-moiety, which results in a structure with an endocyclic P-ylide rather than an exocyclic P-ylide (*cf.* Kawashima's **CAYC**^7*d*^ shown in Scheme 1). In 2010, Bertrand pointed out that free **CArYC**s with an endocyclic P-ylide are expected to be unstable,^5*a*^ which is easily understood considering the corresponding **CArY-MIC** resonance structure (see Scheme 1). A **CArY-MIC** of that type is expected to ring-open to afford the unstrained phosphino alkyne (see Scheme 1). This notion, however, provides a blueprint for the metal-templated assembly of **CArY-MIC**s, namely by reversing the ring-opening process. For electron-withdrawing Ph_2_P-substituted tolanes, such ring-closure reactions are known to afford phosphindolium-based organic materials, usually under harsh reaction conditions.^8^ The use of more nucleophilic alkyl phosphines has not been explored yet, although these phosphines are expected to attack the alkyne more readily. This attack may be further promoted by using a metal template,^8*d*,8*e*^ which is kept in place by means of an appropriately positioned chelate. As explained in the following, we reasoned that ^i^Pr_2_P-substituted 2,2′-diphosphinotolanes are well-suited precursors for the synthesis of the desired **CArY-MIC**s.

**Scheme 2:**
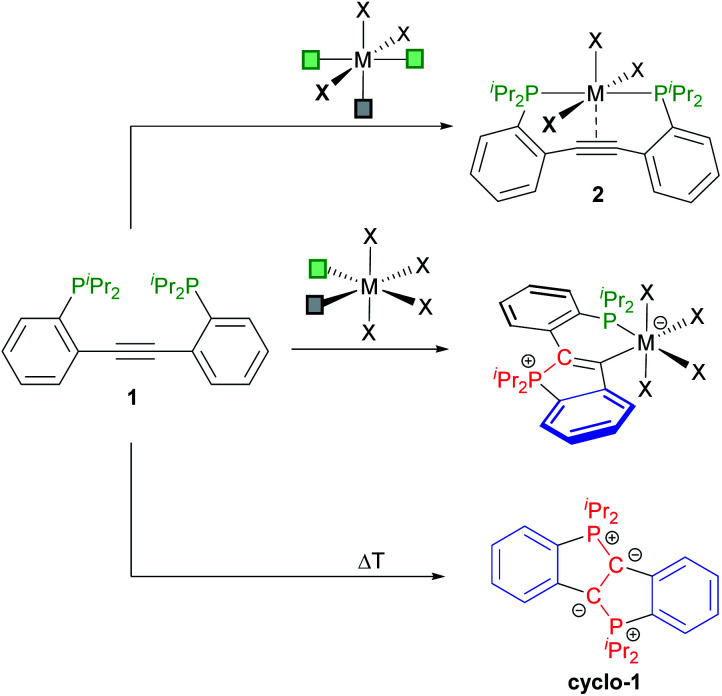
Known routes to [PCCP]-pincer complexes **2** (top) and to the doubly-cyclised diylide **cyclo-1** (bottom) together with the κ^2^-P,C-coordinated **CArY-MIC** complexes targeted herein (middle).‡

In previous studies, 2,2′-diphosphinotolanes, such as **1** (see Scheme 2) were shown to readily form [PCCP]-pincer type complexes (**2**), namely *via* reaction of **1** (or its Ph_2_P-derivative) with octahedral (or square planar) metal precursors with three accessible *mer*-arranged coordination sites.^9^ We have also shown that **1** is prone to a twofold cyclisation to selectively afford the diylide **cyclo-1** (see Scheme 2).^10^ In view of these findings, it seemed possible that the reaction between **1** and a metal template with two *cis*-positioned open coordination sites may afford the desired κ^2^-P,C-chelating **CArY-MIC** complexes *via* attack of only one phosphine at the central alkyne unit.^11^ As reported herein, **CArY-MIC**s may indeed be trapped this way.

## Results and discussion

**Scheme 3:**
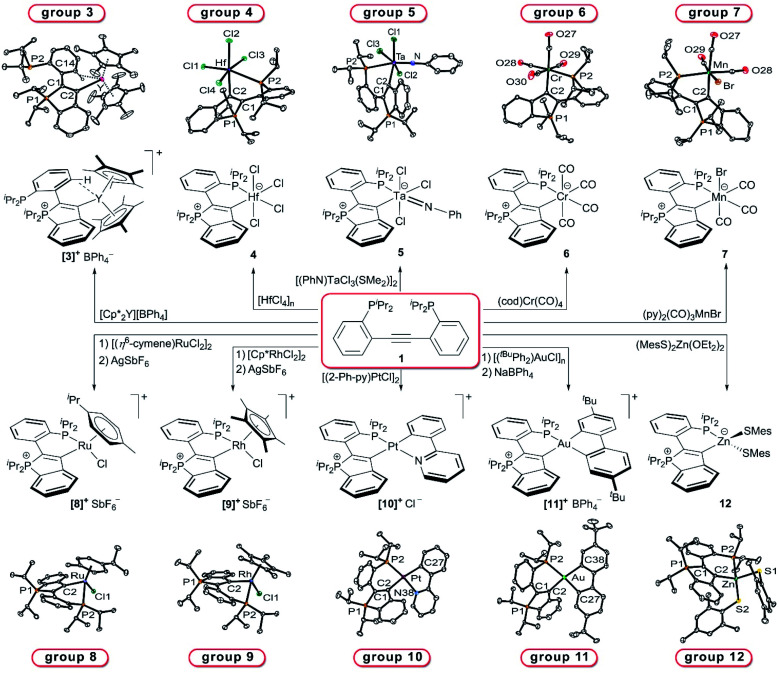
Synthesis of **CArY-MIC** complexes starting from **1**.‡ Each group in the d-block is represented by one crystallographically characterised example.

Following the approach outlined above, numerous transition metal complexes with two accessible coordination sites were reacted with **1** without limiting ourselves to one specific group of the d-block. This screening led to the expected cyclisation of **1** to afford the desired **CArY-MIC**s under mild reaction conditions (all reactions were conducted at or below r.t.). As illustrated in Scheme 3, one example was isolated for each group in the d-block, either as cationic (**3**, **8–11**) or neutral (**4–7**, **12**) derivative (compounds are numbered according to the group in the periodic table). For the successful synthesis of these **CArY-MIC**s, the use of a non-coordinating solvent (chlorobenzene or CH_2_Cl_2_) was found to be mandatory to avoid the formation of coordinatively saturated solvent adducts (*e.g.* HfCl_4_(thf)_2_, which was found to be unreactive towards **1**). To ascertain that the C–P bond formation occurred in the anticipated manner, all complexes were characterised by single crystal X-ray diffraction (see Scheme 3, see ESI† for details, see Table 1 for selected metrical parameters). With exception of the yttrium derivative **3**, a κ^2^-P,C-chelate was formed in each case, independent of the coordination environment (tetrahedral, square planar or octahedral). In the case of **3**, the phosphine is not bound to the metal. Instead, an agostic *ortho*-interaction with one aryl-C–H of the **CArY-MIC** ligand was found (see Scheme 3). This is consistent with the ^31^P{^1^H} NMR spectrum of **3**, which exhibits a yttrium-coupled resonance at *δ* = +52.8 ppm and a singlet at *δ* = −7.7 ppm (*cf. δ*(^31^P{^1^H}) (**1**) = +4.9 ppm). For the zinc complex **12**, a similar situation with one uncoordinated phosphine (^31^P NMR resonance at *δ*(^31^P{^1^H}) = −7.9 ppm) is observed in solution as judged by NMR spectroscopy. For all other complexes, two (mutually coupled) ^31^P{^1^H} NMR resonances are observed between +10 and +63 ppm (see Table 1). For the tantalum derivative **5**, the κ^1^-C- and the κ^2^-C,P-derivatives are both observed in solution (*cf.*^31^P{^1^H} NMR shifts in Table 1), while two stereoisomers (differing in the orientation of the bromide) were found for the manganese complex **7**. In the latter case, only one set of ^31^P{^1^H} NMR signals was observed after treatment with AgBF_4_, but the resulting tetrafluoroborate derivative was not isolated due to its instability.

**Table 1:** Selected experimental data and selected DFT-modelling data (on the PBE1PBE/def2-TZVP or on the BP86-TZ2P-D3 level of theory) for complexes **3–12**

Compound (d^*n*^)	**3** (d^0^)	**4** (d^0^)	**5** (d^0^)	**6** (d^6^)	**7** (d^6^)	**8** (d^6^)	**9** (d^6^)	**10** (d^8^)	**11** (d^8^)	**12** (d^10^)
*δ*(^31^P{^1^H})^a^ (P1)	52.8^b^	53.9^c^	54.6/54.5^c^^,^^d^	43.2^c^	44.1/45.8^c^^,^^d^	49.8^c^	48.2^c^	53.2^c^	55.3^c^	55.0^e^
*δ*(^31^P{^1^H})^a^ (P2)	−7.7^b^	10.3^c^	32.2/−3.4^c^^,^^d^	69.2^c^	57.5/62.4^c^^,^^d^	46.5^c^	47.7^c^	29.6^c^	43.2^c^	−7.9^e^
*δ*(^13^C{^1^H})^a^ (C2)	n.d.^f^	220.8^c^	n.d.^g^	245.5^c^	n.d.^g^	214.1^c^	208.3^c^	198.8^c^	195.6^c^	n.d.^h^
*d*(P1–C1)^i^	1.83	1.82	1.82	1.79	1.80	1.79	1.81	1.80	1.82	1.83–1.84^j^
*d*(C1–C2)^i^	1.37	1.37	1.37	1.38	1.37	1.39	1.37	1.37	1.36	1.36–1.37^j^
*d*(C2–M)^i^	2.47	2.36	2.26	2.11	2.08	2.07	2.05	2.06	2.08	2.04–2.04^j^
IBO σ^C–M^ e^−^(C)/e^−^(M)	1.76/0.15	1.78/0.12	1.78/0.13	1.53/0.30	1.51/0.34	1.36/0.57	1.35/0.54	1.52/0.36	1.49/0.32	1.86/0.06
IBO π^M–C^ e^−^(M)/e^−^(C)	—/—	—/—	—/—	1.18, 1.24^k^/0.06, 0.07^k^	1.46, 1.51^k^/0.04, 0.04^k^	1.86/0.05	1.87/0.05	1.86/0.06	1.95/0.02	—/—
Δ*E*_int_(EDA)^l^	−92.6	−107.7	−111.5	−114.2	−122.7	−190.2	−182.1	−199.1	−167.5	101.6
|*V*_bcp_|/*G*_bcp_(C–M)^m^	1.277	1.456	1.552	1.192	1.278	1.522	1.504	1.555	1.574	1.275
*H*_bcp_/*ρ*_bcp_(C–M)^m^	−0.176	−0.251	−0.322	−0.200	−0.289	−0.428	−0.423	−0.494	−0.455	−0.281
∇^2^*ρ*_bcp_(C–M)^m^	0.096	0.101	0.108	0.282	0.272	0.208	0.217	0.214	0.170	0.259

a Values in ppm.

b In *o*-Cl_2_C_6_D_4_.

c In CD_2_Cl_2_.

d Two isomers (see text).

e In C_6_D_5_NO_2_.

f Not detected due to signal broadening.

g Not detected unambiguously due to the presence of two isomers.

h Not detected due to decomposition over the time of the measurement at −40 °C.

i Values in Å, rounded to two digits (see ESI for additional metrical parameters/standard deviations).

j Four independent molecules are present in the asymmetric unit (see ESI for details).

k Two intrinsic bond orbitals (reminiscent of two d-orbitals) are involved in back-bonding.

l Internal binding energy from energy decomposition analysis.

m Values at the (3,–1) bond critical point of the C–M bond.

**Scheme 4:**
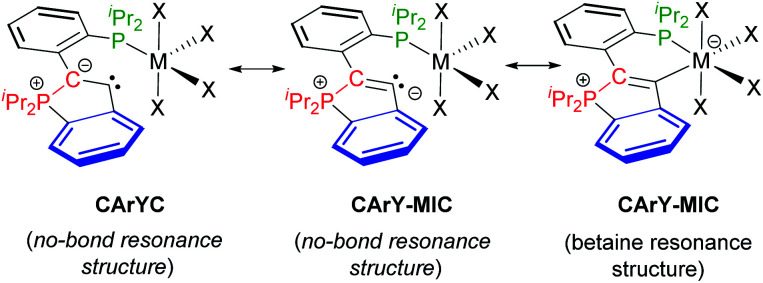
Resonance structures for complexes **3–12**. The **CArYC** no-bond resonance structure with its C1–C2 single bond is considered less important on basis of the observed metrical parameters. The **CArY-MIC** notation (no-bond and betaine resonance structure) is in line with the crystallographic data and the computational results. Note that a dative C→M bond is implied by the superimposition of both **CArY-MIC** resonance structures.

With the molecular structures of complexes **3–12** ascertained, their electronic structures were elucidated, in particular with respect to the nature of the C–M interactions and with respect to the bonding situation within the five-membered P-heterocycles. For true ylides (without resonance stabilisation), significantly shortened P–C bonds in the range of 1.63–1.75 Å are commonly observed.^12^ In the case of complexes **3–12**, this shortening is not observed, *i.e.* the lengths of the P1–C1 bonds (1.81 ± 0.03 Å, see Table 1) are in line with an interpretation as single bonds, which was further confirmed by DFT modelling studies (*vide infra*). The bonds between the carbenoid carbon atoms (C2) and the (formally) ylidic carbon atoms (C1) are best interpreted as partially delocalised C=C double bonds (1.37 ± 0.02 Å, see Table 1, the corresponding C=C bond lengths in 2-aryl 1*H*-indene derivatives range from 1.32–1.42 Å (ref. 13)). Taken together, these crystallographic data support a description of complexes **3–12** as **CArY-MIC**s, which may be denoted with a no-bond and a betaine resonance structure (see Scheme 4). For the betaine resonance structure, a certain degree of backbonding or delocalisation is expected, at least for d^1^- to d^9^-configured metals.^14^ For d^0^- and d^10^-configured metals, backbonding is not possible (empty d-shell) or commonly not observed (closed d-shell),^15^ while hyperconjugative X → C2 interactions are frequently found in these cases (X = metal-bound co-ligand). Keeping in mind that differences between d^1^- to d^9^-configured complexes and d^0^/d^10^-configured complexes have to be expected, we set out to model all complexes by DFT-calculations.

**Fig. 1:**
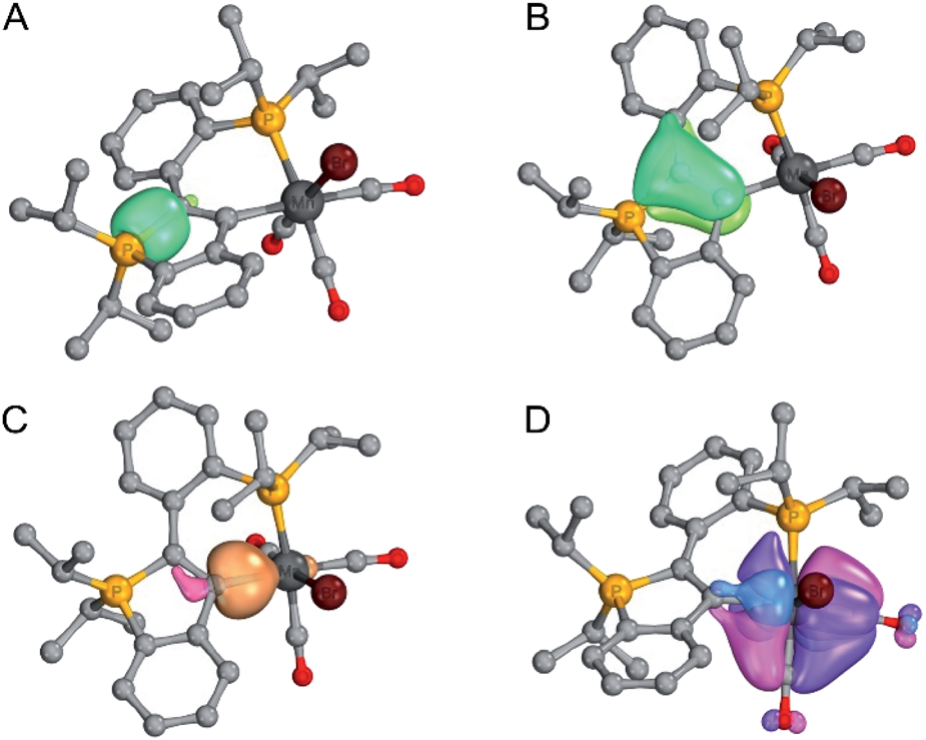
Selected IBO plots for complex **7** ((A) P–C σ-bond, (B) C=C π-bond, (C) dative C→Mn σ-bond, (D) Mn–C π-backbonding *via* two orthogonal IBOs). Threshold value for printing: 75.0 for (A–C), 85.0 for (D).

The geometry of each complex was optimised on the PBE1PBE/Def2-TZVP level of theory and the bonding situation was examined by means of IBO, EDA-NOCV (re-optimised on the BP86/TZ2P-D3 level) and QTAIM analysis. In all complexes, a σ-symmetric P1–C1 single bond (*cf.* example shown in Fig. 1-A) and a π-symmetric C1=C2 double bond (*cf.* example shown in Fig. 1-B) were found by IBO analysis (see ESI† for full details), which is in line with our interpretation of the crystallographic data (*vide supra*).

The IBO charge distributions e^−^(C2)/e^−^(M) in the σ-symmetric C2–M bonds (see Table 1) are indicative of dative C→M bonds, *i.e.* the electrons in these bonds are mainly located at the C2 centres (as represented by the **CArY-MIC** no-bond resonance structure shown in Scheme 4). In the case of the d^6^-configured (**6**, **7**, **[8]+**, **[9]+**) and the d^8^-configured (**[10]+**, **[11]+**) complexes, backbonding interactions between the respective metals and the carbene centres were identified by IBO analysis (see Table 1), suggesting that a delocalized betaine resonance structure is also suited to describe these complexes. For the four complexes with π-acceptor ligands in *trans*-position to the carbene (**6**, **7**, **10** and **11**), delocalised 3c-(4σ + *k*π)e^−^ bonds (with *k* = number of delocalised π-electrons) are equally suited to represent the bonding situation, given that backbonding interactions between the respective metals and the *trans*-positioned π-acceptors were also found in these complexes. As an example, the most important IBOs of **7** are shown in Fig. 1 (see ESI† for analogous IBO plots for all other complexes).

**Fig. 2:**
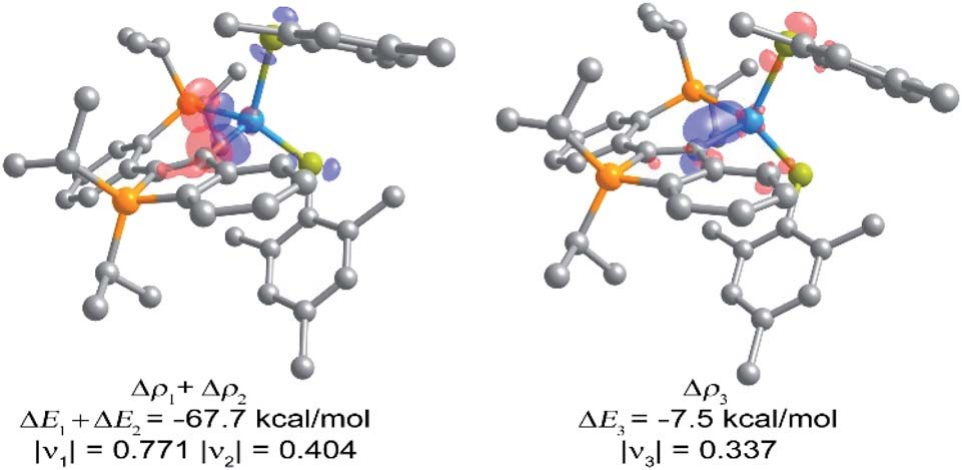
Plots of the EDA-NOCV deformation densities Δ*ρ*_*n*_ for **12** (charge flow: red → blue). The sum Δ*ρ*_1_ + Δ*ρ*_2_ (left) is interpreted as combined σ-donation (C → Zn(SMes)_2_ and P → Zn(SMes)_2_), Δ*ρ*_3_ (right) is interpreted as MesS → C hyperconjugative stabilisation. The eigenvalues |*ν*_*n*_| indicate the relative magnitude of the charge flow.

As expected (*vide supra*), π-backbonding IBO interactions were only detected for complexes **6–11**, but not for the d^0^- and d^10^-configured derivatives (**[3]+**, **4**, **5** and **12**). To gain further insights into the electronic structures of the latter four complexes, energy decomposition analysis (EDA-NOCV) was used to fragment each complex into a closed-shell ligand part and a closed-shell metal-containing part. Due to the chelating nature of our ligand, a cautionary note is required, given that EDA-NOCV is commonly used for monodentate ligands, which affords a clean picture of the fragmented metal–ligand bond.^16^ In our case, however, the individual contributions from the phosphine and the carbene donors are not separated, which requires a visual inspection of all EDA-NOCV deformation densities and interferes with a quantitative analysis. Nevertheless, a coarse picture of the bonding situation in complexes **3–12** was obtained using this methodology and the expected π-backbonding interactions were found for complexes **6–11**. The respective NOCV deformation densities for complexes **4**, **5** and **12** are in line with the presence of hyperconjugative interactions between one of the metal-bound co-ligands and the carbene centres (Hf–Cl → C2 for **4**, Ta=N^Ph^ → C2 for **5** and Zn–S^Mes^ → C2 for **12**). Given that similar interactions are often found for d^0^-configured NHC-complexes,^4*b*,15^ this analysis argues for an interpretation of **4**, **5** and **12** as carbenoid species. Selected deformation densities of **12** are shown in Fig. 2 as an example (see ESI† for full detail on EDA-NOCV calculation for **3–12**). For the yttrium derivative **3**, EDA-NOCV analysis indicated that hyperconjugative interactions between the [Cp*_2_Y]^+^ fragment and the ligand play a minor role, while a dative C→Y bond is found upon inspection of the NOCV deformation densities (see ESI, Fig. S56†).

To assess the covalency of the metal carbon σ-bond, the electron densities and energy densities at the (3,–1) bond critical points (bcp) of the M–C bonds were calculated for all complexes **3–12** by means of topological wavefunction analysis (Bader's QTAIM methodology).^17^

**Fig. 3:**
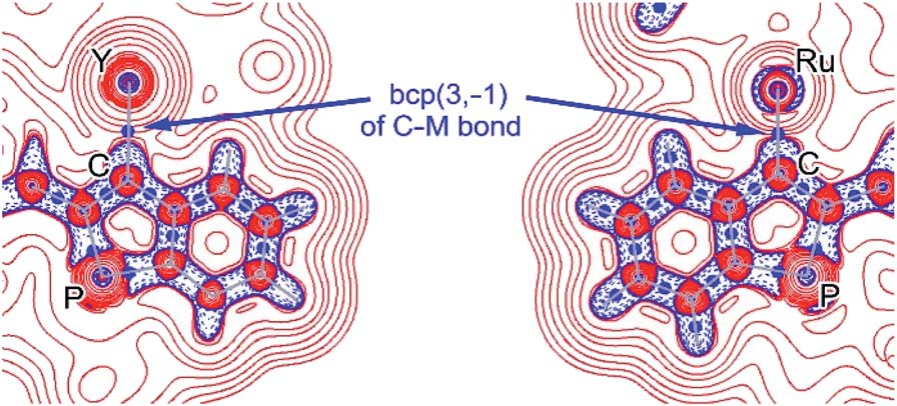
Counter plot of the laplacian of the electron densities (∇^2^*ρ*) of **[3]+** (left) and **8** (right). Positive and negative values are shown in red and blue respectively.

The ratio |*V*_bcp_|/*G*_bcp_ (with *V*_bcp_ = virial potential energy at the bcp, *G*_bcp_ = kinetic energy density at the bcp) is expected to be >2 for covalent bonds and <1 for ionic bonds.^18^ For dative and highly polarised bonds, values in the regime 1 < |*V*_bcp_|/*G*_bcp_ < 2 are expected and indeed found for **3–12** (see Table 1). Together with the bond degrees (defined as *H*_bcp_/*ρ*_bcp_: expected to be negative for dative bonds; *H*_bcp_ = total energy density at the bcp, *ρ*_bcp_ = electron density at the bcp) and the laplacian of electron densities at the bcp (∇^2^*ρ*_bcp_: expected to be positive for dative bonds),^19,18*a*^ it is concluded that the C→M σ-bonds in all complexes **3–12** (see Table 1) are best described as dative bonds as implied by an overlay of both **CArY-MIC** resonance structures shown in Scheme 4. As examples, ∇^2^*ρ* contour plots for **[3]+** and **8** are shown in Fig. 3 (see ESI† for the corresponding plots for complexes **3–12**). Notably, the ^13^C{^1^H} NMR shifts of the carbenoid C2 centres (approx. 200 ppm or higher) agree with this notion as similar chemical shift values are commonly found for N-stabilised heterocyclic carbenes.^14*d*^

**Scheme 5:**
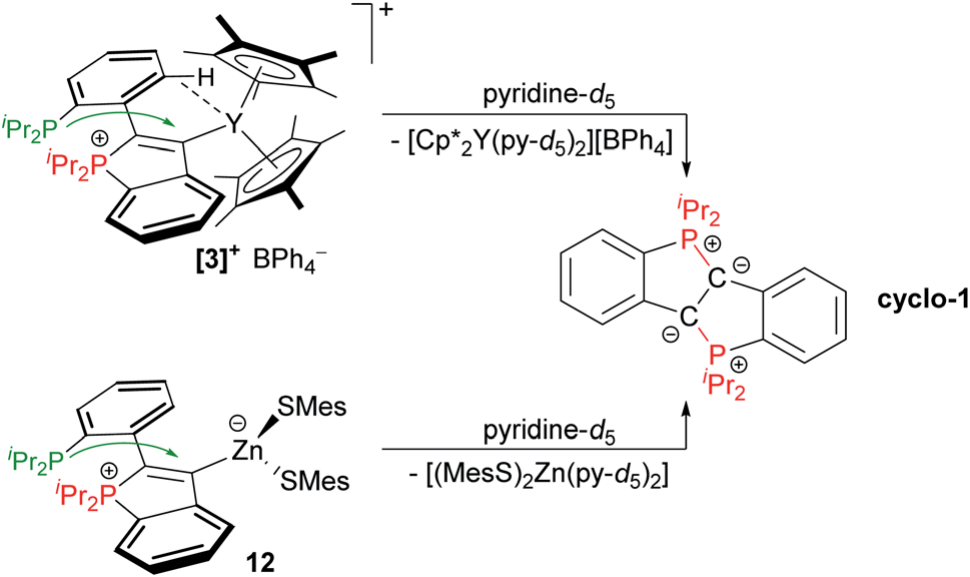
Reaction of **3** and **12** with pyridine-*d*_5_. In the case of **12**, **cyclo-1** is produced exclusively, while **1** (major) is generated along with **cyclo-1** (minor) in the case of **3**.‡

In the case of **3** and **12**, which are both κ^1^-C-coordinated in solution (*vide supra*), ligand substitution reactions were considered conceivable, also as these two complexes were found to exhibit the smallest interaction energies Δ*E*_int_ of the series **3–12** (according to energy decomposition analysis, see Table 1). Upon dissolution of **3** or **12** in pyridine-*d*_5_, deep green solutions containing **cyclo-1** were obtained within seconds at room temperature. For the formation of **cyclo-1** in the absence of a metal complex, solution of **1** need to be kept at 60 °C for several days.^10^ Therefore, it is assumed that pyridine-*d*_5_ coordinates to the metal centres in **3** and **12**, which induces the second cyclisation event, either with the **CArY-MIC** still within the coordination sphere of the metal or *via* displacement and liberation of the free carbene. The free carbene is then expected to either rapidly cyclise to **cyclo-1** or to ring-open to **1**. NMR analysis revealed that **cyclo-1** is the only product formed in the case of **12**. For **3**, however, the deep green solution obtained after reaction with pyridine-*d*_5_ was found to contain only small amounts of **cyclo-1** (3–5%), while **1** was produced in more than 90% conversion. Evidently, the C–P bond, which is formed during the synthesis of **3** was re-opened, at least in this case (Scheme 5).

**Scheme 6:**
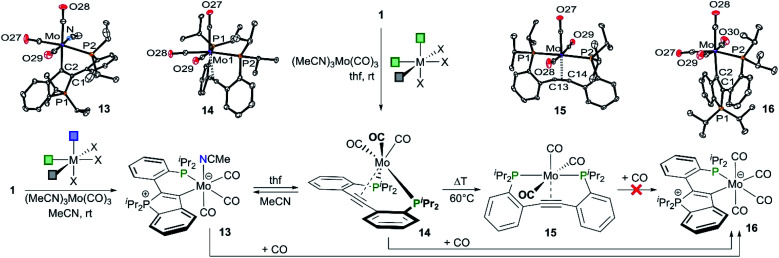
P–C bond cleavage and re-formation in a series of molybdenum complexes (**13–16**).‡

To elucidate whether similar C–P bond cleavage reactions are also possible within the coordination sphere of a metal, a **CArY-MIC** complex with one free coordination site was sought-after. In such a complex, the endocyclic phosphine, which is liberated during ring-opening may be trapped by the metal ion. However, the free coordination site must be *fac*-positioned with respect to the κ^2^-C,P-bound **CArY-MIC** to circumvent the formation of a [PCCP]-pincer complex (*cf.*Scheme 2). Hence, **1** was reacted with *fac*-(MeCN)_3_Mo(CO)_3_ in MeCN and the expected **CArY-MIC** complex **13** was isolated (see Scheme 6). Two MeCN ligands in *fac*-(MeCN)_3_Mo(CO)_3_ were substituted, *i.e.* one MeCN ligand was left behind in *fac*-position to the P,C-chelate. Upon dissolution of **13** in thf, ring-opening of the P-heterocycle and dissociation of the MeCN ligand occurred over 24 h to afford the *fac*-coordinated complex **14** (see Scheme 6). This process was found to be fully reversible as shown by interconverting **13** and **14** several times, simply by changing the solvent (and keeping the thus obtained solutions for 24 h at r.t.). In this context, it needs to be noted that **13** precipitates from solution upon dissolution of **14** in MeCN, which may add its share to the driving force of the reverse reaction. Compound **14** may also be prepared directly *via* reaction between **1** and *fac*-(MeCN)_3_Mo(CO)_3_ in thf at room temperature. Upon heating (60 °C in thf), **14** was found to rearrange irreversibly to the *mer*-coordinated [PCCP]-pincer complex **15** (see Scheme 6). The finding that no reverse reaction between **15** and MeCN was observed, suggested that a local minimum on the potential energy surface was reached upon pincer formation. This was further elaborated by examining the reaction of **13–15** with excess CO. While **13** and **14** both reacted instantaneously with CO to afford **16**^20^ (see Scheme 6, see Table 2 for characterisation data for **13–16**), no reaction was noticed upon pressurising samples of **15** with carbon monoxide, not even under forcing reaction conditions (10 bar CO, 110 °C in toluene-*d*_8_). According to DFT calculations (PBE1PBE/Def2-TZVP-GD3), the reaction of **15** with CO to afford **16** is thermodynamically favoured (Δ*G* = 13.0 kcal mol^−1^, see ESI† for details), indicative of a high kinetic barrier prohibiting this reaction in our experiments. With the different reaction pathways interconnecting **13–16** now established, the focus was set on the most exciting reactivity pattern, namely the reversible P–C bond formation interconnecting **13** and **14**. As the low solubility of **13** in MeCN (*vide supra*) precluded a meaningful kinetic analysis, a DFT modelling study (PBE1PBE/Def2-TZVP-GD3) was carried out to further elucidate this transformation.

**Table 2:** Characterisation data for compounds **13–16**

Compound	**13**	**14**	**15**	**16**
*δ*(^31^P{^1^H})^a^	43.0/49.6	54.3	87.5	46.0/51.0
*ν*(CO)^b^	1994, 1887, 1790, 1751	1930, 1847, 1829, 1818	1974, 1946, 1865, 1837	1987, 1892, 1855, 1826
*d*(C–C)^c^^,^^d^	1.38	1.24^e^	1.25	1.38
*d*(P–Mo)^d^	2.51	2.54/2.60^e^	2.50/2.50	2.50
*d*(C–Mo)^d^	2.25	2.38/2.40^e^	2.30/2.30	2.26

a Recorded in different solvents (**13**: thf-*d*_8_/MeCN-*d*_3_ = 4/1, **14**: thf-*d*_8_, **15**: C_6_D_6_, **16**: CD_2_Cl_2_) values in ppm.

b Carbonyl stretching frequencies (solid state, ATR-IR, some vibrations are coupled to vibrations of the alkyne or the MeCN ligand in **13**), values in cm^−1^.

c C=C distance in **13** and **16**, C≡C distance in **14** and **15**.

d Rounded to two digits (see ESI for additional metrical parameters and standard deviations), values in Å.

e Two independent molecules are present in the asymmetric unit (second molecule: *d*(C–C) = 1.23 Å, *d*(P–Mo) = 2.56/2.57 Å, *d*(C–Mo) = 2.37/2.40 Å).

Three different scenarios for the key step of the forward reaction **13** → **14** (exp. conditions: thf, r.t., approx. 95% conversion in 24 h) were inspected *in silico*: (a) initial dissociation of the MeCN ligand of **13** to afford a five-coordinate molybdenum centre and subsequent ring-opening of the **CArY-MIC** (P–C bond cleavage), (b) initial replacement of the MeCN ligand of **13** for a thf ligand and consecutive P–C bond cleavage once the thf-analogue of **13** has been formed, and (c) initial ring-opening of the **CArY-MIC** in **13** followed by dissociation of MeCN. The possibilities (a) and (b) had to be excluded (see ESI† for details) due to barriers of 34.7 and 31.8 kcal mol^−1^ respectively, which cannot be overcome under the employed reaction conditions.

**Fig. 4:**
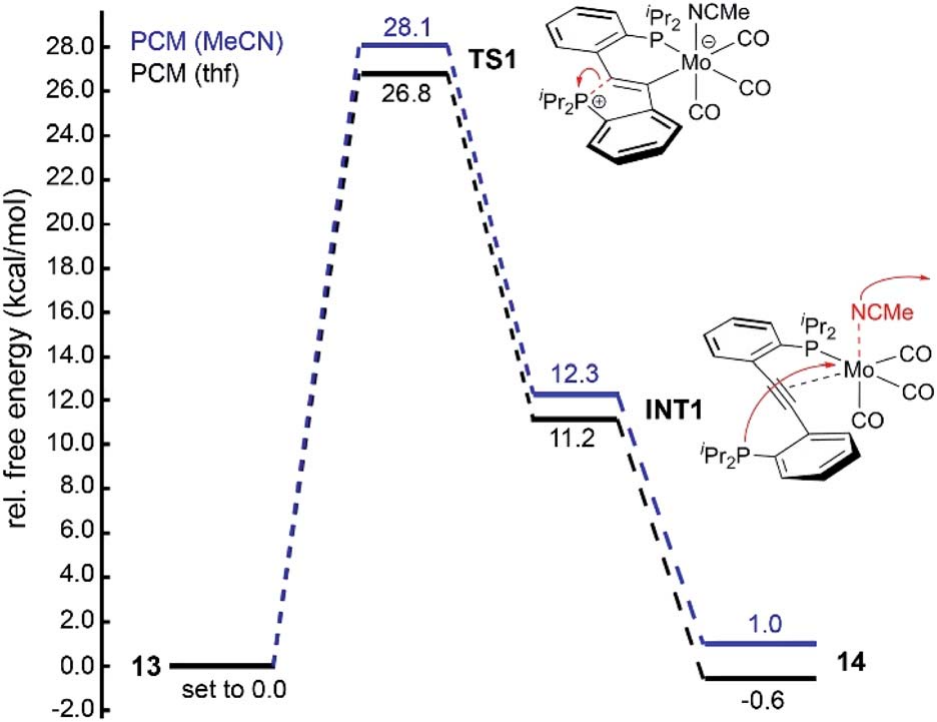
Calculated (PBE1PBE/Def2-TZVP-GD3) energy profile for the conversion of **13** (set to 0.0 kcal mol^−1^) to **14** in thf (PCM solvent correction for thf, black lines) and for the backward reaction in MeCN (PCM solvent correction for MeCN, blue lines).‡ Free energies are corrected for the liberated (**13** → **14**) or incoming (**13** → **14**) MeCN ligand.

For the third scenario, a barrier of 26.8 kcal mol^−1^ was calculated (PBE1PBE/Def2-TZVP-GD3, solvent corrected for thf) for the forward reaction **13** → **14** (*cf.***TS1** in Fig. 4), which is slightly higher than expected (≈25 kcal mol^−1^)^21^ on basis of the reaction conditions. This discrepancy, however, is well within the error (up to 4 kcal mol^−1^)^22^ of our DFT model. We also noticed that the calculated energies for **TS1** were found to be nearly independent of the applied functional, but significantly affected by dispersion and solvent correction terms (see ESI† for details). A considerably lower barrier of only 21.8 kcal mol^−1^ was found for **TS1** when dispersion effects were neglected, suggesting that small geometric distortions may lead to large errors, in particular with respect to **TS1**. Hence, the reaction pathway shown in Fig. 4 was considered the most plausible scenario. In the first step of the forward reaction **13** → **14**, ring-opening of the **CArY-MIC** in **13** takes place, which gives rise to intermediate **INT1**. Throughout this step, the MeCN ligand in **13** remains coordinated to the molybdenum centre. Upon dissociation of the MeCN ligand in **INT1**, a spontaneous (barrier-free) coordination of the phosphine was found to occur, *i.e.* the experimentally observed product **14** is formed without an additional activation barrier. Once again, dispersion effects were found to significantly influence the free energy associated with this second step, *i.e.* upon neglecting dispersion effects, an activation barrier of approximately 10 kcal mol^−1^ was calculated for this step (see Fig. 4, see ESI† for details). Overall, the forward reaction **13** → **14** was found to be exergonic by Δ*G* = −0.6 kcal mol^−1^ (PBE1PBE/Def-2-TZVP-GD3, solvent corrected for thf), which agrees with our experiments. For the reverse reaction **14** → **13** (PBE1PBE/Def-2-TZVP-GD3, solvent corrected for MeCN), a nearly identical barrier of 27.1 kcal mol^−1^ (referenced against **14**) was found for **TS1**. The overall reaction was found to be exergonic by Δ*G* = −1.0 kcal mol^−1^, suggesting that the ground state energies are well-described with the employed functional.

In ongoing work, various modifications^23^ at the **CArY-MIC** backbone and at the co-ligands in **13** and **14** are examined *in vitro* and *in silico* to identify similar interconversions between facially coordinated phosphinoalkyne complexes and **CArY-MIC** complexes. These efforts are directed towards a deeper understanding of this new ligand-cooperative^24^ reactivity pattern.

## Conclusions

In summary, we have shown that a new class of **CArY-MIC** complexes is accessible under mild conditions *via* reaction between **1** and a variety of metal precursors, each featuring two *cis*-positioned open coordination sites. This methodology was found to be applicable all over the d-block as shown by the preparation of **3–12**. The P–C bond formation involved in this transformation was shown to be reversible, at least under certain circumstances, as demonstrated for a series of molybdenum complexes (**13–16**). This new ligand-cooperative reactivity pattern is further explored in our laboratory.

## Author contributions

H. K. Wagner carried out the experimental work and parts of the computational work. The crystallographic work was carried out by H. Wadepohl. J. Ballmann wrote the manuscript and carried out parts of the computational work. The ESI† was compiled by all three authors.

## Conflicts of interest

There are no conflicts to declare.

## Supplementary Material

SC-012-D0SC06128J-s001

SC-012-D0SC06128J-s002
