# Supplementary material for: A 2,2′-diphosphinotolane as a versatile precursor for the synthesis of P-ylidic mesoionic carbenes via reversible C–P bond formation
Source: Chem Sci. 2021 Jan 29;12(10):3693–701. doi: 10.1039/d0sc06128j (PMC8179451; doi:10.1039/d0sc06128j)
Supplement: SC-012-D0SC06128J-s001 [file SC-012-D0SC06128J-s001.pdf]

Electronic Supplementary Material (ESI) for Chemical Science.
This journal is © The Royal Society of Chemistry 2021

Electronic Supplementary Information

A 2,2'-Diphosphinotolane as a Versatile Precursor for the Synthesis of *P*-Ylidic Mesoionic Carbenes via Reversible C–P Bond Formation

Hannah K. Wagner, Hubert Wadepohl and Joachim Ballmann*
Anorganisch-Chemisches Institut, Ruprecht-Karls-Universität Heidelberg, Im Neuenheimer Feld 276, 69120 Heidelberg, Germany.

Abstract: A metal-templated synthetic route to cyclic (aryl)(ylidic) mesoionic carbenes (**CArY-MIC**s) featuring an endocyclic *P*-ylide is presented. This approach, which requires metal templates with two *cis*-positioned open coordination sites, is based on the controlled cyclisation of a *P*,*P*'-diisopropyl-substituted 2,2'-diphosphinotolane (**1**) and leads to chelate complexes coordinated by a phosphine donor and the **CArY-MIC** carbon atom. The C–P bond formation involved in the former cyclisation of **1** proceeds under mild conditions and was shown to be applicable all over the *d*-block. In the presence of a third *fac*-positioned open coordination site, the P–C bond formation was found to be reversible, as shown for a series of molybdenum complexes. DFT modelling studies are in line with an interpretation of the target compounds as **CArY-MIC**s.

Table of Contents

Experimental Procedures 1
NMR Spectra 7
Details on DFT Calculations 27
X-Ray Crystal Structure Determinations 85
References 98

Experimental Procedures

General Remarks

All experiments were conducted under an atmosphere of dry and oxygen-free argon by using standard Schlenk techniques or in a glovebox (MBraun). Argon 5.0 was used and further dried by passing over columns of phosphorus pentoxide. Glassware was heated to 130°C overnight and evacuated while still hot. Dichloromethane, diethyl ether, hexane, pentane, thf and toluene were purified by a MBraun Solvent Purification System. Fluorobenzene was dried over calcium hydride. Deuterated solvents were dried over sodium (C_6D_6, thf-d_8) or over calcium hydride (CD_2Cl_2) and distilled prior to use. $C_6D_5NO_2$ and *o*-$C_6D_4Cl_2$ were stored over activated molecular sieves. 2,2'-Bis(diisopropylphosphanyl)tolane **1** was prepared according to literature.[1] Metal precursors that are not commercially available were prepared according to literature (references are provided when these precursors first appear in the text). All other chemicals were purchased from commercial suppliers and used as received. One and two dimensional ^{1}H, ^{13}C and ^{31}P NMR spectra were recorded on a Bruker Avance II 400 MHz or on a Bruker Avance III 600 spectrometer. Residual (undeuterated) solvent served as reference for ^{1}H and ^{13}C NMR spectra.[2] Chemical shifts δ are given in parts per million (ppm), coupling constants J in Hertz (Hz). Signal multiplicities are stated using common abbreviations (e.g. s – singlet, d – doublet, dd – doublet of doublets). Mass spectra were recorded at the Department of Organic Chemistry at our University on Bruker ApexQe FT-ICR Instrument by ESI technique or on a JEOL AccuTOF GCx Instrument by LIFDI technique. Elemental analyses were carried out at the Department of Inorganic Chemistry at Heidelberg University on an Elementar vario MICRO Cube.

Synthesis of complex [3]$^{+}$BPh$_4^{-}$

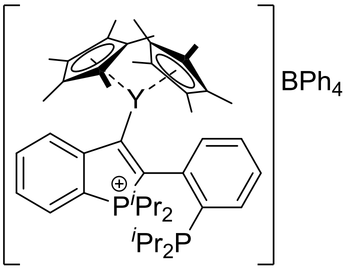

$Cp^*_2Y(\mu\text{-}Ph)_2BPh_2$[3] (147 µmol, 100 mg) and 1.1 eq **1** (147 µmol, 60.5 mg) were dissolved in C_6H_5F (5 mL) and stirred at room temperature for 30 min. The resulting turbid solution was filtered through a PTFE syringe filter. The clear filtrate was layered with pentane and stored at –40°C for 24 h. The precipitate was isolated by decanting the supernatant solution. The solid residue was washed with pentane/toluene 1:1 and dried *in vacuo* to afford the product **3** as a nearly white (slightly greenish) solid (59.5 mg, 37%). ^{1}H NMR (*o*-$C_6D_4Cl_2$, 600 MHz, 22°C): δ (in ppm) = 7.89-7.88 (m, 8H, BPh_4), 7.64 (d, $^{3}J_{H\text{-}H}$ = 7.4 Hz, 1H), 7.60 (t, $^{3}J_{H\text{-}H}$ = 7.5 Hz, 1H), 7.40-7.37 (m, 2H), 7.31-7.28 (m, 1H), 7.22 (t, $^{3}J_{H\text{-}H}$ = 7.2 Hz, 8H, BPh_4), 7.09-7.06 (m, 5H), 6.63 (d, $^{3}J_{H\text{-}H}$ = 7.6 Hz, 1H), 6.33-6.32 (m, 1H), 3.69-3.62 (m, 2H, CH), 2.05-2.00 (m, 2H, CH), 1.65 (s, 30H, Cp*), 1.26 (dd, $^{3}J_{H\text{-}P}$ = 17.2 Hz, $^{3}J_{H\text{-}H}$ = 7.1 Hz, 6H, CH_3), 1.14 (dd, $^{3}J_{H\text{-}P}$ = 13.1 Hz, $^{3}J_{H\text{-}H}$ = 6.8 Hz, 6H, CH_3), 0.92-0.85 (m, 12H, CH_3). ^{13}C NMR spectra interpretation was impeded due to unresolved ^{89}Y coupling and signal broadening. $^{31}P\{^{1}H\}$ NMR (*o*-$C_6D_4Cl_2$, 243 MHz, 22°C): δ (in ppm) =52.8 (dd, $^{3}J_{P\text{-}Y}$ = 14 Hz, $J_{P\text{-}P}$ = 3 Hz, 1P, P-C), –7.7 (s, 1P, free P). Numerous attempts to analyse the compound by mass spectrometry (LIFDI) failed due to its instability in solution at room temperature. Anal. Calcd. for $C_{70}H_{86}BP_2Y$ (1089.12 g/mol): C 77.20, H 7.96. Found: C 77.29, H 7.88.

Synthesis of complex 4

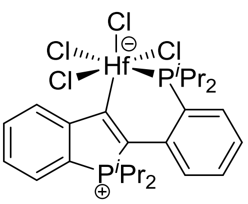

$HfCl_4$ (139 µmol, 44.4 mg) and 1.1 eq **1** (152 µmol, 62.6 mg) were dissolved in CH_2Cl_2 (6 ml) and stirred at room temperature for 1 h. The resulting yellow solution was stored at –40°C overnight to precipitate small amounts of unreacted $HfCl_4$, which were filtered off. The remaining clear solution was evaporated to dryness and the solid residue was washed with pentane, Et_2O and $CHCl_3$ and dried *in vacuo* to afford the product **4** as a yellow solid (57.6 mg, 57%). ^{1}H NMR (CD_2Cl_2, 600 MHz, 22°C): δ (in ppm) = 9.16 (dd, $^{3}J_{H\text{-}H}$ = 8.0 Hz, $^{3}J_{H\text{-}H}$ = 2.9 Hz, 1H), 7.79 (t, $^{3}J_{H\text{-}H}$ = 7.8 Hz, 1H), 7.59-7.55 (m, 2H), 7.53 (t, $^{3}J_{H\text{-}H}$ = 7.6 Hz, 1H), 7.46-7.41 (m, 2H), 7.20-7.18 (m, 1H), 3.03-2.97 (m, 2H, CH), 2.75-2.69 (m, 2H, CH), 1.34-1.25 (m, 18H, CH_3), 1.19 (dd, $^{3}J_{H\text{-}P}$ = 18.0 Hz, $^{3}J_{H\text{-}H}$ = 7.2 Hz, 6H, CH_3). $^{13}C\{^{1}H\}$ NMR (CD_2Cl_2, 151 MHz, 22°C): δ (in ppm) = 220.8 (C_q, 1C, C-Hf), 158.0 (C_q, dd, $J_{C\text{-}P}$ = 36 Hz, $J_{C\text{-}P}$ = 3 Hz, 1C), 144.4 (C_q, dd, $J_{C\text{-}P}$ = 22 Hz, $J_{C\text{-}P}$ = 11 Hz, 1C), 135.5 (CH, d, $J_{C\text{-}P}$ = 7 Hz, 1C), 135.0 (CH, d, $J_{C\text{-}P}$ = 2 Hz, 1C), 135.0 (CH, 1C), 134.7 (C_q, d, $J_{C\text{-}P}$ = 3 Hz, 1C), 130.7 (CH, d, $J_{C\text{-}P}$ = 2 Hz, 1C), 129.2 (CH, dd, $J_{C\text{-}P}$ = 8 Hz, $J_{C\text{-}P}$ = 6 Hz, 1C), 129.0 (CH, d, $J_{C\text{-}P}$ = 11 Hz, 1C), 128.3 (CH, d, $J_{C\text{-}P}$ = 9 Hz, 1C), 128.1 (CH, dd, $J_{C\text{-}P}$ = 5 Hz, $J_{C\text{-}P}$ = 1 Hz, 1C), 126.8 (C_q, dd, $J_{C\text{-}P}$ = 23 Hz, $J_{C\text{-}P}$ = 10 Hz, 1C), 117.2 (C_q, d, $J_{C\text{-}P}$ = 95 Hz, 1C), 25.5 (CH, broad, 1C), 25.3 (CH, broad, 1C), 24.6 (CH, broad, 2C), 19.2 (CH_3, broad, 2C), 17.7 (CH_3, 2C), 17.4 (CH_3,

d, $^{2}J_{C-P}$ = 3 Hz, 2C), 16.8 (CH_3, d, $^{2}J_{C-P}$ = 2 Hz, 2C). The signal at 220.8 ppm was found in a $^{13}C\{^{1}H, ^{31}P\}$ NMR spectrum (CD_2Cl_2, 151 MHz, 22°C). $^{31}P\{^{1}H\}$ NMR (CD_2Cl_2, 243 MHz, 22°C): δ (in ppm) =53.9 (d, $^{4}J_{P-P}$ = 9 Hz, 1P, P-C), 10.3 (d, $^{4}J_{P-P}$ = 9 Hz, d, 1P, P-Hf). MS (LIFDI, CH_2Cl_2): Calcd. for $[C_{26}H_{36}Cl_3HfP_2]^+$: 695.0818. Found: 695.0 $[M-Cl]^+$. The expected isotopic pattern agrees well with the measurement. Anal. Calcd. for $C_{26}H_{36}Cl_4HfP_2$ (730.81 g/mol): C 42.73, H 4.97. Found: C 41.45, H 4.96. Carbon values were consistently low, presumably due to carbide formation upon combustion.

Synthesis of complex 5

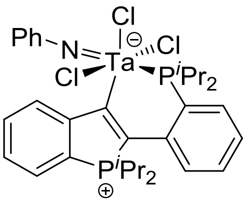

$[PhN{=}TaCl_2(SMe_2)(\mu\text{-}Cl)]_2$[4] (56.8 µmol, 50.0 mg) and 2.0 eq **1** (114 µmol, 46.6 mg) were suspended in toluene (4 mL) and stirred at room temperature for 22 h. The resulting precipitate was filtered off and washed with toluene and dried *in vacuo* to afford the product **5** as a yellow to orange solid (89.0 mg, 99%). The product was obtained as a mixture of isomers in a 1:1 ratio (coordinated and dangling phosphine). Due to signal broadening (exchange broadening), ^{1}H and ^{13}C NMR spectra were not interpretable, not even at low temperature. $^{31}P\{^{1}H\}$ NMR (CD_2Cl_2, 243 MHz, 22°C): δ (in ppm) = 54.5 (d overlapping with broad s, 2P, P-C, isomers a and b), 32.2 (s, 1P, P-Ta, isomer a), –3.4 (s, 1P, decoordinated, isomer b). MS (LIFDI, CH_2Cl_2): Calcd. for $[C_{32}H_{41}Cl_3NP_2Ta]^+$: 787,1254. Found: 787.2 $[M]^+$. The expected isotopic pattern agrees well with the measurement. Anal. Calcd. for $C_{32}H_{41}Cl_3NP_2Ta$ (788.93 g/mol): C 48.72, H 5.24, N 1.78. Found: C 48.76, H 5.01, N 2.15.

Synthesis of complex 6

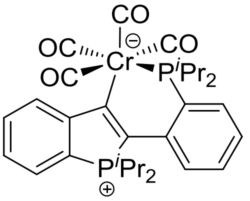

$Cr(CO)_4(COD)$[5] (735 µmol, 200 mg) and 1.0 eq **1** (735 µmol, 302 mg) were dissolved in toluene (12 mL) and stirred at room temperature for 21 h. The resulting dark solution was cooled to –40 °C. The precipitate was filtered off and washed with pentane. Recrystallisation from CH_2Cl_2/Et_2O afforded the product **6** as a dark purple solid (235 mg, 56%). $^{1}H\{^{31}P\}$ NMR (CD_2Cl_2, 400 MHz, 22°C): δ (in ppm) = 8.85 (d, $^{3}J_{H-H}$ = 8.0 Hz, 1H), 7.85 (t, $^{3}J_{H-H}$ = 7.7 Hz, 1H), 7.50-7.47 (m, 2H), 7.43-7.38 (m, 2H), 7.50 (t, $^{3}J_{H-H}$ = 7.5 Hz, 1H), 7.05 (d, $^{3}J_{H-H}$ = 7.7 Hz, 1H), 2.92 (sept, $^{3}J_{H-H}$ = 7.1 Hz, 2H, CH), 2.44 (sept, $^{3}J_{H-H}$ = 6.9 Hz, 2H, CH), 1.25 (d, $^{3}J_{H-H}$ = 6.9 Hz, 6H, CH_3), 1.13 (d, $^{3}J_{H-H}$ = 6.9 Hz, 6H, CH_3), 1.13 (d, $^{3}J_{H-H}$ = 7.1 Hz, 6H, CH_3), 1.01 (d, $^{3}J_{H-H}$ = 7.1 Hz, 6H, CH_3). $^{13}C\{^{1}H, ^{31}P\}$ NMR (CD_2Cl_2, 151 MHz, 22°C): δ (in ppm) = 245.5 (C_q, 1C, C-Cr), 234.1 (C_q, 1C, CO), 231.4 (C_q, 1C, CO), 224.8 (C_q, 2C, CO), 158.3 (C_q, 1C), 145.3 (C_q, 1C), 135.4 (CH, 1C), 134.4 (CH, 1C), 131.9 (CH, 1C), 130.0 (CH, 1C), 128.9 (CH, 1C), 127.5 (CH, 1C), 127.3 (CH, 1C), 126.0 (C_q, 1C), 125.2 (CH, 1C), 121.9 (C_q, 1C), 116.2 (C_q, 1C), 26.3 (CH, 2C), 25.0 (CH, 2C), 18.2 (CH_3, 2C), 17.7 (CH_3, 2C), 17.3 (CH_3, 2C) , 16.7 (CH_3, 2C). $^{31}P\{^{1}H\}$ NMR (CD_2Cl_2, 162 MHz, 22°C): δ (in ppm) = 69.2 (d, $^{4}J_{P-P}$ = 11 Hz, 1P, P-Cr), 43.2 (d, $^{4}J_{P-P}$ = 11 Hz, 1P, P-C). MS (LIFDI, thf): Calcd. for $[C_{30}H_{36}CrO_4P_2]^+$: 574,1488. Found: 574.1 $[M]^+$. The expected isotopic pattern agrees well with the measurement. Anal. Calcd. for $C_{30}H_{36}CrO_4P_2$ (574.56 g/mol): C 62.71, H 6.32. Found: C 62.15, H 6.22. IR (ATR, selected bands only): v (in cm^{-1}) = 1979, 1885, 1854, 1821.

Synthesis of complex 7

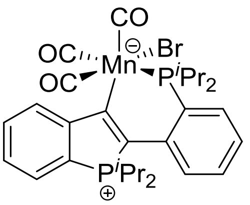

$MnBr(CO)_3(py)_2$[6] (398 µmol, 150 mg) and 1.0 eq **1** (398 µmol, 163 mg) were dissolved in thf (5 mL) and stirred at room temperature for 4 d. The resulting red solution was evaporated *in vacuo* to afford the product **7** as a red solid (247 mg, 99%). The product was obtained as a mixture of two isomers in a ratio of 4:1. ^{1}H and ^{31}P NMR spectra had to be recorded at -40°C because of dynamics in solution. In the ^{1}H NMR only the major species is assigned. Line broadening and the presence of two isomers interfered with an interpretation of the ^{13}C NMR data. ^{1}H NMR (CD_2Cl_2, 400 MHz, -40°C): δ (in ppm) = 9.57 (broad s,1H), 7.82 (broad s,1H), 7.53-7.47 (m, 2H), 7.31 (broad s,1H), 7.13 (broad s,1H), 3.13 (broad s,1H, CH), 3.01 (broad s,1H, CH), 2.87 (broad s,1H, CH), 2.65 (broad s,1H, CH), 1.44-0.60 (m, 24H). $^{31}P\{^{1}H\}$ NMR (CD_2Cl_2, 162 MHz, -40°C): δ (in ppm) = 62.4 (s, 1P, P-Mn, isomer 20%), 57.5 (d, $^{4}J_{P-P}$ = 9 Hz, 1P, P-Mn, isomer 80%), 45.8 (d, $^{4}J_{P-P}$ = 9 Hz, 1P, P-C, isomer 20%), 44.1 (d, $^{4}J_{P-P}$ = 9 Hz, 1P, P-C, isomer 80%). MS (LIFDI, thf): Calcd. for

$[C_{29}H_{36}BrMnO_3P_2]^+$: 628.0698. Found: 628.0 $[M]^+$. The expected isotopic pattern agrees well with the measurement. Anal. Calcd. for $C_{29}H_{36}BrMnO_3P_2$ (629.39 g/mol): C 55.34, H 5.77. Found: C 55.33, H 5.91. IR (ATR, selected bands only): *v* (in cm^{-1}) = 1992, 1920, 1883.

Synthesis of complex $[8]^+SbF_6^-$

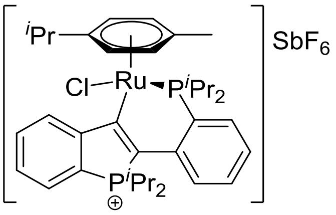

$[RuCl(p\text{-cymene})(\mu\text{-}Cl)]_2$ (348 µmol, 213 mg) and 2.1 eq **1** (731 µmol, 300 mg) were dissolved in CH_2Cl_2 (20 mL) and stirred at room temperature for 16. The solvent was removed *in vacuo*, and the residue was washed with pentane and dried *in vacuo* to afford the product **8** as a brown solid (480 mg, quant.). For crystallisation an aliquot of compound **8** in CH_2Cl_2 was treated with $AgSbF_6$. After 10 min AgCl was filtered off and the filtrate was layered with pentane. Single crystals suitable for x-ray diffraction were obtained after several days at-40°C. ^1H NMR (CD_2Cl_2, 600 MHz, 22°C): δ (in ppm) = 8.85 (dd, $^3J_{H\text{-}H}$ = 7.9 Hz, J = 2.8 Hz, 1H), 7.89-7.87 (m, 1H), 7.70-7.66 (m, 2H), 7.61-7.57 (m, 1H), 7.53-7.51 (m, 1H), 7.48-7.46 (m, 1H), 7.28 (dd, $^3J_{H\text{-}H}$ = 7.7 Hz, J = 4.2 Hz, 1H), 5.72 (dd, $^3J_{H\text{-}H}$ = 6.3 Hz, $^3J_{H\text{-}P}$ = 1.1 Hz, 1H), 5.59 (d, $^3J_{H\text{-}H}$ = 6.0 Hz, 1H), 5.45-5.43 (m, 1H), 5.24 (d, $^3J_{H\text{-}H}$ = 6.3 Hz, 1H), 3.46-3.39 (m, 1H, CH), 3.14-3.07 (m, 1H, CH), 2.80-2.74 (m, 1H, CH), 2.69-2.65 (m, 1H, CH), 2.37-2.31 (m, 1H, CH), 1.82 (dd, $^3J_{H\text{-}P}$ = 16.3 Hz, $^3J_{H\text{-}H}$ = 7.3 Hz, 3H, CH_3), 1.77 (dd, $^3J_{H\text{-}P}$ = 12.5 Hz, $^3J_{H\text{-}H}$ = 7.0 Hz, 3H, CH_3), 1.66 (dd, $^3J_{H\text{-}P}$ = 16.8 Hz, $^3J_{H\text{-}H}$ = 6.9 Hz, 3H, CH_3), 1.36 (dd, $^3J_{H\text{-}P}$ = 17.7 Hz, $^3J_{H\text{-}H}$ = 7.3 Hz, 3H, CH_3), 1.33 (dd, $^3J_{H\text{-}P}$ = 14.8 Hz, $^3J_{H\text{-}H}$ = 7.2 Hz, 3H, CH_3), 1.17-1.08 (m, 15H, CH_3), 0.66 (dd, $^3J_{H\text{-}P}$ = 14.3 Hz, $^3J_{H\text{-}H}$ = 7.5 Hz, 3H, CH_3). $^{13}C\{^1H, ^{31}P\}$ NMR (CD_2Cl_2, 151 MHz, 22°C): δ (in ppm) = 215.9 (C_q, 1C, C-Ru), 155.6 (C_q, 1C), 141.7 (C_q, 1C), 136.5 (CH, 1C), 135.2 (CH, 1C), 131.3 (CH, 1C), 129.4 (CH, 1C), 129.1 (CH, 1C), 128.8 (CH, 1C), 128.6 (CH, 1C), 127.9 (C_q, 1C), 127.4 (C_q, 1C), 120.8 (C_q, 1C, Cym), 120.0 (C_q, 1C), 117.0 (C_q, 1C), 96.9 (CH, 1C, Cym), 94.4 (broad, C_q, 1C, Cym), 91.2 (CH, 1C, Cym), 89.8 (CH, 1C,Cym), 88.4 (CH, 1C, Cym), 31.0 (CH, 1C, Cym), 29.2 (CH, 1C), 27.6 (CH, 1C), 26.0 (CH, 1C), 24.3 (CH, 1C), 23.1 (CH_3, 1C), 22.8 (CH_3, 1C), 21.9 (CH_3, 1C), 21.0 (CH_3, 1C), 20.7 (CH_3, 1C), 20.4 (CH_3, 1C), 17.6 (CH_3, 1C), 17.5 (CH_3, 1C), 16.9 (CH_3, 1C), 16.5 (CH_3, 1C), 16.4 (CH_3, 1C). $^{31}P\{^1H\}$ NMR (CD_2Cl_2, 162 MHz, 22°C): δ (in ppm) = 49.8 (s, 1P, P-C), 46.5 (d, $^4J_{P\text{-}P}$ = 4 Hz, 1P, P-Ru). HR-MS (ESI+, CH_2Cl_2): Calcd. for $[C_{36}H_{50}ClP_2Ru]^+$: 681.2114. Found: 681.2123 $[M]^+$. Anal. Calcd. for $C_{36}H_{50}Cl_2P_2Ru$ (716.71 g/mol): C 60.33, H 7.03. Found: C 60.61, H 7.09.

Synthesis of complex $[9]^+Cl^-$

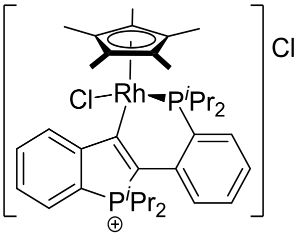

$[RhClCp^*(\mu\text{-}Cl)]_2$[7] (244 µmol, 151 mg) and 2.0 eq **1** (487 µmol, 200 mg) were dissolved in CH_2Cl_2 (20 mL) and stirred at room temperature for 2 h. The solvent was removed *in vacuo*, and the residue was washed with pentane and dried *in vacuo* to afford the product **9** as an orange solid (324 mg, 97%). ^1H NMR (CD_2Cl_2, 600 MHz, 22°C): δ (in ppm) = 8.57 (dd, $^3J_{H\text{-}H}$ = 7.9 Hz, J = 2.8 Hz, 1H), 7.88-7.86 (m, 1H), 7.81-7.78 (m, 1H), 7.77-7.75 (m, 1H), 7.66-7.63 (m, 1H), 7.62-7.59 (m, 1H), 7.58-7.55 (m, 1H), 7.52-7.50 (m, 1H), 4.43-4.35 (m, 1H, CH), 3.20-3.12 (m, 2H, CH), 3.11-3.02 (m, 2H, CH), 2.45-2.39 (m, 1H, CH), 1.78-1.72 (m, 6H, CH_3), 1.64 (dd, $^3J_{H\text{-}P}$ = 17.6 Hz, $^3J_{H\text{-}H}$ = 7.5 Hz, 3H, CH_3), 1.51 (dd, $^3J_{H\text{-}P}$ = 16.0 Hz, $^3J_{H\text{-}H}$ = 7.3 Hz, 3H, CH_3), 1.44 (dd, $^3J_{H\text{-}P}$ = 17.7 Hz, $^3J_{H\text{-}H}$ = 7.2 Hz, 3H, CH_3), 1.28 (d, $^3J_{H\text{-}Rh}$ = 2.7 Hz, 15H, $^{Cp*}CH_3$), 1.18 (dd, $^3J_{H\text{-}P}$ = 19.0 Hz, $^3J_{H\text{-}H}$ = 7.1 Hz, 3H, CH_3), 1.04 (dd, $^3J_{H\text{-}P}$ = 18.6 Hz, $^3J_{H\text{-}H}$ = 7.1 Hz, 3H, CH_3), 0.82 (dd, $^3J_{H\text{-}P}$ = 14.1 Hz, $^3J_{H\text{-}H}$ = 7.6 Hz, 3H, CH_3). $^{13}C\{^1H, ^{31}P\}$ NMR (CD_2Cl_2, 151 MHz, 22°C): δ (in ppm) = 208.3 (C_q, d, $^1J_{C\text{-}Rh}$ = 38 Hz, 1C, C-Rh), 152.6 (C_q, 1C), 140.9 (C_q, d, $^2J_{C\text{-}Rh}$ = 1 Hz, 1C), 135.2 (CH, 1C), 134.9 (CH, 1C), 131.9 (CH, 1C), 130.0 (CH, 1C), 129.8 (CH, 1C), 129.6 (CH, 1C), 128.5 (CH, 1C), 127.9 (CH, 1C), 126.6 (C_q, 1C), 121.8 (C_q, 1C), 119.1 (C_q, 1C), 101.7 (C_q, d, $^1J_{C\text{-}Rh}$ = 5 Hz, 5C, Cp*), 29.6 (CH, 1C), 25.8 (CH, 1C), 24.4 (CH, 1C), 24.3 (CH, 1C), 23.5 (CH_3, 1C), 21.4 (CH_3, 1C), 20.9 (CH_3, 1C), 20.4 (CH_3, 1C), 17.8 (CH_3, 1C), 17.5 (CH_3, 1C), 17.2 (CH_3, 1C), 16.2 (CH_3, 1C), 9.1 (CH_3, 5C, Cp*). $^{31}P\{^1H\}$ NMR (CD_2Cl_2, 243 MHz, 22°C): δ (in ppm) = 48.2 (dd, $^3J_{P\text{-}Rh}$ = 9 Hz $^4J_{P\text{-}P}$ = 5 Hz, 1P, P-C), 47.7 (dd, $^1J_{P\text{-}Rh}$ = 132 Hz $^4J_{P\text{-}P}$ = 5 Hz, 1P, P-Rh). HR-MS (ESI+, CH_2Cl_2): Calcd. for $[C_{36}H_{51}ClP_2Rh]^+$: 683,2204. Found: 683.2203 $[M]^+$. Anal. Calcd. for $C_{36}H_{51}Cl_2P_2Rh\bullet 0.5CH_2Cl_2$ (762.02 g/mol): C 57.49, H 6.94. Found: C 57.24, H 6.82. For crystallisation, an aliquot of compound **9** in CH_2Cl_2 was treated with $AgSbF_6$. After 10 min AgCl was filtered off and the filtrate was layered with pentane. Single crystals suitable for x-ray diffraction were obtained after several days at –40°C.

Synthesis of complex [10]$^+$Cl$^-$

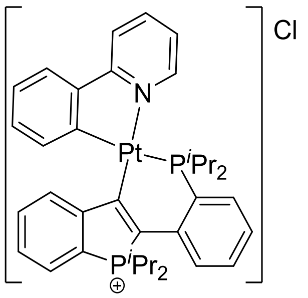

[Pt(ppy)(µ-Cl)]$_2$[8] (97.5 µmol, 75.0 mg) and 2.0 eq **1** (195 µmol, 80.0 mg) were dissolved in CH_2Cl_2 (5 mL) and stirred at room temperature for 7 h and at 60 °C for 1 d. The resulting solution was cooled to –40 °C, filtered through a PTFE syringe filter and the filtrate was evaporated *in vacuo.* Recrystallisation from thf/Et_2O afforded the product **10** as a dark green solid (148 mg, 95%). ^{1}H{^{31}P} NMR (CD_2Cl_2, 600 MHz, 22°C): *δ* (in ppm) = 8.23 (d, $^3J_{H\text{-}H}$ = 6.7 Hz, 1H), 8.05 (d, $^3J_{H\text{-}H}$ = 5.6 Hz, 1H), 7.90-7.84 (m, 5H), 7.75 (d, $^3J_{H\text{-}H}$ = 7.6 Hz, 1H), 7.64-7.59 (m, 2H), 7.54-7.50 (m, 2H), 7.43 (t, $^3J_{H\text{-}H}$ = 7.4 Hz, 1H), 7.28 (t, $^3J_{H\text{-}H}$ = 7.2 Hz, 1H), 7.21 (t, $^3J_{H\text{-}H}$ = 7.5 Hz, 1H), 6.86 (t, $^3J_{H\text{-}H}$ = 6.3 Hz, 1H), 4.04-4.01 (m, 1H), 3.64-3.55 (m, 2H), 2.52 (sept, $^3J_{H\text{-}H}$ = 7.2 Hz, 1H, CH), 1.77 (d, $^3J_{H\text{-}H}$ = 6.8 Hz, 3H, CH_3), 1.71 (d, $^3J_{H\text{-}H}$ = 7.2 Hz, 3H, CH_3), 1.41 (d, $^3J_{H\text{-}H}$ = 7.1 Hz, 3H, CH_3), 1.33 (d, $^3J_{H\text{-}H}$ = 6.9 Hz, 3H, CH_3), 1.25 (d, $^3J_{H\text{-}H}$ = 7.2 Hz, 6H, CH_3), 1.14-.12 (m, 6H, CH_3). ^{13}C{^{1}H, ^{31}P} NMR (CD_2Cl_2, 151 MHz, 22°C): *δ* (in ppm) = 198.8 (C_q, 1C, C-Pt), 168.7 (C_q, 1C), 168.5 (C_q, 1C), 156.0 (C_q, 1C), 149.5 (CH, 1C), 149.2 (C_q, 1C), 142.4 (C_q, 1C), 139.8 (CH, 1C), 135.0 (CH, 1C), 132.7 (CH, 1C), 132.3 (CH, 1C), 131.4 (CH, 1C), 131.2 (CH, 1C), 130.7 (CH, 1C), 129.6 (CH, 1C), 129.5 (CH, 1C), 128.0 (CH, 1C), 124.9 (CH, 1C), 124.5 (CH, 1C), 122.7 (C_q, 1C), 122.5 (CH, 1C), 121.1 (CH, 1C), 120.1 (C_q, 1C), 120.0 (CH, 1C), 78.1 (C_q, 1C, C-Pt), 28.5 (CH, 1C), 25.0 (CH, 1C), 23.5 (CH, 1C), 22.2 (CH, 1C), 19.5 (CH_3, 1C), 19.4 (CH_3, 1C), 19.0 (CH_3, 1C), 18.9 (CH_3, 1C), 17.7 (CH_3, 1C), 16.9 (CH_3, 1C), 16.9(CH_3, 1C), 16.7 (CH_3, 1C). ^{31}P{^{1}H} NMR (CD_2Cl_2, 243 MHz, 22°C): *δ* (in ppm) =53.2 (d, $^4J_{P\text{-}P}$ = 5 Hz, d, $^3J_{P\text{-}Pt}$ = 123 Hz, 1P, P-C), 29.6 (d, $^4J_{P\text{-}P}$ = 5 Hz, d, $^1J_{P\text{-}Pt}$ = 3926 Hz, 1P, P-Pt). HR-MS (ESI+, CH_3CN): Calcd. for $[C_{37}H_{44}NP_2Pt]^+$: 759.2591. Found: 759.2606 [M]$^+$. Anal. Calcd. for $C_{37}H_{44}ClNP_2Pt \cdot 2$ thf (939.46 g/mol): C 57.53, H 6.44, N 1.49. Found: C 57.79, H 6.46, N 1.48.

Synthesis of complex [11]$^+$BPh$_4^-$

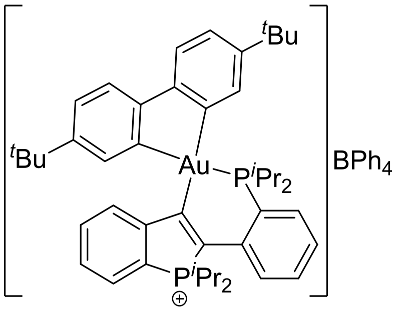

[Au(^{t}Bu-biphen)Cl]$_n$[9] (195 µmol, 100 mg) and 1.0 eq **1** (195 µmol, 80.0 mg) and 1.1 eq $NaBPh_4$ (221 µmol, 75.6 mg) were suspended in CH_2Cl_2 (5 mL) and stirred at room temperature for 18 h. The mixture was filtered through a PTFE syringe filter and the filtrate was evaporated *in vacuo*. The residue was washed with toluene and pentane and dried *in vacuo* to afford the product **11** as a green solid (120 mg, 52%). ^{1}H{^{31}P} NMR (CD_2Cl_2, 600 MHz, 22°C): *δ* (in ppm) = 8.07 (d, $^3J_{H\text{-}H}$ = 7.8 Hz, 1H), 7.96 (d, $^3J_{H\text{-}H}$ = 7.8 Hz, 1H), 7.75-7.73 (m, 2H), 7.66 (t, $^3J_{H\text{-}H}$ = 7.6 Hz, 1H), 7.62 (t, $^3J_{H\text{-}H}$ = 7.7 Hz, 1H), 7.56-7.52 (m, 2H), 7.43 (d, $^3J_{H\text{-}H}$ = 8.1 Hz, 1H), 7.38-7.36 (m, 2H), 7.34-7.32 (m, 8H, BPh_4), 7.27-7.25 (m, 1H), 7.12-7.10 (m, 1H), 7.03 (t, $^3J_{H\text{-}H}$ = 7.3 Hz, 8H, BPh_4), 6.88 (t, $^3J_{H\text{-}H}$ = 7.3 Hz, 4H, BPh_4), 6.77-6.76 (m, 1H), 3.83 (sept, $^3J_{H\text{-}H}$ = 7.3 Hz, 1H, CH), 3.44 (sept, $^3J_{H\text{-}H}$ = 7.0 Hz, 1H, CH), 2.92 (sept, $^3J_{H\text{-}H}$ = 7.2 Hz, 1H, CH), 2.83 (sept, $^3J_{H\text{-}H}$ = 7.3 Hz, 1H, CH), 1.83 (d, $^3J_{H\text{-}H}$ = 7.3 Hz, 3H, CH_3), 1.66 (d, $^3J_{H\text{-}H}$ = 7.0 Hz, 3H, CH_3), 1.42 (d, $^3J_{H\text{-}H}$ = 7.2 Hz, 3H, CH_3), 1.38 (d, $^3J_{H\text{-}H}$ = 7.0 Hz, 3H, CH_3), 1.38 (s, 9H, ^{t}Bu), 1.35 (d, $^3J_{H\text{-}H}$ = 7.2 Hz, 3H, CH_3), 1.27 (d, $^3J_{H\text{-}H}$ = 7.3 Hz, 3H, CH_3), 1.14 (d, $^3J_{H\text{-}H}$ = 7.3 Hz, 3H, CH_3), 1.06 (d, $^3J_{H\text{-}H}$ = 7.2 Hz, 3H, CH_3), 0.92 (s, 9H, ^{t}Bu). ^{13}C{^{1}H, ^{31}P} NMR (CD_2Cl_2, 151 MHz, 22°C): *δ* (in ppm) = 195.6 (C_q, 1C, C-Au), 164.4 (C_q, q, $^1J_{C\text{-}B}$ = 49 Hz, 4C, BPh_4), 163.5 (C_q, 1C), 162.4 (C_q, 1C), 153.8 (C_q, 1C), 153.3 (C_q, 1C), 152.9 (C_q, 1C), 150.2 (C_q, 1C), 149.1 (C_q, 1C), 141.7 (C_q, 1C), 136.3 (CH, q, $^3J_{C\text{-}B}$ = 1 Hz, 8C, BPh_4), 135.6 (CH, 1C), 135.1 (CH, 1C), 134.4 (CH, 1C), 133.4 (CH, broad, 1C), 133.3 (CH, 1C), 133.0 (CH, 1C), 131.1 (CH, 1C), 130.4 (CH, 1C), 129.9 (CH, 1C), 129.7 (CH, 1C), 126.0 (CH, q, $^2J_{C\text{-}B}$ = 3 Hz, 8C, BPh_4), 124.5 (CH, 1C), 124.4 (CH, 1C), 122.7 (C_q, 1C), 122.1 (CH, 4C, BPh_4), 121.6 (CH, 1C), 121.1 (C_q, broad, 1C), 121.0 (CH, 1C),120.0 (C_q, 1C), 35.3 (C_q, 1C, ^{t}Bu), 34.8 (C_q, 1C, ^{t}Bu), 31.7 (CH, 3C, ^{t}Bu), 31.2 (CH, 3C, ^{t}Bu), 27.6 (CH, 1C), 25.1 (CH, 1C), 24.3 (CH,1C), 21.7 (CH, 1C), 19.9 (CH_3, 1C), 19.4 (CH_3, 1C), 19.2 (CH_3, 1C), 19.0 (CH_3, 1C), 17.6 (CH_3, 1C), 17.3 (CH_3, 1C), 16.8 (CH_3, 1C), 16.5 (CH_3, 1C). ^{31}P{^{1}H} NMR (CD_2Cl_2, 243 MHz, 22°C): *δ* (in ppm) = 55.3 (d, $^4J_{P\text{-}P}$ = 10 Hz, 1P, P-C), 43.2 (d, $^4J_{P\text{-}P}$ = 10 Hz, 1P, P-Au). HR-MS (ESI+, CH_2Cl_2/MeOH): Calcd. for $[C_{46}H_{60}AuP_2]^+$: 871.3830. Found: 871.3828 [M]$^+$. Anal. Calcd. for $C_{70}H_{80}AuBP_2$ (1191.13 g/mol): C 70.59, H 6.77. Found: C 70.72, H 6.81.

Synthesis of complex 12

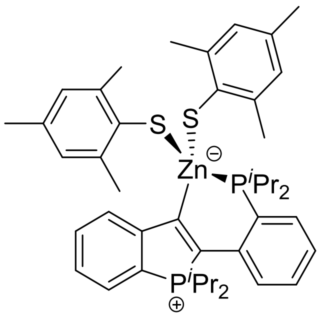

Zn(SMes)$_2$[10] (408 µmol, 150 mg) and 1.0 eq **1** (408 µmol, 167 mg) were dissolved in toluene (5 mL) and stirred at room temperature for 14 h. The resulting yellow solid was filtered off and washed with toluene. The powder was dried *in vacuo* to afford the product **12** as a yellow solid (282 mg, 89%). Recrystallisation from thf at -40°C gives yellow crystals of **12**•3thf suitable for x-ray diffraction. ^{1}H NMR (thf-d$_8$, 600 MHz, 40°C): *δ* (in ppm) = 7.75-7.73 (m, 1H), 7.53-7.48 (m, 2H), 7.37-7.33 (m, 1H), 7.28-7.24 (m, 2H), 7.20-7.17 (m, 1H), 7.06-7.04 (m, 1H), 6.44 (s, 4H, ^{Mes}CH), 3.20-3.14 (m, 2H), 2.61-2.56 (m, 2H), 2.15 (s, 12H, $^{Mes}CH_3$), 2.00 (s, 6H, $^{Mes}CH_3$), 1.27-1.16 (m, 20H, $^{Pr}CH_3$), 0.87-0.85 (m, 3H, $^{Pr}CH_3$). Fast decoordination of the ligand (and cyclisation of the liberated ligand) in solution interfered with our attempts to record ^{13}C NMR and mass spectra. ^{31}P{^{1}H} NMR ($C_6D_5NO_2$, 162 MHz, 22°C): *δ* (in ppm) =55.0 (s, 1P, P-C), –7.9 (s, 1P, decoordinated). Anal. Calcd. for $C_{44}H_{58}P_2S_2Zn$ · 3 thf (994.72 g/mol): C 67.62, H 8.31. Found: C 67.99, H 8.13.

Synthesis of complex 13

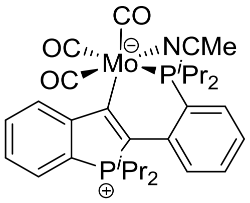

Mo(CO)$_3$(NCMe)$_3$[11] (660 µmol, 200 mg) and 1.0 eq **1** (660 µmol, 271 mg) were dissolved in CH_3CN (10 mL) and stirred at room temperature for 18 h. The resulting dark solution was evaporated *in vacuo* and the solid was washed with Et_2O. Recrystallisation from thf/Et_2O afforded the product **13** as a dark violet solid (248 mg, 60%). As the low solubility of the product does not allow to measure NMR spectra in acetonitrile, thf had to be used. The fast formation of complex **14** in thf solution made it impossible to measure a ^{13}C NMR spectrum of **13**. ^{1}H NMR (thf-d$_8$/CD_3CN 4:1, 600 MHz, 22°C): *δ* (in ppm) = 8.90-8.89 (m, 1H), 7.73 (t, $^3J_{H-H}$ = 7.7 Hz, 1H), 7.56 (t, $^3J_{H-H}$ = 6.5 Hz, 1H), 7.48 (bt, $^3J_{H-H}$ = 6.5 Hz, 1H), 7.36-7.30 (m, 2H), 7.19-7.17 (m, 1H), 7.14 (d, $^3J_{H-H}$ = 7.4 Hz, 1H), 3.25-3.19 (m, 1H, CH), 2.98-2.92 (m, 1H, CH), 2.61-2.57 (m, 1H, CH), 2.22-2.20 (m, 1H, CH), 1.95 (s, 3H, CH_3CN), 1.42-1.38 (m, 3H, CH_3), 1.17-0.97 (m, 21H, CH_3). ^{31}P{^{1}H} NMR (thf-d$_8$/CD_3CN 4:1, 243 MHz, 22°C): *δ* (in ppm) = 49.6 (s, 1P, P-Mo), 43.0 (d, $^4J_{P-P}$ = 9 Hz, 1P, P-C). By mass spectrometry, a fragment [M-CH_3CN]$^+$ was detected. Although this is in line with the molecular formula of **13**, we cannot exclude ring opening to **14** during measurement. IR (ATR, selected bands only): *v* (in cm^{-1}) = 1994, 1887, 1790, 1751. (As judged by DFT calculations, CO stretching and MeCN stretching vibrations are partially coupled). Anal. Calcd. for $C_{31}H_{39}MoNO_3P_2$ (631.56 g/mol): C 58.96, H 6.22, N 2.22. Found: C 58.61, H 6.09, N 2.67.

Synthesis of complex 14

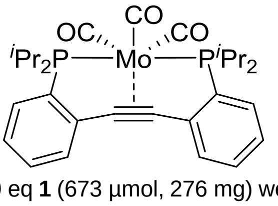

Mo(CO)$_3$(NCMe)$_3$[11] (660 µmol, 200 mg) and 1.0 eq **1** (673 µmol, 276 mg) were dissolved in thf (10 mL) and stirred at room temperature for 1.5 h. The resulting red solution was concentrated and stored at -40°C for 14 h. The solid was decanted, washed with Et_2O and dried *in vacuo* to afford the product **14** as a red solid (335 mg, 86%). ^{1}H{^{31}P} NMR (thf-d$_8$, 400 MHz, 22°C): *δ* (in ppm) = 7.71 (d, $^3J_{H-H}$ = 7.6 Hz, 2H), 7.68 (d, $^3J_{H-H}$ = 7.8 Hz, 2H), 7.38 (t, $^3J_{H-H}$ = 7.6 Hz, 2H), 7.28 (t, $^3J_{H-H}$ = 7.5 Hz, 2H), 2.94 (sept, $^3J_{H-H}$ = 7.0 Hz, 2H, CH), 1.98 (sept, $^3J_{H-H}$ = 7.1 Hz, 2H, CH), 1.33 (d, $^3J_{H-H}$ = 7.0 Hz, 6H, CH_3), 1.26 (d, $^3J_{H-H}$ = 7.1 Hz, 6H, CH_3), 1.16 (d, $^3J_{H-H}$ = 7.1 Hz, 6H, CH_3), 0.92 (d, $^3J_{H-H}$ = 7.0 Hz, 6H, CH_3). ^{13}C{^{1}H, ^{31}P} NMR (thf-d$_8$, 151 MHz, 22°C): *δ* (in ppm) = 225.8 (C_q, 1C, CO), 219.7 (C_q, 2C, CO), 145.4 (C_q, 2C), 139.5 (C_q, 2C), 131.5 (CH, 2C), 129.9 (CH, 2C), 129.9 (CH, 2C), 127.1 (CH, 2C), 99.4 (C_q, 2C, C-Mo), 30.2 (CH, 2C), 25.8 (CH, 2C), 20.6 (CH_3, 2C), 19.7 (CH_3, 2C), 19.0 (CH_3, 2C) , 18.6 (CH_3, 2C). ^{31}P{^{1}H} NMR (thf-d$_8$, 162 MHz, 22°C): *δ* (in ppm) = 54.3 (s, 1P). MS (EI+, thf): Calcd. for $C_{29}H_{36}MoO_3P_2$: 592.1194. Found: 592.1 [M]$^+$. The expected isotopic pattern agrees well with the measurement. IR (ATR, selected bands only): *v* (in cm^{-1}) = 1930, 1847, 1829, 1818. (As judged by DFT calculations, CO stretching and alkyne stretching vibrations are partially coupled). Anal. Calcd. for $C_{29}H_{36}MoO_3P_2$ (590.51 g/mol): C 58.99, H 6.15. Found: C 58.91, H 6.01.

Synthesis of complex 15

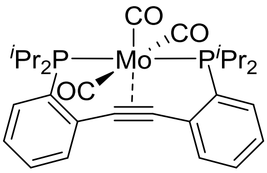

The *fac*-isomer **14** (169 µmol, 100 mg) was dissolved in benzene (5 mL) and stirred at 80°C for 4.5 h. The red solution was filtered through a PTFE syringe filter and evaporated *in vacuo* to afford the product **15** as an orange solid (98.6 mg, 99%). ^1H NMR (thf-d_8, 600 MHz, 22°C): δ (in ppm) = 8.00 (d, $^3J_{H\text{-}H}$ = 7.7 Hz, 2H), 7.85-7.83 (m, 2H), 7.46 (t, $^3J_{H\text{-}H}$ = 7.4 Hz, 2H), 7.34 (t, $^3J_{H\text{-}H}$ = 7.4 Hz, 2H), 2.84-2.76 (m, 4H, CH), 1.44 (dd, $^3J_{H\text{-}H}$ = 15.3 Hz, $^3J_{H\text{-}H}$ = 6.8 Hz, 12H, CH_3), 1.00 (dd, $^3J_{H\text{-}H}$ = 13.9 Hz, $^3J_{H\text{-}H}$ = 7.1 Hz, 12H, CH_3). $^{13}C\{^1H\}$ NMR (thf-d_8, 151 MHz, 22°C): δ (in ppm) = 225.2 (C_q, 1C, CO), 210.4 (C_q, 2C, CO), 155.7 (C_q, 2C), 139.6 (C_q, 2C), 131.8 (CH, 2C), 131.5 (CH, 2C), 130.3 (CH, 2C), 126.7 (CH, 2C), 110.6 (C_q, 2C, C-Mo), 32.5 (CH, 4C), 20.9 (CH_3, 4C), 20.2 (CH_3, 4C). $^{31}P\{^1H\}$ NMR (C_6D_6, 162 MHz, 22°C): δ (in ppm) = 87.5 (s, 1P). MS (EI+): Calcd. for $C_{28}H_{36}MoO_2P_2$: 564.1239. Found: 564.1 $[M\text{-}CO]^+$. The expected isotopic pattern agrees well with the measurement. IR (ATR, selected bands only): v (in cm^{-1}) = 1974, 1946, 1865, 1837. (As judged by DFT calculations, CO stretching and alkyne stretching vibrations are partially coupled). Anal. Calcd. for $C_{29}H_{36}MoO_3P_2$ (590.51 g/mol): C 58.99, H 6.15. Found: C 59.43, H 6.08.

Synthesis of complex 16

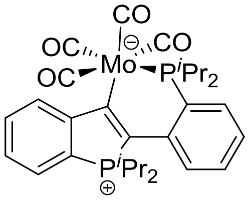

$Mo(CO)_4(pip)_2$[12] (793 µmol, 300 mg) and 1.0 eq **1** (793 µmol, 326 mg) were dissolved in thf (15 mL) and stirred at room temperature for 18 h. The resulting red solution was evaporated *in vacuo* to afford the product **16** as a dark red solid (472 mg, 96%). This complex may as well be synthesised in quantitative yield by pressurising a solution of **13** or **14** with CO. $^1H\{^{31}P\}$ NMR (CD_2Cl_2, 600 MHz, 22°C): δ (in ppm) = 8.74 (d, $^3J_{H\text{-}H}$ = 7.9 Hz, 1H), 7.83 (t, $^3J_{H\text{-}H}$ = 7.7 Hz, 1H), 7.50 (d, $^3J_{H\text{-}H}$ = 7.7 Hz, 1H), 7.48 (d, $^3J_{H\text{-}H}$ = 7.2 Hz, 1H), 7.41-7.37 (m, 2H), 7.23 (t, $^3J_{H\text{-}H}$ = 7.5 Hz, 1H), 7.09 (d, $^3J_{H\text{-}H}$ = 7.7 Hz, 1H), 2.91 (sept, $^3J_{H\text{-}H}$ = 7.1 Hz, 2H, CH), 2.37 (sept, $^3J_{H\text{-}H}$ = 6.9 Hz, 2H, CH), 1.26 (d, $^3J_{H\text{-}H}$ = 6.9 Hz, 6H, CH_3), 1.13 (d, $^3J_{H\text{-}H}$ = 6.9 Hz, 6H, CH_3), 1.11 (d, $^3J_{H\text{-}H}$ = 7.1 Hz, 6H, CH_3), 1.04 (d, $^3J_{H\text{-}H}$ = 7.1 Hz, 6H, CH_3). $^{13}C\{^1H, ^{31}P\}$ NMR (CD_2Cl_2, 151 MHz, 22°C): δ (in ppm) = 236.7 (C_q, 1C, C-Mo), 224.3 (C_q, 1C, CO), 221.7 (C_q, 1C, CO), 213.2 (C_q, 2C, CO), 159.3 (C_q, 1C), 145.4 (C_q, 1C), 136.6 (CH, 1C), 134.5 (CH, 1C), 132.0 (CH, 1C), 129.6 (CH, 1C), 129.3 (CH, 1C), 128.9 (C_q, 1C), 127.6 (CH, 1C), 127.3 (CH, 1C), 125.4 (CH, 1C), 122.5 (C_q, 1C), 116.7 (C_q, 1C), 25.5 (CH, 2C), 24.8 (CH, 2C), 18.5 (CH_3, 2C), 18.3 (CH_3, 2C), 17.3 (CH_3, 2C) , 16.6 (CH_3, 2C). $^{31}P\{^1H\}$ NMR (CD_2Cl_2, 243 MHz, 22°C): δ (in ppm) = 51.0 (d, $^4J_{P\text{-}P}$ = 9 Hz, 1P, P-Mo), 46.0 (d, $^4J_{P\text{-}P}$ = 9 Hz, 1P, P-C). MS (LIFDI, thf): Calcd. for $[C_{30}H_{36}MoO_4P_2]^+$: 620.1137. Found: 620.1 $[M]^+$. The expected isotopic pattern agrees well with the measurement. IR (ATR, selected bands only): v (in cm^{-1}) = 1987, 1892, 1855, 1826. Anal. Calcd. for $C_{30}H_{36}MoO_4P_2$ (618.52 g/mol): C 58.26, H 5.87. Found: C 58.07, H 5.70.

Synthesis of complex 16-W

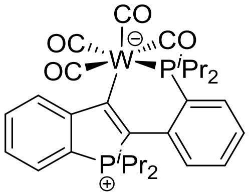

$W(CO)_4(pip)_2$[12] (644 µmol, 300 mg) and 1.0 eq **1** (644 µmol, 264 mg) were dissolved in thf (10 mL) and stirred at room temperature for 2 d. The resulting red solution was filtered through a PTFE syringe filter and evaporated *in vacuo*. The resulting brownish solid was washed with Et_2O and dried *in vacuo* to afford the product **17** as a dark red solid (220 mg, 48%). $^1H\{^{31}P\}$ NMR (CD_2Cl_2, 600 MHz, 22°C): δ (in ppm) = 8.69 (d, $^3J_{H\text{-}H}$ = 7.9 Hz, 1H), 7.84 (t, $^3J_{H\text{-}H}$ = 7.6 Hz, 1H), 7.51 (d, $^3J_{H\text{-}H}$ = 7.6 Hz, 1H), 7.47 (d, $^3J_{H\text{-}H}$ = 7.1 Hz, 1H), 7.42 (t, $^3J_{H\text{-}H}$ = 7.3 Hz, 1H), 7.38 (t, $^3J_{H\text{-}H}$ = 7.3 Hz, 1H), 7.26 (t, $^3J_{H\text{-}H}$ = 7.5 Hz, 1H), 7.09 (d, $^3J_{H\text{-}H}$ = 7.8 Hz, 1H), 2.92 (sept, $^3J_{H\text{-}H}$ = 7.1 Hz, 2H, CH), 2.38 (sept, $^3J_{H\text{-}H}$ = 6.9 Hz, 2H, CH), 1.26 (d, $^3J_{H\text{-}H}$ = 6.9 Hz, 6H, CH_3), 1.12 (d, $^3J_{H\text{-}H}$ = 7.1 Hz, 6H, CH_3), 1.11 (d, $^3J_{H\text{-}H}$ = 7.1 Hz, 6H, CH_3), 1.03 (d, $^3J_{H\text{-}H}$ = 6.9 Hz, 6H, CH_3). $^{13}C\{^1H, ^{31}P\}$ NMR (CD_2Cl_2, 151 MHz, 22°C): δ (in ppm) = 224.7 (C_q, 1C, C-W), 215.1 (C_q, 1C, CO), 213.5 (C_q, 1C, CO), 205.1 (C_q, 2C, CO), 160.1 (C_q, 1C), 146.2 (C_q, 1C), 137.6 (CH, 1C), 134.7 (CH, 1C), 132.0 (CH, 1C), 130.0 (CH, 1C), 129.0 (CH, 1C), 128.3 (C_q, 1C), 127.8 (CH, 1C), 127.4 (CH, 1C), 125.5 (CH, 1C), 122.6 (C_q, 1C), 116.7 (C_q, 1C), 26.0 (CH, 2C), 24.8 (CH, 2C), 18.6 (CH_3, 2C), 18.4 (CH_3, 2C), 17.2 (CH_3, 2C), 16.7 (CH_3, 2C). $^{31}P\{^1H\}$ NMR (CD_2Cl_2, 243 MHz, 22°C): δ (in ppm) = 46.6 (d, $^4J_{P\text{-}P}$ = 9 Hz, d, $^3J_{P\text{-}W}$ = 18 Hz, 1P, P-C), 34.2 (d, $^4J_{P\text{-}P}$ = 9 Hz, d, $^1J_{P\text{-}W}$ = 217 Hz, 1P, P-W). MS (LIFDI, thf): Calcd. for $[C_{30}H_{36}O_4P_2W]^+$: 706.1593. Found: 706.1 $[M]^+$. The expected isotopic pattern agrees well with the measurement. Anal. Calcd. for $C_{30}H_{36}O_4P_2W$ (706.40 g/mol): C 51.01, H 5.14. Found: C 51.27, H 5.07. IR (ATR, selected bands only): v (in cm^{-1}) = 1983, 1887, 1846, 1820.

NMR Spectra

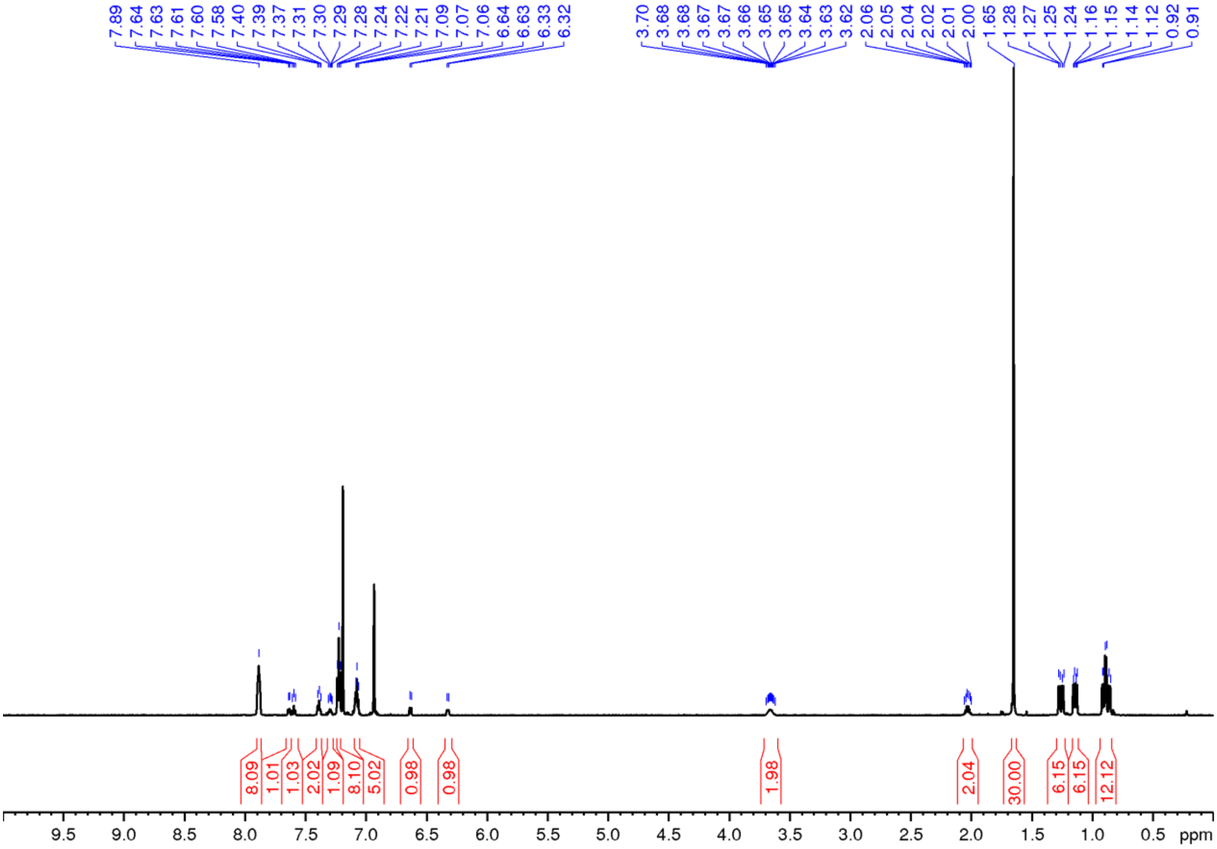

Figure S1: ^{1}H NMR spectrum of **[3]$^{+}$BPh$_4$$^{-}$** (*o*-$C_6D_4Cl_2$, 600 MHz, 22°C).

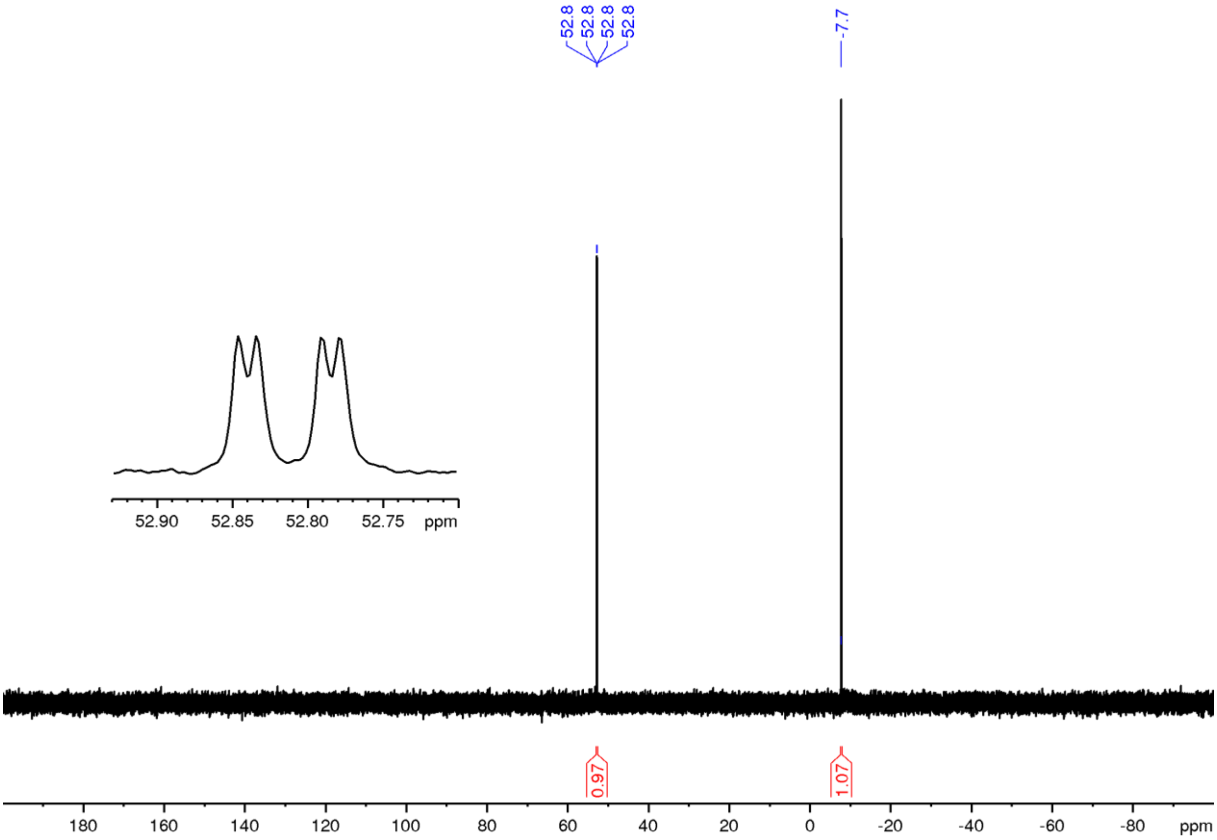

Figure S2: ^{31}P{^{1}H} NMR spectrum of **[3]$^{+}$BPh$_4$$^{-}$** (*o*-$C_6D_4Cl_2$, 243 MHz, 22°C).

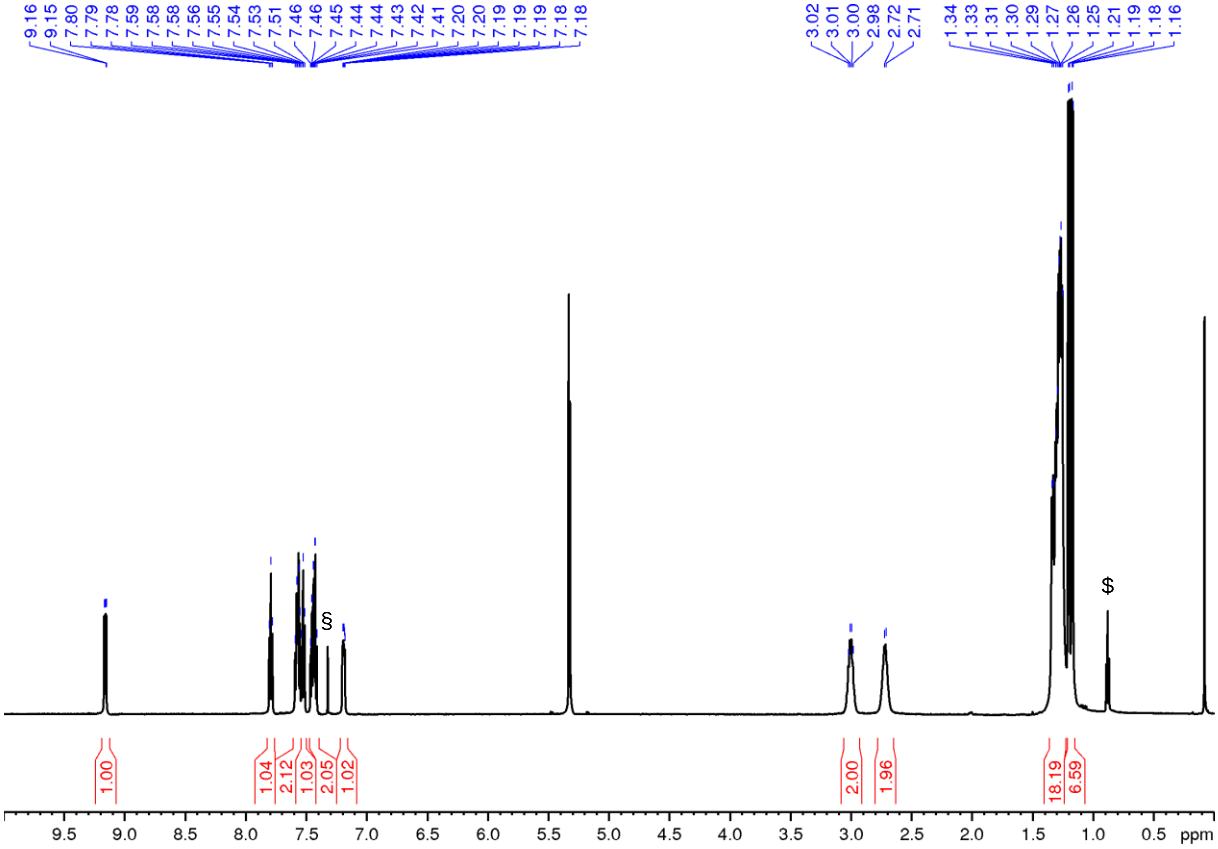

Figure S3: ^{1}H NMR spectrum of **4** (CD_2Cl_2, 600 MHz, 22°C). Residual solvent signals from washing the crystals with $CHCl_3$ and pentane are labelled § and $, respectively. Note that most of the product in the NMR tube is undissolved, i.e. the actual amounts of residual solvents are lower than implied by the relative signal intensities.

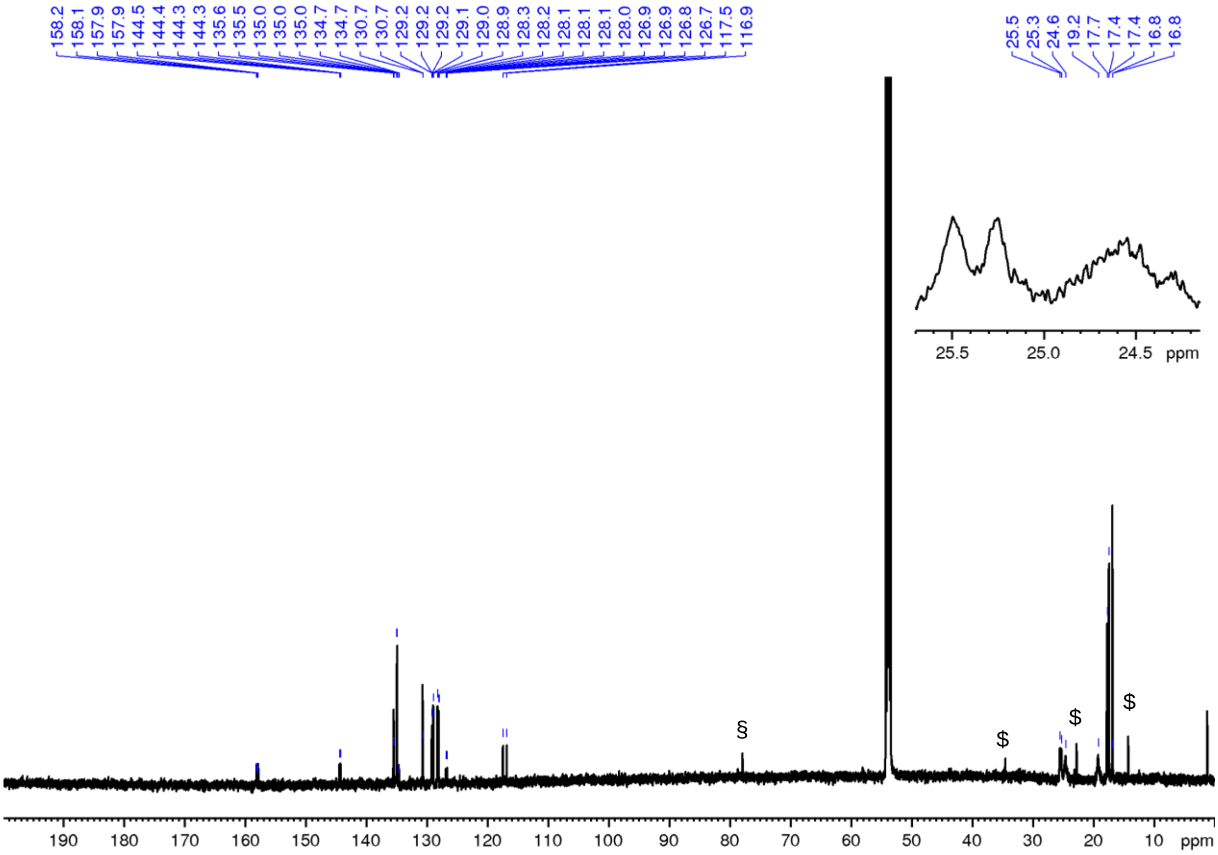

Figure S4: $^{13}C\{^{1}H\}$ NMR spectrum of **4** (CD_2Cl_2, 151 MHz, 22°C). Residual solvent signals from washing the crystals with $CHCl_3$ and pentane are labelled § and $, respectively. Note that most of the product in the NMR tube is undissolved, i.e. the actual amounts of residual solvents are lower than implied by the relative signal intensities.

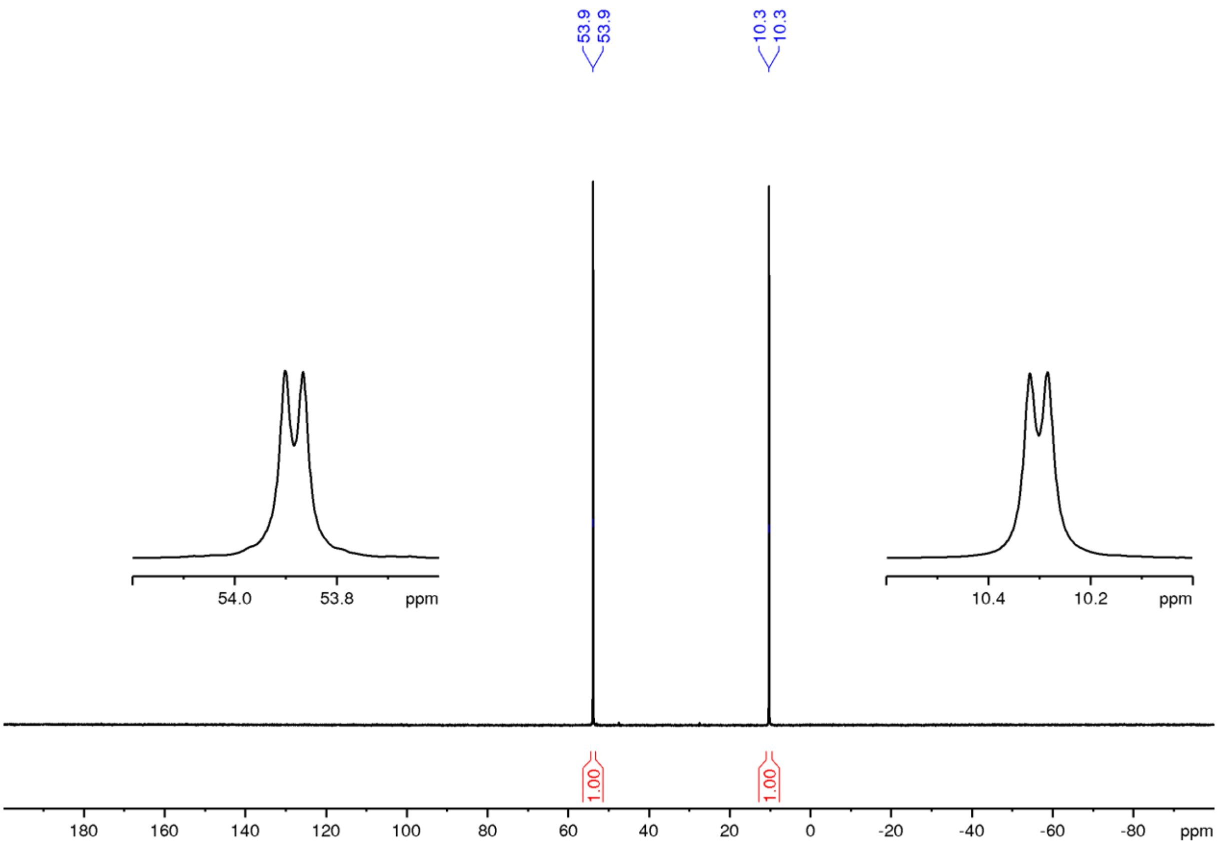

Figure S5: $^{31}P\{^{1}H\}$ NMR spectrum of **4** (CD_2Cl_2, 243 MHz, 22°C).

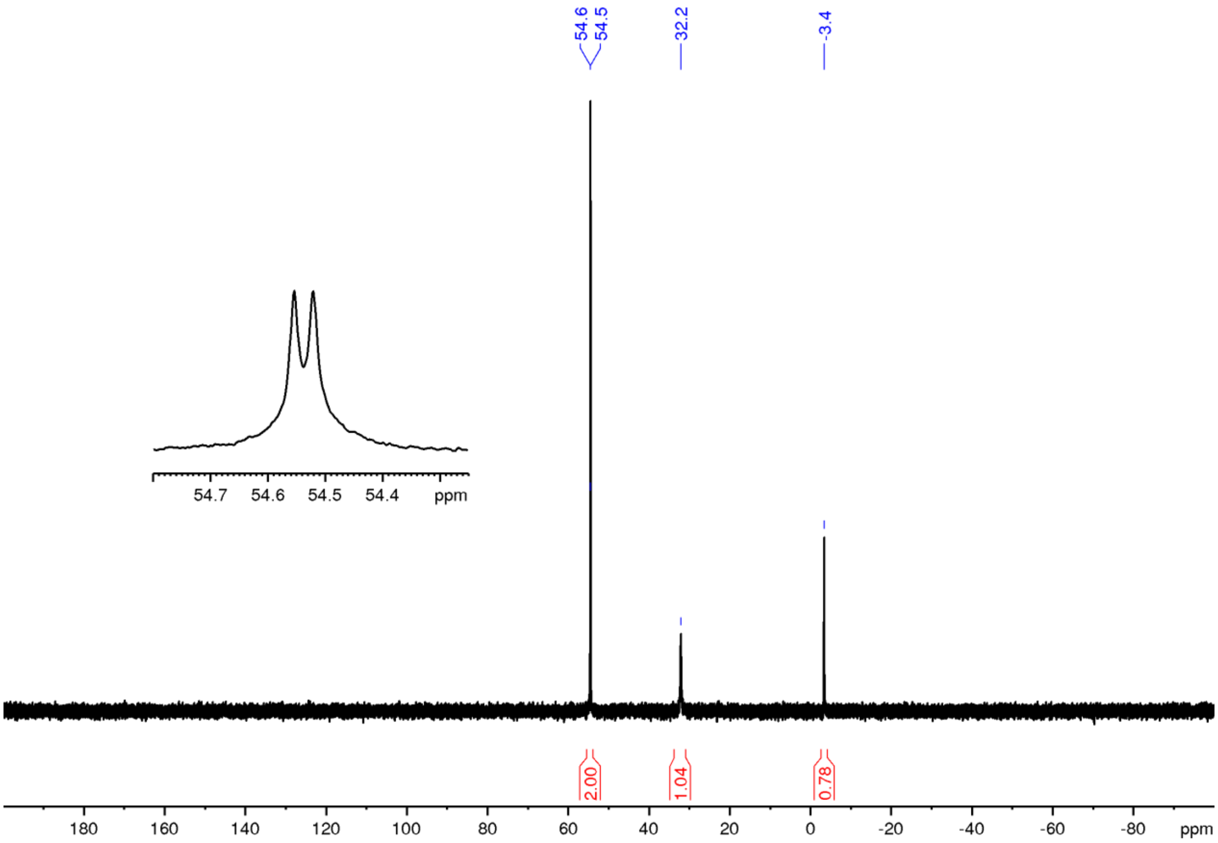

Figure S6: $^{31}P\{^{1}H\}$ NMR spectrum of **5** (CD_2Cl_2, 243 MHz, 22°C). Overall four signals are expected due to the presence of two isomers (with a coordinated and a decoordinated phosphine, see article text and experimental procedure). At r.t., only three signals are detected (an exchange broadened signal is buried below the doublet at 54.5 ppm). At -35°C (see Figure S7), all four signals have been observed.

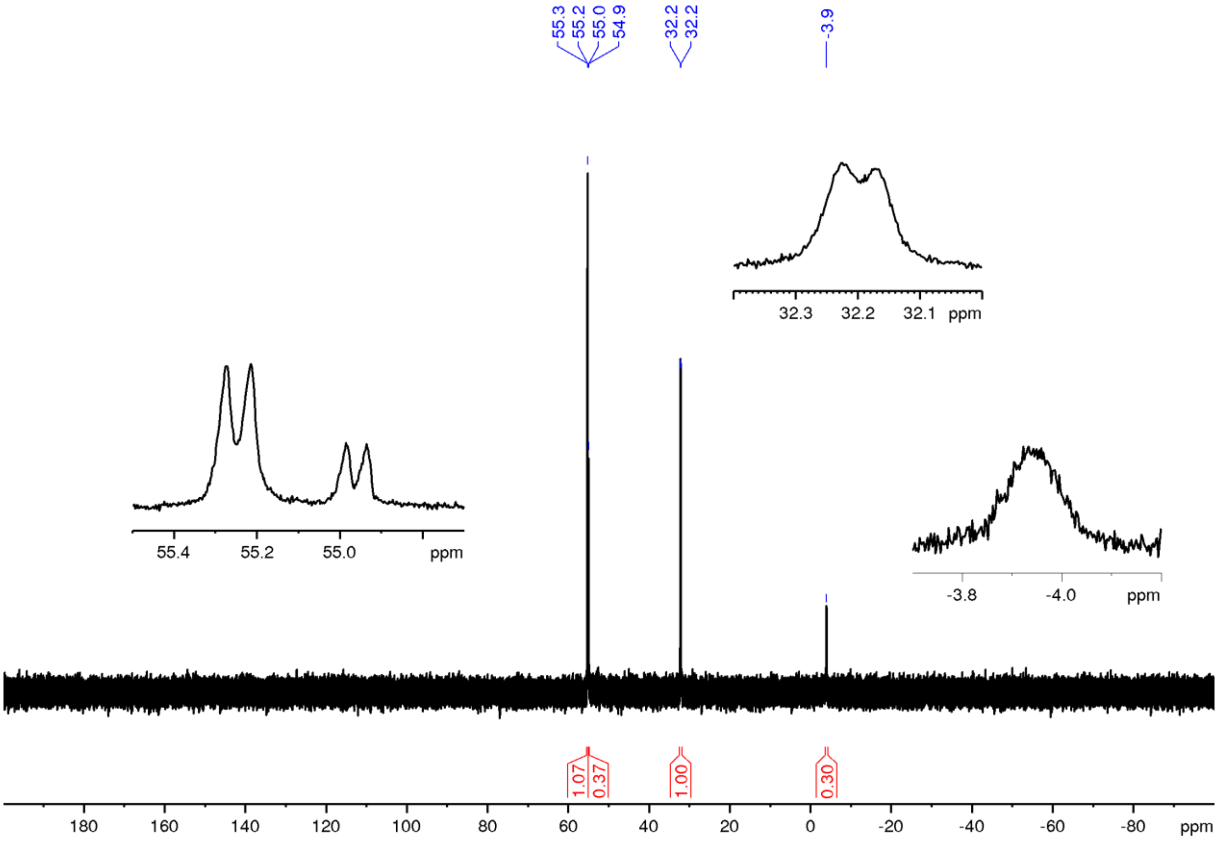

Figure S7: $^{31}P\{^{1}H\}$ NMR spectrum of **5** (CH_2Cl_2, 162 MHz, -35°C). Overall four signals are expected (cf. caption of Figure S6) and indeed observed at -35°C. The two signals with an integral value of approximately 1 correspond to the κ^2-*P*,*C*-coordinated compound. The two signals with an integral value of approximately 0.3 correspond to the species with a decoordinated phosphine. Upon lowering the temperature (from r.t. to -35°C), the latter signals decrease indicative of a coordination of the phosphine at lower temperatures. Below -50°C, however, the compound precipitates from solution as indicated by a very low signals to noise ratio at -50°C.

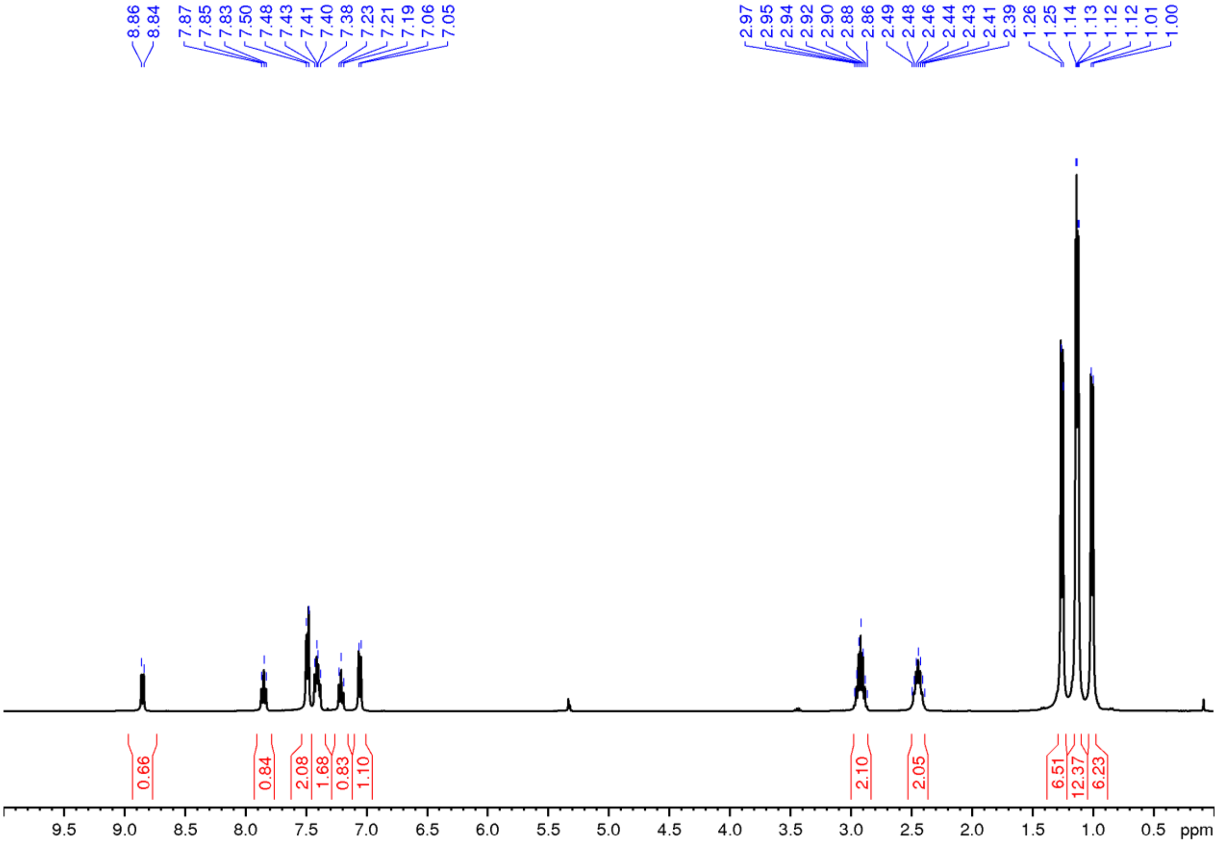

Figure S8: $^{1}H\{^{31}P\}$ NMR spectrum of **6** (CD_2Cl_2, 400 MHz, 22°C).

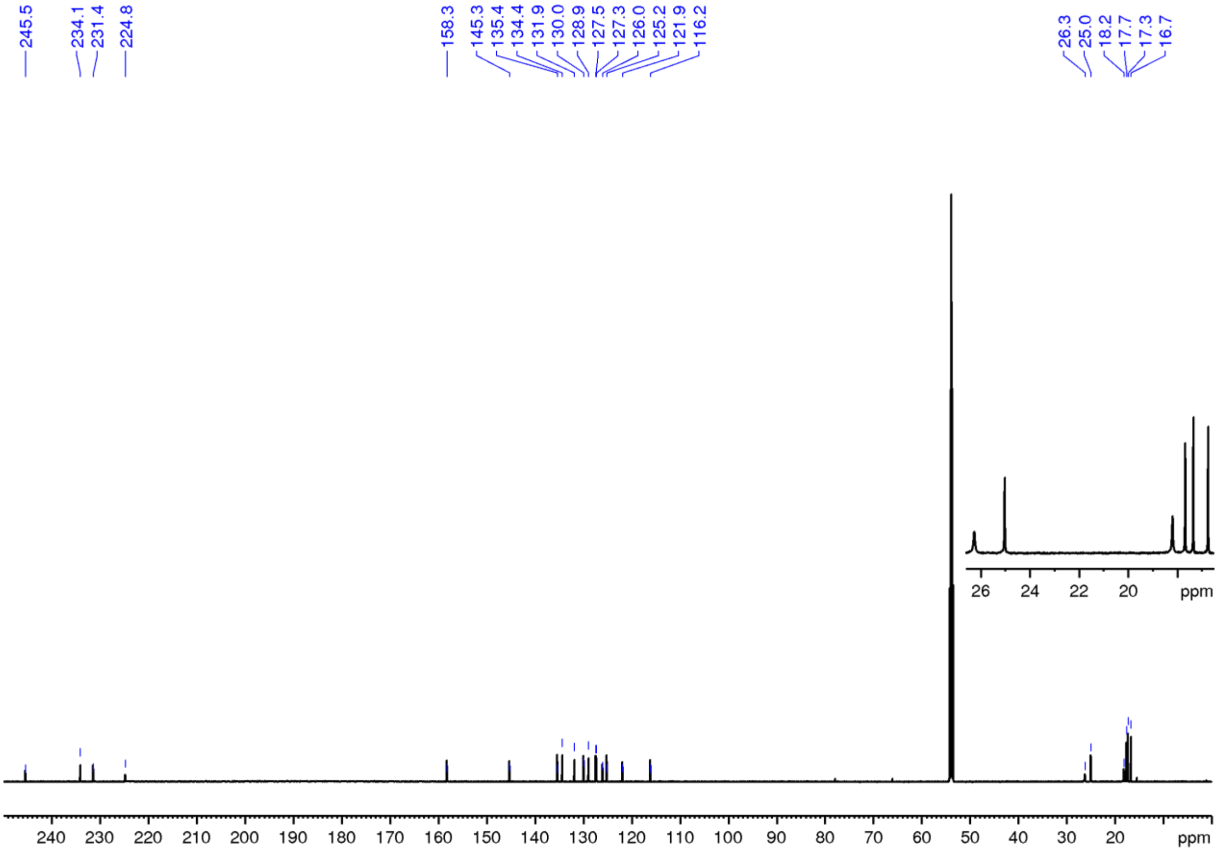

Figure S9: $^{13}C\{^{1}H, ^{31}P\}$ NMR spectrum of **6** (CD_2Cl_2, 151 MHz, 22°C).

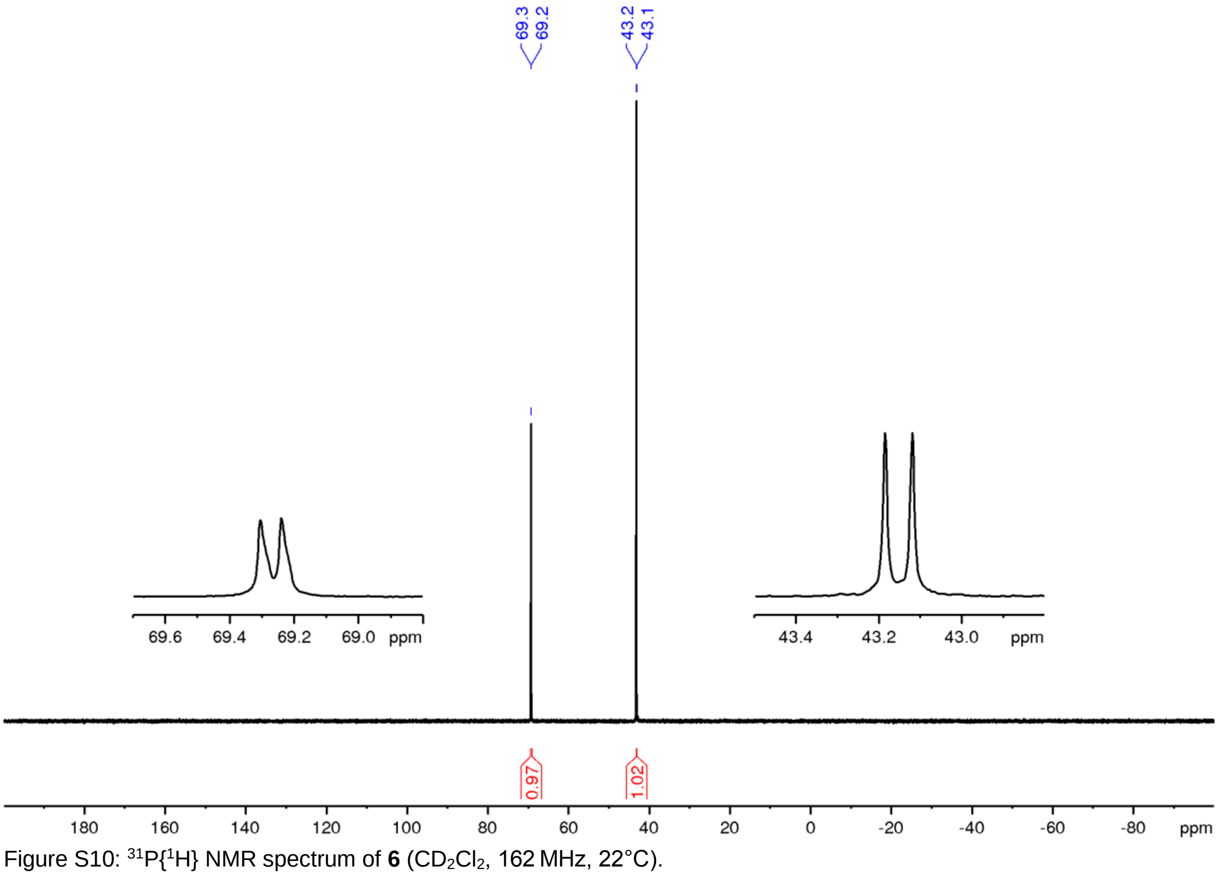

Figure S10: $^{31}P\{^{1}H\}$ NMR spectrum of **6** (CD_2Cl_2, 162 MHz, 22°C).

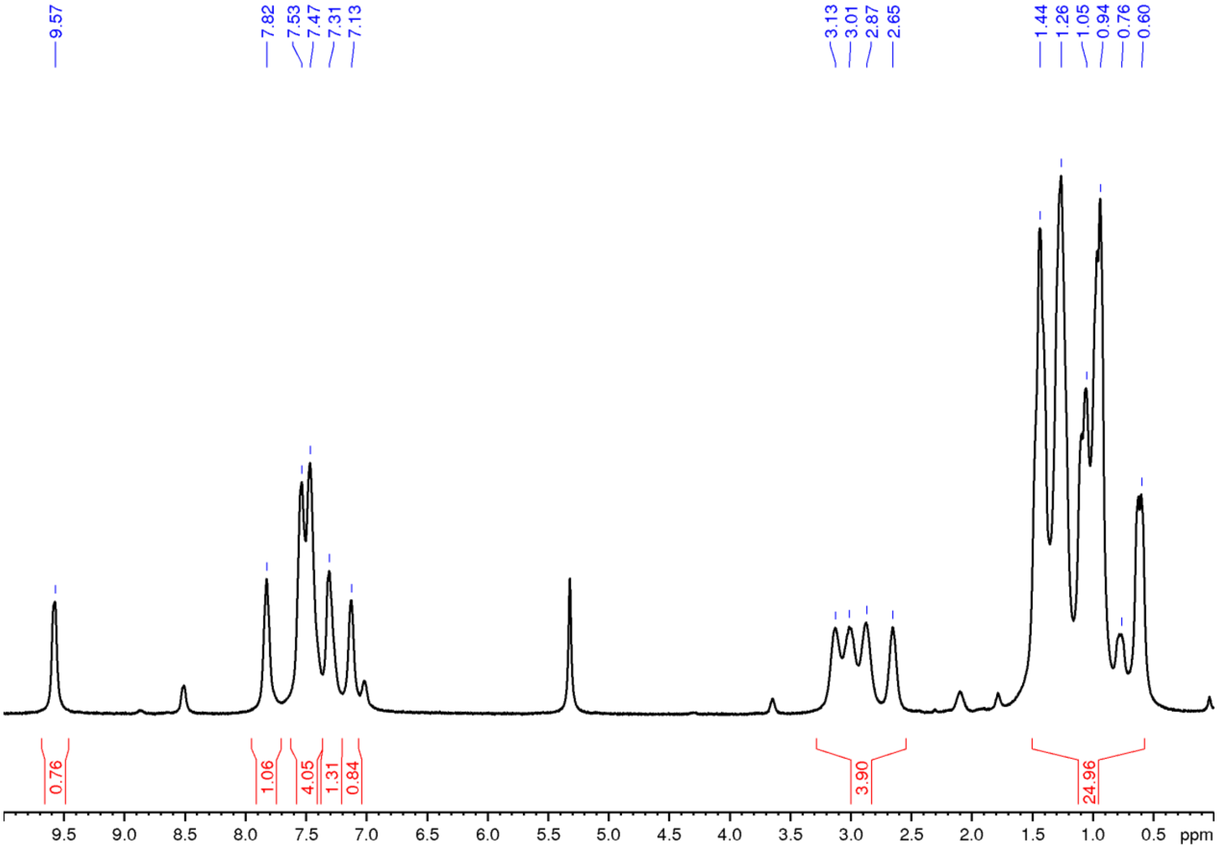

Figure S11: ^{1}H NMR spectrum of **7** (CD_2Cl_2, 400 MHz, –40°C). Broadened signals were detected due to the presence of two isomers (with different bromide orientations, see article text, see ^{31}P NMR spectrum of **7** in the following figure).

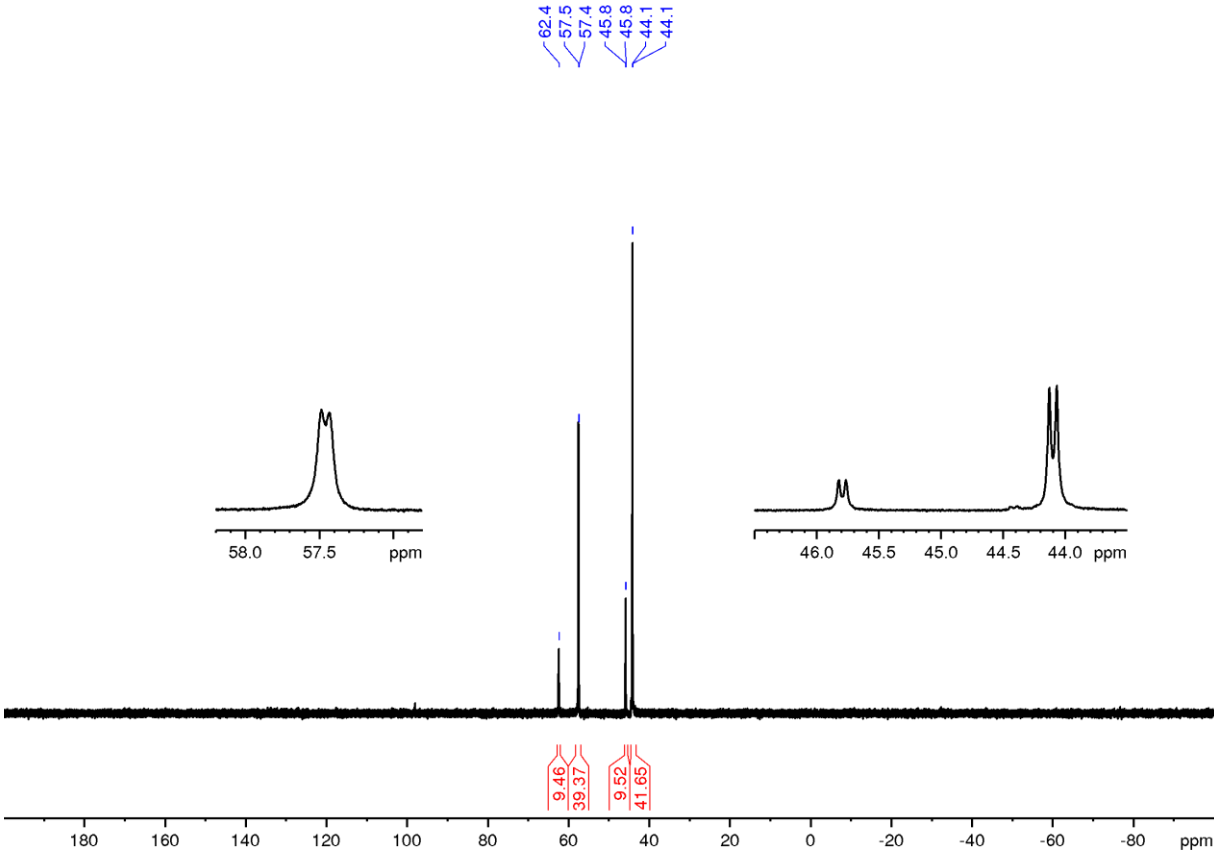

Figure S12: $^{31}P\{^{1}H\}$ NMR spectrum of 7 (CD_2Cl_2, 162 MHz, –40°C). Overall four signals were detected due to the presence of two isomers in a 4:1 ration (see article text and experimental procedure).

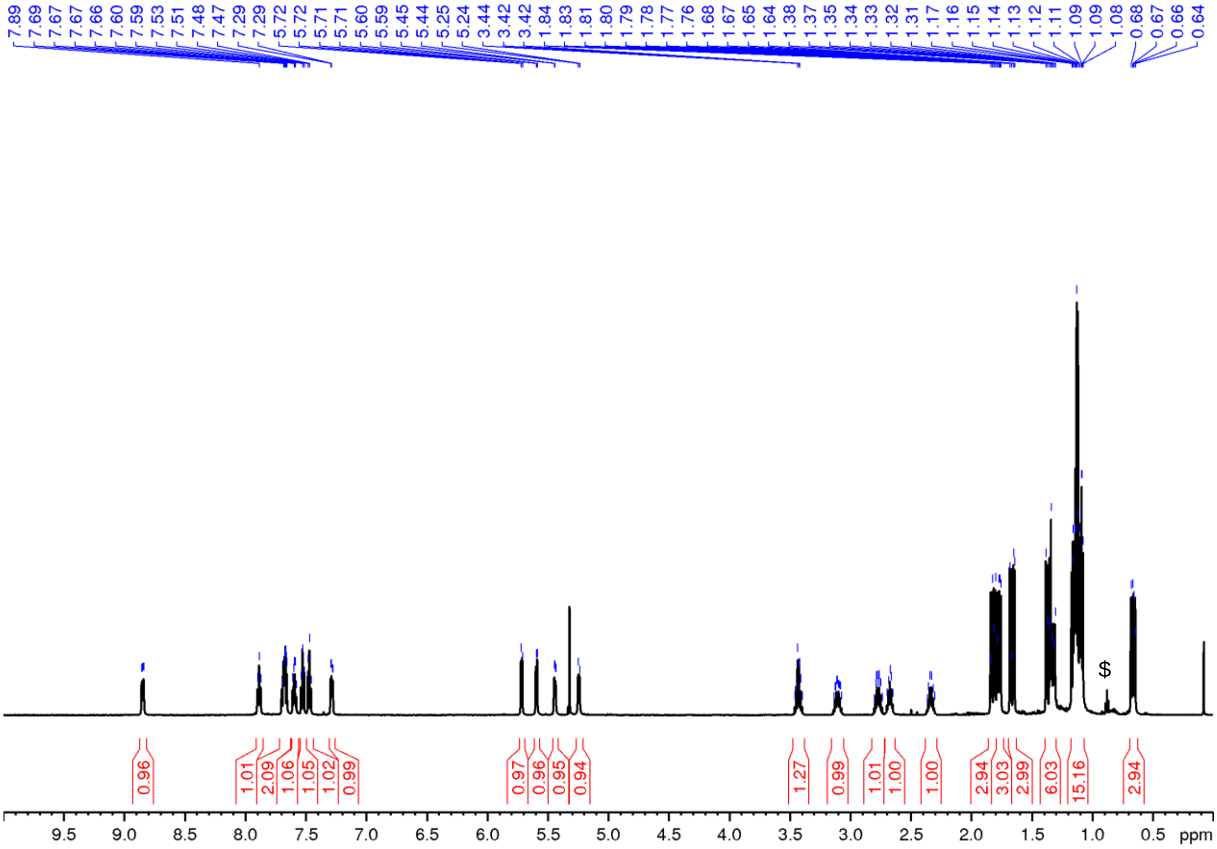

Figure S13: ^{1}H NMR spectrum of **[8]$^{+}$SbF$_6^{-}$** (CD_2Cl_2, 600 MHz, 22°C). Residual pentane from washing is labelled with $.

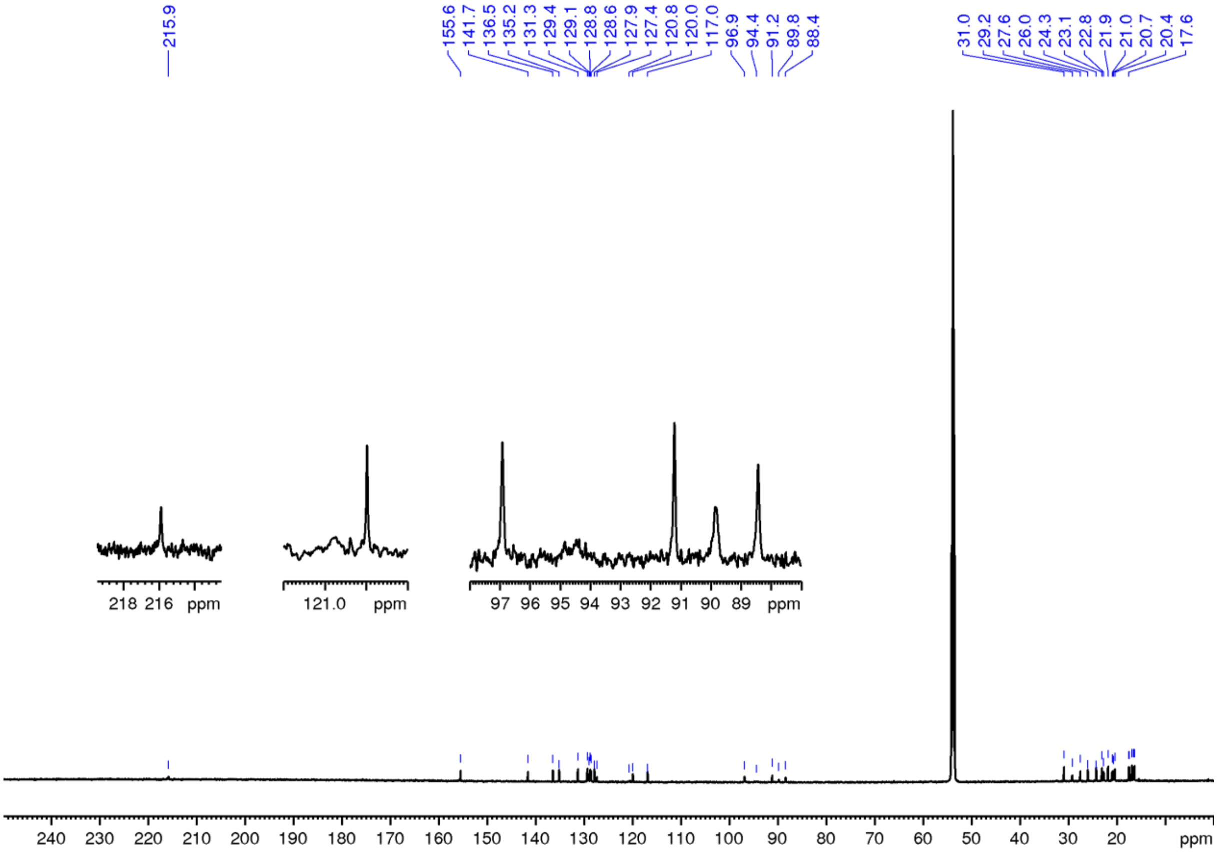

Figure S14: ^{13}C{^{1}H, ^{31}P} NMR spectrum of **[8]$^{+}$SbF$_6^{-}$** (CD_2Cl_2, 151 MHz, 22°C).

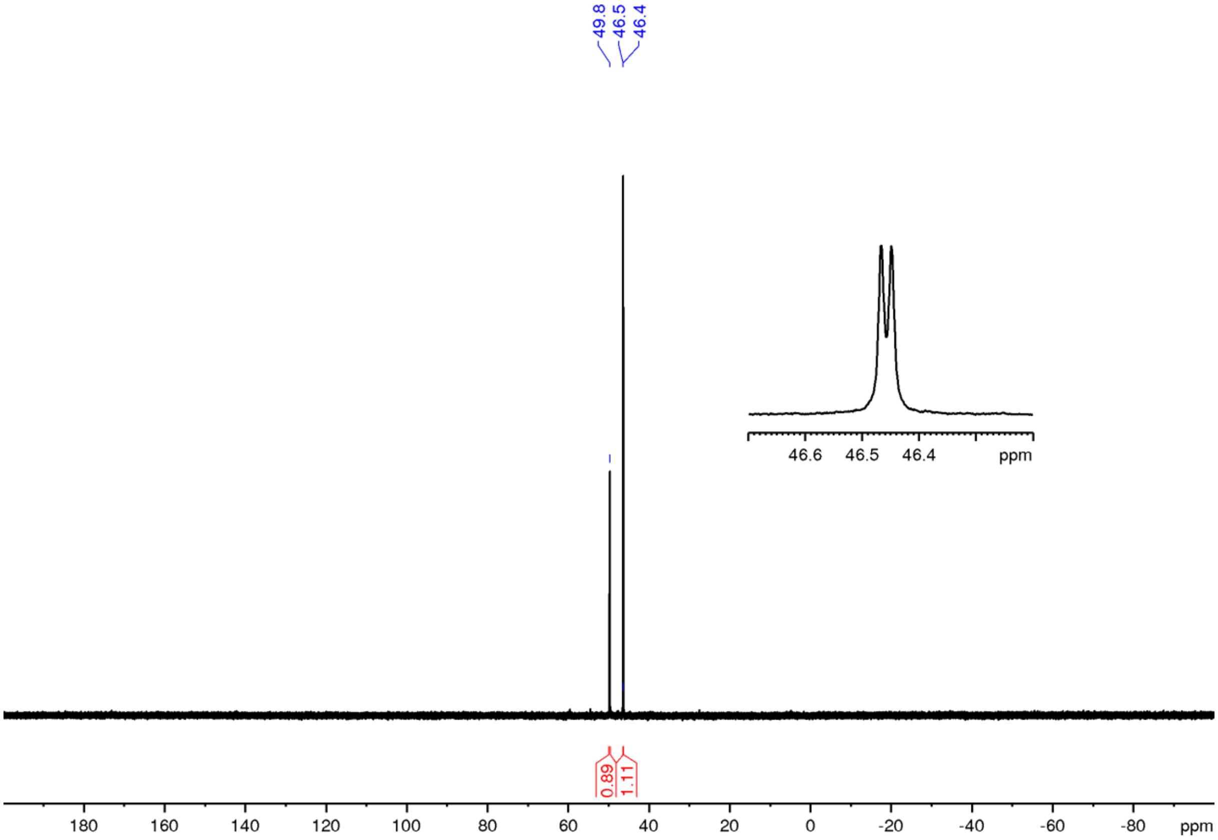

Figure S15: $^{31}P\{^1H\}$ NMR spectrum of **[8]$^+$SbF$_6^-$** (CD_2Cl_2, 243 MHz, 22°C).

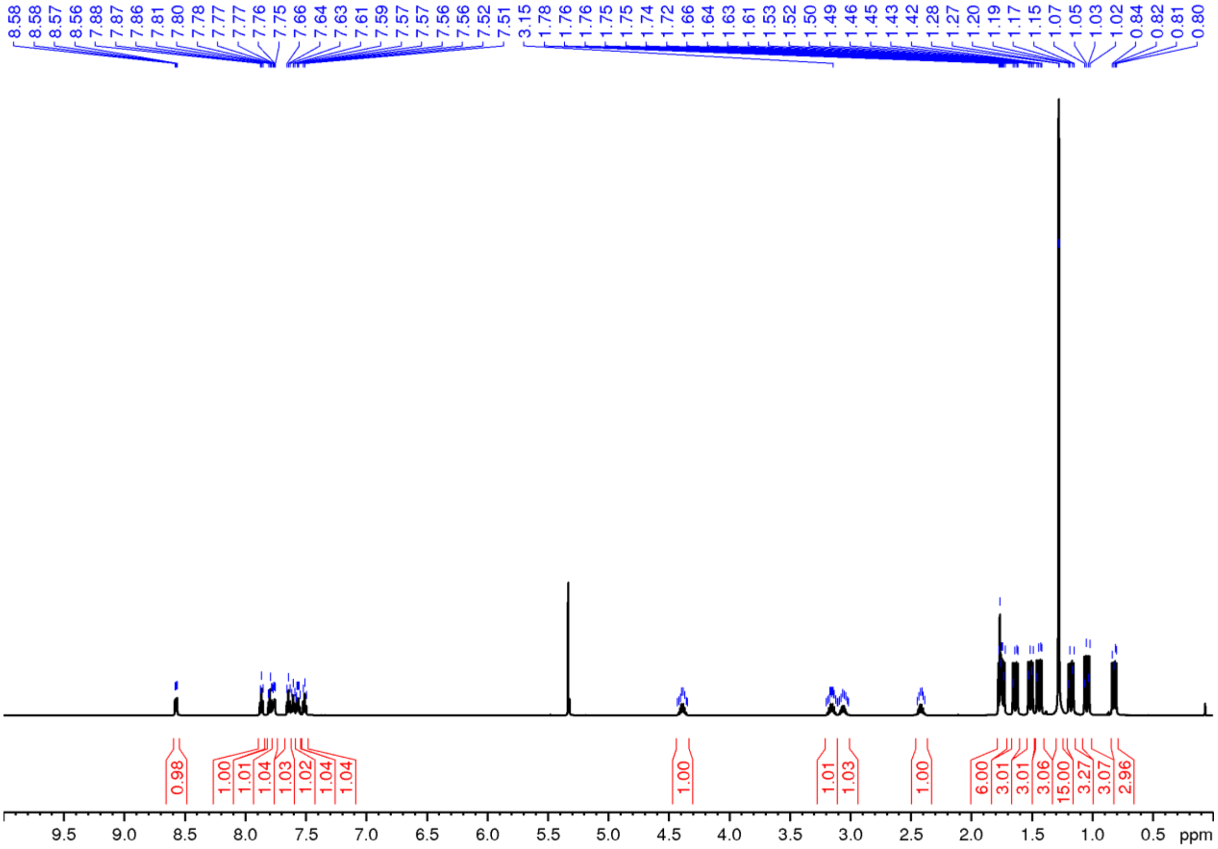

Figure S16: ^1H NMR spectrum of **[9]$^+$Cl$^-$** (CD_2Cl_2, 600 MHz, 22°C).

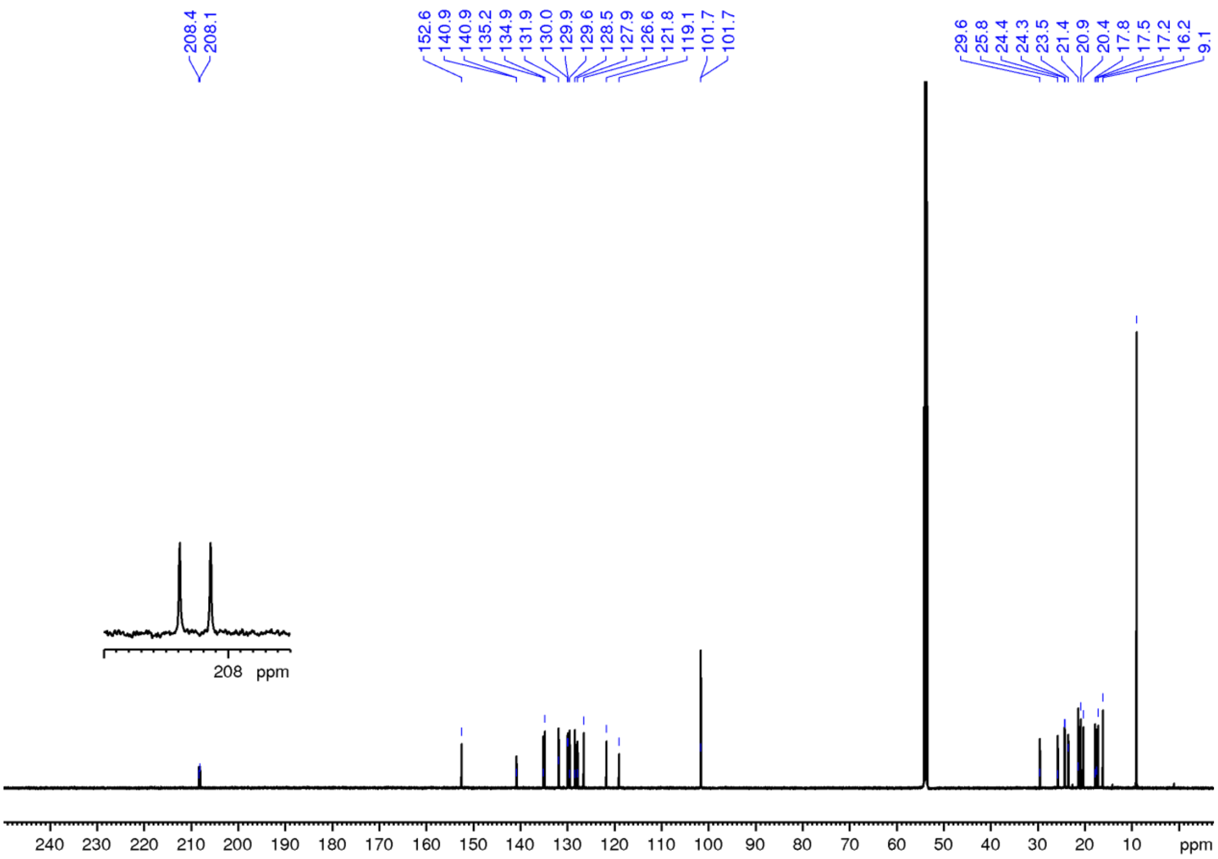

Figure S17: $^{13}C\{^{1}H, ^{31}P\}$ NMR spectrum of **[9]⁺Cl⁻** (CD_2Cl_2, 151 MHz, 22°C). Coupling to the ^{103}Rh (I = $^{1}/_{2}$) nucleus is observed, e.g. for the carbene signal.

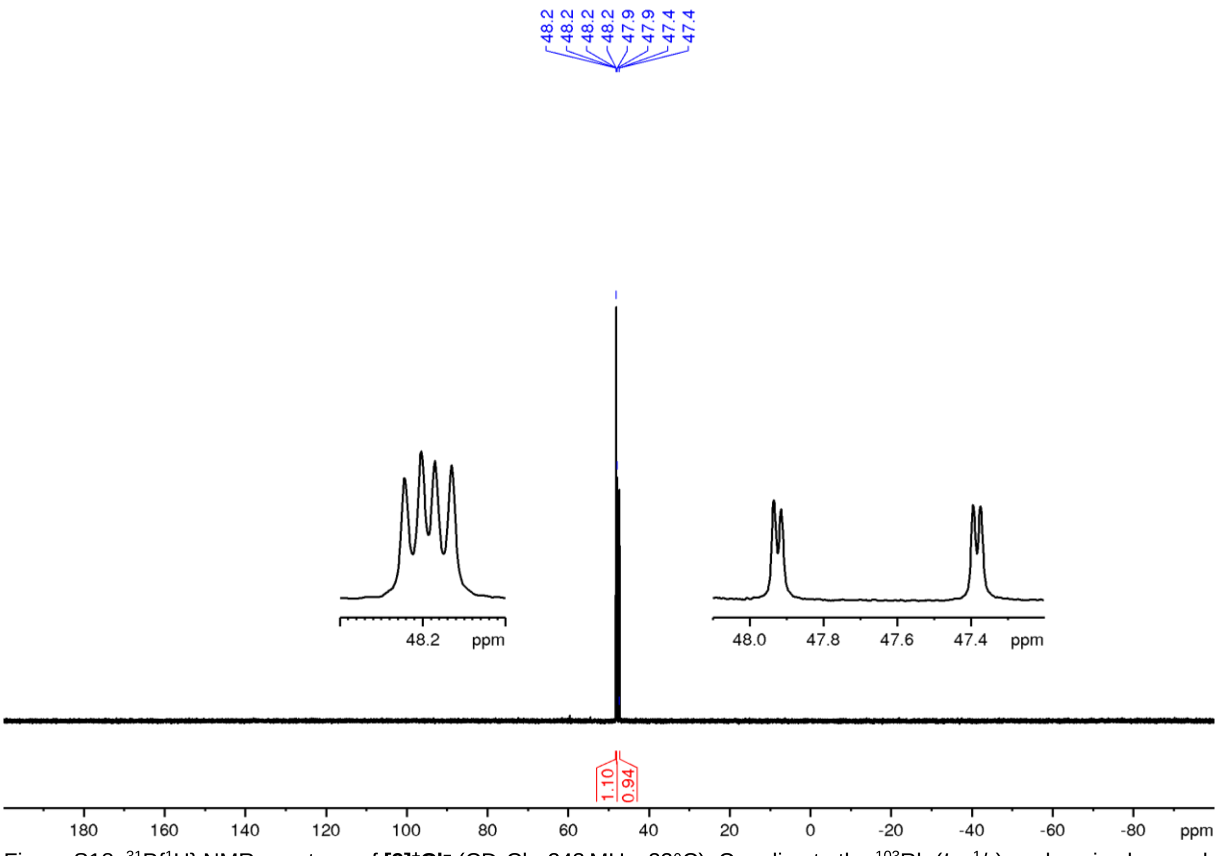

Figure S18: $^{31}P\{^{1}H\}$ NMR spectrum of **[9]⁺Cl⁻** (CD_2Cl_2, 243 MHz, 22°C). Coupling to the ^{103}Rh (I = $^{1}/_{2}$) nucleus is observed.

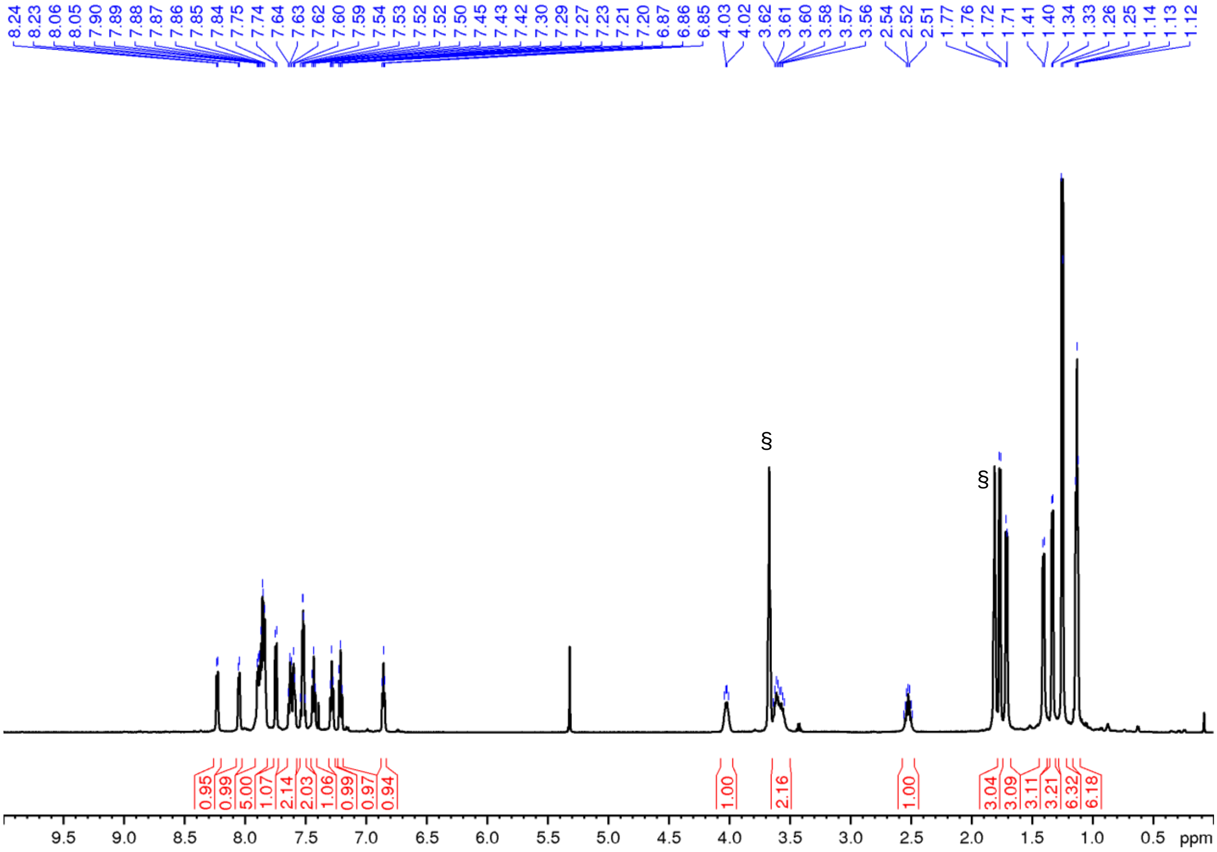

Figure S19: $^{1}H\{^{31}P\}$ NMR spectrum of **[10]⁺Cl⁻** (CD_2Cl_2, 600 MHz, 22°C). Co-crystallised thf is labelled with §.

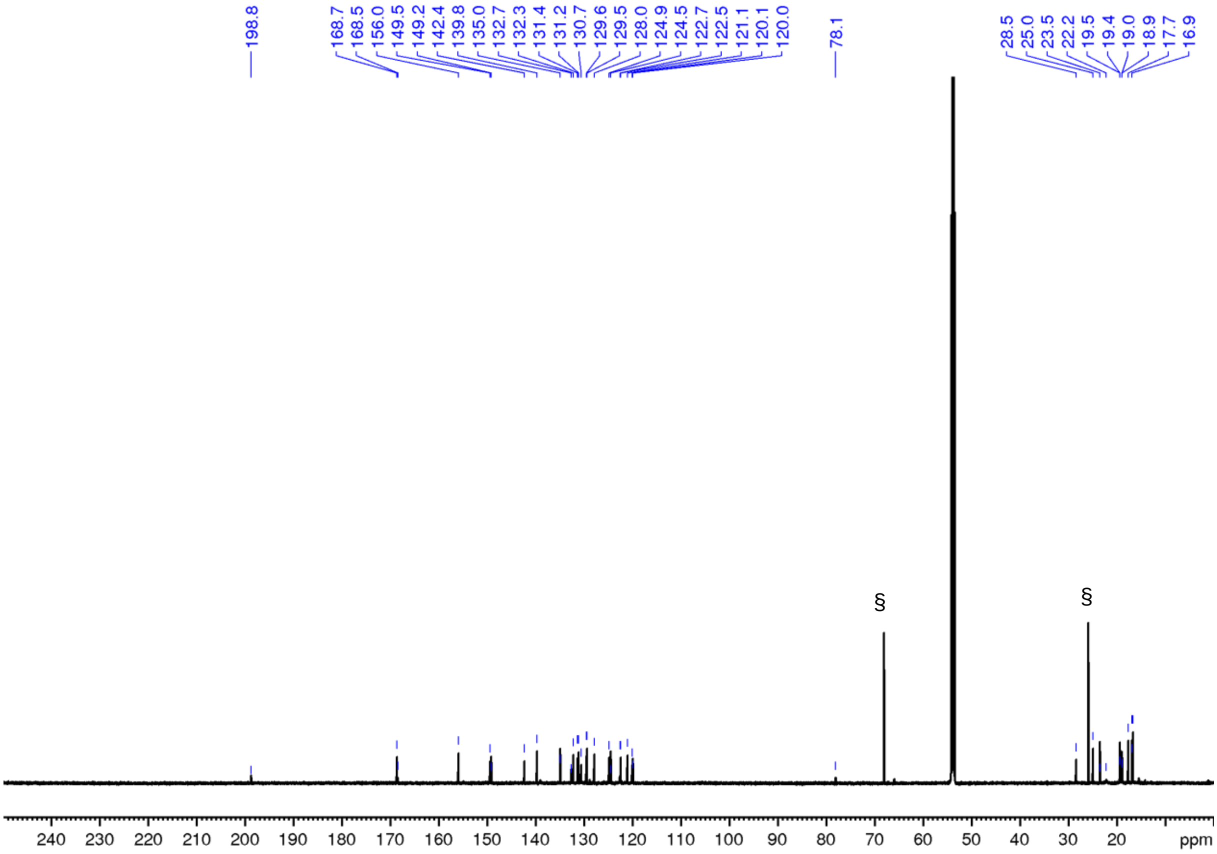

Figure S20: $^{13}C\{^{1}H, ^{31}P\}$ NMR spectrum of **[10]⁺Cl⁻** (CD_2Cl_2, 151 MHz, 22°C). Co-crystallised thf is labelled with §.

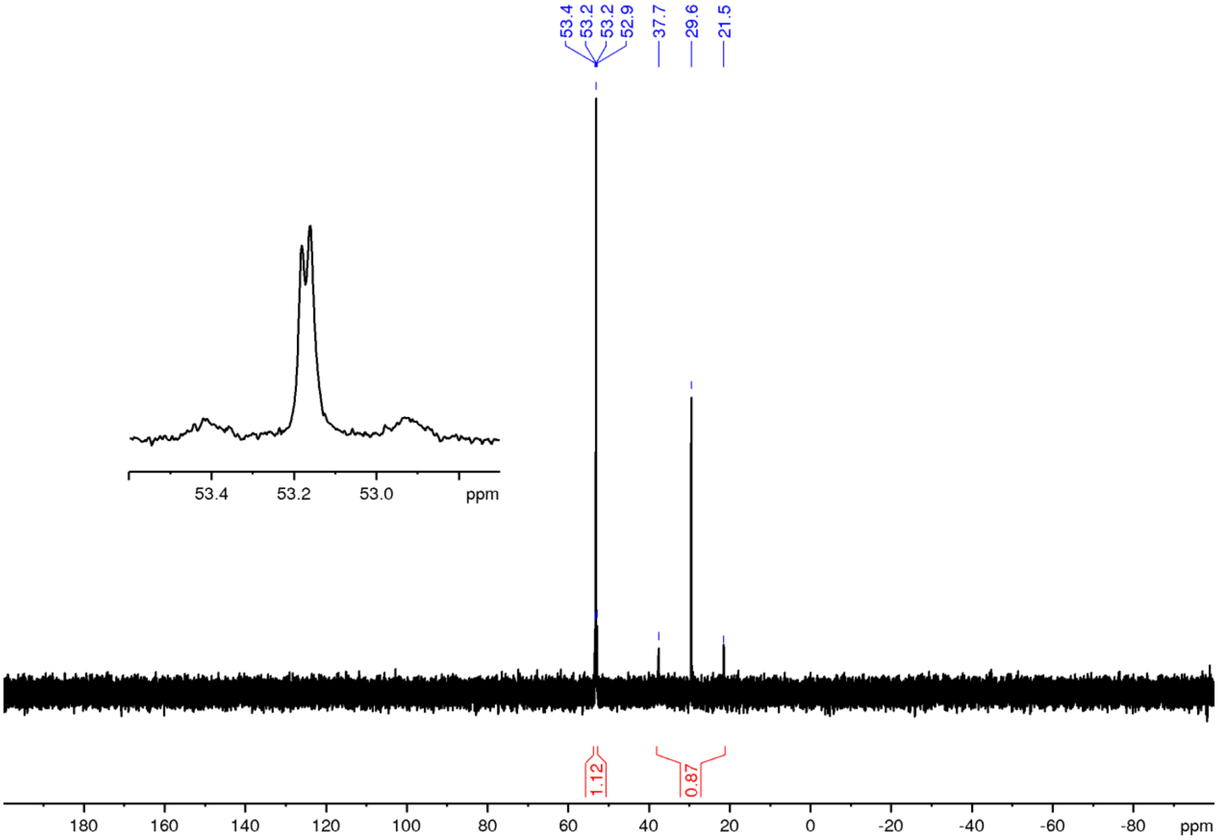

Figure S21: $^{31}P\{^{1}H\}$ NMR spectrum of **[10]⁺Cl⁻** (CD$_2$Cl$_2$, 242 MHz, 22°C). Coupling to the ^{195}Pt (I = $^{1}/_{2}$, natural abundance = 33.83%) is observed.

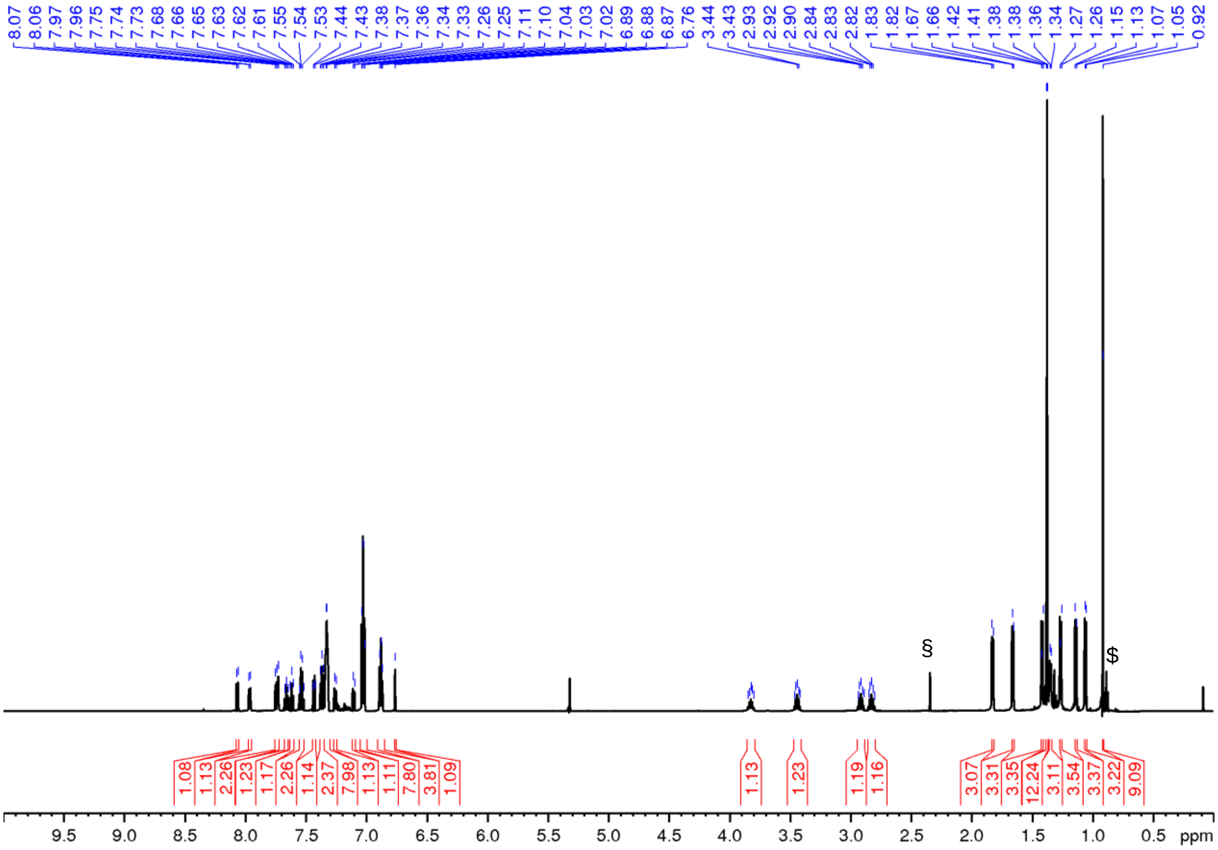

Figure S22: $^{1}H\{^{31}P\}$ NMR spectrum of **[11]⁺BPh₄⁻** (CD$_2$Cl$_2$, 600 MHz, 22°C). Residual solvent signals from washing are labelled with § (toluene) and $ (pentane), respectively.

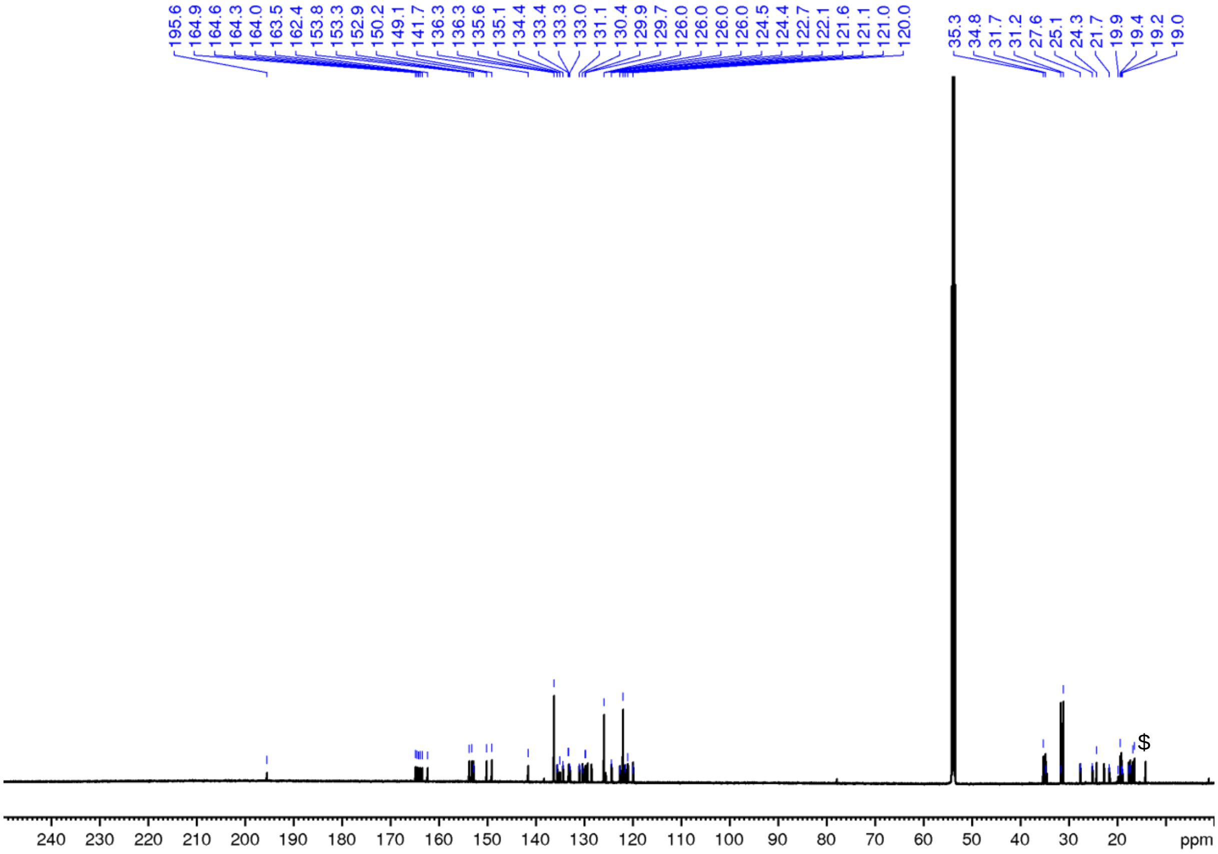

Figure S23: $^{13}C\{^1H, ^{31}P\}$ NMR spectrum of **[11]$^+$BPh$_4^-$** (CD_2Cl_2, 151 MHz, 22°C). Residual solvent signals from washing are labelled with $ (pentane), respectively.

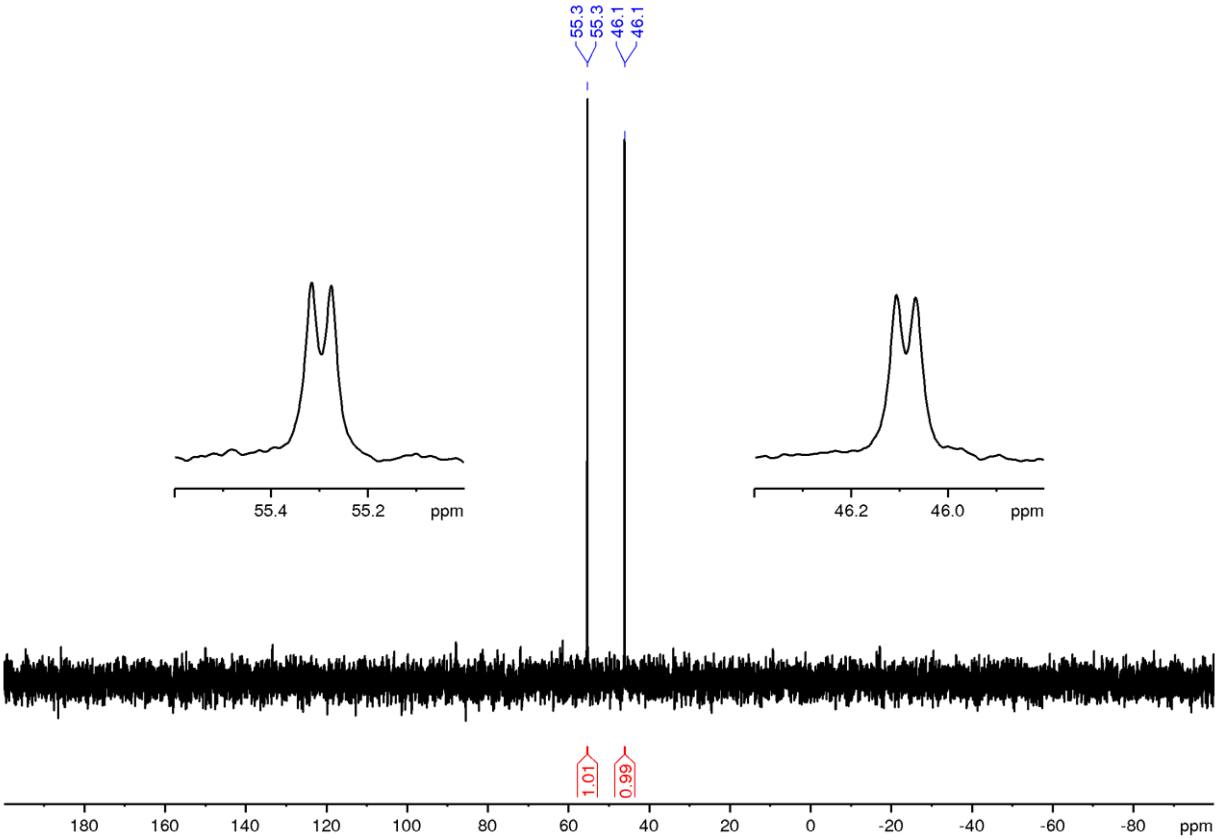

Figure S24: $^{31}P\{^1H\}$ NMR spectrum of **[11]$^+$BPh$_4^-$** (CD_2Cl_2, 243 MHz, 22°C).

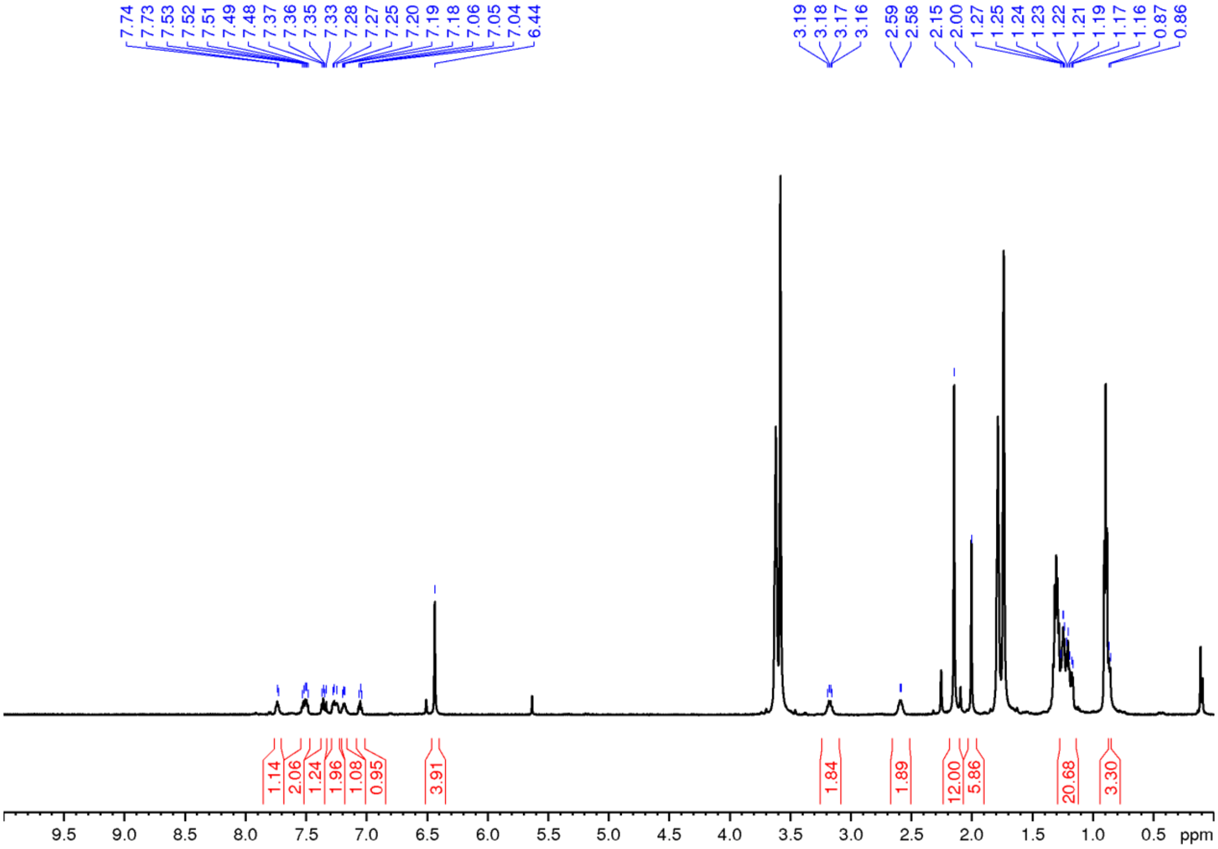

Figure S25: ^1H NMR spectrum of **12** (thf-d_8, 600 MHz, –40°C).

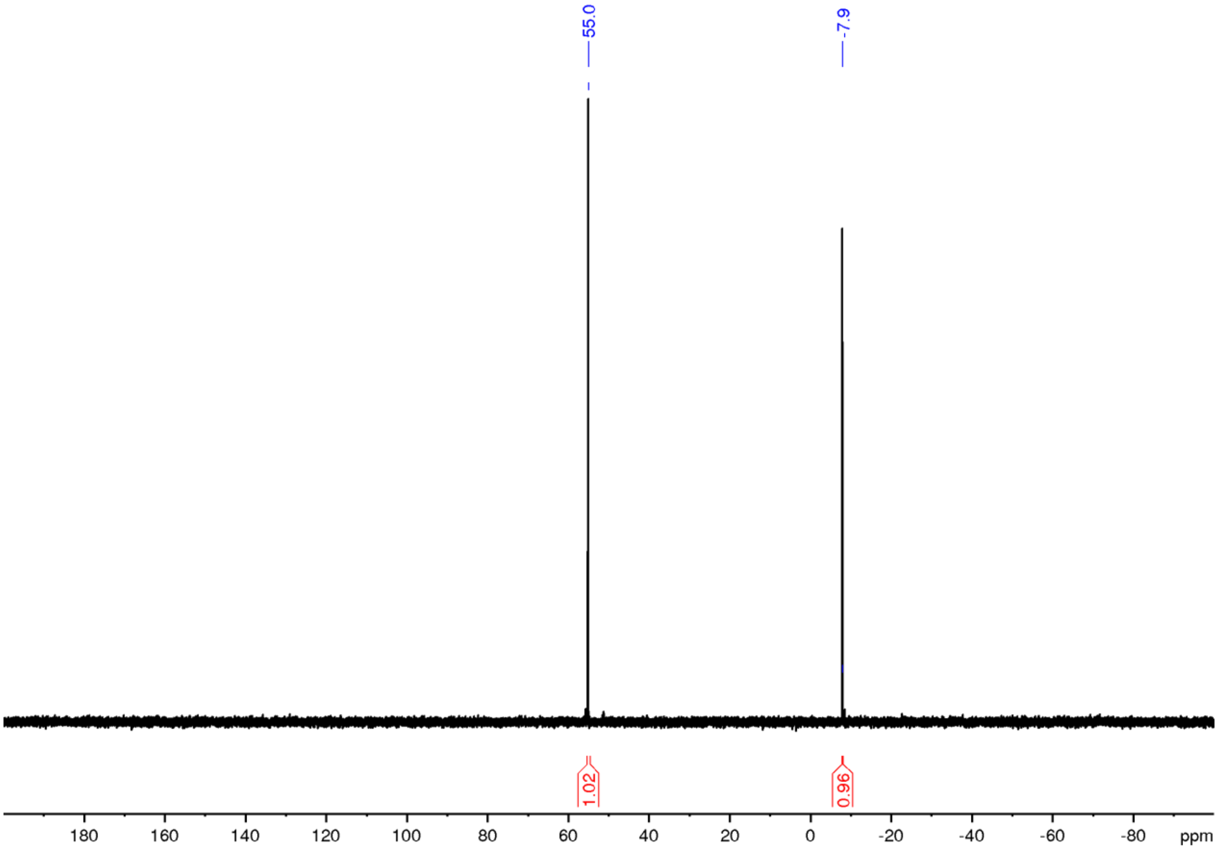

Figure S26: $^{31}P\{^1H\}$ NMR spectrum of **12** ($C_6D_5NO_2$, 162 MHz, 22°C).

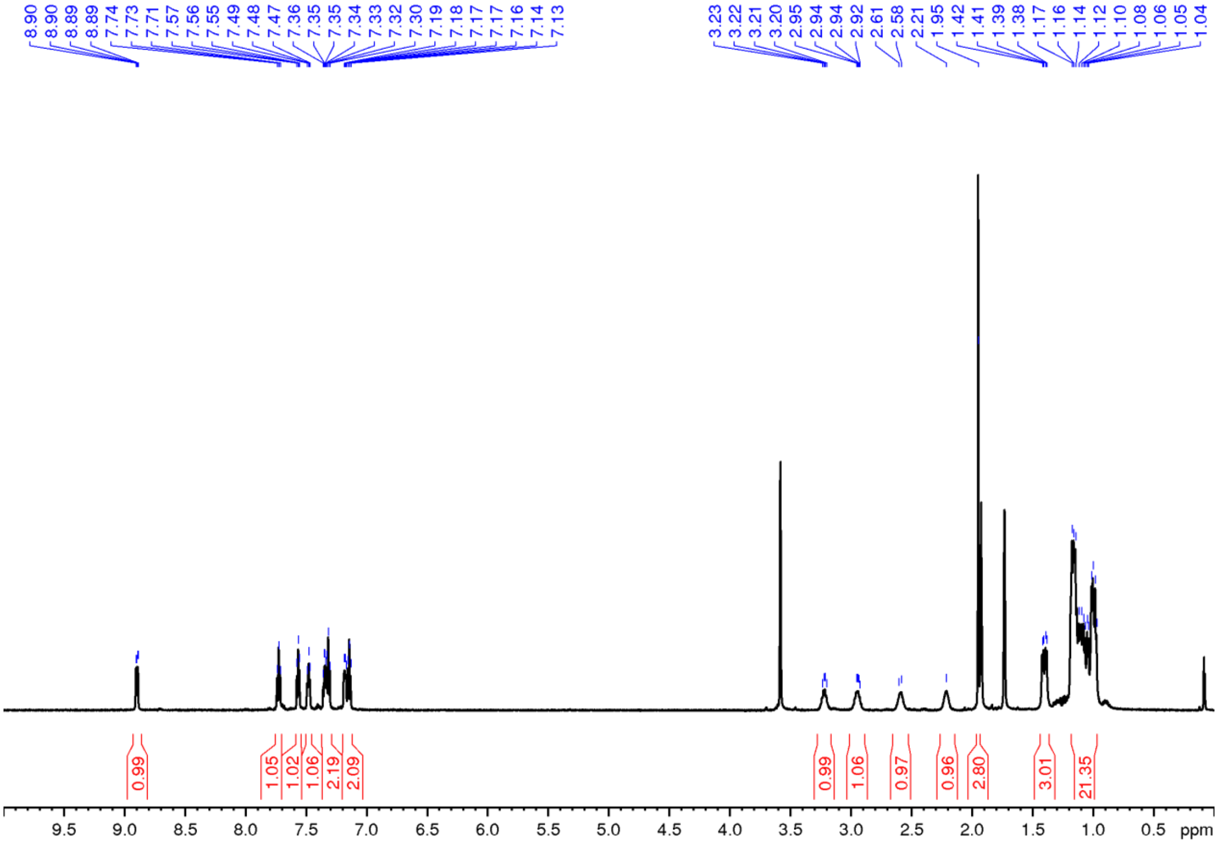

Figure S27: $^{1}H\{^{31}P\}$ NMR spectrum of **13** (thf-d_8/CD_3CN 4:1, 600 MHz, 22°C).

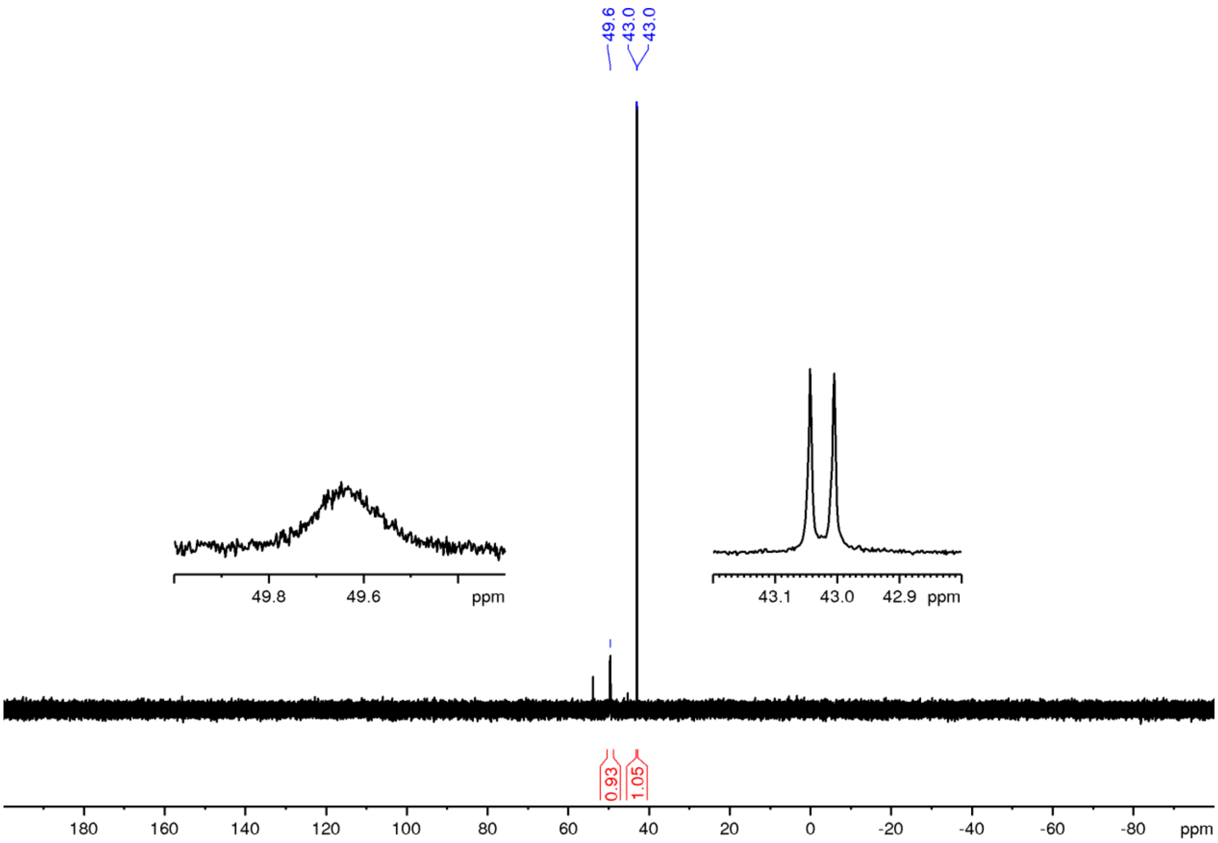

Figure S28: $^{31}P\{^{1}H\}$ NMR spectrum of **13** (thf-d_8/CD_3CN 4:1, 243 MHz, 22°C). The use of thf-d_8 was required due to the low solubility of this compound in the pure CD_3CN. Upon addition of thf-d_8, however, the conversion of **13** to **14** also set in, which led to the singlet at 54.3 ppm (not integrated).

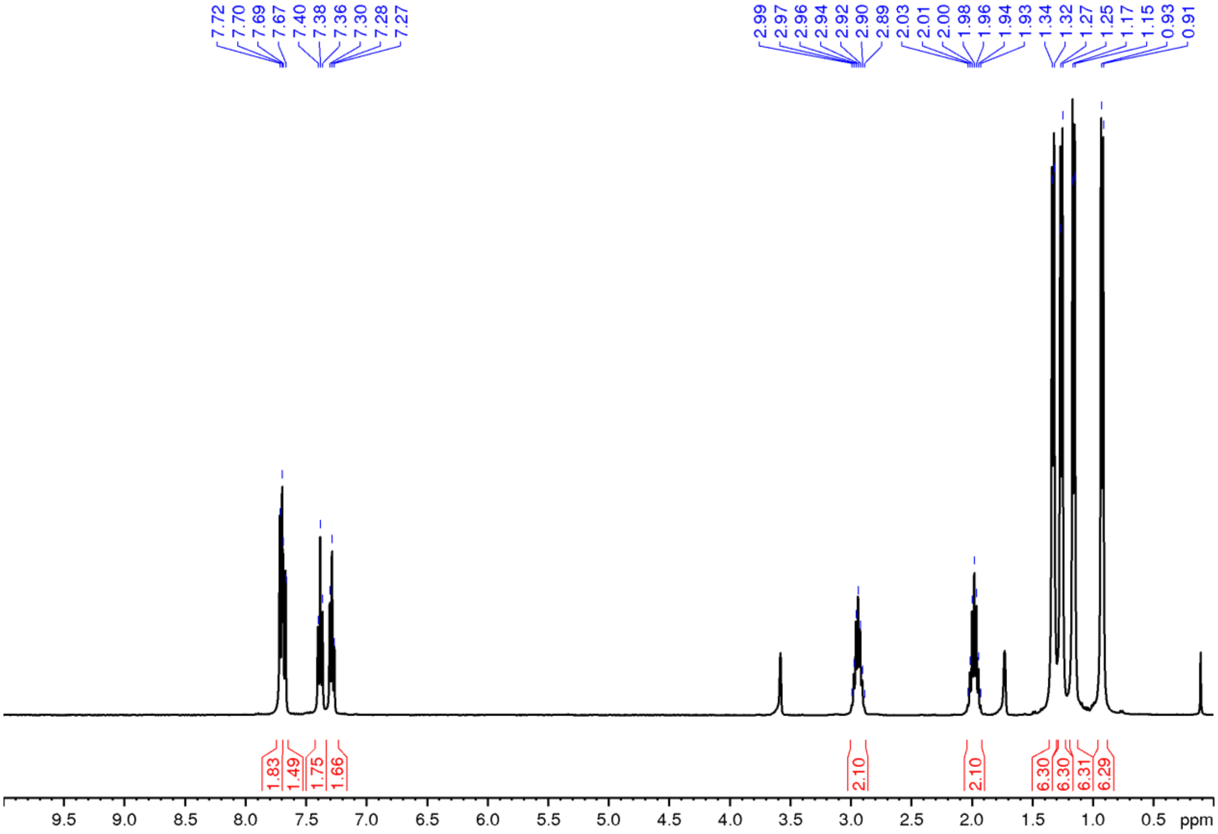

Figure S29: $^{1}H\{^{31}P\}$ NMR spectrum of **14** (thf-d_8, 400 MHz, 22°C).

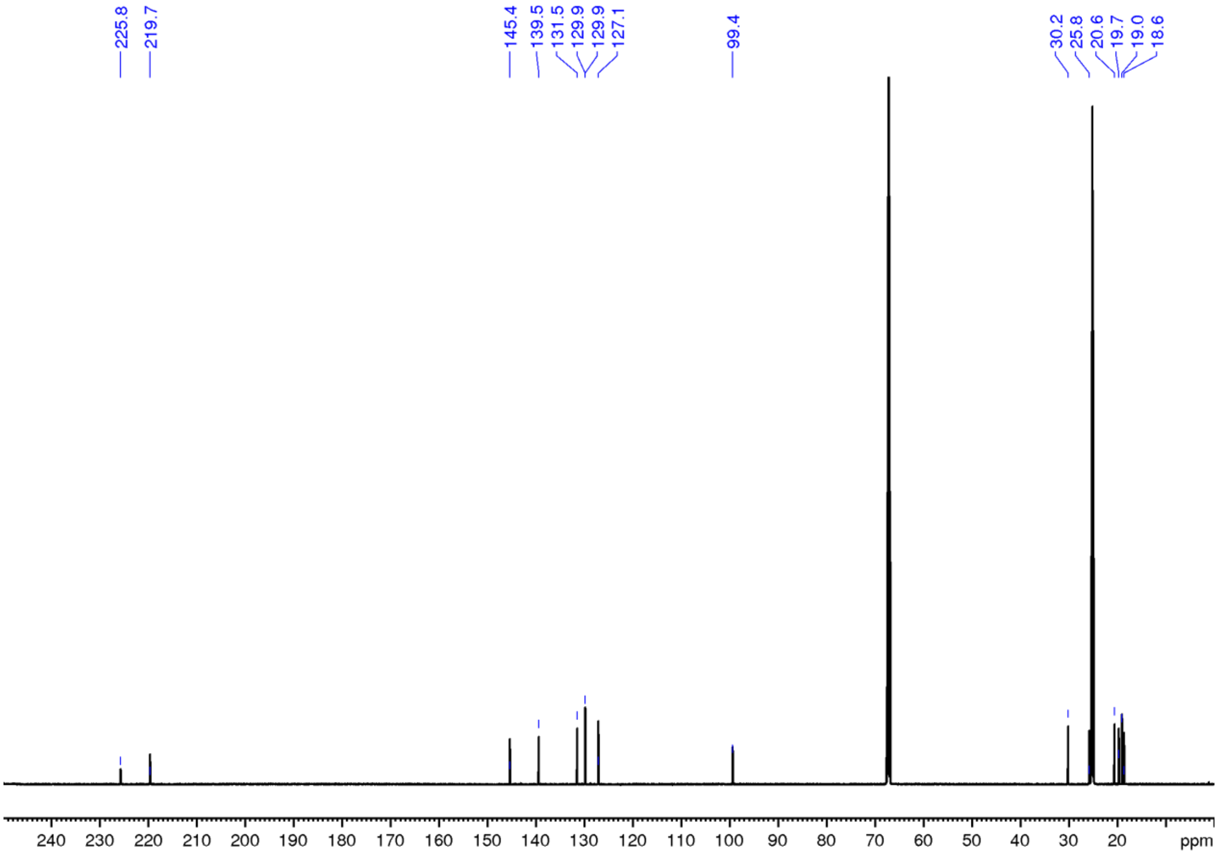

Figure S30: $^{13}C\{^{1}H, ^{31}P\}$ NMR spectrum of **14** (thf-d_8, 151 MHz, 22°C).

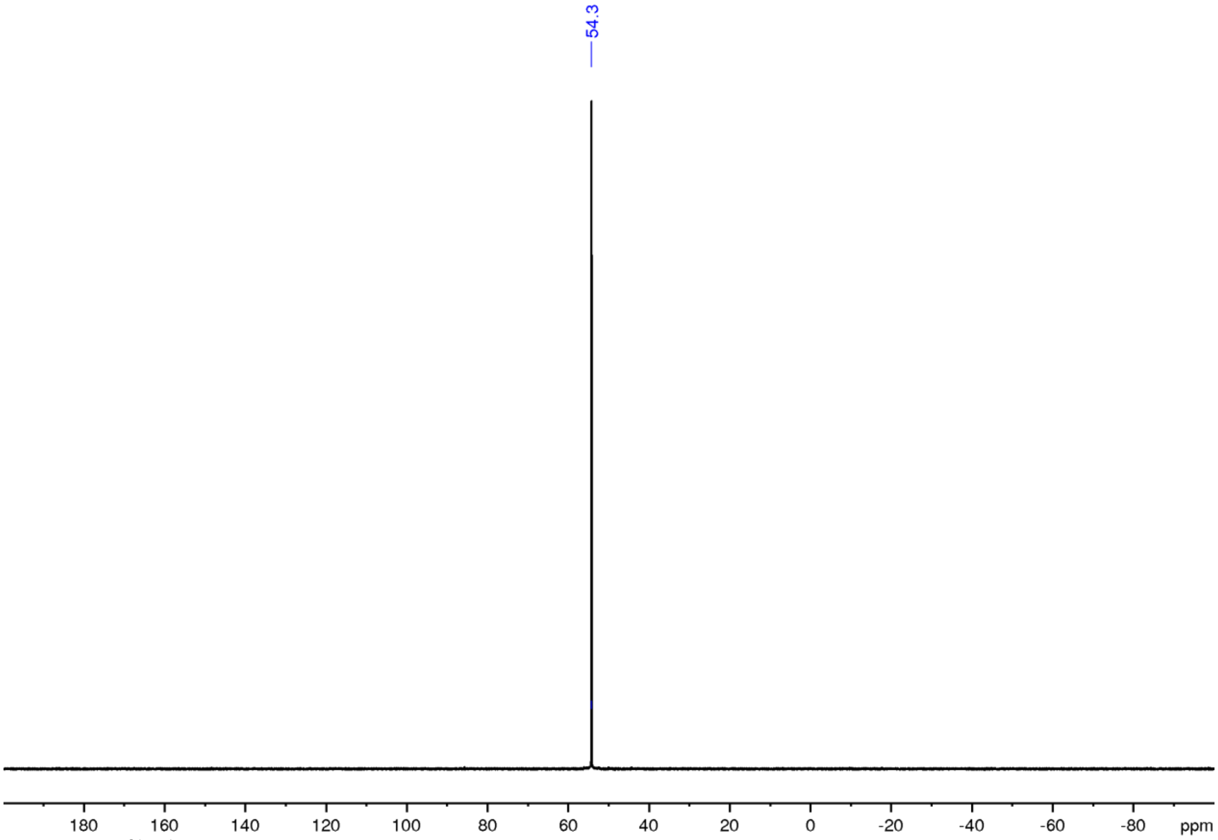

Figure S31: $^{31}P\{^{1}H\}$ NMR spectrum of **14** (thf-d_8, 162 MHz, 22°C).

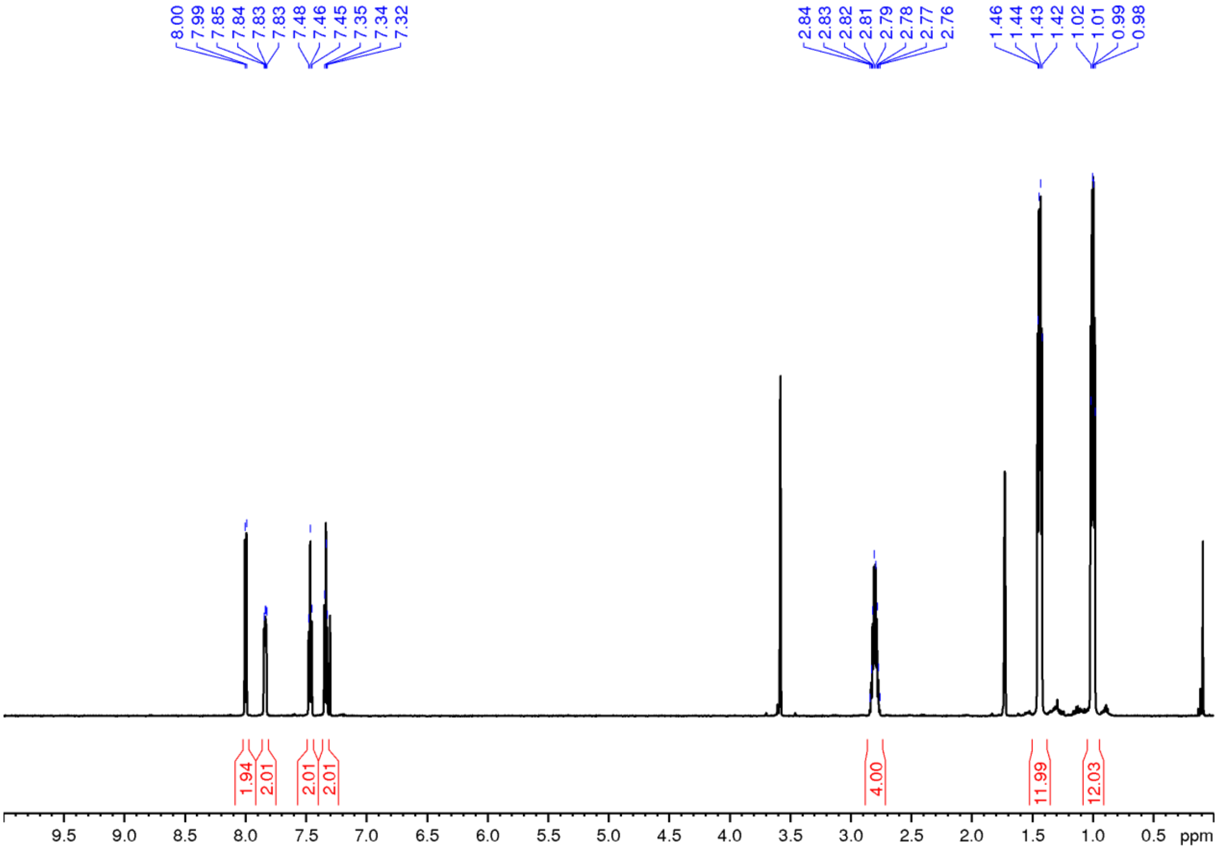

Figure S32: ^{1}H NMR spectrum of **15** (thf-d_8, 600 MHz, 22°C).

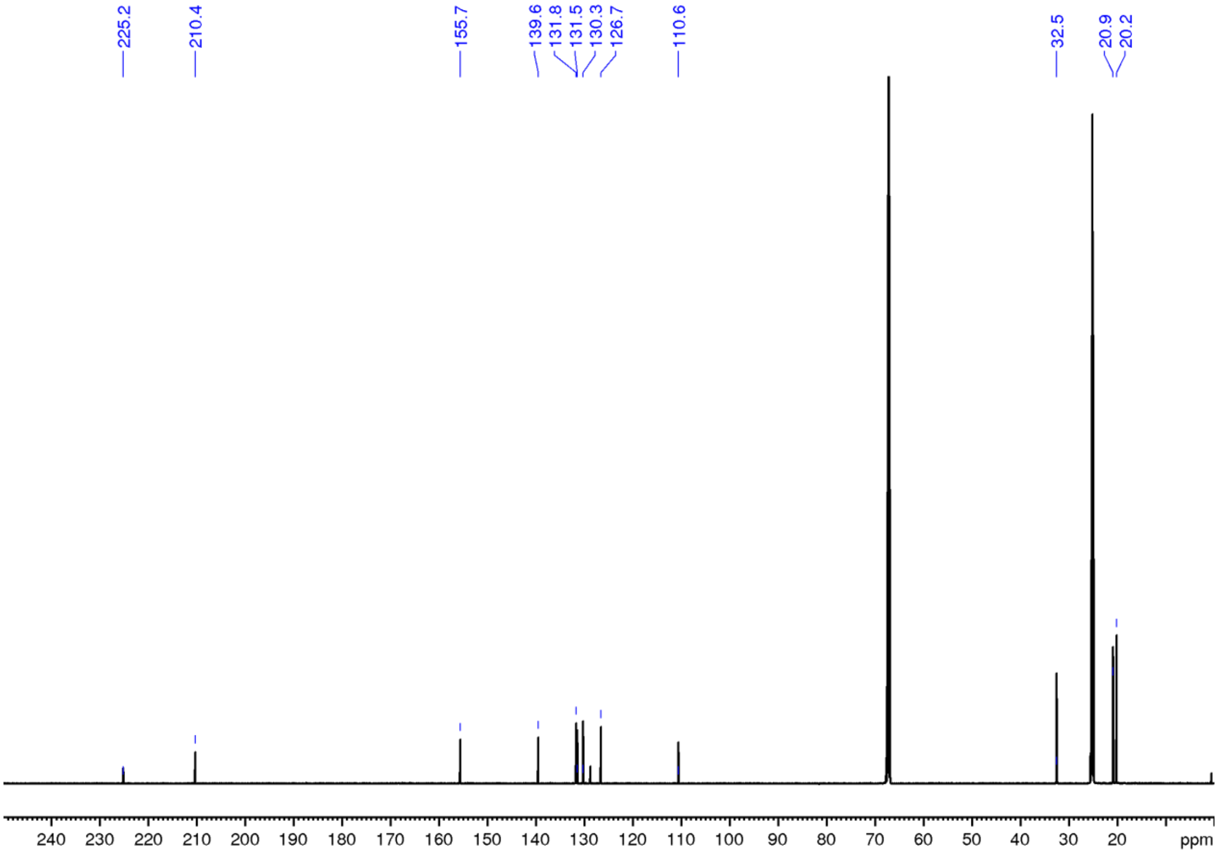

Figure S33: $^{13}C\{^{1}H, ^{31}P\}$ NMR spectrum of **15** (thf-d_8, 151 MHz, 22°C).

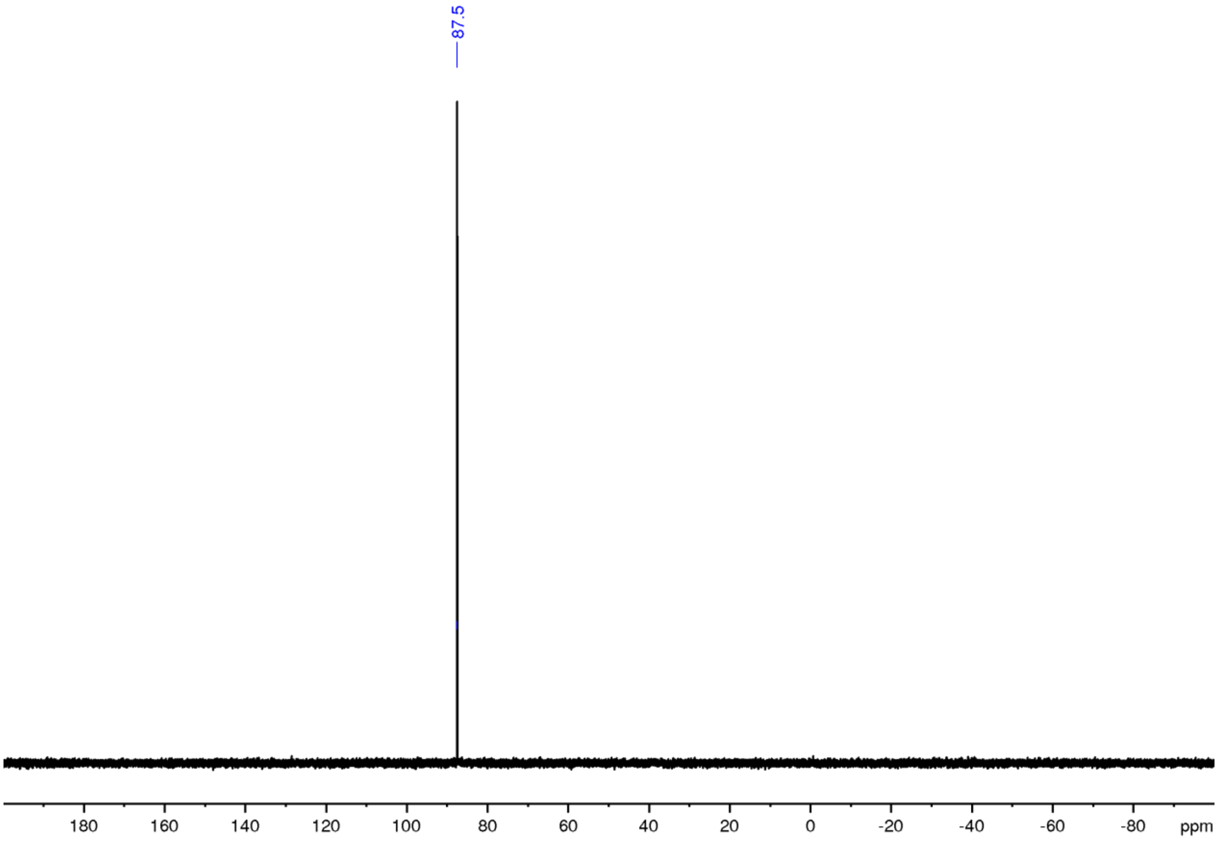

Figure S34: $^{31}P\{^{1}H\}$ NMR spectrum of **15** (C_6D_6, 162 MHz, 22°C).

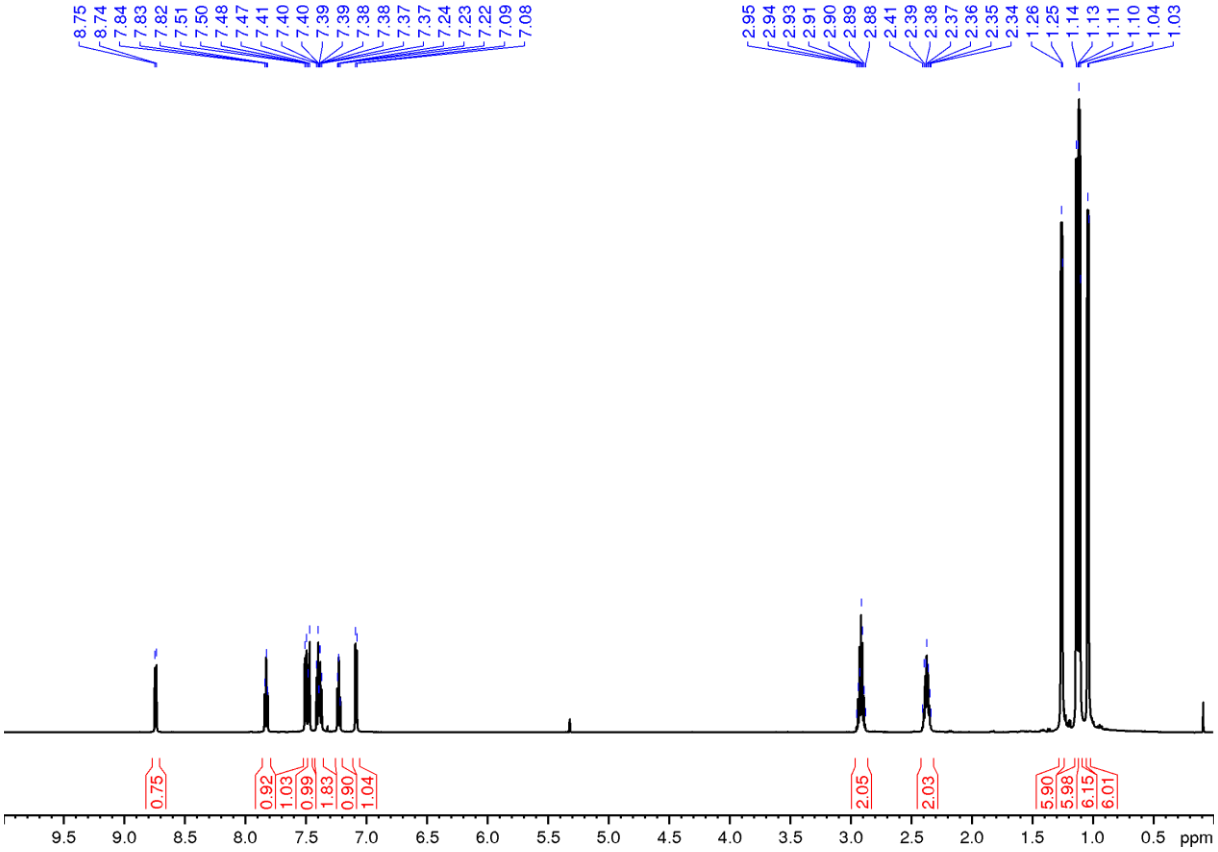

Figure S35: $^{1}H\{^{31}P\}$ NMR spectrum of **16** (CD_2Cl_2, 600 MHz, 22°C).

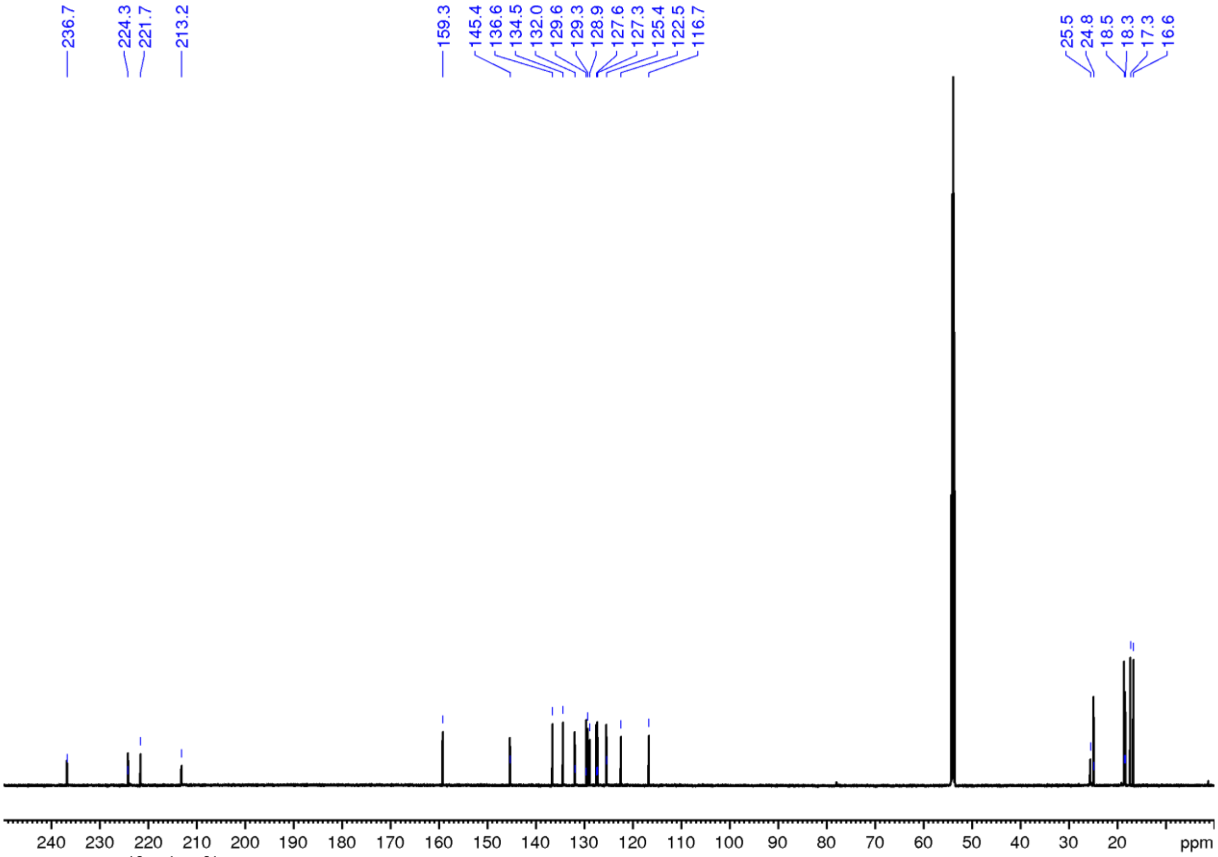

Figure S36: $^{13}C\{^{1}H, ^{31}P\}$ NMR spectrum of **16** (CD_2Cl_2, 151 MHz, 22°C).

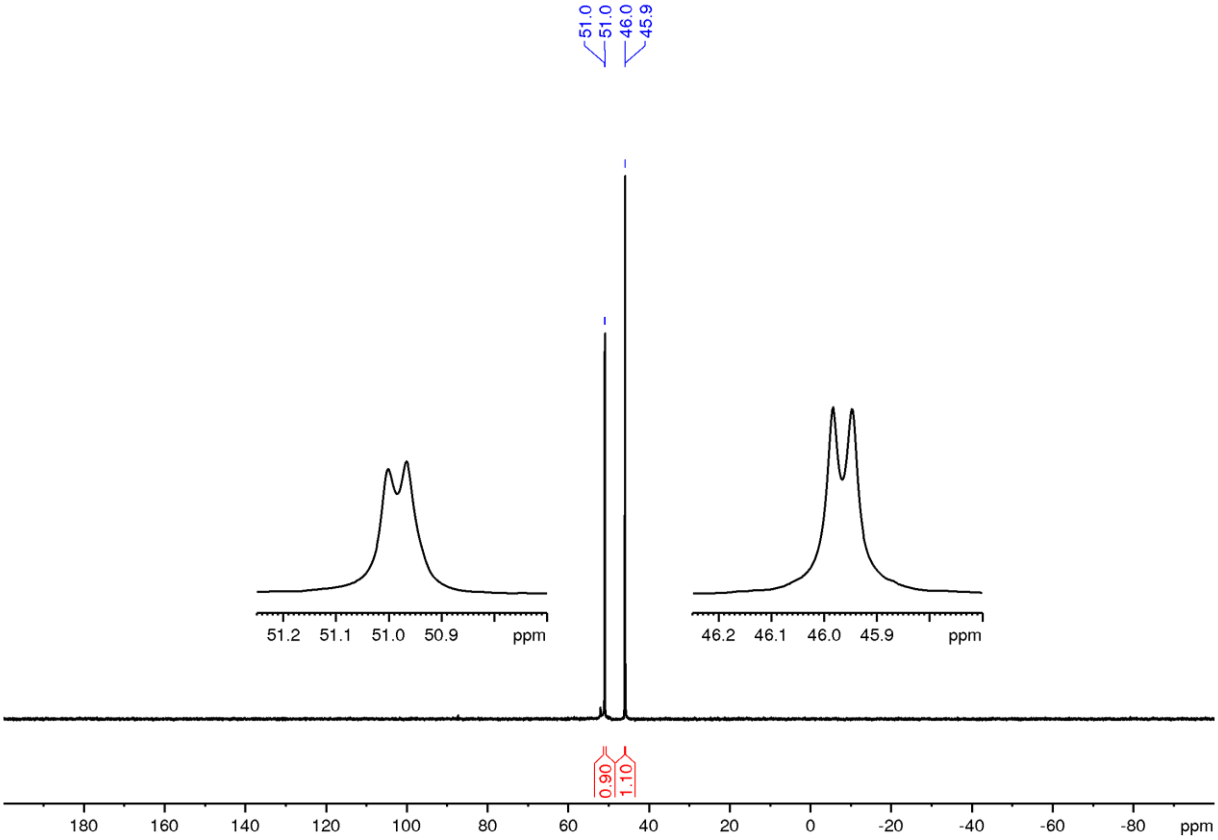

Figure S37: $^{31}P\{^{1}H\}$ NMR spectrum of **16** (CD_2Cl_2, 242 MHz, 22°C).

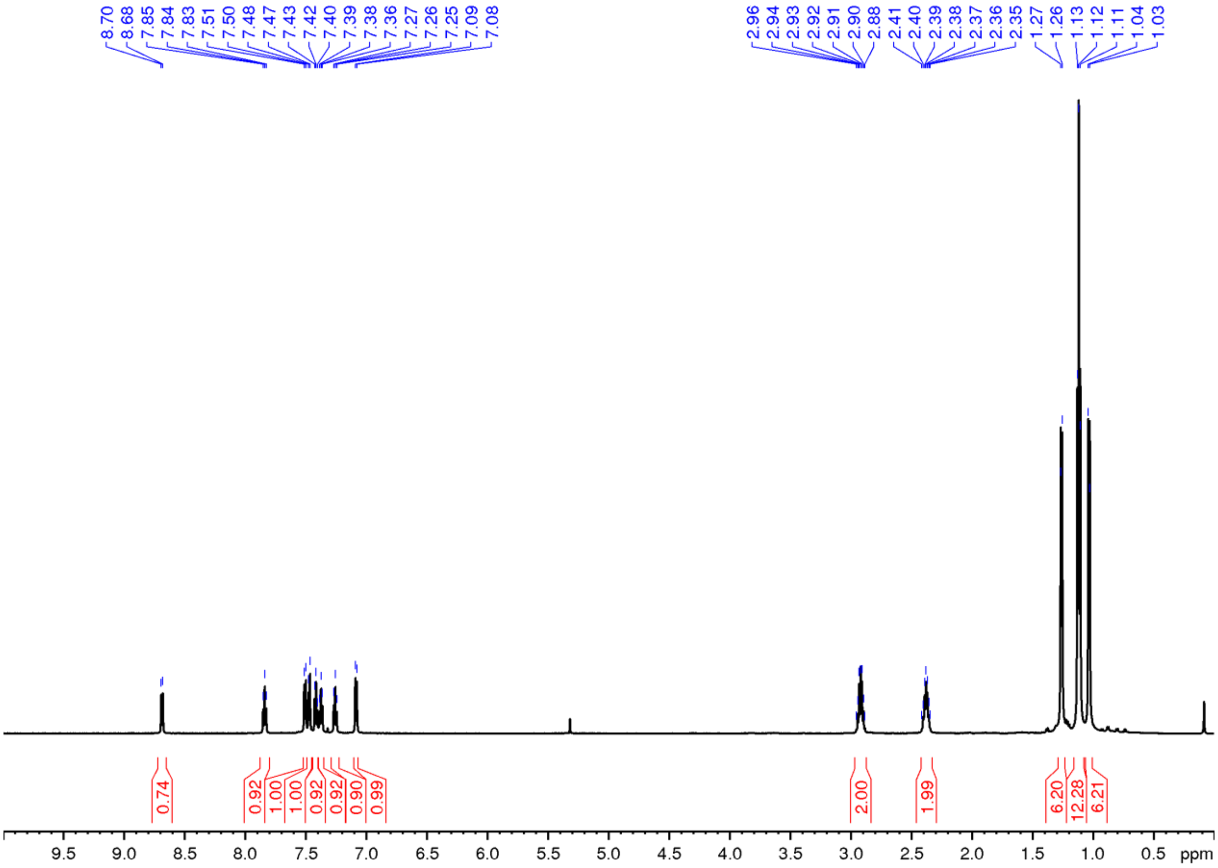

Figure S38: $^{1}H\{^{31}P\}$ NMR spectrum of **16-W** (CD_2Cl_2, 600 MHz, 22°C).

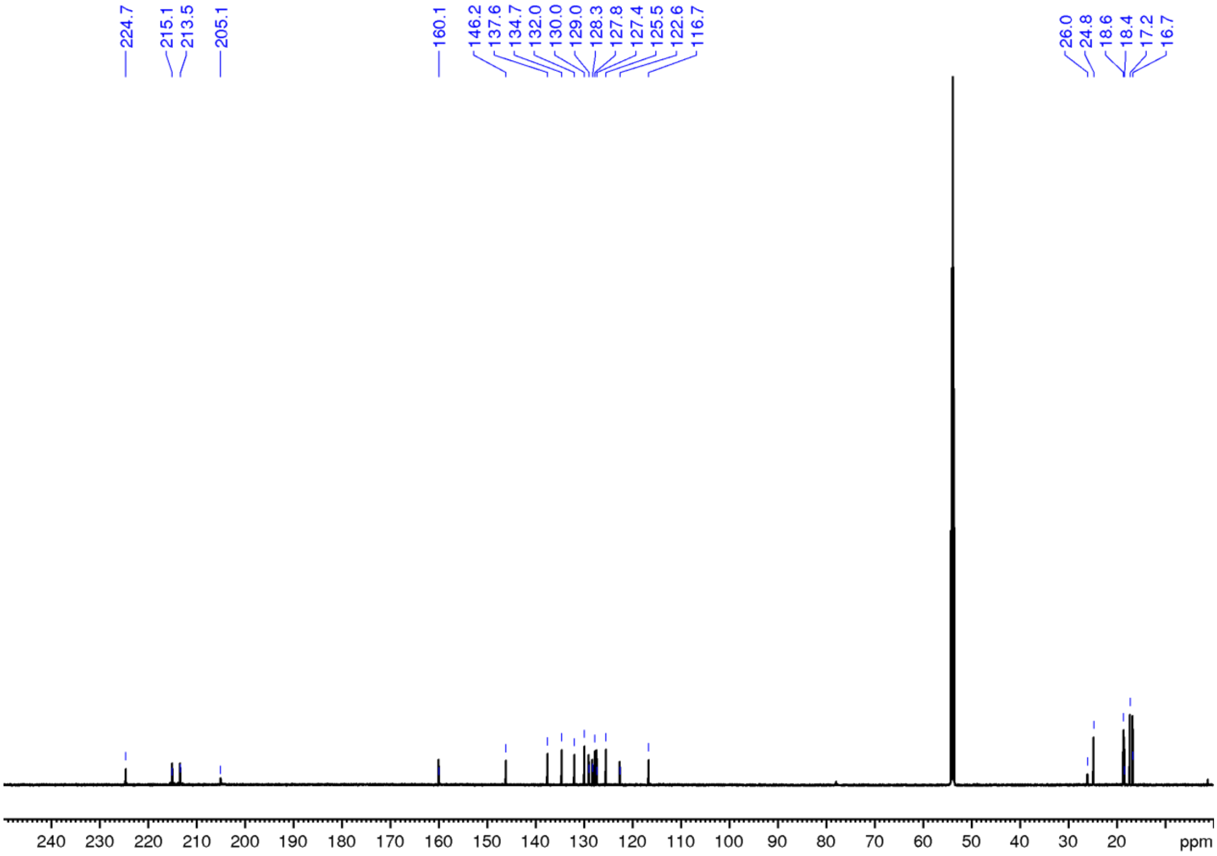

Figure S39: $^{13}C\{^1H, ^{31}P\}$ NMR spectrum of **16-W** (CD_2Cl_2, 151 MHz, 22°C).

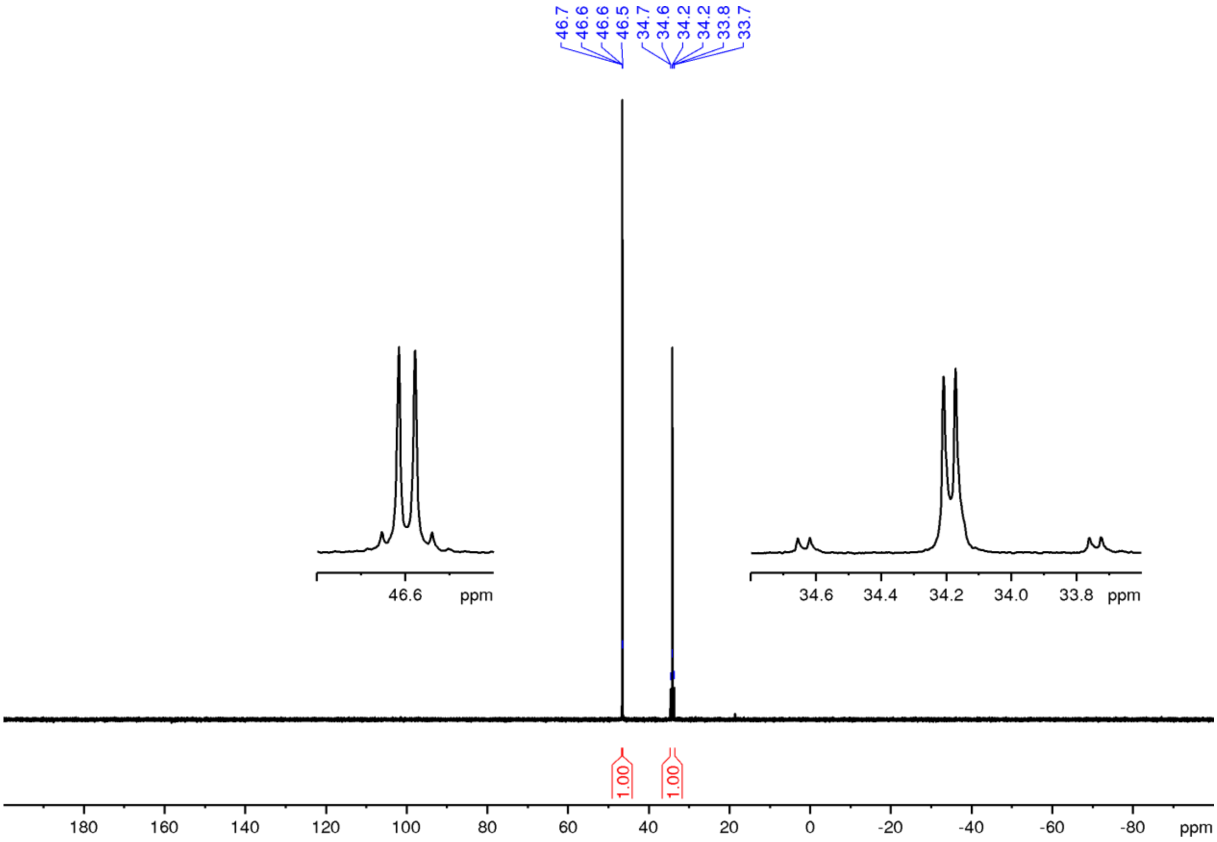

Figure S40: $^{31}P\{^1H\}$ NMR spectrum of **16-W** (CD_2Cl_2, 243 MHz, 22°C).

Details on DFT Calculations

Computational Results

DFT calculations for wave function and IBO analysis were carried out with Gaussian 16 (G16RevB.01).[13] All structures were optimised without symmetry restrictions and identified as minima or transition states by analytical frequency analyses. The complexes were modeled using the PBE0 (PBE1PBE) functional[14] on the Def2-TZVP basis set.[15] For modelling the mechansim for the interconversion between **13** and **14**, dispersion corrections (GD3)[16] were taken into account. Energies were corrected for solvent effects using the polarizable continuum model (PCM for thf or MeCN)[17,18] as implemented in Gaussian.

Topological wave function analysis was carried out on the optimised structures (PBE1PBE/Def2-TZVP) using MultiWFN (version 3.7, release 2020-April-10).[19] The calculated Intrinsic bond orbitals were visualised using IBOview (v20150427).[20] Energy decomposition analysis (EDA) and NOCV analysis (natural orbitals for chemical valence)[21] were carried out on the BP86 functional[22,23] using ADF 2017.[24,25] In this case, the geometries were optimised on the BP86/TZVP-D3(BJ) level using effective core potentials. Scalar relativistic effects (ZORA)[26] were included as implemented in ADF. The complexes were fragmented into a ligand part (singlet) and a metal-containing part (singlet, positively charged in the case of **[3]**$^+$, **[8]**$^+$, **[9]**$^+$, **[10]**$^+$ and **[11]**$^+$). Deformation densities were calculated with ADF 2017 and plotted using ChemCraft (v1.8).[27] The EDA results are summarised in Table S1.

Table S1: Summary of the EDA results. The interaction energy (ΔE_{int}) is decomposed according to $\Delta E_{int} = \Delta E_{elstat} + \Delta E_{pauli} + \Delta E_{orb} + \Delta E_{disp}$ with ΔE_{elstat} = electrostatic interaction, ΔE_{pauli} = Pauli repulsion, ΔE_{orb} = orbital interaction and ΔE_{disp} =dispersion interaction.

compound	ΔE_{int}	ΔE_{elstat}	ΔE_{pauli}	ΔE_{orb}	ΔE_{disp}
**[3]**$^+$	–92.58	–102.19	107.04	–63.69	–33.73
**4**	–107.74	–146.19	165.48	–99.82	–27.21
**5**	–111.54	–165.83	196.97	–110.01	–32.67
**6**	–114.23	–147.38	165.83	–110.21	–22.46
**7**	–122.68	–181.77	217.86	–133.56	–25.21
**[8]**$^+$	–190.21	–279.72	342.33	–221.25	–31.57
**[9]**$^+$	–182.07	–284.49	345.39	–207.44	–35.53
**[10]**$^+$	–199.10	–324.14	358.11	–206.70	–26.37
**[11]**$^+$	–167.47	–265.34	303.59	–175.13	–30.58
**12**	–101.60	–174.00	224.34	–105.42	–46.53

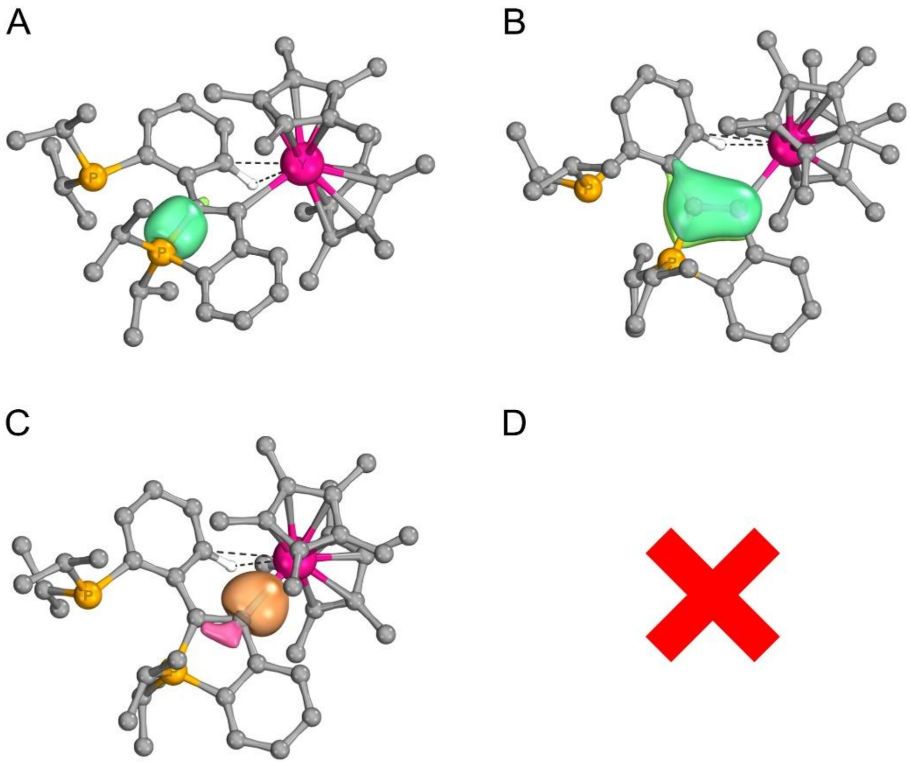

Figure S41: Selected IBO plots for complex **[3]⁺** (A: *σ*-P-C bond, B: *π*-C=C-bond, C: dative *σ*-C-Y bond, D: no *π*-Y-C backbonding IBO was found). Threshold value for printing: 75.0 for A, B and C.

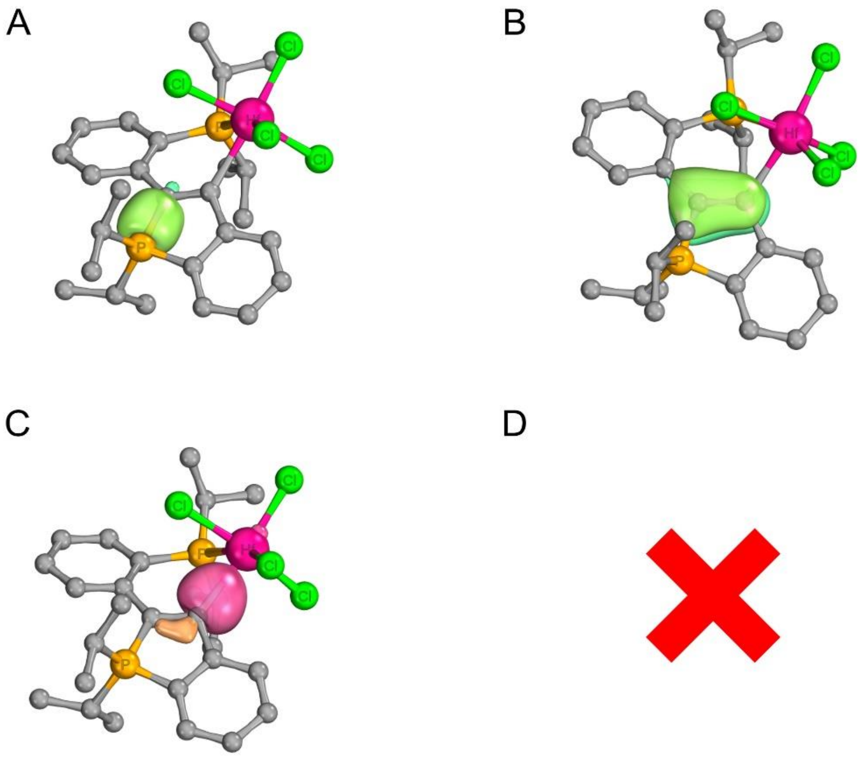

Figure S42: Selected IBO plots for complex **4** (A: *σ*-P-C bond, B: *π*-C=C-bond, C: dative *σ*-C-Hf bond, D: no *π*-Hf-C backbonding IBO was found). Threshold value for printing: 75.0 for A, B and C.

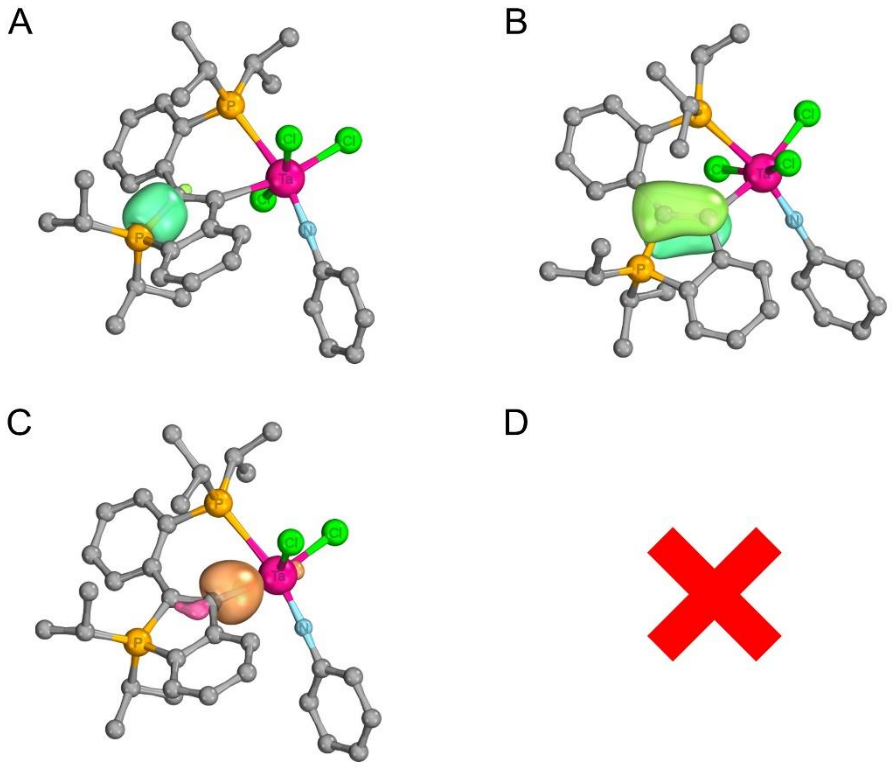

Figure S43: Selected IBO plots for complex **5** (A: *σ*-P-C bond, B: *π*-C=C-bond, C: dative *σ*-C-Ta bond, D: no *π*-Ta-C backbonding IBO was found). Threshold value for printing: 75.0 for A, B and C.

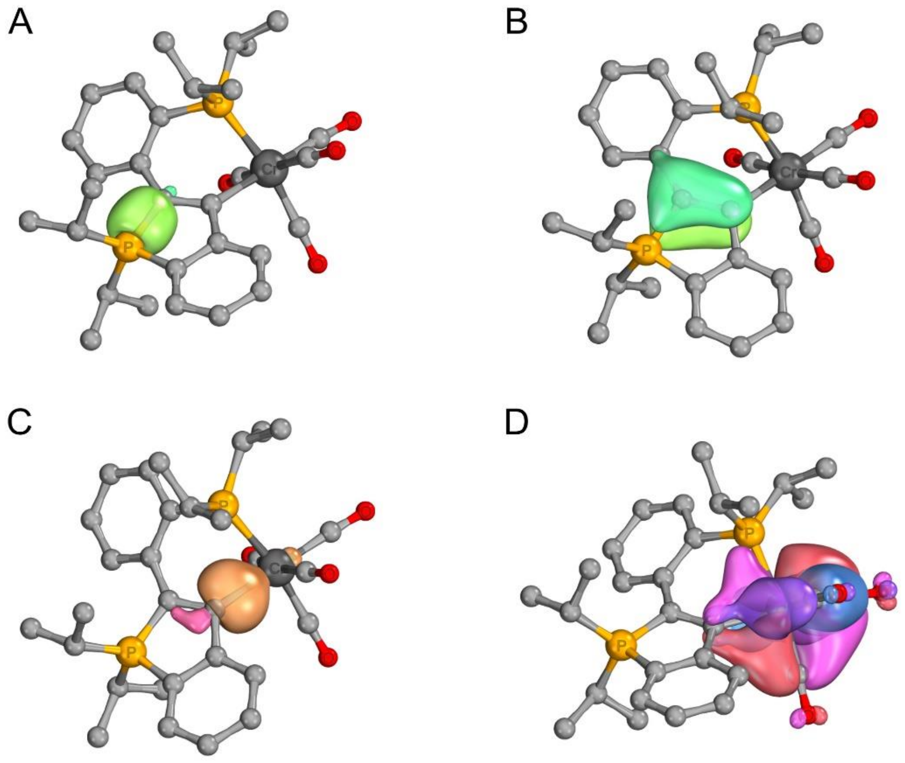

Figure S44: Selected IBO plots for complex **6** (A: *σ*-P-C bond, B: *π*-C=C-bond, C: dative *σ*-C-Cr bond, D: *π*-Cr-C backbonding via two orthogonal IBOs). Threshold value for printing: 75.0 for A - C, 85.0 for D.

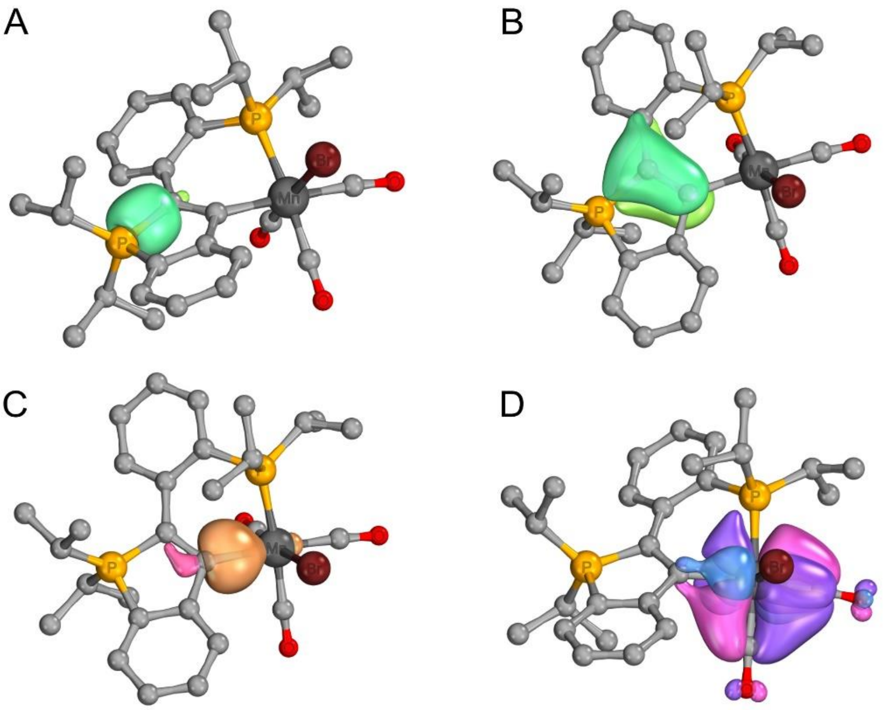

Figure S45: Selected IBO plots for complex **7** (A: σ-P-C bond, B: π-C=C-bond, C: dative σ-C-Mn bond, D: π-Mn-C backbonding via two orthogonal IBOs). Threshold value for printing: 75.0 for A - C, 85.0 for D.

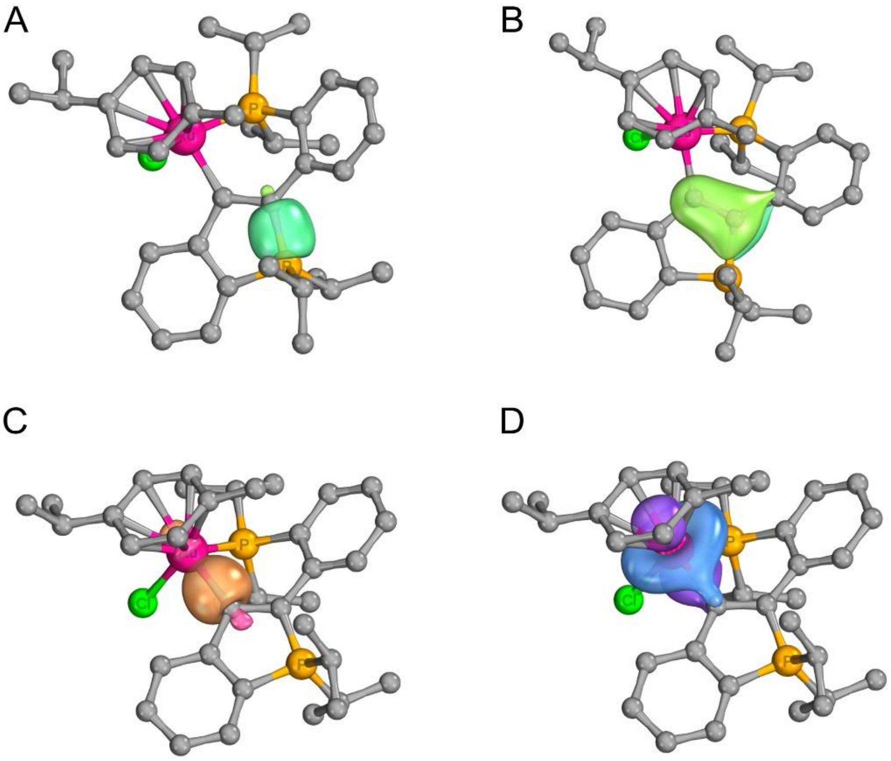

Figure S46: Selected IBO plots for complex **[8]**$^+$ (A: σ-P-C bond, B: π-C=C-bond, C: dative σ-C-Ru bond, D: π-Ru-C backbonding via one d_{z^2}-shaped IBO). Threshold value for printing: 75.0 for A - C, 85.0 for D.

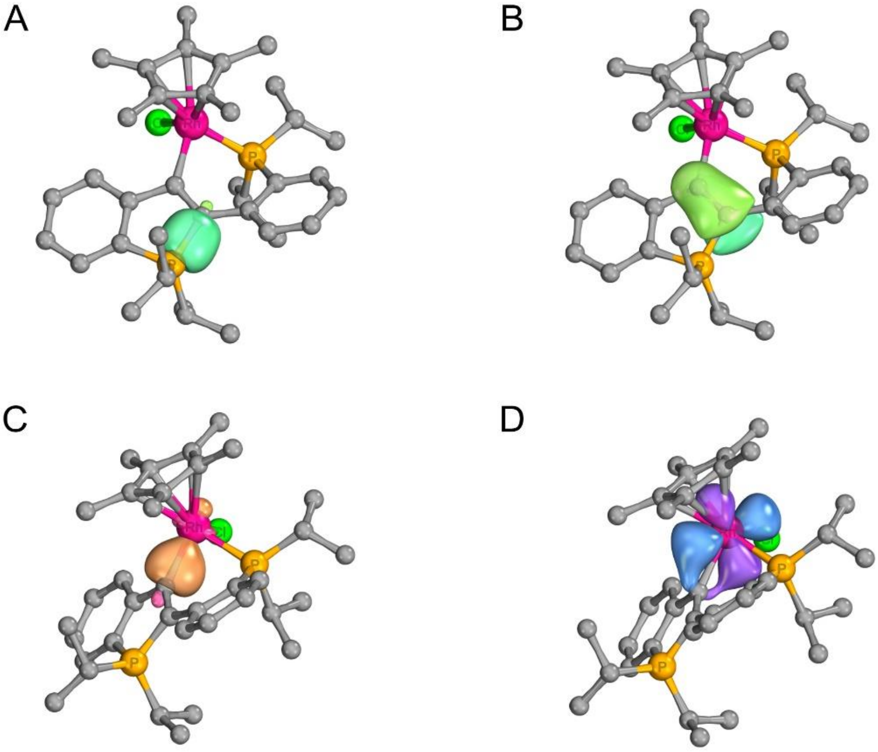

Figure S47: Selected IBO plots for complex **[9]+** (A: σ-P-C bond, B: π-C=C-bond, C: dative σ-C-Rh bond, D: π-Rh-C backbonding via one $d_{x2\text{-}y2}$-shaped IBO). Threshold value for printing: 75.0 for A - C, 85.0 for D.

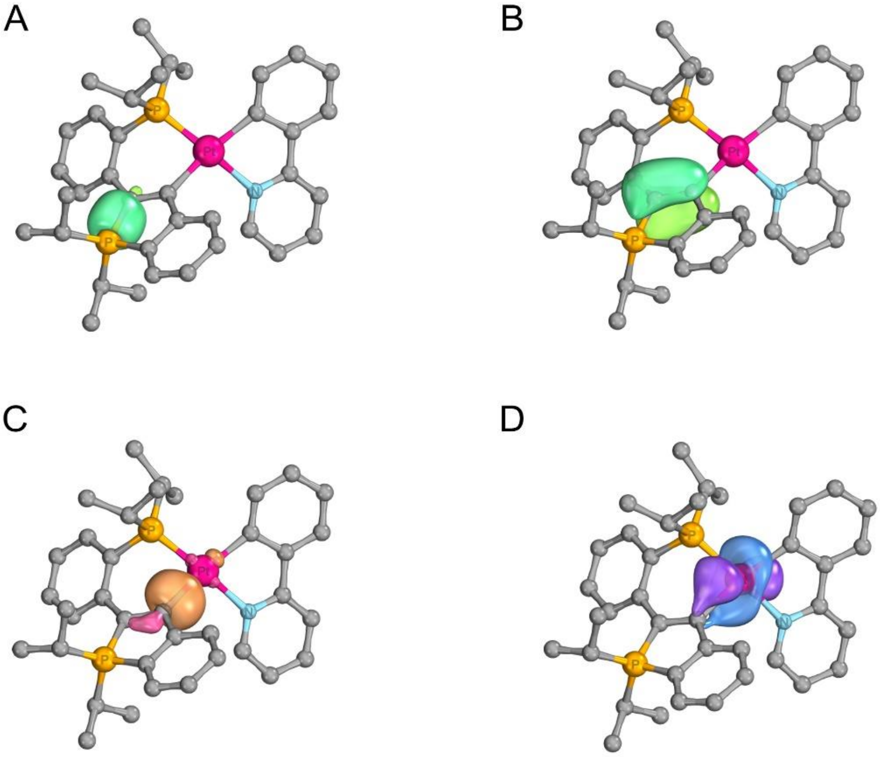

Figure S48: Selected IBO plots for complex **[10]**$^{+}$ (A: σ-P-C bond, B: π-C=C-bond, C: dative σ-C-Pt bond, D: π-Pt-C backbonding via one d_{z2}-shaped IBO). Threshold value for printing: 75.0 for A - C, 85.0 for D.

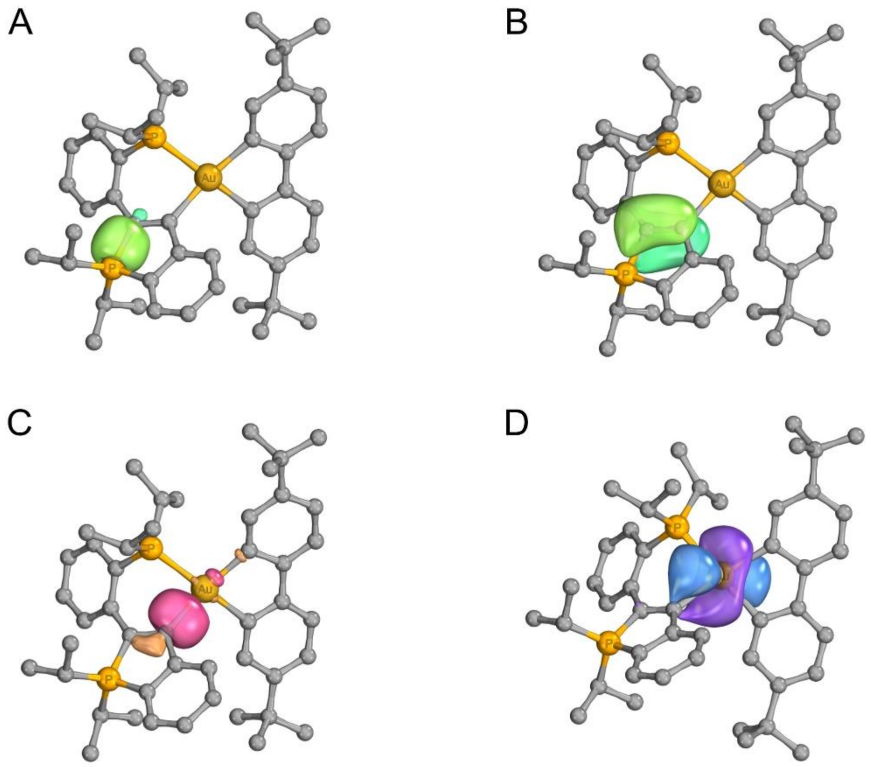

Figure S49: Selected IBO plots for complex **[11]$^+$** (A: σ-P-C bond, B: π-C=C-bond, C: dative σ-C-Au bond, D: π-Au-C backbonding via one d_{z2}-shaped IBO). Threshold value for printing: 75.0 for A - C, 95.0 for D.

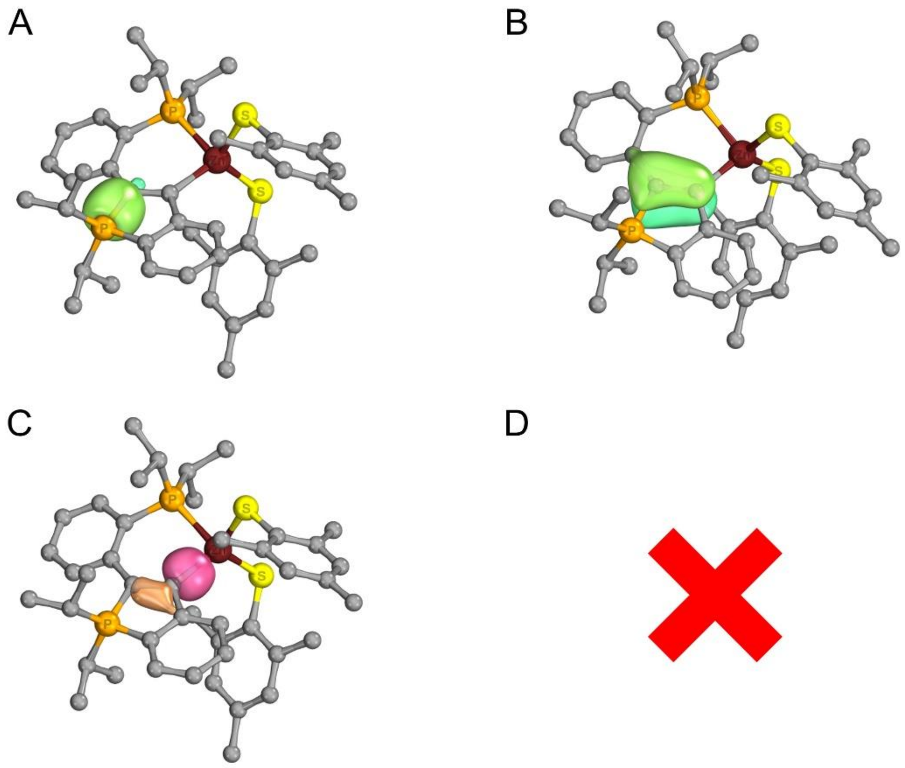

Figure S50: Selected IBO plots for complex **12** (A: σ-P-C bond, B: π-C=C-bond, C: dative σ-C-Zn bond, D: no π-Zn-C backbonding IBO was found). Threshold value for printing: 75% for A, B and C.

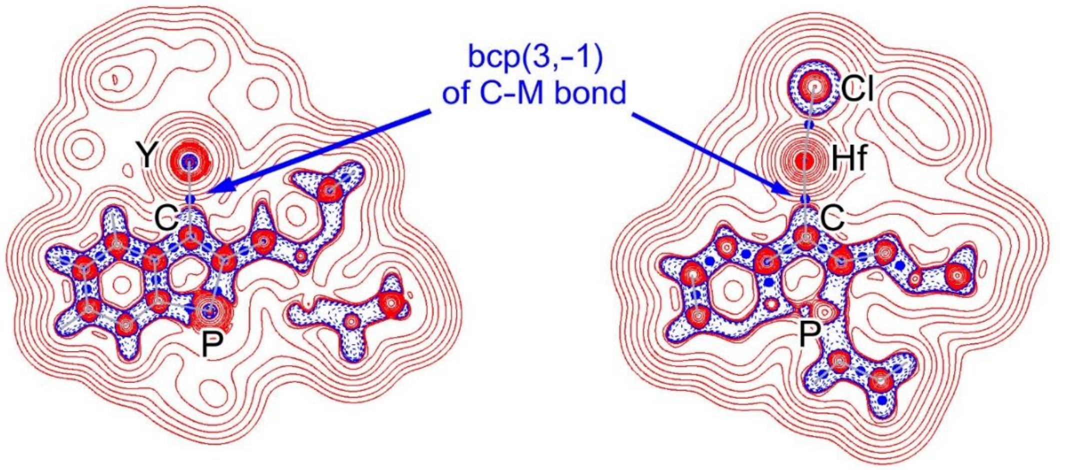

Figure S51: Counter plot of the laplacian of the electron densities ($\nabla^2\rho$) of **[3]**$^+$ (left) and **4** (right). Positive and negative values are shown in red and blue respectively. Selected electron and energy density values are summarised in Table 1 (see article).

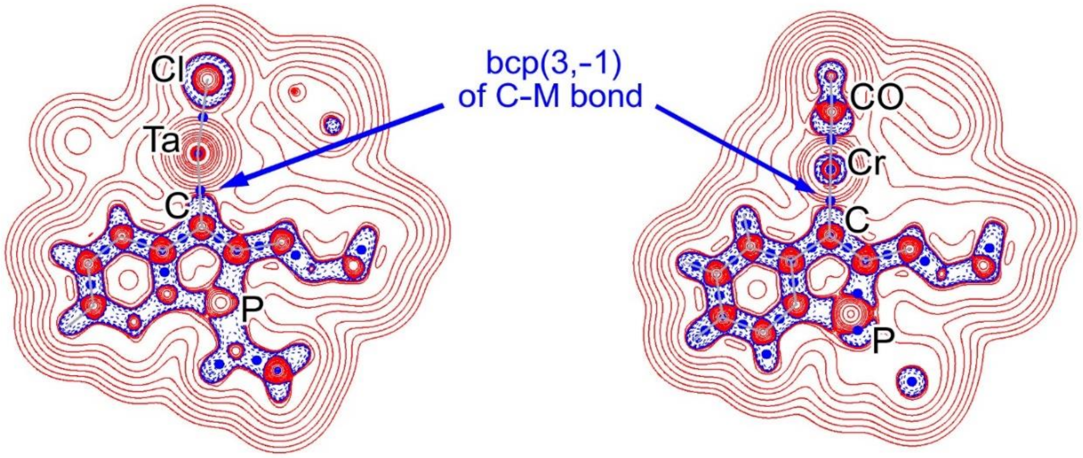

Figure S52: Counter plot of the laplacian of the electron densities ($\nabla^2\rho$) of **5** (left) and **6** (right). Positive and negative values are shown in red and blue respectively. Selected electron and energy density values are summarised in Table 1 (see article).

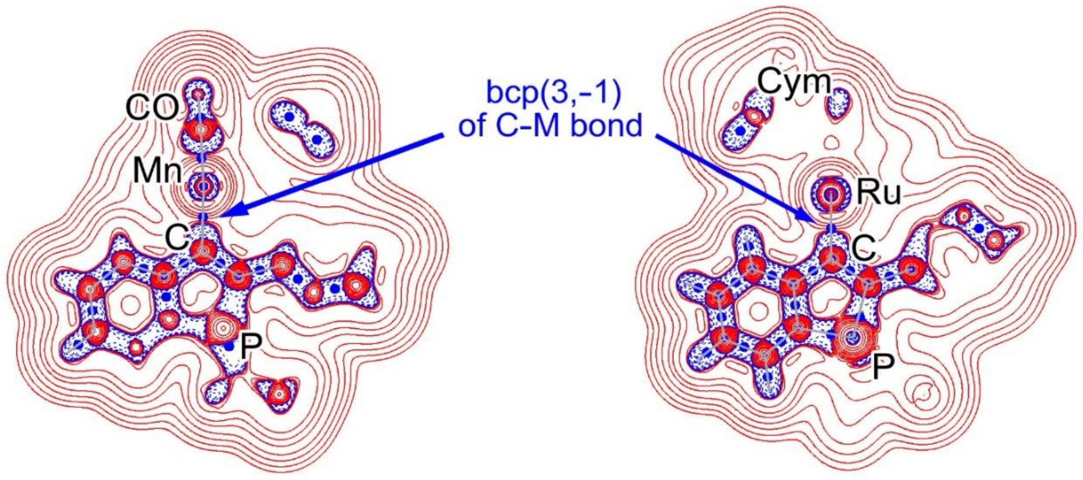

Figure S53: Counter plot of the laplacian of the electron densities ($\nabla^2\rho$) of **7** (left) and **[8]**$^+$ (right). Positive and negative values are shown in red and blue respectively. Selected electron and energy density values are summarised in Table 1 (see article).

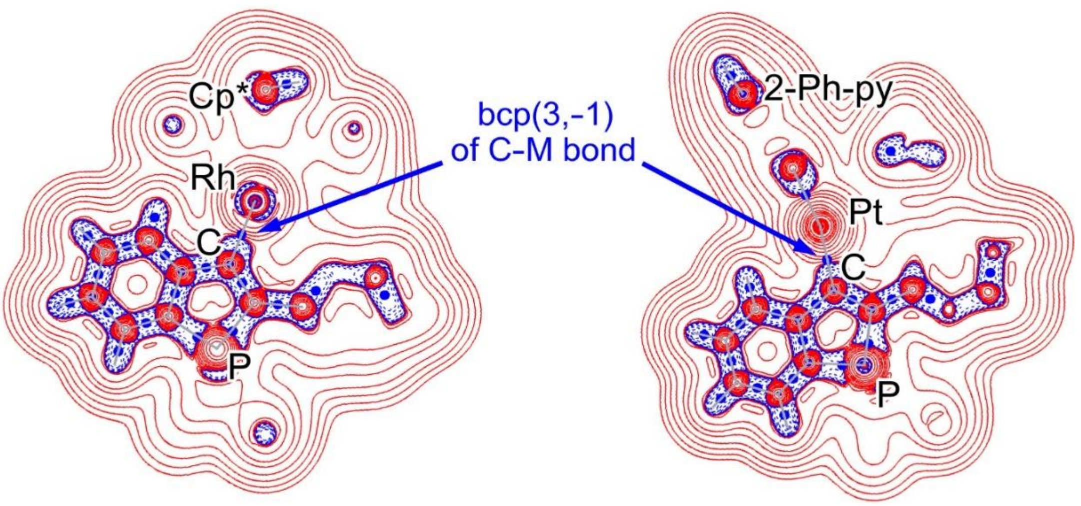

Figure S54: Counter plot of the laplacian of the electron densities ($\nabla^2\rho$) of **[9]⁺** (left) and **[10]⁺** (right). Positive and negative values are shown in red and blue respectively. Selected electron and energy density values are summarised in Table 1 (see article).

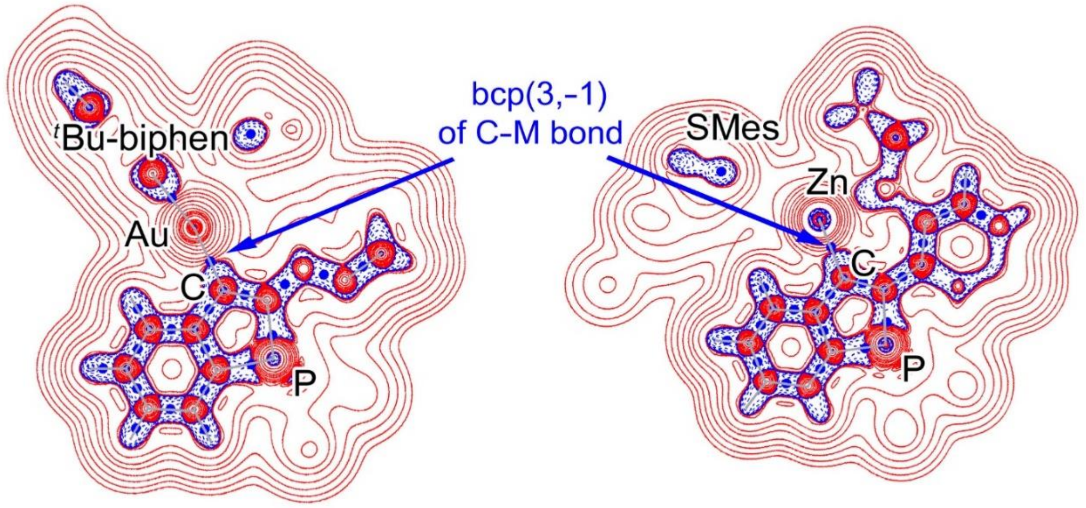

Figure S55: Counter plot of the laplacian of the electron densities ($\nabla^2\rho$) of **[11]⁺** (left) and **[12]⁺** (right). Positive and negative values are shown in red and blue respectively. Selected electron and energy density values are summarised in Table 1 (see article).

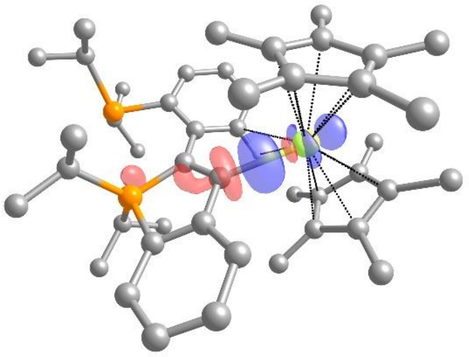

$\Delta\rho_1$: ΔE_1 = -25.7 $|\nu_1|$ = 0.552
C→Y σ-donation

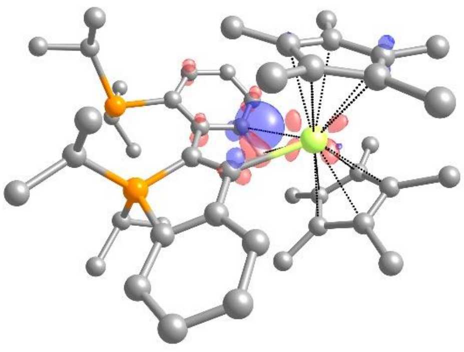

$\Delta\rho_2$: ΔE_2 = -7.5 $|\nu_2|$ = 0.301
not assigned to a specific interaction

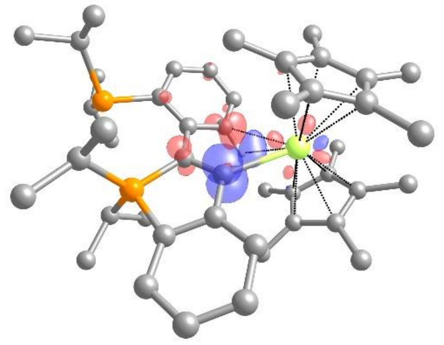

$\Delta\rho_3$: ΔE_3 = -5.8 $|\nu_3|$ = 0.237
not assigned to a specific interaction

Figure S56: Plot of the ETS-NOCV deformation densities $\Delta\rho_n$ for **[3]**$^+$ (isovalues: 0.005 for n=1, 0.002 for n=2, 0.0015 for n=3). All contributions ΔE_n to the orbital interaction energy (E_{orb}) with ΔE_n > –4 kcal/mol are shown (E_{orb} = –63.7 kcal/mol). The eigenvalues $|\nu_n|$ indicate the relative magnitude of the charge flow (charge flow: red → blue).

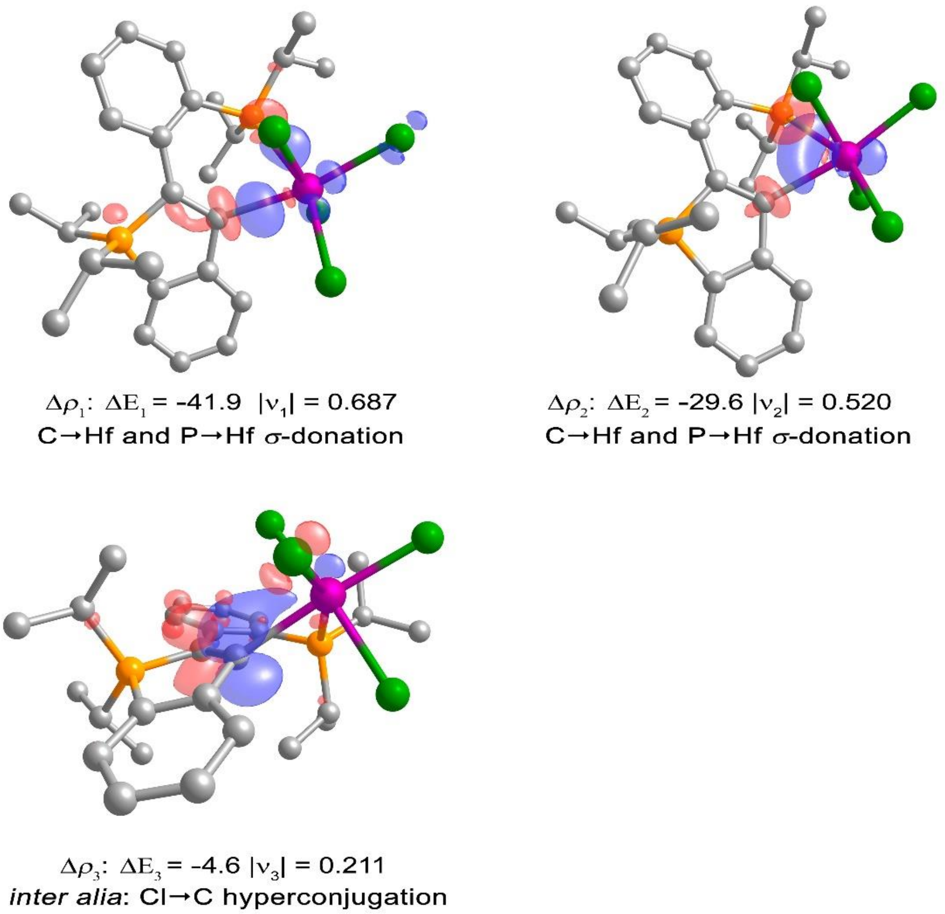

Figure S57: Plot of the ETS-NOCV deformation densities $\Delta\rho_n$ for **4** (isovalues: 0.004 for n=1, 0.004 for n=2, 0.001 for n=3). All contributions ΔE_n to the orbital interaction energy (E_{orb}) with $\Delta E_n > -4$ kcal/mol are shown (E_{orb} = –99.8 kcal/mol). The eigenvalues $|v_n|$ indicate the relative magnitude of the charge flow (charge flow: red → blue).

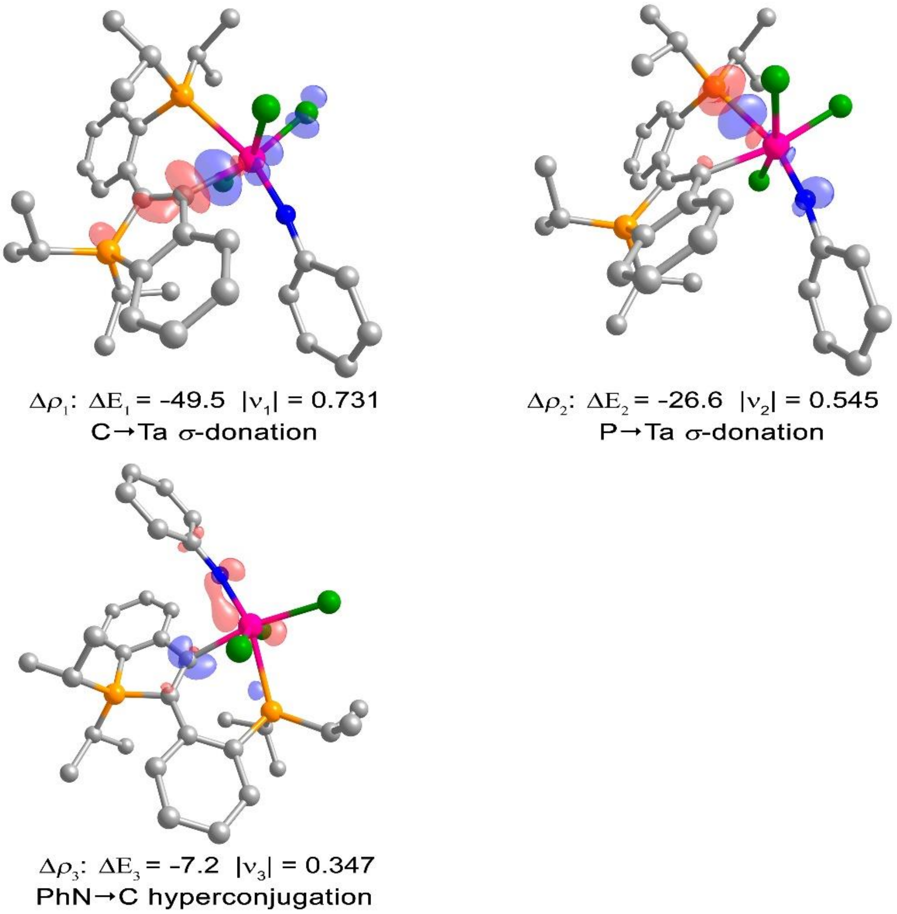

Figure S58: Plot of the ETS-NOCV deformation densities $\Delta\rho_n$ for **5** (isovalues: 0.005 for n=1, 0.005 for n=2, 0.0025 for n=3). All contributions ΔE_n to the orbital interaction energy (E_{orb}) with $\Delta E_n > -4$ kcal/mol are shown ($E_{orb} = -110.0$ kcal/mol). The eigenvalues $|\nu_n|$ indicate the relative magnitude of the charge flow (charge flow: red $\rightarrow$ blue).

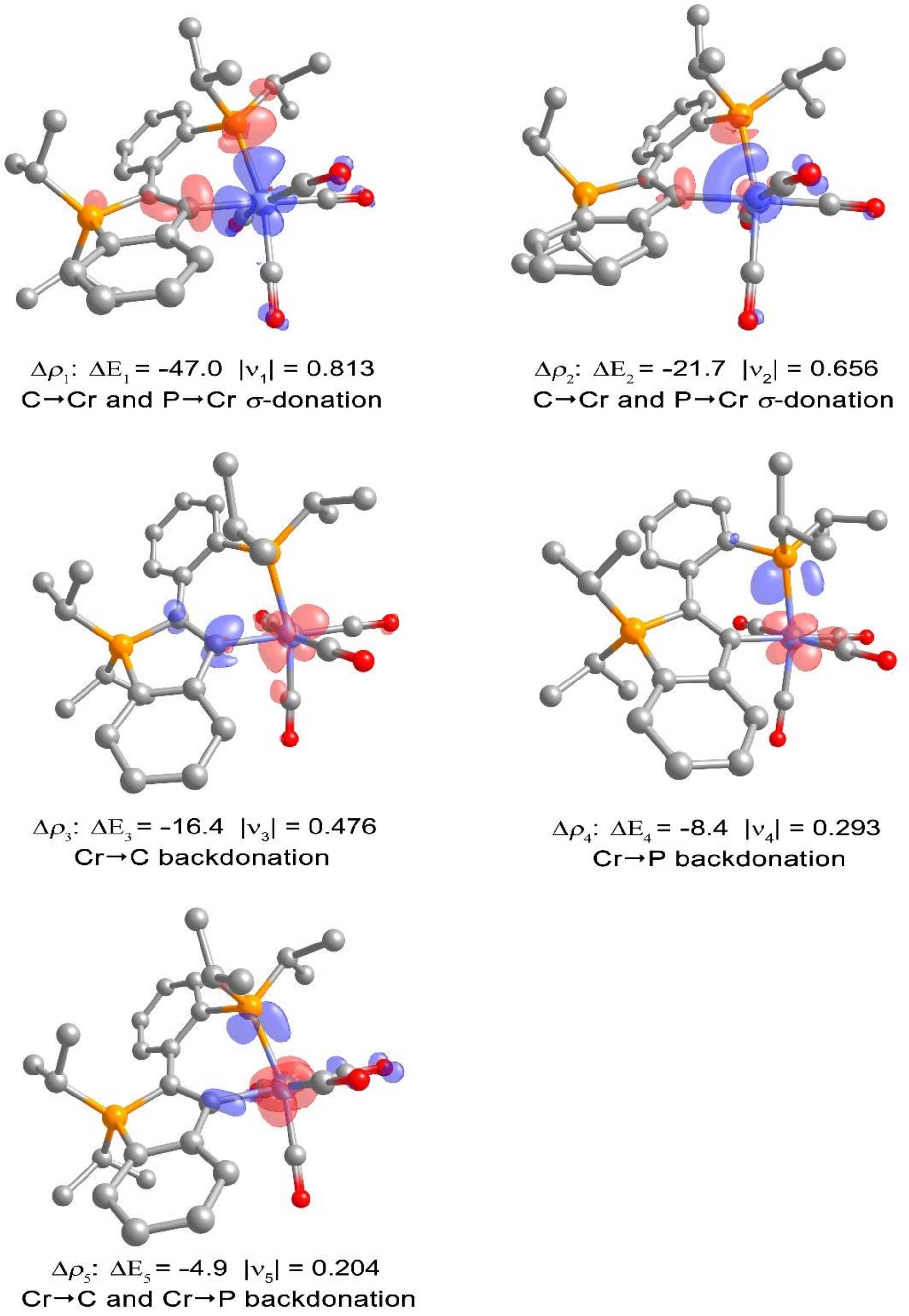

Figure S59: Plot of the ETS-NOCV deformation densities $\Delta\rho_n$ for **6** (isovalues: 0.004 for n=1, 0.004 for n=2, 0.004 for n=3, 0.002 for n= 4, 0.001 for n=5). All contributions ΔE_n to the orbital interaction energy (E_{orb}) with $\Delta E_n > -4$ kcal/mol are shown (E_{orb} = –110.2 kcal/mol). The eigenvalues $|\nu_n|$ indicate the relative magnitude of the charge flow (charge flow: red → blue).

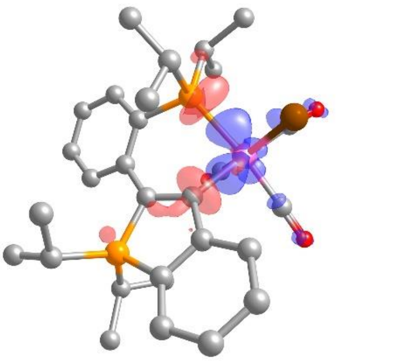

$\Delta\rho_1$: ΔE_1 = -61.1 $|\nu_1|$ = 0.955
C→Mn and P→Mn σ-donation

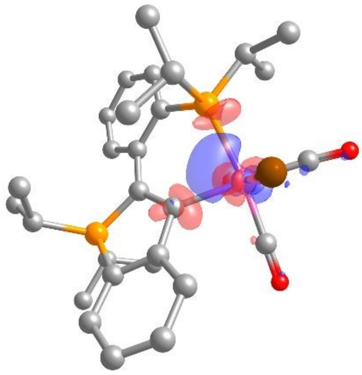

$\Delta\rho_2$: ΔE_2 = -27.0 $|\nu_2|$ = 0.585
C→Mn and P→Mn σ-donation

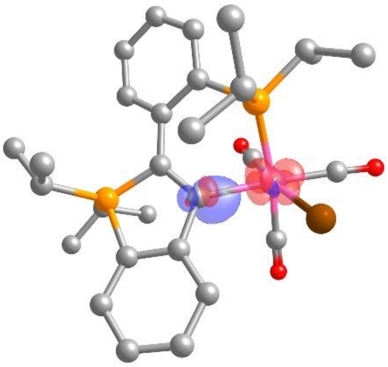

$\Delta\rho_3$: ΔE_3 = -13.7 $|\nu_3|$ = 0.502
Mn→C backdonation

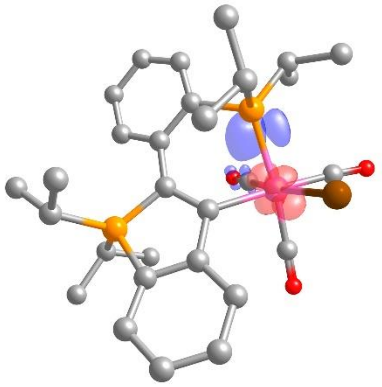

$\Delta\rho_4$: ΔE_4 = -7.2 $|\nu_4|$ = 0.258
Mn→P backdonation

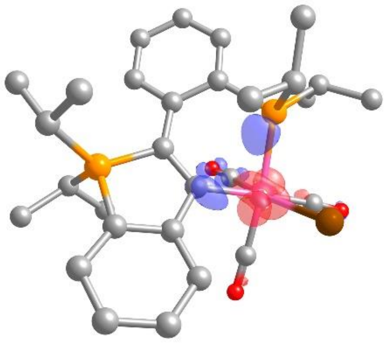

$\Delta\rho_5$: ΔE_5 = -5.3 $|\nu_5|$ = 0.209
Mn→C and Mn→P backdonation

Figure S60: Plot of the ETS-NOCV deformation densities $\Delta\rho_n$ for **7** (isovalues: 0.004 for n=1, 0.004 for n=2, 0.004 for n=3, 0.002 for n= 4, 0.0015 for n=5). All contributions ΔE_n to the orbital interaction energy (E_{orb}) with $\Delta E_n > -4$ kcal/mol are shown ($E_{orb} = -133.6$ kcal/mol). The eigenvalues $|\nu_n|$ indicate the relative magnitude of the charge flow (charge flow: red → blue).

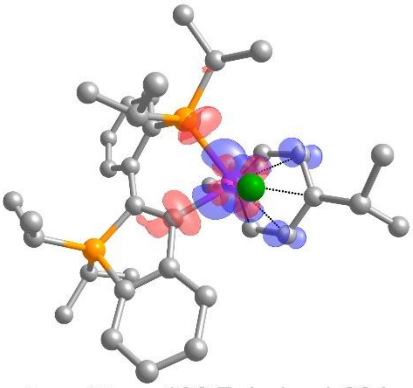

$\Delta\rho_1$: ΔE_1 = -106.7 $|\nu_1|$ = 1.384
C→Ru and P→Ru σ-donation

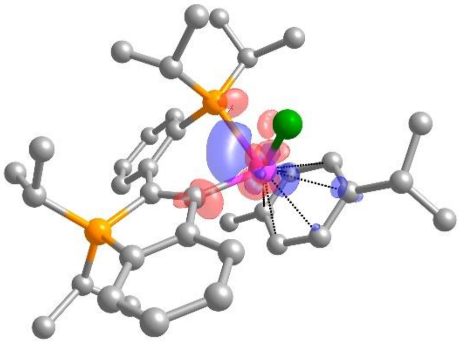

$\Delta\rho_2$: ΔE_2 = -39.2 $|\nu_2|$ = 0.714
C→Ru and P→Ru σ-donation

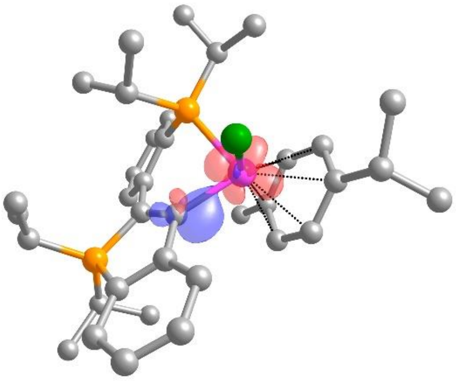

$\Delta\rho_3$: ΔE_3 = -21.6 $|\nu_3|$ = 0.559
Ru→C backdonation

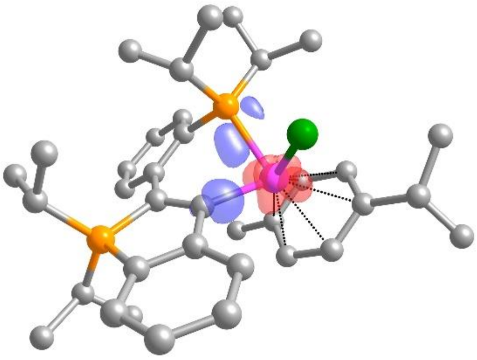

$\Delta\rho_4$: ΔE_4 = -11.7 $|\nu_4|$ = 0.325
Ru→C and Ru→P backdonation

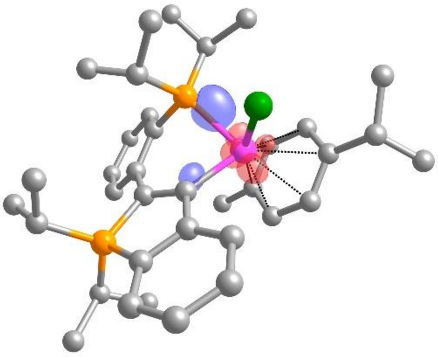

$\Delta\rho_5$: ΔE_5 = -10.0 $|\nu_5|$ = 0.264
Ru→C and Ru→P backdonation

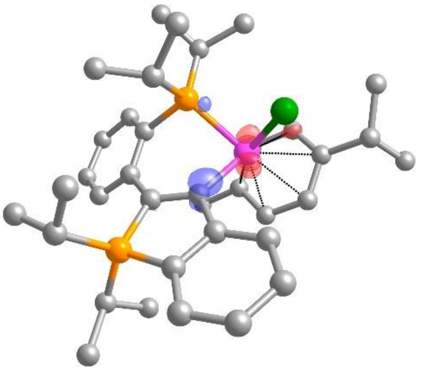

$\Delta\rho_6$: ΔE_6 = -5.7 $|\nu_6|$ = 0.209
Ru→C and Ru→P backdonation

Figure S61: Plot of the ETS-NOCV deformation densities $\Delta\rho_n$ for **[8]**$^+$ (isovalues: 0.008 for n=1, 0.006 for n=2, 0.005 for n=3, 0.003 for n= 4, 0.003 for n=5, 0.003 for n=6). All contributions ΔE_n to the orbital interaction energy (E_{orb}) with ΔE_n > –4 kcal/mol are shown (E_{orb} = –221.3 kcal/mol). The eigenvalues $|\nu_n|$ indicate the relative magnitude of the charge flow (charge flow: red → blue).

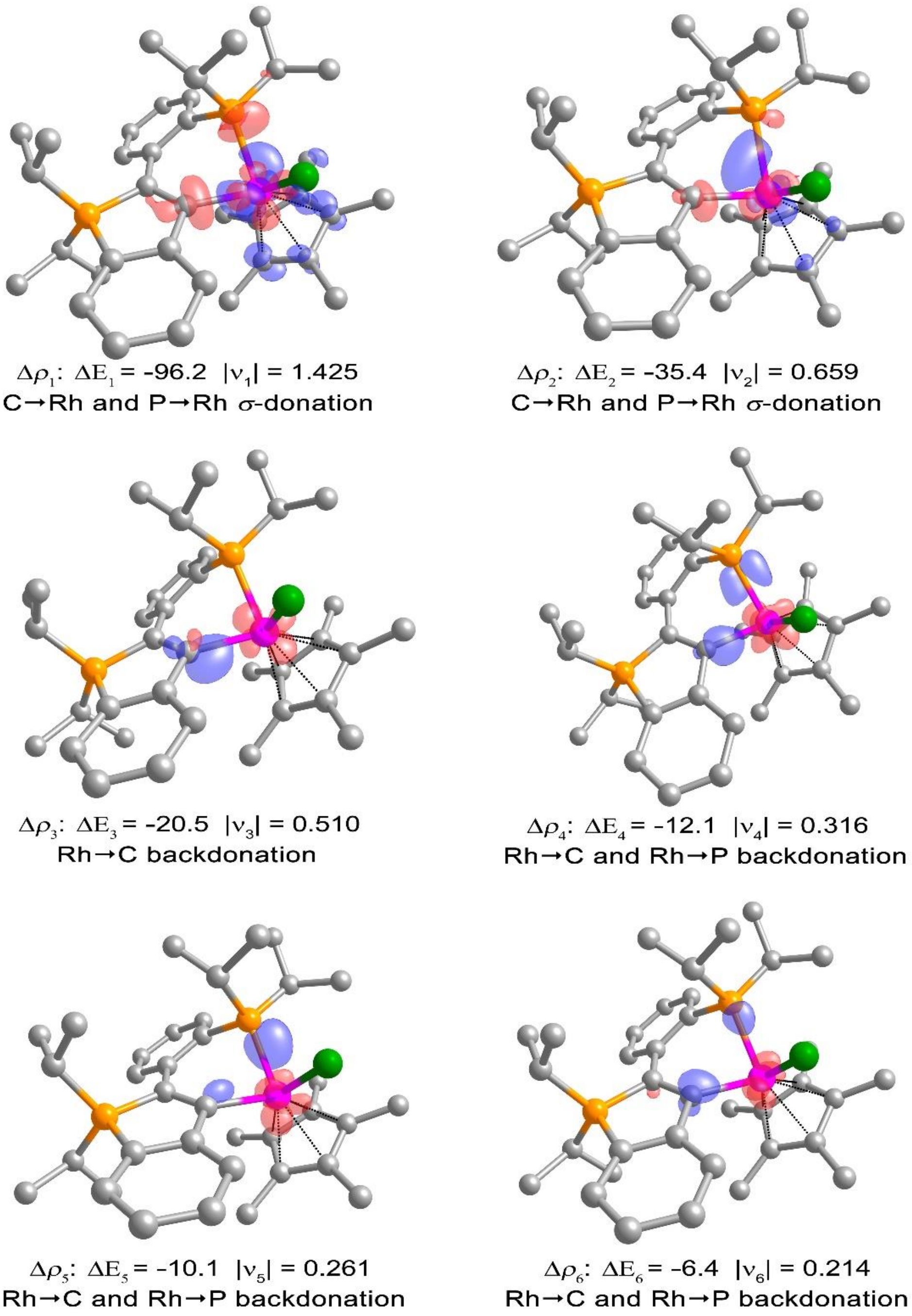

Figure S62: Plot of the ETS-NOCV deformation densities $\Delta\rho_n$ for **[9]**$^+$ (isovalues: 0.008 for n=1, 0.007 for n=2, 0.006 for n=3, 0.0025 for n= 4, 0.0025 for n=5, 0.0025 for n=6). All contributions ΔE_n to the orbital interaction energy (E_{orb}) with ΔE_n > –4 kcal/mol are shown (E_{orb} = –207.4 kcal/mol). The eigenvalues $|\nu_n|$ indicate the relative magnitude of the charge flow (charge flow: red $\rightarrow$ blue).

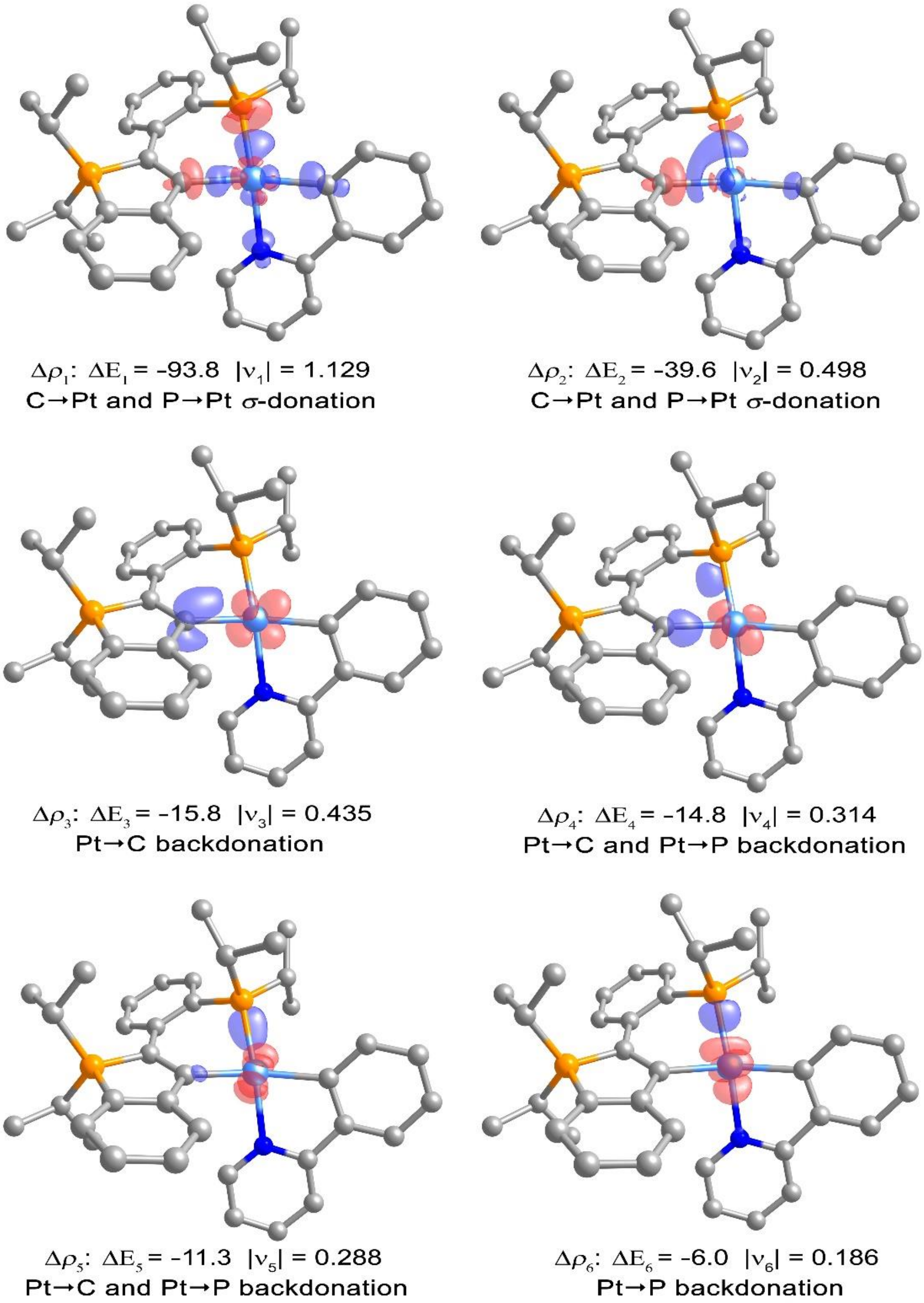

Figure S63: Plot of the ETS-NOCV deformation densities $\Delta\rho_n$ for **[10]⁺** (isovalues: 0.009 for n=1, 0.006 for n=2, 0.004 for n=3, 0.004 for n= 4, 0.004 for n=5, 0.002 for n=6). All contributions ΔE_n to the orbital interaction energy (E_{orb}) with ΔE_n > –4 kcal/mol are shown (E_{orb} = –206.8 kcal/mol). The eigenvalues $|v_n|$ indicate the relative magnitude of the charge flow (charge flow: red → blue).

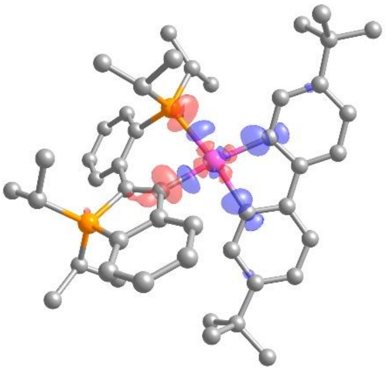

$\Delta\rho_1$: ΔE_1 = -70.8 $|\nu_1|$ = 1.027
C→Au and P→Au σ-donation

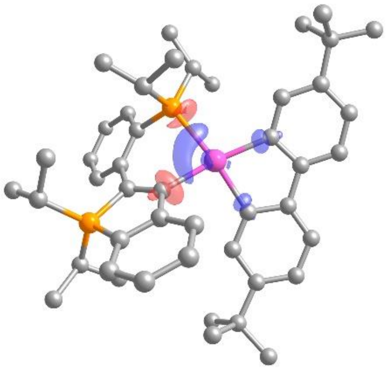

$\Delta\rho_2$: ΔE_2 = -47.0 $|\nu_2|$ = 0.577
C→Au and P→Au σ-donation

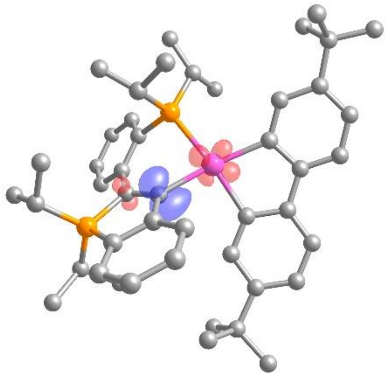

$\Delta\rho_3$: ΔE_3 = -9.2 $|\nu_3|$ = 0.288
Au→C backdonation

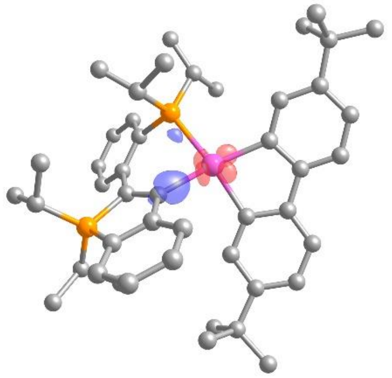

$\Delta\rho_4$: ΔE_4 = -8.8 $|\nu_4|$ = 0.232
Au→C and Au→P backdonation

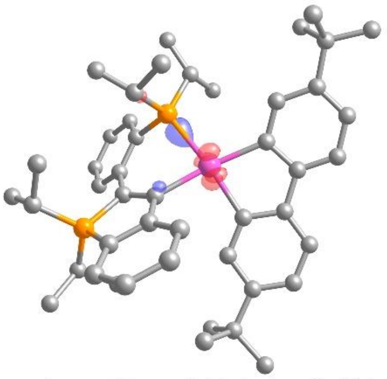

$\Delta\rho_5$: ΔE_5 = -5.5 $|\nu_5|$ = 0.191
Au→C and Au→P backdonation

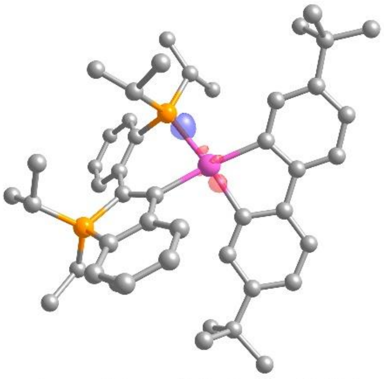

$\Delta\rho_6$: ΔE_6 = -4.6 $|\nu_6|$ = 0.170
Au→P backdonation

Figure S64: Plot of the ETS-NOCV deformation densities $\Delta\rho_n$ for **[11]**$^+$ (isovalues: 0.007 for n=1, 0.007 for n=2, 0.003 for n=3, 0.003 for n= 4, 0.003 for n=5, 0.003 for n=6). All contributions ΔE_n to the orbital interaction energy (E_{orb}) with ΔE_n > –4 kcal/mol are shown (E_{orb} = –175.1 kcal/mol). The eigenvalues $|\nu_n|$ indicate the relative magnitude of the charge flow (charge flow: red $\rightarrow$ blue).

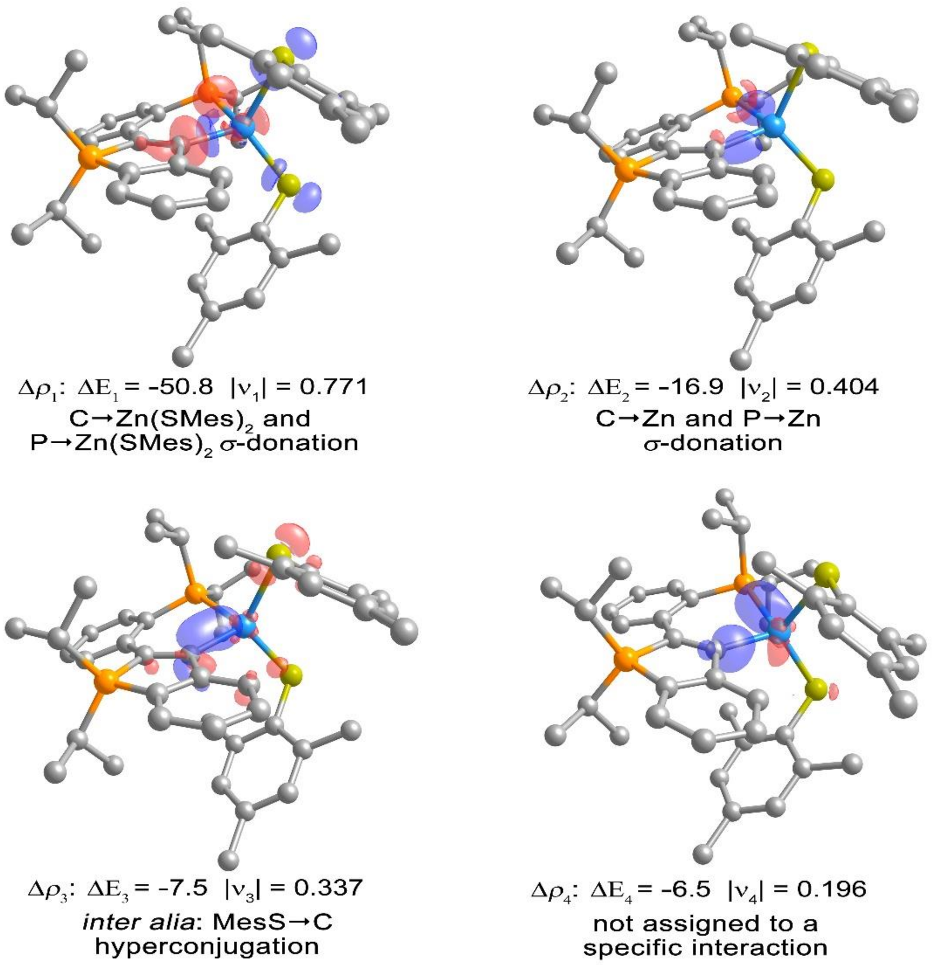

Figure S65: Plot of the ETS-NOCV deformation densities $\Delta\rho_n$ for **12** (isovalues: 0.009 for n=1, 0.005 for n=2, 0.002 for n=3, 0.001 for n= 4). All contributions ΔE_n to the orbital interaction energy (E_{orb}) with $\Delta E_n > -4$ kcal/mol are shown (E_{orb} = –105.4 kcal/mol). The eigenvalues $|v_n|$ indicate the relative magnitude of the charge flow (charge flow: red → blue).

Optimised geometries (PBE1PBE/Def2-TZVP)
[3]+

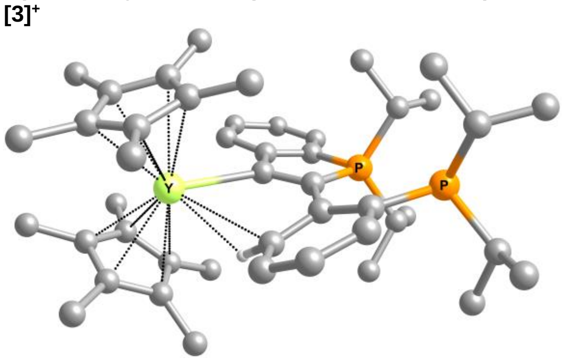

Y	2.286595000	-0.372933000	-0.005667000
P	-2.326519000	1.841478000	0.067512000
P	-4.124240000	-1.144342000	-0.004396000
C	-1.127633000	0.476812000	0.176462000
C	0.133210000	0.964797000	-0.066843000
C	0.148667000	2.414668000	-0.320093000
C	1.268514000	3.153895000	-0.684798000
H	2.223733000	2.661169000	-0.829839000
C	1.176714000	4.527602000	-0.862843000
H	2.057616000	5.094621000	-1.141281000
C	-0.032708000	5.183123000	-0.682433000
H	-0.093100000	6.257091000	-0.811076000
C	-1.173019000	4.461988000	-0.344481000
H	-2.115007000	4.981458000	-0.221527000
C	-1.070614000	3.092530000	-0.167485000
C	-1.320783000	-0.949932000	0.538421000
C	-2.436251000	-1.791118000	0.320333000
C	-2.270576000	-3.174001000	0.465713000
H	-3.100553000	-3.822495000	0.217788000
C	-1.101030000	-3.753290000	0.918787000
H	-1.020641000	-4.829068000	1.018055000
C	-0.068457000	-2.917360000	1.314450000
H	0.819405000	-3.323665000	1.786164000
C	-0.189523000	-1.555899000	1.122605000
C	-3.362467000	1.910665000	-1.460621000
H	-3.944157000	0.989587000	-1.396436000
C	-4.330505000	3.085901000	-1.499799000
H	-5.066401000	3.047822000	-0.696280000
H	-4.879823000	3.042850000	-2.443657000
H	-3.820735000	4.051069000	-1.472295000
C	-2.476259000	1.867774000	-2.698171000
H	-1.889516000	2.781339000	-2.810214000
H	-3.110996000	1.769183000	-3.582043000
H	-1.792750000	1.017853000	-2.684229000
C	-3.283745000	2.129686000	1.611041000
H	-4.189249000	1.530196000	1.474997000
C	-3.661181000	3.592730000	1.831260000
H	-2.775227000	4.198146000	2.030410000
H	-4.304186000	3.654343000	2.712385000
H	-4.204948000	4.036626000	0.999008000
C	-2.495492000	1.611015000	2.810187000
H	-2.265530000	0.548837000	2.735550000
H	-3.082706000	1.768727000	3.717992000
H	-1.558425000	2.161095000	2.924991000
C	-4.690243000	-2.121277000	-1.483109000
H	-4.578170000	-3.186999000	-1.256795000
C	-6.162214000	-1.834776000	-1.758924000
H	-6.811940000	-2.140684000	-0.937185000
H	-6.486839000	-2.375499000	-2.651876000
H	-6.330246000	-0.769074000	-1.940933000
C	-3.836874000	-1.804520000	-2.703427000
H	-3.945900000	-0.759685000	-3.006219000
H	-4.152734000	-2.420916000	-3.549340000
H	-2.778029000	-1.999501000	-2.526574000
C	-5.176497000	-1.842433000	1.381613000
H	-6.140467000	-1.368491000	1.155668000
C	-5.407834000	-3.345805000	1.461260000
H	-5.691013000	-3.790880000	0.505814000
H	-6.222254000	-3.546323000	2.163453000
H	-4.528263000	-3.868786000	1.840826000
C	-4.711864000	-1.309784000	2.730974000
H	-3.719668000	-1.690993000	2.986965000
H	-5.399375000	-1.633739000	3.516706000
H	-4.675583000	-0.219777000	2.755471000
C	1.500823000	-1.491226000	-2.258426000
C	2.492454000	-2.355703000	-1.727469000
C	3.741519000	-1.687408000	-1.795894000
C	3.518824000	-0.395668000	-2.344034000
C	2.134551000	-0.279939000	-2.638191000
C	0.080439000	-1.849583000	-2.540062000
H	-0.029904000	-2.220501000	-3.565316000
H	-0.580375000	-0.987739000	-2.433593000
H	-0.285555000	-2.631344000	-1.872053000
C	2.310513000	-3.789158000	-1.340336000
H	1.274937000	-4.015222000	-1.082662000
H	2.936514000	-4.073438000	-0.490041000
H	2.588314000	-4.452088000	-2.167268000
C	5.062981000	-2.353223000	-1.593533000
H	5.095025000	-2.967992000	-0.691203000
H	5.882618000	-1.638640000	-1.542438000
H	5.273402000	-3.022667000	-2.434932000
C	4.569308000	0.587931000	-2.752600000
H	5.496178000	0.455675000	-2.193969000
H	4.248094000	1.624319000	-2.618474000
H	4.817536000	0.473421000	-3.813264000
C	1.503018000	0.828388000	-3.415839000
H	1.510834000	0.596729000	-4.486911000
H	2.033823000	1.774290000	-3.294560000
H	0.464250000	0.995592000	-3.128852000
C	4.547416000	0.009307000	1.342959000
C	3.841866000	1.238018000	1.429704000
C	2.697755000	1.033242000	2.244923000
C	2.691236000	-0.328008000	2.648295000
C	3.824357000	-0.966276000	2.076122000
C	5.945673000	-0.125115000	0.837863000
H	6.226234000	-1.162144000	0.661256000
H	6.644531000	0.272960000	1.582413000
H	6.118053000	0.435026000	-0.082614000
C	4.375237000	2.553729000	0.955990000
H	4.749181000	2.515871000	-0.071394000
H	5.217663000	2.871047000	1.580201000
H	3.624320000	3.342324000	1.012352000
C	1.803393000	2.095886000	2.795714000
H	1.721936000	2.955437000	2.129697000
H	2.188591000	2.462564000	3.753686000
H	0.793260000	1.724080000	2.977439000
C	1.829216000	-0.907539000	3.725762000
H	1.678867000	-1.983087000	3.614606000
H	0.844634000	-0.438415000	3.775849000
H	2.300877000	-0.757643000	4.703098000
C	4.279496000	-2.358745000	2.379523000
H	4.932967000	-2.756126000	1.601510000
H	3.444781000	-3.054868000	2.495573000
H	4.845141000	-2.390735000	3.317146000
H	0.552674000	-0.896166000	1.571226000

4

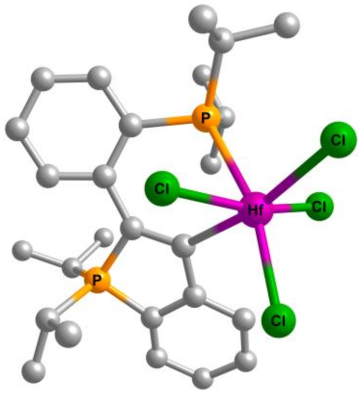

Hf	-1.529999000	-1.223474000	-0.402375000
Cl	-3.886342000	-1.415882000	-0.889933000
Cl	-0.622271000	-3.290235000	-1.194337000
Cl	-1.122855000	0.013783000	-2.460666000
Cl	-1.938602000	-1.840136000	1.932033000
P	-1.748022000	1.375472000	0.648555000
P	3.132348000	0.330733000	-0.127611000
C	-0.466860000	2.425608000	-0.143938000
C	-0.785755000	3.742262000	-0.465961000
H	-1.747451000	4.140797000	-0.173096000
C	0.071701000	4.573008000	-1.170157000
H	-0.224693000	5.588599000	-1.404132000
C	1.293061000	4.075544000	-1.579503000
H	1.977302000	4.686451000	-2.157272000
C	1.640267000	2.781067000	-1.240192000
H	2.603060000	2.417091000	-1.575189000
C	0.804400000	1.924873000	-0.517092000
C	1.341604000	0.613353000	-0.118590000
C	0.718473000	-0.546830000	0.240041000
C	1.668477000	-1.553446000	0.789360000
C	3.032755000	-1.234844000	0.688854000
C	4.035623000	-2.049859000	1.182550000
H	5.081668000	-1.777432000	1.092244000
C	3.676237000	-3.231460000	1.817631000
H	4.440380000	-3.884956000	2.221489000
C	2.334579000	-3.564399000	1.936580000
H	2.054783000	-4.484827000	2.436295000
C	1.335140000	-2.744604000	1.428106000
H	0.298332000	-3.026028000	1.543143000
C	-3.356930000	2.239922000	0.302626000
H	-3.250437000	3.272705000	0.648712000
C	-3.701852000	2.251677000	-1.182213000
H	-2.912064000	2.688563000	-1.794578000
H	-4.613869000	2.837360000	-1.331439000
H	-3.885119000	1.241734000	-1.549354000
C	-4.475319000	1.592969000	1.117618000
H	-4.581704000	0.534046000	0.876786000
H	-5.420874000	2.085846000	0.874806000
H	-4.321180000	1.689017000	2.193845000
C	-1.532016000	1.635759000	2.481662000
H	-2.304011000	0.981786000	2.901646000
C	-0.186723000	1.138764000	2.989233000
H	-0.029388000	0.087341000	2.753424000
H	-0.149077000	1.253434000	4.076616000
H	0.633532000	1.722366000	2.565493000
C	-1.772988000	3.068915000	2.937266000
H	-1.056959000	3.757797000	2.480501000
H	-1.639222000	3.130319000	4.021364000
H	-2.779617000	3.424920000	2.714916000
C	3.794204000	0.072881000	-1.827597000
H	3.885034000	1.070971000	-2.267796000
C	2.810630000	-0.745511000	-2.657206000
H	2.649257000	-1.734166000	-2.221552000
H	3.224667000	-0.885544000	-3.658728000
H	1.837078000	-0.262978000	-2.752095000
C	5.178945000	-0.566298000	-1.789646000
H	5.899877000	0.006211000	-1.201332000
H	5.566183000	-0.631605000	-2.809182000
H	5.131397000	-1.581323000	-1.391127000
C	4.280609000	1.399229000	0.832077000
H	5.122390000	0.716123000	1.007573000
C	3.655247000	1.747664000	2.178026000
H	2.831475000	2.452345000	2.049942000
H	4.405209000	2.219543000	2.817355000
H	3.274758000	0.866035000	2.696757000
C	4.809448000	2.633255000	0.112851000
H	5.241785000	2.402609000	-0.862778000
H	5.597503000	3.084714000	0.720778000
H	4.028503000	3.382156000	-0.022185000

5

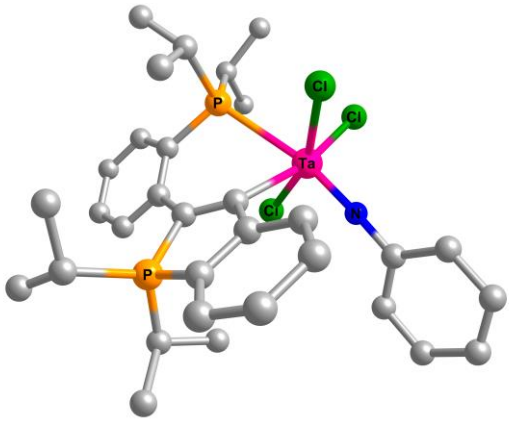

Ta	-0.205280000	-1.698306000	-0.058397000
Cl	0.396875000	-1.055138000	-2.296344000
Cl	-0.069091000	-2.025990000	2.331168000
Cl	0.436041000	-3.969811000	-0.446652000
P	-0.409149000	3.141728000	-0.031798000
P	2.517386000	-0.954014000	0.366275000
N	-1.978116000	-1.750922000	-0.302475000
C	0.491814000	1.574755000	-0.151290000
C	-0.287967000	0.586862000	0.385407000
C	-1.462410000	1.123991000	1.129263000
C	-1.659277000	2.511962000	1.046730000
C	-2.681799000	3.170294000	1.706448000
H	-2.806503000	4.244726000	1.625645000
C	-3.550644000	2.425445000	2.492423000
H	-4.355210000	2.915575000	3.027652000
C	-3.381140000	1.051648000	2.584871000
H	-4.063117000	0.468795000	3.193259000
C	-2.355569000	0.399028000	1.913061000
H	-2.242255000	-0.670311000	2.007465000
C	1.804717000	1.544848000	-0.822241000
C	2.814155000	0.573749000	-0.608467000
C	4.050923000	0.751116000	-1.223883000
H	4.831226000	0.020673000	-1.058498000
C	4.330062000	1.818676000	-2.062213000
H	5.306171000	1.908209000	-2.524201000
C	3.339024000	2.748972000	-2.305307000
H	3.510890000	3.584121000	-2.974841000
C	2.114055000	2.604862000	-1.681105000
H	1.356509000	3.346985000	-1.895951000
C	-1.209148000	3.628879000	-1.621276000
H	-0.415257000	4.063660000	-2.236219000
C	-2.281964000	4.690826000	-1.401742000
H	-3.116302000	4.292364000	-0.821914000
H	-2.675101000	5.001451000	-2.372718000
H	-1.903595000	5.584874000	-0.900761000
C	-1.763822000	2.397908000	-2.328900000
H	-0.994554000	1.653028000	-2.537886000
H	-2.212688000	2.706607000	-3.276266000
H	-2.542063000	1.916924000	-1.731882000
C	0.336827000	4.625985000	0.754249000
H	-0.560659000	5.174305000	1.070949000
C	1.151918000	5.532683000	-0.158665000
H	2.089999000	5.061957000	-0.454232000
H	1.398257000	6.448876000	0.383890000
H	0.610270000	5.823531000	-1.060884000
C	1.116659000	4.222750000	1.999937000
H	0.544233000	3.558765000	2.649879000
H	1.375971000	5.116816000	2.571996000
H	2.044724000	3.718019000	1.727161000
C	3.818055000	-2.149263000	-0.212011000
H	4.789793000	-1.654124000	-0.118640000
C	3.830704000	-3.369819000	0.706909000
H	4.094050000	-3.118441000	1.735975000
H	4.574708000	-4.083187000	0.340949000
H	2.861482000	-3.870622000	0.711042000
C	3.615625000	-2.565101000	-1.664506000
H	2.682190000	-3.114985000	-1.786556000
H	4.438513000	-3.219849000	-1.967261000
H	3.590088000	-1.713009000	-2.345144000
C	3.102864000	-0.526426000	2.085926000
H	2.865083000	-1.436378000	2.647744000
C	2.300404000	0.612672000	2.696490000
H	2.476253000	1.544585000	2.154793000
H	2.619191000	0.766443000	3.731821000
H	1.232036000	0.401055000	2.698525000
C	4.593591000	-0.234891000	2.193086000
H	5.216904000	-1.076617000	1.889073000
H	4.844235000	-0.003809000	3.232941000
H	4.872922000	0.633608000	1.589864000
C	-3.318008000	-1.742734000	-0.545867000
C	-3.829974000	-1.162258000	-1.715875000
H	-3.131517000	-0.738168000	-2.428070000
C	-5.191910000	-1.155629000	-1.958668000
H	-5.569667000	-0.708145000	-2.872036000
C	-6.075429000	-1.724386000	-1.049477000
H	-7.141325000	-1.720276000	-1.245906000
C	-5.577625000	-2.309514000	0.107330000
H	-6.257323000	-2.768088000	0.817671000
C	-4.217029000	-2.322002000	0.361296000
H	-3.820974000	-2.792235000	1.254180000

6

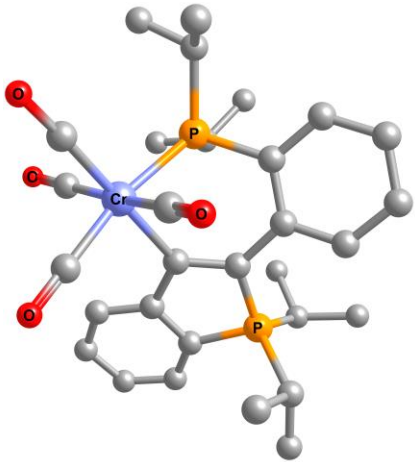

Cr	1.516563000	-1.277946000	-0.636412000
P	2.018765000	0.683255000	0.543275000
P	-2.754326000	0.452199000	0.011669000
O	4.240447000	-2.075191000	-1.589137000
O	1.336636000	0.281473000	-3.215941000
O	2.270789000	-3.239011000	1.526149000
O	0.369562000	-3.549521000	-2.206335000
C	1.029930000	2.040673000	-0.201573000
C	1.568130000	3.298079000	-0.455978000
H	2.594947000	3.504329000	-0.183598000
C	0.836262000	4.310362000	-1.059474000
H	1.295564000	5.272632000	-1.253054000
C	-0.476513000	4.066320000	-1.418204000
H	-1.066642000	4.831140000	-1.910439000
C	-1.034839000	2.828924000	-1.151439000
H	-2.060398000	2.657703000	-1.455232000
C	-0.321016000	1.793429000	-0.539209000
C	-0.992823000	0.528545000	-0.219616000
C	-0.429741000	-0.731244000	-0.073670000
C	-1.446864000	-1.715698000	0.406419000
C	-2.762049000	-1.237768000	0.544496000
C	-3.802085000	-2.014275000	1.024966000
H	-4.802949000	-1.611105000	1.134605000
C	-3.540852000	-3.330462000	1.382233000
H	-4.335574000	-3.957873000	1.768079000
C	-2.255779000	-3.833198000	1.239589000
H	-2.050051000	-4.862124000	1.512282000
C	-1.220401000	-3.042969000	0.757073000
H	-0.230995000	-3.463057000	0.657528000
C	3.758419000	1.337043000	0.527332000
H	3.744182000	2.323478000	1.002791000
C	4.300639000	1.475540000	-0.892170000
H	4.457731000	0.497696000	-1.346533000
H	5.269008000	1.984475000	-0.862607000
H	3.641904000	2.043457000	-1.550255000
C	4.671715000	0.431780000	1.348665000
H	4.419840000	0.435016000	2.409888000
H	5.706621000	0.772978000	1.253663000
H	4.630829000	-0.599929000	0.992122000
C	1.447671000	0.895993000	2.319480000
H	0.364985000	0.976952000	2.172926000
C	1.682698000	-0.306305000	3.222407000
H	1.188717000	-1.197575000	2.840086000
H	1.275814000	-0.097552000	4.217140000
H	2.741349000	-0.540692000	3.346185000
C	1.930160000	2.186337000	2.969251000
H	2.997261000	2.147762000	3.201861000
H	1.402369000	2.343365000	3.915151000
H	1.748562000	3.060999000	2.340928000
C	-3.531899000	1.484254000	1.319237000
H	-4.462383000	0.942099000	1.533011000
C	-2.662324000	1.463144000	2.571141000
H	-2.396479000	0.447479000	2.870009000
H	-3.201776000	1.930127000	3.398847000
H	-1.740357000	2.025367000	2.410128000
C	-3.885236000	2.905314000	0.898018000
H	-2.990255000	3.492406000	0.687415000
H	-4.420267000	3.396209000	1.715009000
H	-4.532446000	2.935770000	0.019301000
C	-3.755475000	0.604784000	-1.529125000
H	-3.765323000	1.673377000	-1.766912000
C	-3.072320000	-0.147183000	-2.665450000
H	-2.066915000	0.227946000	-2.859654000
H	-3.662954000	-0.036769000	-3.578179000
H	-2.995362000	-1.214009000	-2.443232000
C	-5.196272000	0.147395000	-1.327620000
H	-5.243756000	-0.924989000	-1.130224000
H	-5.763645000	0.338415000	-2.241857000
H	-5.700415000	0.673800000	-0.513855000
C	3.204331000	-1.736663000	-1.211316000
C	1.380783000	-0.265134000	-2.207514000
C	1.921448000	-2.446368000	0.766794000
C	0.805251000	-2.679380000	-1.589993000

7

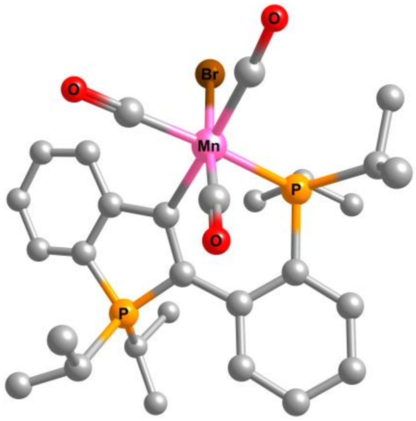

Mn	1.410904000	-0.899086000	-0.799133000
Br	2.278631000	-2.310858000	1.189320000
P	1.932771000	1.021854000	0.381342000
P	-2.943146000	0.291289000	0.008864000
O	4.091814000	-1.513348000	-1.881867000
O	0.555240000	-3.298161000	-2.281507000
O	0.714630000	0.662238000	-3.159266000
C	3.077919000	-1.241090000	-1.427777000
C	0.685004000	2.310080000	-0.017276000
C	1.052371000	3.650597000	-0.107652000
H	2.073994000	3.936771000	0.103748000
C	0.162386000	4.648319000	-0.473243000
H	0.494244000	5.677695000	-0.539101000
C	-1.144252000	4.302086000	-0.763194000
H	-1.861469000	5.054223000	-1.072191000
C	-1.532994000	2.979803000	-0.659524000
H	-2.560888000	2.736950000	-0.899060000
C	-0.658514000	1.953767000	-0.282646000
C	-1.183982000	0.593899000	-0.118195000
C	-0.503244000	-0.606899000	-0.095808000
C	-1.403024000	-1.736432000	0.281732000
C	-1.015989000	-3.038951000	0.579761000
H	0.034353000	-3.301092000	0.564363000
C	-1.967475000	-3.981202000	0.951313000
H	-1.643643000	-4.988164000	1.189275000
C	-3.313912000	-3.658151000	1.031474000
H	-4.040676000	-4.406365000	1.325346000
C	-3.726215000	-2.363176000	0.743282000
H	-4.775385000	-2.098234000	0.817081000
C	-2.770769000	-1.431249000	0.379476000
C	1.969358000	0.988445000	2.243714000
H	2.726198000	0.223555000	2.447098000
C	0.659272000	0.494681000	2.836009000
H	-0.155536000	1.190353000	2.623185000
H	0.760483000	0.422078000	3.923092000
H	0.400037000	-0.492226000	2.454268000
C	2.377183000	2.308108000	2.885827000
H	3.376516000	2.636813000	2.597230000
H	2.377937000	2.191525000	3.973720000
H	1.670864000	3.107676000	2.645219000
C	3.554734000	1.838880000	-0.019104000
H	3.564263000	2.803576000	0.496135000
C	4.717963000	1.020585000	0.536167000
H	4.716122000	0.987562000	1.626617000
H	5.661496000	1.474267000	0.219372000
H	4.707482000	-0.008562000	0.177338000
C	3.717350000	2.097475000	-1.511851000
H	3.805147000	1.166759000	-2.072401000
H	4.633032000	2.670513000	-1.684816000
H	2.883101000	2.662636000	-1.931506000
C	-3.850512000	0.485911000	-1.584490000
H	-3.971419000	1.564353000	-1.728071000
C	-5.234959000	-0.151514000	-1.526132000
H	-5.163166000	-1.235458000	-1.419847000
H	-5.761191000	0.052745000	-2.461742000
H	-5.851046000	0.237811000	-0.712574000
C	-3.018800000	-0.070304000	-2.734158000
H	-2.063420000	0.443900000	-2.839208000
H	-3.574784000	0.046911000	-3.667606000
H	-2.817640000	-1.135409000	-2.597286000
C	-3.946271000	1.051550000	1.350830000
H	-4.785671000	0.347335000	1.424646000
C	-4.509671000	2.437353000	1.063337000
H	-3.722214000	3.191584000	1.044234000
H	-5.205941000	2.708181000	1.861109000
H	-5.059121000	2.485728000	0.121037000
C	-3.173221000	1.015735000	2.663492000
H	-2.754688000	0.029594000	2.870837000
H	-3.841009000	1.281401000	3.486628000
H	-2.354214000	1.736452000	2.648827000
C	0.869839000	-2.387457000	-1.664506000
C	0.996312000	0.064284000	-2.216670000

[8]$^+$

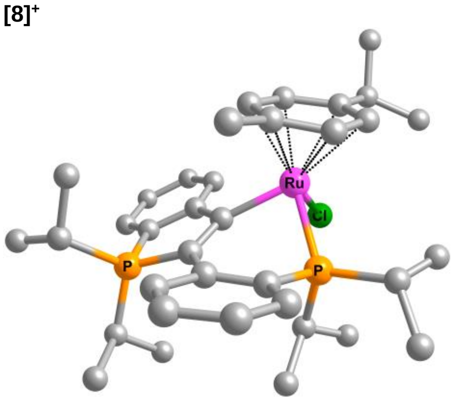

Ru	-1.413233000	-0.354190000	0.269843000
Cl	-1.916229000	-0.986573000	-1.975427000
P	-0.919150000	1.751938000	-0.557153000
P	3.186714000	-0.581830000	-0.067899000
C	0.338377000	2.354210000	0.617770000
C	0.196681000	3.541341000	1.329500000
H	-0.686704000	4.149844000	1.195543000
C	1.167096000	3.983865000	2.217072000
H	1.027229000	4.914152000	2.754373000
C	2.316116000	3.233924000	2.389111000
H	3.096428000	3.567800000	3.062845000
C	2.454233000	2.032962000	1.714424000
H	3.341898000	1.443738000	1.902922000
C	1.474172000	1.544785000	0.846669000
C	1.607999000	0.208244000	0.251381000
C	0.581156000	-0.656773000	-0.068622000
C	1.078242000	-1.959014000	-0.598740000
C	2.474804000	-2.079079000	-0.695918000
C	3.094272000	-3.205298000	-1.208742000
H	4.172103000	-3.263199000	-1.303605000
C	2.304366000	-4.271855000	-1.618959000
H	2.766242000	-5.162485000	-2.027553000
C	0.925324000	-4.186246000	-1.506665000
H	0.310529000	-5.017591000	-1.831931000
C	0.311076000	-3.043803000	-1.007621000
H	-0.765957000	-2.974549000	-0.980393000
C	-0.091417000	1.883826000	-2.226723000
H	0.439315000	0.930039000	-2.293233000
C	0.914814000	3.027188000	-2.319917000
H	0.429319000	4.001666000	-2.253821000
H	1.411068000	2.984532000	-3.294013000
H	1.681772000	2.987045000	-1.545786000
C	-1.058227000	1.941199000	-3.407768000
H	-1.812716000	1.159490000	-3.362622000
H	-0.485188000	1.796983000	-4.327820000
H	-1.545557000	2.913668000	-3.487329000
C	-2.305180000	2.990502000	-0.503809000
H	-2.506047000	3.060800000	0.572353000
C	-2.010574000	4.396181000	-1.028892000
H	-1.084212000	4.829733000	-0.655286000
H	-2.829520000	5.060304000	-0.738532000
H	-1.965471000	4.407309000	-2.117737000
C	-3.569450000	2.452165000	-1.173223000
H	-3.497199000	2.511944000	-2.258912000
H	-4.427501000	3.056323000	-0.867165000
H	-3.764341000	1.412247000	-0.917200000
C	4.224346000	-0.958322000	1.408100000
H	4.659004000	0.000196000	1.708534000
C	3.363971000	-1.496819000	2.543239000
H	2.594979000	-0.785112000	2.843959000
H	3.994966000	-1.700077000	3.411152000
H	2.878327000	-2.432949000	2.258018000
C	5.372002000	-1.909313000	1.078902000
H	5.003261000	-2.902123000	0.817186000
H	5.999653000	-2.018660000	1.966180000
H	6.012867000	-1.547352000	0.273378000
C	4.262944000	0.186832000	-1.334320000
H	5.056364000	-0.561051000	-1.459950000
C	4.893375000	1.496412000	-0.872351000
H	4.138680000	2.267363000	-0.707773000
H	5.571783000	1.856733000	-1.648744000
H	5.477139000	1.386058000	0.043391000
C	3.532011000	0.335045000	-2.662817000
H	3.095217000	-0.604850000	-3.002567000
H	4.240466000	0.671795000	-3.422868000
H	2.739674000	1.080035000	-2.594313000
C	-3.337134000	-1.527804000	0.896673000
C	-3.481530000	-0.190959000	1.280818000
H	-4.349826000	0.373394000	0.973805000
C	-2.453451000	0.451362000	2.017661000
H	-2.566062000	1.497401000	2.279189000
C	-1.325740000	-0.244918000	2.506970000
C	-1.193133000	-1.594870000	2.092999000
H	-0.304297000	-2.146384000	2.378034000
C	-2.165042000	-2.225044000	1.304807000
H	-2.024407000	-3.253268000	0.998713000
C	-4.371565000	-2.269097000	0.090482000
H	-3.815814000	-2.847971000	-0.655331000
C	-5.347646000	-1.374476000	-0.653796000
H	-5.981536000	-0.804056000	0.031275000
H	-6.011024000	-1.988175000	-1.265776000
H	-4.824062000	-0.686144000	-1.319056000
C	-5.110003000	-3.245887000	1.010165000
H	-4.427064000	-3.929240000	1.519310000
H	-5.812439000	-3.844894000	0.427473000
H	-5.679261000	-2.708378000	1.773079000
C	-0.372966000	0.385015000	3.466917000
H	0.625435000	-0.040599000	3.374646000
H	-0.715527000	0.207300000	4.491139000
H	-0.303208000	1.462794000	3.319135000

[9]$^+$

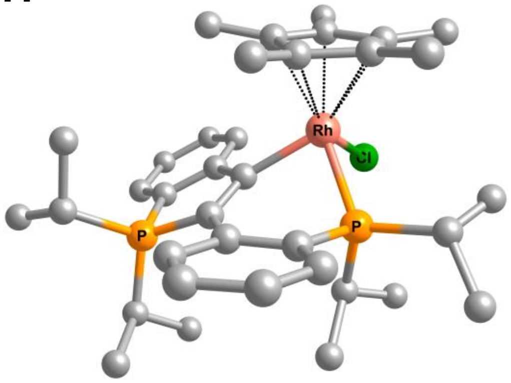

Rh	-1.361610000	-0.748945000	0.035580000
Cl	-1.813632000	-1.441115000	-2.204545000
P	3.149212000	0.161755000	0.027306000
P	-1.371035000	1.437163000	-0.648280000
C	1.403682000	0.515890000	0.280001000
C	0.902997000	1.736971000	0.927121000
C	1.668295000	2.390736000	1.895114000
H	2.653781000	2.016595000	2.139184000
C	1.195250000	3.487069000	2.595982000
H	1.817834000	3.956556000	3.348422000
C	-0.080242000	3.958793000	2.345113000
H	-0.475942000	4.803614000	2.895879000
C	-0.848275000	3.347733000	1.364651000
H	-1.836941000	3.740334000	1.170921000
C	-0.375285000	2.262407000	0.633517000
C	0.651950000	-0.541467000	-0.176852000
C	1.477011000	-1.631940000	-0.756930000
C	2.865981000	-1.417512000	-0.730728000
C	3.766649000	-2.322675000	-1.263813000
H	4.832626000	-2.128287000	-1.261294000
C	3.278081000	-3.496392000	-1.825358000
H	3.964570000	-4.218481000	-2.250632000
C	1.911935000	-3.732636000	-1.847044000
H	1.534672000	-4.644468000	-2.295630000
C	1.011957000	-2.811828000	-1.323128000
H	-0.052634000	-2.984572000	-1.393451000
C	4.177868000	-0.015292000	1.544920000
H	4.342457000	1.008151000	1.896794000
C	3.439478000	-0.802856000	2.618060000
H	2.489081000	-0.340343000	2.887328000
H	4.056756000	-0.854289000	3.517590000
H	3.248227000	-1.826251000	2.288279000
C	5.541002000	-0.633026000	1.243129000
H	5.444019000	-1.671476000	0.922765000
H	6.135676000	-0.629383000	2.159350000
H	6.106398000	-0.082548000	0.489705000
C	4.053374000	1.256734000	-1.130479000
H	5.023353000	0.752696000	-1.228071000
C	3.390768000	1.284523000	-2.501927000
H	3.239408000	0.284531000	-2.910036000
H	4.029851000	1.837619000	-3.193740000
H	2.427692000	1.792684000	-2.462801000
C	4.282893000	2.657492000	-0.572338000
H	3.341159000	3.189742000	-0.428059000
H	4.881192000	3.229026000	-1.285256000
H	4.823885000	2.653778000	0.375625000
C	-3.021299000	2.279647000	-0.653614000
H	-3.279586000	2.276830000	0.410462000
C	-4.071212000	1.441156000	-1.379808000
H	-3.935598000	1.478792000	-2.460513000
H	-5.065519000	1.838315000	-1.157917000
H	-4.037103000	0.393051000	-1.086784000
C	-3.067824000	3.724537000	-1.151065000
H	-2.304485000	4.369635000	-0.717530000
H	-4.043325000	4.150129000	-0.899501000
H	-2.970248000	3.775438000	-2.235219000
C	-0.501640000	1.831366000	-2.253197000
H	0.266116000	1.054097000	-2.297763000
C	-1.372667000	1.672629000	-3.497430000
H	-1.880109000	0.711041000	-3.524346000
H	-0.726910000	1.735178000	-4.377656000
H	-2.107102000	2.473840000	-3.585499000
C	0.172141000	3.200836000	-2.252923000
H	-0.556760000	4.011078000	-2.214840000
H	0.731697000	3.320853000	-3.185246000
H	0.863821000	3.337928000	-1.420901000
C	-1.583208000	-0.907743000	2.196463000
C	-1.143787000	-2.203206000	1.732293000
C	-2.130264000	-2.709306000	0.864877000
C	-3.236563000	-1.769756000	0.834882000
C	-2.920749000	-0.701554000	1.695241000
C	-0.941169000	-0.118630000	3.283235000
H	0.142859000	-0.230116000	3.270080000
H	-1.295036000	-0.460288000	4.262175000
H	-1.165066000	0.945384000	3.202938000
C	0.083549000	-2.907200000	2.189212000
H	0.412806000	-3.660828000	1.474799000
H	-0.113218000	-3.412024000	3.140694000
H	0.906953000	-2.212802000	2.352214000
C	-2.147826000	-4.021483000	0.169266000
H	-2.379032000	-3.896040000	-0.891630000
H	-2.922389000	-4.662406000	0.602178000
H	-1.196939000	-4.545523000	0.260353000
C	-4.513076000	-2.024571000	0.116170000
H	-5.206864000	-1.189415000	0.206977000
H	-5.007886000	-2.909850000	0.527535000
H	-4.331778000	-2.209900000	-0.945623000
C	-3.840969000	0.373023000	2.155092000
H	-3.304506000	1.285105000	2.419393000
H	-4.360532000	0.036934000	3.058873000
H	-4.602681000	0.613230000	1.413967000

[10]$^{+}$

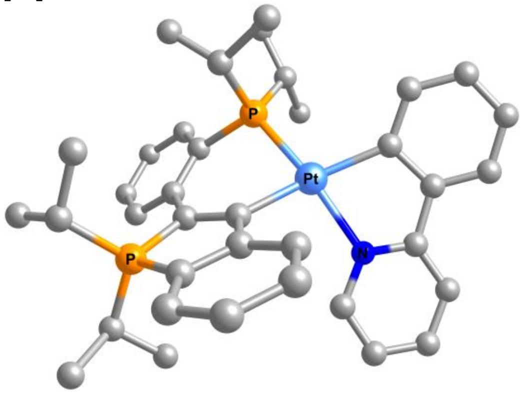

Pt	1.195898000	-0.158925000	0.057313000
P	0.657954000	2.005537000	0.214499000
P	-3.420088000	-0.593424000	0.065553000
C	3.246075000	-0.141520000	0.312115000
N	1.720742000	-2.050430000	-0.733873000
C	-0.692293000	2.315634000	-0.988425000
C	-0.686521000	3.487203000	-1.740682000
H	0.104863000	4.206815000	-1.599640000
C	-1.670033000	3.772992000	-2.675028000
H	-1.625453000	4.693880000	-3.243743000
C	-2.697460000	2.869103000	-2.871503000
H	-3.469860000	3.062216000	-3.606684000
C	-2.731311000	1.707981000	-2.120133000
H	-3.538195000	1.006544000	-2.293490000
C	-1.752329000	1.401448000	-1.172419000
C	-1.861794000	0.178385000	-0.373408000
C	-0.825388000	-0.517175000	0.184122000
C	-1.286857000	-1.617418000	1.068998000
C	-0.477203000	-2.424529000	1.859560000
H	0.597505000	-2.287881000	1.841941000
C	-1.049845000	-3.389895000	2.679733000
H	-0.410893000	-4.010917000	3.296877000
C	-2.425342000	-3.563486000	2.728809000
H	-2.856309000	-4.314638000	3.379552000
C	-3.254606000	-2.763204000	1.950178000
H	-4.330130000	-2.886738000	2.005325000
C	-2.677246000	-1.808358000	1.132556000
C	2.077642000	3.108082000	-0.234169000
H	2.848326000	2.670543000	0.404106000
C	2.034236000	4.593932000	0.114898000
H	1.252911000	5.156204000	-0.396755000
H	2.989671000	5.039609000	-0.175249000
H	1.915596000	4.762276000	1.185139000
C	2.543259000	2.849806000	-1.666234000
H	2.611326000	1.780147000	-1.872508000
H	3.539725000	3.278320000	-1.797771000
H	1.887390000	3.300636000	-2.411666000
C	-0.049166000	2.512827000	1.849622000
H	-0.773321000	1.706681000	2.016482000
C	-0.804813000	3.835171000	1.876636000
H	-0.141366000	4.694255000	1.779078000
H	-1.321632000	3.934319000	2.835400000
H	-1.553259000	3.899474000	1.084697000
C	1.002678000	2.416313000	2.949423000
H	1.493664000	1.441138000	2.951609000
H	0.530636000	2.559076000	3.924712000
H	1.770216000	3.187145000	2.847492000
C	-4.612648000	0.412269000	1.026701000
H	-5.268918000	-0.346889000	1.471854000
C	-5.456987000	1.353329000	0.174821000
H	-4.844511000	2.122093000	-0.298969000
H	-6.181370000	1.857444000	0.818454000
H	-6.020001000	0.831594000	-0.601060000
C	-3.892334000	1.148871000	2.150518000
H	-3.282300000	0.479541000	2.759817000
H	-4.631039000	1.617538000	2.804225000
H	-3.251779000	1.937433000	1.751407000
C	-4.297716000	-1.412876000	-1.324880000
H	-4.726850000	-0.595643000	-1.913518000
C	-3.314512000	-2.187729000	-2.193783000
H	-2.520217000	-1.551362000	-2.586650000
H	-3.849377000	-2.625760000	-3.039249000
H	-2.858193000	-3.005769000	-1.631138000
C	-5.438695000	-2.296772000	-0.829486000
H	-5.061704000	-3.148190000	-0.259917000
H	-5.978471000	-2.694718000	-1.691586000
H	-6.161183000	-1.756414000	-0.215012000
C	4.038114000	0.685238000	1.111194000
H	3.594440000	1.518578000	1.644543000
C	5.397680000	0.467812000	1.295994000
H	5.968598000	1.140313000	1.927367000
C	6.026085000	-0.607076000	0.685226000
H	7.087805000	-0.775268000	0.818948000
C	5.268616000	-1.485953000	-0.065002000
H	5.746832000	-2.359484000	-0.493498000
C	3.900365000	-1.270089000	-0.232240000
C	3.048970000	-2.246866000	-0.897649000
C	3.503739000	-3.330041000	-1.648753000
H	4.566233000	-3.471585000	-1.790631000
C	2.603581000	-4.201835000	-2.223068000
H	2.955790000	-5.041920000	-2.810184000
C	1.244244000	-3.982033000	-2.047519000
H	0.500462000	-4.637403000	-2.480984000
C	0.852376000	-2.891961000	-1.301806000
H	-0.194295000	-2.671221000	-1.142734000

[11]⁺

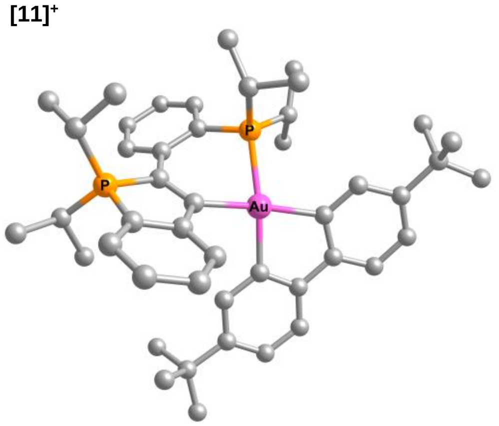

Au	-0.732799000	0.261366000	-0.032885000
P	-1.004189000	-2.087884000	-0.414371000
P	3.660897000	-1.185887000	0.603181000
C	-2.699020000	0.923189000	-0.021955000
C	0.395603000	-2.545186000	-1.502703000
C	0.170974000	-3.438774000	-2.547748000
H	-0.808640000	-3.873131000	-2.674107000
C	1.165445000	-3.797266000	-3.443985000
H	0.946519000	-4.491221000	-4.246391000
C	2.427433000	-3.251534000	-3.304074000
H	3.216998000	-3.498269000	-4.003992000
C	2.677152000	-2.381667000	-2.257501000
H	3.668350000	-1.955656000	-2.167829000
C	1.693757000	-2.013180000	-1.337442000
C	2.053522000	-1.132759000	-0.216881000
C	1.267590000	-0.213497000	0.402238000
C	1.893253000	0.399169000	1.594501000
C	1.298053000	1.306562000	2.461609000
H	0.296554000	1.666222000	2.261189000
C	1.997858000	1.759025000	3.574268000
H	1.525714000	2.465852000	4.246526000
C	3.286522000	1.319492000	3.838668000
H	3.814754000	1.677472000	4.714005000
C	3.902427000	0.414193000	2.981019000
H	4.905278000	0.063731000	3.196033000
C	3.202606000	-0.029513000	1.873492000
C	-2.581670000	-2.448245000	-1.314126000
H	-3.283782000	-1.916355000	-0.666951000
C	-3.094334000	-3.883054000	-1.409069000
H	-2.459742000	-4.551680000	-1.990964000
H	-4.070468000	-3.862010000	-1.901144000
H	-3.241159000	-4.334423000	-0.427978000
C	-2.627165000	-1.710013000	-2.651093000
H	-2.328790000	-0.665352000	-2.543959000
H	-3.654626000	-1.719396000	-3.022064000
H	-1.995029000	-2.171276000	-3.410324000
C	-0.841942000	-3.151865000	1.089583000
H	0.050546000	-2.719583000	1.558075000
C	-0.572731000	-4.629004000	0.833112000
H	-1.444575000	-5.142535000	0.428086000
H	-0.318931000	-5.118342000	1.777590000
H	0.257532000	-4.783623000	0.141479000
C	-2.014003000	-2.925219000	2.038959000
H	-2.154296000	-1.866784000	2.268287000
H	-1.830243000	-3.449132000	2.980015000
H	-2.949664000	-3.314025000	1.630618000
C	4.140257000	-2.783842000	1.358501000
H	4.931738000	-2.488621000	2.059701000
C	4.713877000	-3.785614000	0.362457000
H	3.970548000	-4.084810000	-0.378453000
H	5.022891000	-4.682950000	0.903141000
H	5.591453000	-3.404528000	-0.162318000
C	2.981944000	-3.368716000	2.157485000
H	2.553463000	-2.646958000	2.855292000
H	3.340130000	-4.222585000	2.736419000
H	2.193122000	-3.725588000	1.493223000
C	5.067547000	-0.543694000	-0.386151000
H	5.282707000	-1.330955000	-1.116040000
C	4.670560000	0.730029000	-1.120622000
H	3.805220000	0.584023000	-1.768803000
H	5.505540000	1.063589000	-1.740395000
H	4.440564000	1.533317000	-0.417591000
C	6.310041000	-0.339409000	0.476506000
H	6.158193000	0.457347000	1.206699000
H	7.138539000	-0.036853000	-0.167555000
H	6.620613000	-1.244542000	1.001817000
C	-3.809985000	0.223159000	0.418977000
H	-3.702692000	-0.800216000	0.759498000
C	-5.088499000	0.795288000	0.490676000
C	-5.214359000	2.121687000	0.099952000
H	-6.180661000	2.608419000	0.120501000
C	-4.105319000	2.863266000	-0.283135000
H	-4.233333000	3.913774000	-0.520906000
C	-2.843678000	2.289246000	-0.323218000
C	-1.598858000	3.013062000	-0.556540000
C	-1.494830000	4.342949000	-0.936995000
H	-2.389885000	4.928382000	-1.117192000
C	-0.254287000	4.942429000	-1.100690000
H	-0.219823000	5.983342000	-1.394726000
C	0.923483000	4.233420000	-0.900249000
C	0.809312000	2.888085000	-0.521592000
H	1.716026000	2.314048000	-0.369135000
C	-0.419961000	2.281600000	-0.337176000
C	2.303437000	4.857737000	-1.090522000
C	-6.265813000	-0.035603000	0.994896000
C	3.019442000	4.151775000	-2.247958000
H	3.119280000	3.080030000	-2.064405000
H	2.466165000	4.277894000	-3.181467000
H	4.021266000	4.568203000	-2.387381000
C	2.225385000	6.346775000	-1.418067000
H	1.728688000	6.911316000	-0.625316000
H	3.234812000	6.749930000	-1.528758000
H	1.695109000	6.530360000	-2.355321000
C	-5.990839000	-0.486270000	2.434557000
H	-5.085250000	-1.093686000	2.504386000
H	-5.868007000	0.374692000	3.095696000
H	-6.824327000	-1.086772000	2.809142000
C	-6.448364000	-1.270864000	0.105025000
H	-6.634361000	-0.982028000	-0.932306000
H	-5.567852000	-1.918198000	0.124038000
H	-7.299344000	-1.864068000	0.450511000
C	-7.573203000	0.752368000	0.983407000
H	-7.840795000	1.081547000	-0.023588000
H	-8.384652000	0.119465000	1.349953000
H	-7.523257000	1.631017000	1.630675000
C	3.125373000	4.698695000	0.193763000
H	2.643760000	5.211717000	1.029674000
H	3.246620000	3.649890000	0.475085000
H	4.122041000	5.128747000	0.060844000

[12]$^{+}$

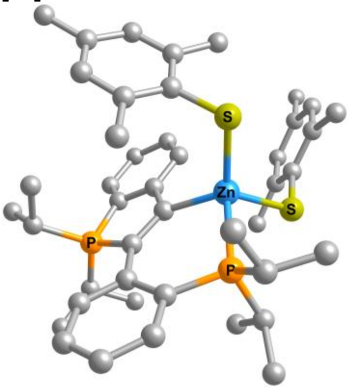

Zn	0.475257000	-1.177786000	0.591638000
S	1.838029000	-2.555801000	-0.740970000
S	1.239840000	-0.684347000	2.731811000
P	-2.117042000	1.980852000	-1.610660000
P	-1.463997000	-2.665298000	0.514874000
C	-1.868752000	0.472123000	-0.618611000
C	-0.530584000	0.331556000	-0.392489000
C	0.291281000	1.389849000	-0.999514000
C	1.675404000	1.471326000	-0.915258000
H	2.227271000	0.728644000	-0.351628000
C	2.349338000	2.486886000	-1.580249000
H	3.430343000	2.530219000	-1.519445000
C	1.660964000	3.424074000	-2.338283000
H	2.202715000	4.200590000	-2.865394000
C	0.274128000	3.368121000	-2.427116000
H	-0.258303000	4.096774000	-3.027897000
C	-0.390208000	2.363369000	-1.746429000
C	-3.020802000	-0.331565000	-0.154183000
C	-2.986341000	-1.653219000	0.368690000
C	-4.179690000	-2.244291000	0.786183000
H	-4.148518000	-3.248050000	1.190445000
C	-5.407293000	-1.610032000	0.711247000
H	-6.305693000	-2.111359000	1.051125000
C	-5.454298000	-0.330353000	0.192434000
H	-6.393821000	0.205307000	0.116528000
C	-4.283754000	0.273931000	-0.223689000
H	-4.361392000	1.281554000	-0.607565000
C	-2.783476000	1.779683000	-3.311747000
H	-2.477358000	2.717138000	-3.794846000
C	-2.074555000	0.624038000	-4.009735000
H	-0.988547000	0.720598000	-3.967226000
H	-2.371253000	0.600161000	-5.061035000
H	-2.346085000	-0.332105000	-3.559896000
C	-4.298947000	1.658796000	-3.405193000
H	-4.653216000	0.725892000	-2.964333000
H	-4.589924000	1.658612000	-4.458567000
H	-4.820165000	2.488718000	-2.923319000
C	-2.976294000	3.386770000	-0.782870000
H	-4.045706000	3.162362000	-0.843272000
C	-2.561718000	3.450677000	0.681938000
H	-2.821490000	2.539617000	1.221968000
H	-3.068825000	4.290505000	1.163499000
H	-1.485783000	3.603493000	0.789489000
C	-2.733996000	4.710444000	-1.500672000
H	-1.691801000	5.020341000	-1.413327000
H	-3.347158000	5.483356000	-1.030778000
H	-3.003106000	4.678558000	-2.559031000
C	-1.519769000	-3.774366000	-0.978342000
H	-0.549334000	-4.281780000	-0.932397000
C	-1.534640000	-2.937749000	-2.250511000
H	-2.475834000	-2.387787000	-2.343266000
H	-1.444849000	-3.593058000	-3.121486000
H	-0.698499000	-2.236756000	-2.270424000
C	-2.633980000	-4.809363000	-0.986683000
H	-2.598460000	-5.476205000	-0.123150000
H	-2.544815000	-5.431905000	-1.882154000
H	-3.619664000	-4.337145000	-1.016915000
C	-1.720113000	-3.788298000	1.961061000
H	-2.609494000	-4.405515000	1.796432000
C	-0.505808000	-4.710559000	2.054213000
H	-0.395695000	-5.350045000	1.176094000
H	-0.607118000	-5.358965000	2.928775000
H	0.417125000	-4.136239000	2.166292000
C	-1.889860000	-2.984637000	3.244442000
H	-1.018766000	-2.347333000	3.420457000
H	-1.990862000	-3.667598000	4.092953000
H	-2.776050000	-2.347347000	3.219181000
C	3.324292000	-1.695665000	-1.155805000
C	4.411123000	-1.665157000	-0.259970000
C	5.575784000	-1.003184000	-0.630052000
H	6.405398000	-0.984163000	0.072169000
C	5.718347000	-0.379936000	-1.864728000
C	4.646949000	-0.442315000	-2.743490000
H	4.735206000	0.023193000	-3.722039000
C	3.458014000	-1.089673000	-2.416849000
C	4.338368000	-2.348556000	1.068286000
H	3.559403000	-1.911494000	1.699408000
H	5.294522000	-2.278148000	1.590999000
H	4.077747000	-3.402869000	0.943079000
C	6.999432000	0.303768000	-2.240642000
H	7.339956000	0.979676000	-1.451425000
H	6.884259000	0.885649000	-3.157381000
H	7.802226000	-0.421074000	-2.409360000
C	2.348587000	-1.136938000	-3.420291000
H	2.083261000	-2.169522000	-3.662264000
H	2.635109000	-0.619940000	-4.338866000
H	1.438828000	-0.676764000	-3.023616000
C	1.095121000	1.067399000	2.929948000
C	-0.129195000	1.661225000	3.277381000
C	-0.191354000	3.037751000	3.478404000
H	-1.144515000	3.480562000	3.757949000
C	0.922231000	3.855247000	3.352568000
C	2.129035000	3.249328000	3.024541000
H	3.022617000	3.861955000	2.934038000
C	2.241058000	1.879298000	2.820328000
C	-1.361718000	0.835081000	3.463530000
H	-1.630279000	0.304854000	2.545333000
H	-2.205701000	1.457218000	3.770805000
H	-1.199636000	0.063362000	4.220507000
C	0.840379000	5.333177000	3.595955000
H	1.303002000	5.900281000	2.783226000
H	1.359221000	5.614582000	4.517771000
H	-0.196491000	5.662772000	3.689672000
C	3.583232000	1.290912000	2.517573000
H	3.869781000	0.562895000	3.281167000
H	4.345968000	2.071002000	2.472241000
H	3.586684000	0.741538000	1.572963000

Mechanism for the Conversion of 13 to 14

DFT calculations were carried out with the Gaussian 16 program suite (G16RevB.01).[13] All structures were optimised without symmetry restrictions and identified as minima or transition states by analytical frequency analyses. The complexes were modeled using the PBE0 (PBE1PBE) functional[14] on the Def2-TZVP[15] basis set. All relevant structures were re-optimised with dispersion (GD3)[16] and solvent correction (PCM for thf or MeCN).[17,18]

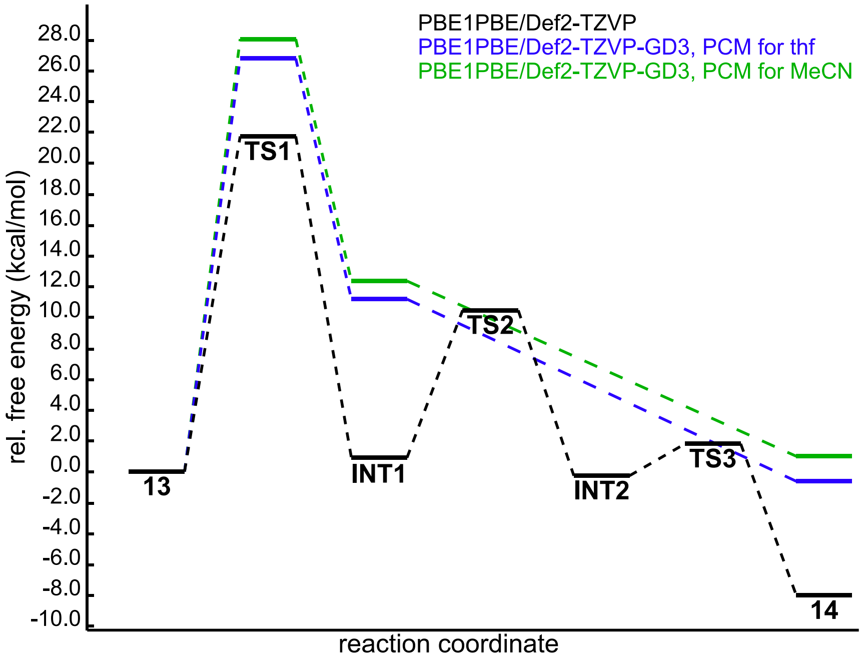

Figure S66: Calculated mechanism for the conversion of **13** to **14** in the gas phase and calculated mechanisms in solution (employing dispersion and solvent correction during optimisation of the structures in solution). Compound **13** was set to 0 kcal/mol in each case.

13 (PBE1PBE/Def2-TZVP)

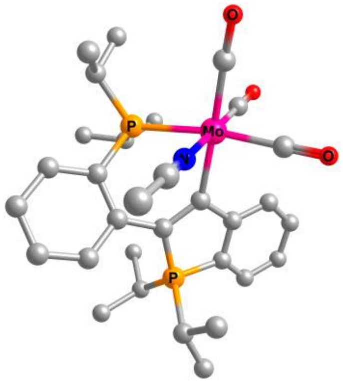

Mo	-1.549052000	-1.228341000	-0.524181000
P	-1.844152000	0.822832000	0.887432000
P	2.908728000	0.378036000	0.068453000
O	-0.729970000	-3.688303000	-2.278252000
O	-4.546394000	-1.829948000	-1.285292000
O	-2.098681000	-3.352576000	1.684124000
N	-1.357831000	0.098474000	-2.237270000
C	-0.802985000	2.133515000	0.120620000
C	-1.280523000	3.429396000	-0.054724000
H	-2.267301000	3.684131000	0.309496000
C	-0.541066000	4.424155000	-0.679083000
H	-0.954848000	5.419006000	-0.796841000
C	0.725294000	4.119591000	-1.144369000
H	1.326266000	4.869102000	-1.647934000
C	1.226459000	2.842471000	-0.961294000
H	2.215572000	2.626030000	-1.346188000
C	0.505526000	1.820491000	-0.328978000
C	1.145986000	0.515169000	-0.115382000
C	0.554644000	-0.745176000	-0.037186000
C	1.560589000	-1.780837000	0.334110000
C	1.307616000	-3.128073000	0.577137000
H	0.301367000	-3.504884000	0.466545000
C	2.332949000	-3.977271000	0.969802000
H	2.108654000	-5.020540000	1.161584000
C	3.632835000	-3.514892000	1.129668000
H	4.418826000	-4.189148000	1.449120000
C	3.919796000	-2.180716000	0.877365000
H	4.931704000	-1.810614000	1.002286000
C	2.889013000	-1.343325000	0.482270000
C	-1.120341000	0.872890000	2.614212000
H	-0.051133000	0.835110000	2.378255000
C	-1.428204000	-0.348642000	3.468177000
H	-2.485766000	-0.422033000	3.726935000
H	-0.867060000	-0.285983000	4.406284000
H	-1.146213000	-1.271352000	2.964742000
C	-1.398091000	2.169231000	3.362704000
H	-1.159977000	3.052957000	2.766241000
H	-0.792096000	2.209915000	4.273448000
H	-2.443953000	2.238544000	3.672737000
C	-3.525156000	1.604109000	1.041196000
H	-3.415566000	2.570760000	1.544807000
C	-4.429701000	0.720636000	1.895940000
H	-4.113598000	0.685234000	2.938912000
H	-5.451393000	1.110831000	1.872675000
H	-4.455164000	-0.302447000	1.512396000
C	-4.161363000	1.814171000	-0.329038000
H	-3.541863000	2.411832000	-0.999485000
H	-5.123295000	2.323006000	-0.212702000
H	-4.345815000	0.855353000	-0.815068000
C	3.752077000	1.311045000	1.410828000
H	4.683878000	0.747503000	1.551107000
C	2.934938000	1.217224000	2.693794000
H	2.018677000	1.805073000	2.611858000
H	3.517071000	1.615358000	3.528708000
H	2.660559000	0.187843000	2.931736000
C	4.103057000	2.753744000	1.067998000
H	4.725662000	2.834886000	0.174752000
H	4.663099000	3.192920000	1.897729000
H	3.205774000	3.355957000	0.917242000
C	3.898926000	0.601761000	-1.475833000
H	3.922582000	1.681146000	-1.657347000
C	5.335521000	0.114264000	-1.323704000
H	5.862413000	0.596249000	-0.497246000
H	5.890155000	0.336532000	-2.239043000
H	5.369249000	-0.966273000	-1.174029000
C	3.191346000	-0.080849000	-2.639945000
H	3.128063000	-1.159950000	-2.482663000
H	3.752912000	0.092814000	-3.561448000
H	2.175395000	0.293256000	-2.773705000
C	-1.028402000	-2.767309000	-1.647995000
C	-3.451845000	-1.579999000	-1.013381000
C	-1.865464000	-2.527399000	0.901483000
C	-1.289171000	0.779991000	-3.160889000
C	-1.209946000	1.650718000	-4.311223000
H	-2.213002000	1.905032000	-4.660152000
H	-0.672062000	1.159406000	-5.124649000
H	-0.686939000	2.571541000	-4.043214000

TS1 (PBE1PBE/Def2-TZVP)

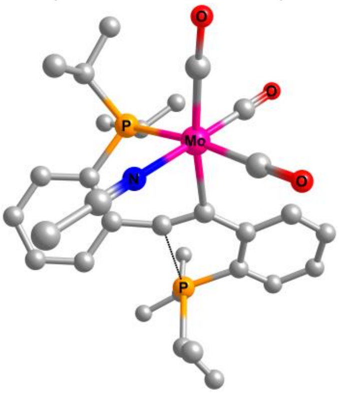

Mo	1.585884000	-1.264427000	0.446117000
P	2.058471000	0.811810000	-0.920979000
P	-3.173332000	0.632491000	-0.062730000
O	0.717389000	-3.644058000	2.278208000
O	4.478973000	-2.343968000	0.827084000
O	1.637017000	-3.278899000	-1.923250000
C	1.104415000	2.138010000	-0.079954000
C	1.582211000	3.427311000	0.123358000
H	2.559050000	3.699449000	-0.257534000
C	0.846932000	4.381194000	0.812671000
H	1.251844000	5.375452000	0.963206000
C	-0.406169000	4.047609000	1.300142000
H	-0.997186000	4.777250000	1.842337000
C	-0.912373000	2.778078000	1.087431000
H	-1.899936000	2.527474000	1.451533000
C	-0.180005000	1.799920000	0.407016000
C	-0.730376000	0.480200000	0.209360000
C	-0.680846000	-0.788256000	0.068072000
C	-1.771628000	-1.694521000	-0.263532000
C	-1.597398000	-3.057484000	-0.480128000
H	-0.610584000	-3.482622000	-0.364302000
C	-2.668562000	-3.855529000	-0.849919000
H	-2.511284000	-4.914449000	-1.019739000
C	-3.929981000	-3.304801000	-1.012679000
H	-4.765197000	-3.927028000	-1.313648000
C	-4.122933000	-1.948818000	-0.784719000
H	-5.113389000	-1.527069000	-0.915748000
C	-3.061694000	-1.138315000	-0.402007000
C	1.345963000	1.015266000	-2.641898000
H	0.272162000	1.007154000	-2.416934000
C	1.616774000	-0.151467000	-3.581957000
H	2.670318000	-0.226783000	-3.856277000
H	1.050764000	-0.006228000	-4.507372000
H	1.316263000	-1.103498000	-3.150073000
C	1.686887000	2.347066000	-3.296312000
H	1.467698000	3.198856000	-2.649093000
H	1.101284000	2.468997000	-4.212554000
H	2.741190000	2.394894000	-3.581529000
C	3.789058000	1.478058000	-1.002129000
H	3.751390000	2.476338000	-1.451825000
C	4.658338000	0.585605000	-1.883270000
H	4.359875000	0.618023000	-2.931369000
H	5.699133000	0.916847000	-1.825416000
H	4.620971000	-0.453952000	-1.548798000
C	4.393042000	1.576697000	0.394484000
H	3.802907000	2.203770000	1.065044000
H	5.399066000	2.002036000	0.329681000
H	4.473845000	0.586586000	0.847129000
C	-4.319363000	1.292713000	-1.363113000
H	-5.234104000	0.688467000	-1.356638000
C	-3.654162000	1.159839000	-2.727947000
H	-2.737517000	1.754311000	-2.773805000
H	-4.326783000	1.521553000	-3.510815000
H	-3.396226000	0.124150000	-2.956718000
C	-4.680470000	2.742601000	-1.064429000
H	-5.227730000	2.851804000	-0.125686000
H	-5.311816000	3.143362000	-1.862390000
H	-3.783512000	3.366100000	-1.007892000
C	-4.126907000	0.743069000	1.537572000
H	-4.169916000	1.822160000	1.734352000
C	-5.549593000	0.204244000	1.500237000
H	-6.159942000	0.676670000	0.727767000
H	-6.038927000	0.386353000	2.462127000
H	-5.554843000	-0.874549000	1.331202000
C	-3.324362000	0.084854000	2.654809000
H	-3.241513000	-0.992555000	2.493174000
H	-3.824433000	0.240598000	3.614848000
H	-2.310797000	0.484032000	2.727478000
C	1.023391000	-2.758381000	1.605569000
C	3.412591000	-1.912642000	0.696032000
C	1.587068000	-2.493678000	-1.072158000
N	1.666227000	-0.004906000	2.216969000
C	1.718036000	0.611440000	3.186330000
C	1.790751000	1.384911000	4.404368000
H	1.345339000	2.369670000	4.248217000
H	1.253626000	0.876010000	5.207328000
H	2.832286000	1.512789000	4.706529000

INT1 (PBE1PBE/Def2-TZVP)

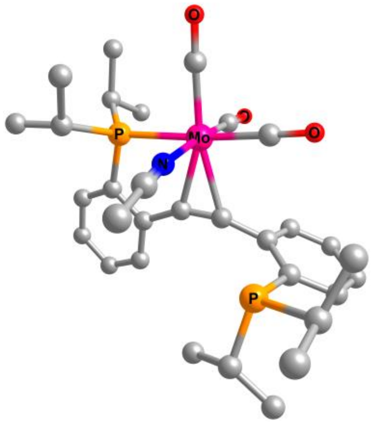

Mo	0.930752000	-1.212871000	-0.252543000
P	-3.379017000	0.315584000	0.867771000
P	2.942821000	0.246764000	0.277238000
O	2.624555000	-3.760635000	0.440848000
O	-1.422361000	-3.209696000	-0.821509000
O	1.687204000	-1.645474000	-3.235808000
C	-3.258596000	0.063746000	-0.949428000
C	-1.997553000	0.095756000	-1.574850000
C	-1.910326000	-0.096146000	-2.961306000
H	-0.931558000	-0.087407000	-3.425749000
C	-3.039710000	-0.315493000	-3.723427000
H	-2.945152000	-0.466507000	-4.792756000
C	-4.285955000	-0.355587000	-3.114253000
H	-5.179482000	-0.537618000	-3.700455000
C	-4.381691000	-0.166489000	-1.747038000
H	-5.361932000	-0.205840000	-1.288097000
C	2.457525000	1.965909000	-0.132348000
C	1.107887000	2.107350000	-0.480381000
C	0.601731000	3.375786000	-0.786505000
H	-0.442444000	3.477946000	-1.055052000
C	1.434232000	4.478775000	-0.771863000
H	1.036791000	5.456750000	-1.019358000
C	2.782431000	4.333724000	-0.464937000
H	3.439182000	5.196067000	-0.475016000
C	3.288779000	3.082338000	-0.151102000
H	4.345993000	2.981473000	0.063813000
C	-0.753706000	0.306780000	-0.878295000
C	0.292436000	0.926937000	-0.572029000
C	-4.838316000	-0.744554000	1.329275000
H	-5.694939000	-0.511604000	0.688737000
C	-5.240803000	-0.465737000	2.772836000
H	-4.404648000	-0.646454000	3.455864000
H	-6.055709000	-1.131201000	3.072720000
H	-5.581637000	0.560978000	2.922699000
C	-4.473433000	-2.212642000	1.144385000
H	-4.200419000	-2.443333000	0.113516000
H	-5.316339000	-2.851935000	1.423974000
H	-3.622931000	-2.485694000	1.775661000
C	-4.066501000	2.054092000	0.990363000
H	-4.187261000	2.189668000	2.072826000
C	-3.005980000	3.040078000	0.516463000
H	-2.046672000	2.875287000	1.011953000
H	-3.323158000	4.068450000	0.714130000
H	-2.847235000	2.943903000	-0.561339000
C	-5.401146000	2.329268000	0.316560000
H	-5.324817000	2.219008000	-0.767668000
H	-5.718783000	3.357507000	0.518879000
H	-6.194814000	1.667660000	0.670555000
C	4.576396000	0.079581000	-0.592108000
H	5.216575000	0.862656000	-0.169181000
C	4.433234000	0.333868000	-2.088096000
H	3.842426000	-0.446463000	-2.568304000
H	5.424648000	0.332143000	-2.550621000
H	3.964316000	1.295043000	-2.304265000
C	5.233276000	-1.271744000	-0.341693000
H	5.459495000	-1.443645000	0.711105000
H	6.175685000	-1.326588000	-0.894278000
H	4.597703000	-2.088967000	-0.687975000
C	3.347290000	0.373986000	2.103309000
H	2.439105000	0.861637000	2.478530000
C	4.540285000	1.246679000	2.467735000
H	5.477452000	0.825174000	2.095320000
H	4.628171000	1.307251000	3.556912000
H	4.445380000	2.265852000	2.093323000
C	3.453172000	-0.986788000	2.784265000
H	2.606930000	-1.629510000	2.546678000
H	3.487123000	-0.847922000	3.869490000
H	4.364616000	-1.515641000	2.500515000
C	2.011328000	-2.812046000	0.212459000
C	-0.573840000	-2.472015000	-0.595711000
C	1.435842000	-1.467073000	-2.120846000
N	0.194902000	-0.865590000	1.786785000
C	-0.311840000	-0.667184000	2.797288000
C	-0.978589000	-0.416562000	4.052518000
H	-0.356249000	0.203048000	4.700861000
H	-1.918875000	0.101077000	3.849759000
H	-1.191859000	-1.358828000	4.560793000

TS2 (PBE1PBE/Def2-TZVP)

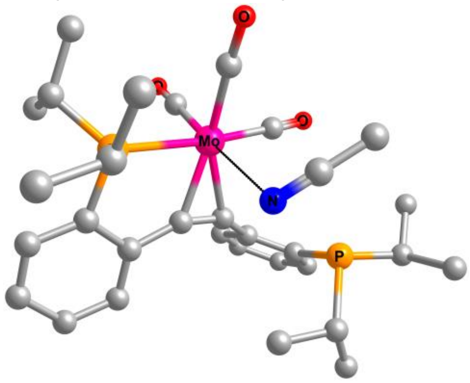

Mo	0.774431000	-0.889032000	-0.721173000
P	-3.360110000	-0.241836000	0.743940000
P	2.959908000	-0.026166000	0.295083000
O	1.796863000	-3.857844000	-0.518071000
O	-1.722110000	-2.150769000	-2.135046000
O	1.833688000	-0.633371000	-3.589712000
C	-3.204069000	0.892670000	-0.692305000
C	-1.922612000	1.242375000	-1.156194000
C	-1.797891000	2.091488000	-2.264171000
H	-0.803638000	2.341938000	-2.615380000
C	-2.910531000	2.587223000	-2.914557000
H	-2.787995000	3.232576000	-3.777007000
C	-4.177439000	2.251678000	-2.459895000
H	-5.060222000	2.635196000	-2.958779000
C	-4.310326000	1.420561000	-1.361398000
H	-5.307995000	1.173599000	-1.019987000
C	2.699506000	1.769343000	0.540044000
C	1.363278000	2.150874000	0.377488000
C	0.983817000	3.476308000	0.604916000
H	-0.054070000	3.762851000	0.482943000
C	1.937618000	4.414337000	0.955650000
H	1.644556000	5.444820000	1.121955000
C	3.273030000	4.045684000	1.075093000
H	4.020041000	4.788330000	1.330993000
C	3.652055000	2.727676000	0.869361000
H	4.696802000	2.454547000	0.962413000
C	-0.680014000	0.786794000	-0.564312000
C	0.447592000	1.141682000	-0.089096000
C	-5.066186000	-0.958159000	0.533082000
H	-5.801870000	-0.159824000	0.391973000
C	-5.450780000	-1.730986000	1.790427000
H	-4.707807000	-2.499835000	2.024550000
H	-6.410162000	-2.235582000	1.642960000
H	-5.549135000	-1.084557000	2.664721000
C	-5.092205000	-1.872616000	-0.686178000
H	-4.824662000	-1.347080000	-1.604221000
H	-6.090620000	-2.300666000	-0.818007000
H	-4.387662000	-2.700881000	-0.570093000
C	-3.587507000	0.942558000	2.175800000
H	-3.761566000	0.265865000	3.021631000
C	-2.284445000	1.685092000	2.444412000
H	-1.438827000	1.002344000	2.544193000
H	-2.368238000	2.269192000	3.365892000
H	-2.060286000	2.382567000	1.633004000
C	-4.756760000	1.908611000	2.071337000
H	-4.633081000	2.584082000	1.220925000
H	-4.816845000	2.524867000	2.974389000
H	-5.716048000	1.398177000	1.960883000
C	4.563589000	-0.156949000	-0.634217000
H	5.349949000	0.114969000	0.080052000
C	4.620250000	0.806452000	-1.815340000
H	3.840868000	0.583091000	-2.545485000
H	5.584034000	0.697797000	-2.321321000
H	4.514369000	1.848667000	-1.514168000
C	4.806912000	-1.584266000	-1.114962000
H	4.784622000	-2.318147000	-0.309805000
H	5.787304000	-1.644752000	-1.596000000
H	4.054660000	-1.873690000	-1.851372000
C	3.309100000	-0.591988000	2.043508000
H	2.348644000	-0.372633000	2.522097000
C	4.406565000	0.162824000	2.780765000
H	5.384797000	0.032489000	2.309110000
H	4.488871000	-0.234065000	3.797069000
H	4.199676000	1.229091000	2.863446000
C	3.538818000	-2.094168000	2.157699000
H	2.793056000	-2.675055000	1.618294000
H	3.484072000	-2.382738000	3.211052000
H	4.529472000	-2.380316000	1.796981000
C	1.462795000	-2.757664000	-0.587581000
C	-0.824713000	-1.694408000	-1.587332000
C	1.461255000	-0.752327000	-2.498671000
N	0.087846000	-1.419984000	2.321652000
C	-0.551952000	-2.324614000	2.010987000
C	-1.352291000	-3.465332000	1.625453000
H	-1.773815000	-3.943027000	2.511387000
H	-2.160491000	-3.118098000	0.978830000
H	-0.735027000	-4.185484000	1.085979000

INT2 (PBE1PBE/Def2-TZVP)

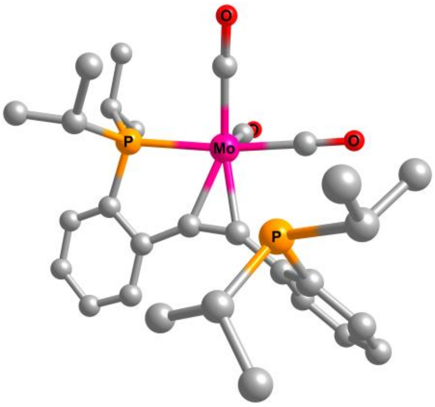

Mo	-0.691381000	1.109528000	-0.585631000
P	2.962240000	0.112413000	1.093329000
P	-2.724574000	0.008369000	0.447076000
O	-1.305370000	3.735376000	1.029199000
O	1.675156000	2.811423000	-1.758410000
O	-2.191846000	2.046720000	-3.107180000
C	3.210137000	-0.464631000	-0.636192000
C	2.072910000	-0.711621000	-1.428236000
C	2.223193000	-1.150520000	-2.747694000
H	1.336384000	-1.326328000	-3.345725000
C	3.479933000	-1.334846000	-3.292245000
H	3.578465000	-1.664083000	-4.320458000
C	4.608118000	-1.090268000	-2.522454000
H	5.598171000	-1.228392000	-2.941876000
C	4.464947000	-0.666386000	-1.211780000
H	5.357038000	-0.481302000	-0.625440000
C	-2.604106000	-1.765648000	0.010354000
C	-1.311434000	-2.102704000	-0.408430000
C	-0.990716000	-3.432644000	-0.679579000
H	0.012736000	-3.688194000	-0.999345000
C	-1.966148000	-4.409337000	-0.564549000
H	-1.723748000	-5.441688000	-0.789826000
C	-3.257560000	-4.070374000	-0.180240000
H	-4.020421000	-4.837360000	-0.110783000
C	-3.576124000	-2.751158000	0.114406000
H	-4.589348000	-2.501072000	0.408286000
C	0.732865000	-0.501715000	-0.924631000
C	-0.398012000	-0.998113000	-0.585528000
C	4.492145000	1.116606000	1.424377000
H	5.388824000	0.544257000	1.164308000
C	4.558239000	1.460006000	2.908785000
H	3.646246000	1.969827000	3.233389000
H	5.399639000	2.131965000	3.101733000
H	4.691823000	0.576492000	3.536594000
C	4.455010000	2.385545000	0.580819000
H	4.415285000	2.170504000	-0.488448000
H	5.345416000	2.992335000	0.771192000
H	3.578625000	2.990726000	0.827643000
C	3.265539000	-1.451624000	2.078794000
H	3.197520000	-1.096351000	3.114483000
C	2.117855000	-2.427764000	1.851086000
H	1.147085000	-1.960526000	2.029034000
H	2.211913000	-3.288436000	2.520073000
H	2.123482000	-2.806114000	0.825155000
C	4.609062000	-2.135792000	1.885230000
H	4.720835000	-2.501013000	0.861333000
H	4.691318000	-3.000144000	2.552548000
H	5.450620000	-1.474677000	2.103229000
C	-4.469504000	0.480858000	0.054797000
H	-5.104623000	-0.174913000	0.661173000
C	-4.788257000	0.241780000	-1.416527000
H	-4.208778000	0.902320000	-2.062061000
H	-5.847259000	0.448832000	-1.595694000
H	-4.591608000	-0.787330000	-1.721803000
C	-4.745168000	1.927632000	0.445436000
H	-4.616962000	2.105985000	1.514494000
H	-5.774767000	2.189757000	0.186795000
H	-4.082096000	2.609186000	-0.092630000
C	-2.628128000	-0.145659000	2.317521000
H	-1.827970000	-0.888778000	2.421008000
C	-3.884305000	-0.710288000	2.966463000
H	-4.713148000	0.000764000	2.925120000
H	-3.686884000	-0.913918000	4.022793000
H	-4.208744000	-1.646709000	2.510266000
C	-2.166792000	1.120692000	3.026548000
H	-1.188491000	1.447150000	2.674528000
H	-2.087896000	0.923538000	4.099759000
H	-2.863884000	1.950618000	2.897082000
C	-1.120586000	2.771722000	0.431021000
C	0.827683000	2.183965000	-1.316945000
C	-1.661687000	1.731119000	-2.128334000

TS3 (PBE1PBE/Def2-TZVP)

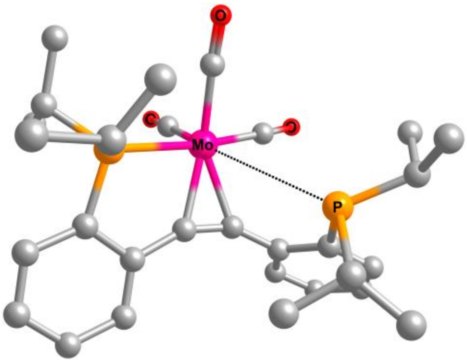

Mo	0.530088000	-0.981962000	-0.778199000
P	-2.633874000	-0.199250000	1.078150000
P	2.575309000	-0.125747000	0.443535000
O	0.982451000	-3.825170000	0.482536000
O	-1.808478000	-2.387925000	-2.332142000
O	2.082566000	-1.661332000	-3.341616000
C	-3.140178000	0.621667000	-0.489773000
C	-2.123815000	1.036135000	-1.373756000
C	-2.454961000	1.659659000	-2.578763000
H	-1.659824000	1.963033000	-3.249860000
C	-3.778753000	1.865861000	-2.922285000
H	-4.022935000	2.338903000	-3.866671000
C	-4.787792000	1.461493000	-2.060634000
H	-5.827544000	1.616945000	-2.325030000
C	-4.463954000	0.853337000	-0.857641000
H	-5.266234000	0.544000000	-0.198178000
C	2.635695000	1.677637000	0.132159000
C	1.422123000	2.162248000	-0.371046000
C	1.251686000	3.531524000	-0.580196000
H	0.308899000	3.900619000	-0.966745000
C	2.295818000	4.401371000	-0.315490000
H	2.169800000	5.463524000	-0.492267000
C	3.508857000	3.918179000	0.159840000
H	4.328278000	4.602453000	0.347780000
C	3.676510000	2.559573000	0.391239000
H	4.630974000	2.196294000	0.755215000
C	-0.742090000	0.796790000	-1.039530000
C	0.417242000	1.176963000	-0.681889000
C	-4.063556000	-1.327886000	1.448936000
H	-5.004062000	-0.768791000	1.400441000
C	-3.912790000	-1.891729000	2.857779000
H	-2.949249000	-2.396204000	2.976575000
H	-4.697429000	-2.628654000	3.052127000
H	-3.987379000	-1.120820000	3.627598000
C	-4.105666000	-2.452593000	0.421437000
H	-4.216294000	-2.078749000	-0.597818000
H	-4.946109000	-3.121450000	0.629554000
H	-3.189172000	-3.047244000	0.457661000
C	-2.844299000	1.173366000	2.334734000
H	-2.620252000	0.665761000	3.281091000
C	-1.776144000	2.238443000	2.118802000
H	-0.772717000	1.808871000	2.097549000
H	-1.813669000	2.980652000	2.921721000
H	-1.931817000	2.766434000	1.174120000
C	-4.225388000	1.801252000	2.433397000
H	-4.491520000	2.314105000	1.505919000
H	-4.242656000	2.545702000	3.236148000
H	-5.004686000	1.068138000	2.652101000
C	4.297764000	-0.714493000	0.109161000
H	4.943419000	-0.162422000	0.801751000
C	4.727713000	-0.391210000	-1.317038000
H	4.140112000	-0.949049000	-2.046608000
H	5.776449000	-0.672095000	-1.450563000
H	4.633301000	0.671002000	-1.548581000
C	4.436404000	-2.204545000	0.394637000
H	4.213277000	-2.456938000	1.432642000
H	5.461291000	-2.525219000	0.188347000
H	3.769911000	-2.787155000	-0.245284000
C	2.379646000	-0.117811000	2.311962000
H	1.642642000	0.684768000	2.439092000
C	3.634134000	0.267286000	3.083377000
H	4.401543000	-0.507930000	3.017405000
H	3.387885000	0.387656000	4.142325000
H	4.064506000	1.209575000	2.740910000
C	1.761070000	-1.394697000	2.866965000
H	0.792294000	-1.603543000	2.411820000
H	1.610390000	-1.284716000	3.945064000
H	2.398314000	-2.267675000	2.713265000
C	0.854098000	-2.783609000	0.013094000
C	-0.985458000	-1.862876000	-1.734683000
C	1.528199000	-1.434174000	-2.350091000

14 (PBE1PBE/Def2-TZVP)

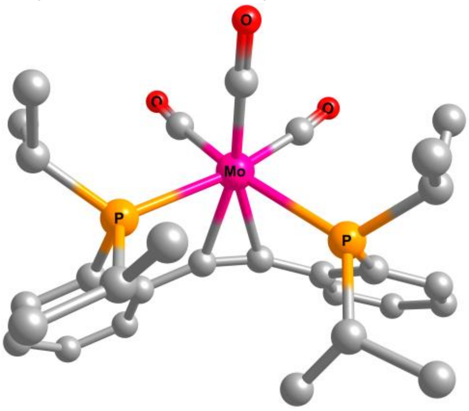

Mo	0.011441000	-0.442368000	-1.011665000
P	-1.940204000	-0.578202000	0.688510000
P	2.171227000	-0.439059000	0.356839000
O	0.224573000	-3.516663000	-1.560201000
O	-2.164644000	-0.316651000	-3.247581000
O	1.573626000	0.182903000	-3.630077000
C	-2.945008000	0.882145000	0.206941000
C	-2.204550000	1.927324000	-0.378477000
C	-2.839494000	3.111520000	-0.750821000
H	-2.260613000	3.906223000	-1.205815000
C	-4.204375000	3.252554000	-0.560255000
H	-4.698573000	4.165898000	-0.870992000
C	-4.939301000	2.226652000	0.015225000
H	-6.009353000	2.334140000	0.150477000
C	-4.306869000	1.053388000	0.409252000
H	-4.896268000	0.258778000	0.852289000
C	2.681273000	1.327394000	0.440074000
C	1.710438000	2.251998000	0.021632000
C	1.992386000	3.621887000	0.043126000
H	1.234301000	4.322493000	-0.285839000
C	3.230022000	4.069488000	0.464783000
H	3.447545000	5.131463000	0.466284000
C	4.196611000	3.159671000	0.873993000
H	5.173677000	3.508237000	1.188295000
C	3.917927000	1.801070000	0.866540000
H	4.692446000	1.106902000	1.169574000
C	-0.800455000	1.714478000	-0.548981000
C	0.434009000	1.767478000	-0.402559000
C	-3.114135000	-2.011477000	0.600419000
H	-3.981924000	-1.739881000	1.210691000
C	-2.468519000	-3.255668000	1.201531000
H	-1.587423000	-3.552874000	0.628272000
H	-3.175735000	-4.089368000	1.172514000
H	-2.166440000	-3.115303000	2.241602000
C	-3.591665000	-2.284761000	-0.818196000
H	-4.039275000	-1.405913000	-1.284717000
H	-4.343002000	-3.079837000	-0.802132000
H	-2.769180000	-2.617706000	-1.453068000
C	-1.769864000	-0.311450000	2.539891000
H	-1.059916000	-1.089164000	2.841412000
C	-1.138399000	1.046739000	2.825964000
H	-0.248748000	1.230934000	2.222838000
H	-0.857692000	1.112661000	3.881175000
H	-1.845830000	1.854314000	2.622048000
C	-3.043599000	-0.471171000	3.358602000
H	-3.802891000	0.254416000	3.055636000
H	-2.823965000	-0.284322000	4.414508000
H	-3.474547000	-1.470786000	3.288697000
C	3.733394000	-1.240614000	-0.267608000
H	4.505114000	-0.950904000	0.454784000
C	4.143309000	-0.713123000	-1.636087000
H	3.484576000	-1.091161000	-2.417393000
H	5.157284000	-1.054915000	-1.863992000
H	4.136657000	0.376633000	-1.689074000
C	3.654286000	-2.762115000	-0.293529000
H	3.548561000	-3.196986000	0.700197000
H	4.573767000	-3.163424000	-0.729660000
H	2.821150000	-3.109458000	-0.907201000
C	2.087857000	-0.945832000	2.160593000
H	1.414177000	-0.179078000	2.559167000
C	3.383386000	-0.893577000	2.957466000
H	4.114276000	-1.619465000	2.592479000
H	3.179651000	-1.143566000	4.003366000
H	3.841212000	0.095969000	2.944660000
C	1.397218000	-2.295572000	2.334467000
H	0.520421000	-2.386000000	1.690908000
H	1.080279000	-2.419312000	3.374561000
H	2.064479000	-3.126764000	2.102231000
C	0.156980000	-2.385331000	-1.342335000
C	-1.422204000	-0.371245000	-2.370227000
C	1.075588000	-0.060390000	-2.619127000

13 (PBE1PBE/Def2-TZVP-GD3, PCM for thf)

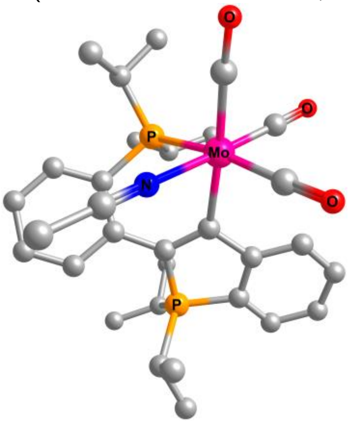

Mo	1.617841000	-1.182773000	0.608182000
P	1.803540000	0.836750000	-0.883893000
P	-2.909972000	0.294138000	-0.100205000
O	0.904247000	-3.660617000	2.369915000
O	4.628802000	-1.619990000	1.364786000
O	2.307709000	-3.320855000	-1.534547000
N	1.276949000	0.230404000	2.249745000
C	0.741363000	2.144022000	-0.152982000
C	1.182079000	3.457036000	-0.019315000
H	2.155338000	3.736788000	-0.400894000
C	0.413393000	4.440283000	0.589356000
H	0.794495000	5.450962000	0.677574000
C	-0.836588000	4.107074000	1.079710000
H	-1.454736000	4.848451000	1.573299000
C	-1.300704000	2.810464000	0.933565000
H	-2.278447000	2.572968000	1.333211000
C	-0.550278000	1.803805000	0.316614000
C	-1.146048000	0.472591000	0.125834000
C	-0.526015000	-0.761414000	0.074643000
C	-1.491000000	-1.831062000	-0.305257000
C	-1.191815000	-3.171664000	-0.524929000
H	-0.177439000	-3.516396000	-0.383461000
C	-2.183274000	-4.055163000	-0.934527000
H	-1.927182000	-5.094299000	-1.108534000
C	-3.490109000	-3.629790000	-1.133399000
H	-4.248819000	-4.328755000	-1.464795000
C	-3.821721000	-2.299575000	-0.905745000
H	-4.838655000	-1.957850000	-1.061388000
C	-2.825879000	-1.431384000	-0.494607000
C	1.032442000	0.776104000	-2.582403000
H	-0.027111000	0.726852000	-2.314099000
C	1.342539000	-0.481213000	-3.379891000
H	2.397055000	-0.555985000	-3.650958000
H	0.763925000	-0.470825000	-4.308869000
H	1.076101000	-1.377651000	-2.822616000
C	1.259881000	2.041000000	-3.397123000
H	1.021059000	2.943829000	-2.830242000
H	0.622179000	2.027434000	-4.286131000
H	2.294350000	2.118167000	-3.741402000
C	3.453491000	1.648815000	-1.110580000
H	3.304765000	2.591418000	-1.647251000
C	4.352312000	0.749726000	-1.953604000
H	4.008234000	0.666004000	-2.984820000
H	5.366341000	1.158851000	-1.974056000
H	4.404410000	-0.256062000	-1.527620000
C	4.115266000	1.927389000	0.235121000
H	3.483959000	2.507838000	0.909505000
H	5.044626000	2.482785000	0.078073000
H	4.362942000	0.991815000	0.738060000
C	-3.712491000	1.202347000	-1.470810000
H	-4.613273000	0.605334000	-1.661874000
C	-2.821496000	1.154100000	-2.706404000
H	-1.947650000	1.795315000	-2.574260000
H	-3.379868000	1.520524000	-3.570458000
H	-2.477620000	0.141845000	-2.929081000
C	-4.131192000	2.626087000	-1.125902000
H	-4.801208000	2.670646000	-0.265677000
H	-4.663037000	3.052451000	-1.979530000
H	-3.264248000	3.257059000	-0.923715000
C	-3.907476000	0.507599000	1.427814000
H	-3.985525000	1.588554000	1.578394000
C	-5.314909000	-0.055003000	1.263905000
H	-5.842160000	0.367362000	0.405954000
H	-5.897343000	0.181048000	2.157233000
H	-5.293117000	-1.141090000	1.159692000
C	-3.175068000	-0.111198000	2.611539000
H	-3.031910000	-1.184735000	2.467332000
H	-3.768809000	0.031431000	3.517132000
H	-2.194034000	0.341162000	2.764551000
C	1.160433000	-2.716303000	1.741891000
C	3.512648000	-1.422027000	1.100996000
C	2.013507000	-2.485414000	-0.772467000
C	1.061983000	1.006383000	3.068700000
C	0.770302000	2.010608000	4.063129000
H	1.697367000	2.417086000	4.470444000
H	0.183674000	1.578715000	4.875549000
H	0.200365000	2.817601000	3.596900000

TS1 (PBE1PBE/Def2-TZVP-GD3, PCM for thf)

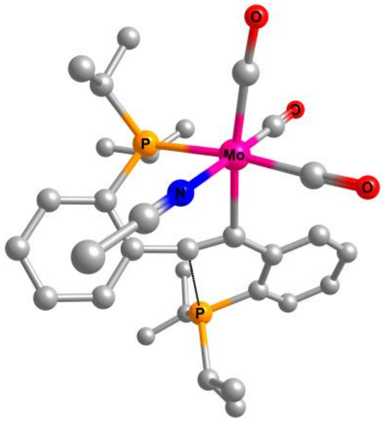

Mo	1.652210000	-1.270738000	0.487045000
P	1.953428000	0.802498000	-0.969645000
P	-3.162514000	0.633636000	-0.047588000
O	1.025220000	-3.647856000	2.406577000
O	4.571987000	-2.315718000	0.548709000
O	1.495338000	-3.342536000	-1.818249000
C	1.086997000	2.133204000	-0.058302000
C	1.560021000	3.429467000	0.099012000
H	2.493989000	3.723396000	-0.364234000
C	0.861838000	4.364486000	0.851524000
H	1.256209000	5.367109000	0.969178000
C	-0.338139000	4.007123000	1.447978000
H	-0.891126000	4.726366000	2.040849000
C	-0.838204000	2.727319000	1.282465000
H	-1.784973000	2.455169000	1.729699000
C	-0.139645000	1.774441000	0.538471000
C	-0.656953000	0.441371000	0.378683000
C	-0.676502000	-0.819572000	0.276747000
C	-1.764722000	-1.728144000	-0.039580000
C	-1.601656000	-3.104924000	-0.156236000
H	-0.627622000	-3.534050000	0.032565000
C	-2.666695000	-3.913240000	-0.522828000
H	-2.521190000	-4.983645000	-0.612229000
C	-3.907539000	-3.354338000	-0.788593000
H	-4.737700000	-3.982222000	-1.091404000
C	-4.087214000	-1.982660000	-0.662535000
H	-5.061412000	-1.557810000	-0.875599000
C	-3.036296000	-1.159966000	-0.275986000
C	1.038206000	0.951881000	-2.590131000
H	-0.000142000	0.972178000	-2.237383000
C	1.178106000	-0.253858000	-3.507348000
H	2.198811000	-0.382682000	-3.871638000
H	0.533160000	-0.117251000	-4.380470000
H	0.880253000	-1.174299000	-3.009271000
C	1.321814000	2.256134000	-3.321553000
H	1.204479000	3.127711000	-2.673983000
H	0.625903000	2.367313000	-4.157959000
H	2.333131000	2.270727000	-3.735755000
C	3.655008000	1.463275000	-1.253028000
H	3.561484000	2.454031000	-1.709639000
C	4.415076000	0.553655000	-2.212364000
H	3.991673000	0.563666000	-3.217026000
H	5.454161000	0.885576000	-2.286773000
H	4.417746000	-0.478348000	-1.852048000
C	4.403049000	1.583134000	0.070132000
H	3.884242000	2.216929000	0.791096000
H	5.393983000	2.010433000	-0.106921000
H	4.533298000	0.599765000	0.526254000
C	-4.201846000	1.206253000	-1.464987000
H	-5.118132000	0.606246000	-1.495716000
C	-3.425999000	0.991800000	-2.759479000
H	-2.507975000	1.586841000	-2.765213000
H	-4.031414000	1.301319000	-3.615729000
H	-3.150465000	-0.055530000	-2.900712000
C	-4.570366000	2.672365000	-1.275288000
H	-5.182715000	2.835142000	-0.385945000
H	-5.137645000	3.029449000	-2.139075000
H	-3.672820000	3.291740000	-1.186524000
C	-4.220379000	0.817016000	1.472634000
H	-4.238327000	1.901806000	1.636066000
C	-5.653268000	0.319905000	1.353615000
H	-6.189072000	0.776188000	0.519122000
H	-6.202392000	0.559235000	2.269280000
H	-5.682220000	-0.764761000	1.229734000
C	-3.513042000	0.163393000	2.655314000
H	-3.474022000	-0.921827000	2.530550000
H	-4.057499000	0.374679000	3.579356000
H	-2.486962000	0.517811000	2.776085000
C	1.240454000	-2.748153000	1.707886000
C	3.490826000	-1.883699000	0.542349000
C	1.515914000	-2.530734000	-0.984393000
N	1.861622000	0.108196000	2.175608000
C	1.947601000	0.869791000	3.030795000
C	2.043986000	1.856162000	4.079039000
H	1.469044000	2.739836000	3.794268000
H	1.646535000	1.454223000	5.012206000
H	3.086912000	2.140730000	4.227902000

INT1 (PBE1PBE/Def2-TZVP-GD3, PCM for thf)

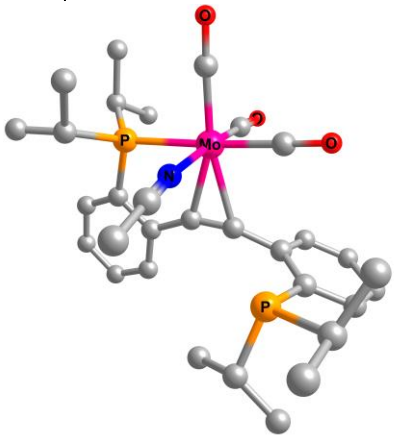

Mo	0.863643000	-1.239737000	-0.168680000
P	-3.255802000	0.347004000	0.858429000
P	2.902362000	0.223651000	0.260386000
O	2.488905000	-3.748387000	0.758382000
O	-1.569010000	-3.175458000	-0.546641000
O	1.615121000	-2.037267000	-3.072329000
C	-3.253865000	-0.021330000	-0.939821000
C	-2.026636000	-0.008804000	-1.627333000
C	-1.993813000	-0.285313000	-3.000066000
H	-1.037273000	-0.284963000	-3.509442000
C	-3.154643000	-0.573963000	-3.691744000
H	-3.109556000	-0.791028000	-4.752874000
C	-4.370400000	-0.593631000	-3.020754000
H	-5.286430000	-0.824736000	-3.552366000
C	-4.409428000	-0.317064000	-1.663991000
H	-5.367434000	-0.336311000	-1.158894000
C	2.469602000	1.902585000	-0.322881000
C	1.137868000	2.041910000	-0.732826000
C	0.674386000	3.283685000	-1.181415000
H	-0.356641000	3.383150000	-1.497211000
C	1.536038000	4.363409000	-1.242213000
H	1.173981000	5.321441000	-1.597628000
C	2.868806000	4.216953000	-0.871600000
H	3.548378000	5.058453000	-0.940648000
C	3.332049000	2.991025000	-0.419136000
H	4.378040000	2.888113000	-0.157566000
C	-0.769259000	0.265254000	-0.985158000
C	0.288861000	0.883213000	-0.739183000
C	-4.764284000	-0.550509000	1.463564000
H	-5.635883000	-0.285220000	0.857357000
C	-5.047208000	-0.144357000	2.905460000
H	-4.185998000	-0.353177000	3.547827000
H	-5.895465000	-0.713750000	3.296256000
H	-5.289933000	0.916320000	2.999463000
C	-4.529111000	-2.052594000	1.359657000
H	-4.346022000	-2.367821000	0.330772000
H	-5.399815000	-2.599437000	1.733479000
H	-3.662176000	-2.352211000	1.955988000
C	-3.791940000	2.138378000	0.875498000
H	-3.835973000	2.371746000	1.946583000
C	-2.691824000	2.990854000	0.255956000
H	-1.716669000	2.790427000	0.705292000
H	-2.916886000	4.054095000	0.380117000
H	-2.610544000	2.791828000	-0.816476000
C	-5.141051000	2.450183000	0.249238000
H	-5.142002000	2.210022000	-0.817094000
H	-5.362499000	3.517879000	0.346862000
H	-5.958108000	1.901153000	0.721831000
C	4.531332000	-0.070249000	-0.570161000
H	5.185465000	0.741625000	-0.233423000
C	4.390648000	0.020708000	-2.084553000
H	3.783898000	-0.798169000	-2.472545000
H	5.380385000	-0.048808000	-2.544408000
H	3.936958000	0.960797000	-2.403270000
C	5.153690000	-1.399370000	-0.165790000
H	5.379318000	-1.449965000	0.899569000
H	6.091693000	-1.542769000	-0.709188000
H	4.493082000	-2.232677000	-0.414092000
C	3.277880000	0.496336000	2.069633000
H	2.381259000	1.040875000	2.388505000
C	4.495769000	1.359589000	2.367341000
H	5.420147000	0.878065000	2.038992000
H	4.576237000	1.509030000	3.447933000
H	4.435894000	2.345354000	1.906996000
C	3.336399000	-0.803112000	2.866715000
H	2.470696000	-1.436680000	2.681436000
H	3.365143000	-0.567715000	3.934621000
H	4.234020000	-1.380132000	2.638555000
C	1.896039000	-2.804472000	0.449545000
C	-0.687139000	-2.454220000	-0.384057000
C	1.362367000	-1.718235000	-1.984422000
N	0.170779000	-0.604267000	1.822605000
C	-0.295908000	-0.222026000	2.798047000
C	-0.893913000	0.269564000	4.013920000
H	-0.189120000	0.908398000	4.548695000
H	-1.786420000	0.844494000	3.758950000
H	-1.178602000	-0.566194000	4.655013000

14 (PBE1PBE/Def2-TZVP-GD3, PCM for thf)

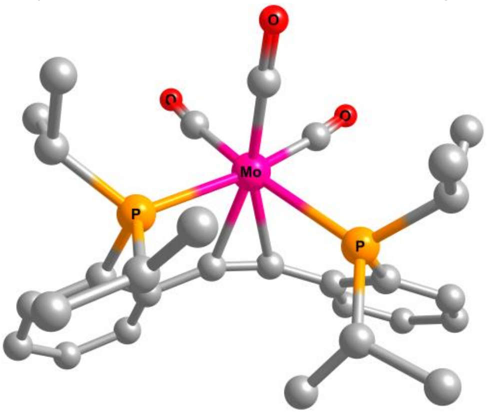

Mo	0.007054000	-0.463682000	-1.038060000
P	-1.885822000	-0.559329000	0.717398000
P	2.143405000	-0.417500000	0.357480000
O	0.226490000	-3.549383000	-1.473503000
O	-2.209974000	-0.413312000	-3.231021000
O	1.539791000	0.087924000	-3.686660000
C	-2.929225000	0.859544000	0.208894000
C	-2.225365000	1.901643000	-0.424837000
C	-2.890277000	3.061705000	-0.817534000
H	-2.337929000	3.856565000	-1.304133000
C	-4.254258000	3.179355000	-0.597849000
H	-4.774382000	4.073804000	-0.920513000
C	-4.954076000	2.155096000	0.024191000
H	-6.022253000	2.245672000	0.183375000
C	-4.289184000	1.006089000	0.438356000
H	-4.850849000	0.214873000	0.921129000
C	2.639001000	1.348895000	0.433238000
C	1.682894000	2.266034000	-0.031819000
C	1.962256000	3.636267000	-0.029105000
H	1.213915000	4.330957000	-0.391416000
C	3.187666000	4.090739000	0.422814000
H	3.405421000	5.152441000	0.412673000
C	4.140480000	3.187824000	0.879496000
H	5.106745000	3.542172000	1.219115000
C	3.862949000	1.828595000	0.888685000
H	4.627686000	1.141581000	1.229366000
C	-0.819382000	1.716379000	-0.610809000
C	0.411937000	1.780231000	-0.469442000
C	-3.029205000	-2.011683000	0.711600000
H	-3.885143000	-1.735976000	1.335186000
C	-2.342203000	-3.222122000	1.333382000
H	-1.475120000	-3.523812000	0.741057000
H	-3.036120000	-4.066555000	1.357769000
H	-2.007993000	-3.038342000	2.356436000
C	-3.535532000	-2.331479000	-0.686627000
H	-4.010773000	-1.470786000	-1.160418000
H	-4.271026000	-3.138851000	-0.631746000
H	-2.720151000	-2.663423000	-1.331855000
C	-1.650008000	-0.209946000	2.539044000
H	-0.924964000	-0.967059000	2.853496000
C	-1.025473000	1.166937000	2.738861000
H	-0.152439000	1.329054000	2.106357000
H	-0.719278000	1.285854000	3.781579000
H	-1.746828000	1.955870000	2.511460000
C	-2.899184000	-0.344588000	3.398214000
H	-3.675234000	0.356657000	3.080842000
H	-2.650901000	-0.105842000	4.436608000
H	-3.318057000	-1.351483000	3.386365000
C	3.711052000	-1.205200000	-0.254800000
H	4.475958000	-0.908830000	0.470933000
C	4.118573000	-0.671599000	-1.621125000
H	3.469414000	-1.065383000	-2.402990000
H	5.139808000	-0.993700000	-1.842831000
H	4.090320000	0.418242000	-1.673204000
C	3.640157000	-2.726241000	-0.280747000
H	3.540449000	-3.159663000	0.713812000
H	4.560137000	-3.122288000	-0.719834000
H	2.805217000	-3.074943000	-0.891645000
C	2.026847000	-0.933707000	2.148114000
H	1.342069000	-0.175668000	2.542153000
C	3.310630000	-0.877737000	2.962489000
H	4.052767000	-1.592225000	2.598592000
H	3.091418000	-1.141110000	4.001422000
H	3.758189000	0.116313000	2.966422000
C	1.345499000	-2.291290000	2.293463000
H	0.481654000	-2.383196000	1.633537000
H	1.008920000	-2.427152000	3.325215000
H	2.025190000	-3.112963000	2.065272000
C	0.158064000	-2.407543000	-1.298347000
C	-1.445443000	-0.438416000	-2.365598000
C	1.053090000	-0.126378000	-2.657993000

13 (PBE1PBE/Def2-TZVP-GD3, PCM for MeCN)

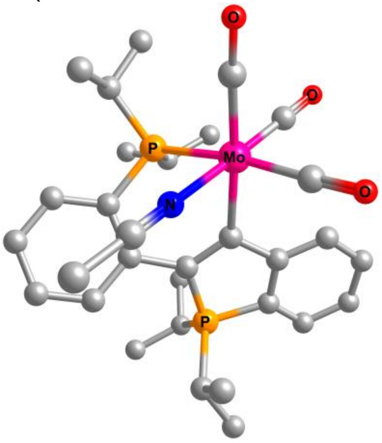

Mo	1.628828000	-1.177676000	0.612388000
P	1.794012000	0.848343000	-0.884580000
P	-2.917872000	0.281037000	-0.100522000
O	0.963379000	-3.674387000	2.363121000
O	4.645666000	-1.604650000	1.336225000
O	2.338011000	-3.310967000	-1.525638000
N	1.277255000	0.241295000	2.255661000
C	0.725114000	2.148145000	-0.152656000
C	1.158027000	3.464145000	-0.022596000
H	2.127858000	3.750926000	-0.407466000
C	0.384039000	4.442284000	0.587573000
H	0.758560000	5.455554000	0.673598000
C	-0.861673000	4.101246000	1.083136000
H	-1.482876000	4.838261000	1.579015000
C	-1.318036000	2.801600000	0.939418000
H	-2.292739000	2.558791000	1.342661000
C	-0.562533000	1.800420000	0.320915000
C	-1.151439000	0.465170000	0.130867000
C	-0.527386000	-0.763837000	0.079955000
C	-1.486167000	-1.838401000	-0.300773000
C	-1.179515000	-3.177569000	-0.518825000
H	-0.163168000	-3.516204000	-0.375224000
C	-2.165649000	-4.067164000	-0.929452000
H	-1.904600000	-5.105317000	-1.102463000
C	-3.474093000	-3.648352000	-1.130777000
H	-4.228986000	-4.351077000	-1.462725000
C	-3.812917000	-2.319158000	-0.905234000
H	-4.831240000	-1.983289000	-1.063434000
C	-2.822599000	-1.445327000	-0.493379000
C	1.022513000	0.777452000	-2.581824000
H	-0.036365000	0.720735000	-2.313026000
C	1.341032000	-0.478401000	-3.378228000
H	2.394722000	-0.541784000	-3.655894000
H	0.756785000	-0.475620000	-4.303570000
H	1.086602000	-1.376099000	-2.817142000
C	1.240068000	2.042712000	-3.398655000
H	0.994545000	2.944356000	-2.832997000
H	0.601184000	2.022273000	-4.286515000
H	2.273691000	2.127305000	-3.743630000
C	3.439625000	1.667949000	-1.111893000
H	3.285098000	2.608766000	-1.649535000
C	4.343827000	0.774107000	-1.954551000
H	3.998656000	0.685422000	-2.984929000
H	5.354187000	1.191976000	-1.978082000
H	4.404731000	-0.230158000	-1.525785000
C	4.100084000	1.953027000	0.233026000
H	3.466858000	2.533748000	0.905242000
H	5.027180000	2.511426000	0.073961000
H	4.350676000	1.019550000	0.738767000
C	-3.715358000	1.187208000	-1.474067000
H	-4.614174000	0.588468000	-1.667752000
C	-2.820918000	1.141326000	-2.707215000
H	-1.950178000	1.786537000	-2.574121000
H	-3.379589000	1.505281000	-3.571948000
H	-2.473487000	0.130173000	-2.929633000
C	-4.138511000	2.609587000	-1.128945000
H	-4.811988000	2.651566000	-0.271566000
H	-4.668372000	3.033660000	-1.984797000
H	-3.274380000	3.243242000	-0.923466000
C	-3.915544000	0.490348000	1.426746000
H	-3.999421000	1.570929000	1.575459000
C	-5.319889000	-0.079428000	1.260673000
H	-5.846681000	0.337269000	0.399969000
H	-5.904349000	0.159144000	2.151863000
H	-5.293703000	-1.165866000	1.161224000
C	-3.182375000	-0.122533000	2.612961000
H	-3.031369000	-1.195301000	2.469862000
H	-3.781545000	0.016282000	3.515374000
H	-2.206856000	0.339590000	2.771752000
C	1.197015000	-2.718236000	1.740498000
C	3.524118000	-1.404799000	1.087332000
C	2.032545000	-2.474830000	-0.765823000
C	1.055931000	1.021820000	3.068506000
C	0.756172000	2.030627000	4.055329000
H	1.680039000	2.458045000	4.447906000
H	0.185410000	1.596107000	4.877396000
H	0.166137000	2.821172000	3.586343000

TS1 (PBE1PBE/Def2-TZVP-GD3, PCM for MeCN)

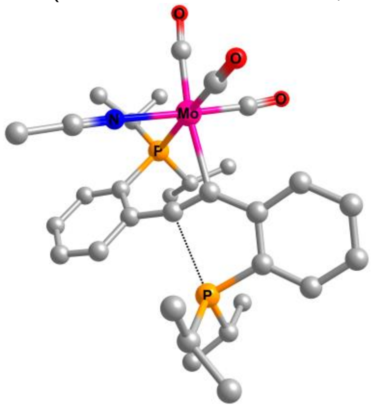

Mo	1.656785000	1.275006000	-0.487004000
P	1.955160000	-0.808854000	0.968739000
P	-3.174575000	-0.636353000	0.048839000
O	1.055842000	3.670128000	-2.389326000
O	4.573207000	2.323827000	-0.526181000
O	1.492535000	3.347799000	1.814324000
C	1.088586000	-2.136783000	0.055259000
C	1.559789000	-3.433832000	-0.100123000
H	2.492837000	-3.729911000	0.363376000
C	0.859858000	-4.367500000	-0.852690000
H	1.252662000	-5.370716000	-0.969581000
C	-0.338464000	-4.008472000	-1.451488000
H	-0.891557000	-4.726817000	-2.045089000
C	-0.836273000	-2.727486000	-1.288620000
H	-1.781744000	-2.454351000	-1.737642000
C	-0.136120000	-1.776531000	-0.543980000
C	-0.649137000	-0.442241000	-0.386163000
C	-0.681752000	0.816705000	-0.287284000
C	-1.769250000	1.726107000	0.028042000
C	-1.604933000	3.103541000	0.139400000
H	-0.630477000	3.530792000	-0.051939000
C	-2.668578000	3.914964000	0.503695000
H	-2.522417000	4.985719000	0.588366000
C	-3.909697000	3.357997000	0.772720000
H	-4.739355000	3.987475000	1.073426000
C	-4.090262000	1.985802000	0.652456000
H	-5.064681000	1.563152000	0.868270000
C	-3.041114000	1.159408000	0.268168000
C	1.040186000	-0.956014000	2.588969000
H	0.001602000	-0.976317000	2.236986000
C	1.181401000	0.249719000	3.506041000
H	2.201698000	0.374894000	3.872967000
H	0.534056000	0.114858000	4.377541000
H	0.887000000	1.170766000	3.006599000
C	1.323629000	-2.260153000	3.320740000
H	1.205775000	-3.131732000	2.673473000
H	0.627216000	-2.370120000	4.156752000
H	2.335009000	-2.275220000	3.734554000
C	3.657323000	-1.467553000	1.249467000
H	3.563517000	-2.458575000	1.704955000
C	4.418484000	-0.560428000	2.210174000
H	3.994387000	-0.570155000	3.214497000
H	5.456254000	-0.896104000	2.285403000
H	4.424849000	0.471861000	1.850382000
C	4.404453000	-1.587138000	-0.074169000
H	3.886835000	-2.222664000	-0.794268000
H	5.395729000	-2.013196000	0.103260000
H	4.533123000	-0.603909000	-0.531374000
C	-4.215330000	-1.195875000	1.470078000
H	-5.130611000	-0.594443000	1.496636000
C	-3.440024000	-0.975148000	2.763797000
H	-2.521847000	-1.569940000	2.773467000
H	-4.046386000	-1.280519000	3.620832000
H	-3.165301000	0.073087000	2.900427000
C	-4.586483000	-2.662491000	1.288902000
H	-5.196803000	-2.829566000	0.399042000
H	-5.156887000	-3.011836000	2.153741000
H	-3.690236000	-3.284577000	1.206439000
C	-4.235006000	-0.820810000	-1.469422000
H	-4.254153000	-1.905842000	-1.629815000
C	-5.667191000	-0.321779000	-1.350077000
H	-6.202553000	-0.774956000	-0.513743000
H	-6.216790000	-0.564253000	-2.264591000
H	-5.695767000	0.763357000	-1.229655000
C	-3.529051000	-0.172209000	-2.655649000
H	-3.489066000	0.913612000	-2.535040000
H	-4.075922000	-0.386582000	-3.577462000
H	-2.504190000	-0.529572000	-2.778349000
C	1.258579000	2.759389000	-1.698837000
C	3.492689000	1.886223000	-0.529036000
C	1.514244000	2.532639000	0.981824000
N	1.870958000	-0.109614000	-2.176618000
C	1.961015000	-0.880573000	-3.022833000
C	2.064020000	-1.879759000	-4.057616000
H	1.446723000	-2.739201000	-3.788198000
H	1.719358000	-1.473333000	-5.009477000
H	3.101422000	-2.202368000	-4.159374000

INT1 (PBE1PBE/Def2-TZVP-GD3, PCM for MeCN)

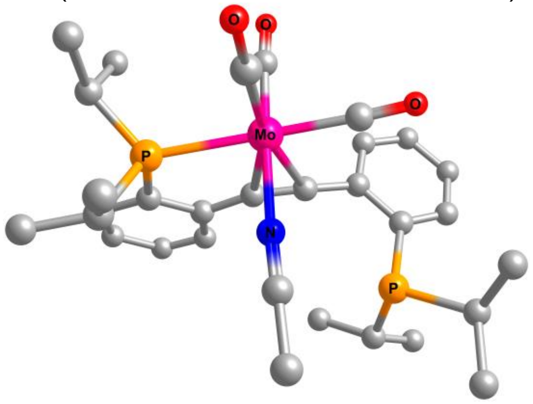

Mo	0.869964000	-1.251796000	-0.114849000
P	-3.256850000	0.408063000	0.843475000
P	2.900876000	0.247722000	0.253021000
O	2.501862000	-3.707317000	0.927881000
O	-1.538006000	-3.224071000	-0.436809000
O	1.638317000	-2.191992000	-2.969578000
C	-3.257580000	-0.087121000	-0.925079000
C	-2.033462000	-0.111323000	-1.618396000
C	-2.004337000	-0.478193000	-2.970003000
H	-1.050529000	-0.501490000	-3.484102000
C	-3.165450000	-0.824052000	-3.634881000
H	-3.123593000	-1.109845000	-4.679746000
C	-4.377800000	-0.810654000	-2.957184000
H	-5.294114000	-1.085227000	-3.467163000
C	-4.413371000	-0.442377000	-1.622044000
H	-5.369207000	-0.434831000	-1.112612000
C	2.458015000	1.892408000	-0.412281000
C	1.127613000	2.004987000	-0.834367000
C	0.659557000	3.221157000	-1.344305000
H	-0.370533000	3.300423000	-1.668597000
C	1.515781000	4.301656000	-1.452417000
H	1.150195000	5.239728000	-1.854247000
C	2.847282000	4.180425000	-1.068369000
H	3.522592000	5.021566000	-1.173250000
C	3.315011000	2.980117000	-0.555698000
H	4.359999000	2.896360000	-0.283905000
C	-0.775385000	0.213851000	-1.002121000
C	0.280466000	0.845309000	-0.789399000
C	-4.748468000	-0.466138000	1.520045000
H	-5.625374000	-0.265062000	0.897241000
C	-5.037697000	0.044742000	2.927044000
H	-4.171304000	-0.092616000	3.581356000
H	-5.872404000	-0.511868000	3.362807000
H	-5.304742000	1.103515000	2.938198000
C	-4.485041000	-1.967144000	1.532137000
H	-4.292461000	-2.356641000	0.530508000
H	-5.347090000	-2.500090000	1.943993000
H	-3.615912000	-2.203037000	2.153675000
C	-3.817306000	2.187871000	0.736141000
H	-3.869933000	2.491211000	1.788876000
C	-2.725666000	3.012702000	0.066494000
H	-1.752289000	2.861638000	0.538434000
H	-2.969339000	4.077961000	0.114057000
H	-2.630627000	2.741257000	-0.988882000
C	-5.167616000	2.437079000	0.084956000
H	-5.159437000	2.129885000	-0.964058000
H	-5.405805000	3.505182000	0.114015000
H	-5.978435000	1.906235000	0.587964000
C	4.532426000	-0.077431000	-0.560029000
H	5.183179000	0.750172000	-0.257304000
C	4.396287000	-0.053195000	-2.077287000
H	3.788539000	-0.887315000	-2.430164000
H	5.387256000	-0.144774000	-2.530386000
H	3.946183000	0.873256000	-2.438243000
C	5.157615000	-1.385490000	-0.095936000
H	5.378468000	-1.388942000	0.971546000
H	6.098888000	-1.547830000	-0.628126000
H	4.500940000	-2.231079000	-0.311621000
C	3.267559000	0.605234000	2.048466000
H	2.366733000	1.157497000	2.340569000
C	4.478690000	1.489706000	2.309263000
H	5.407603000	1.000344000	2.006440000
H	4.553005000	1.688975000	3.382122000
H	4.413586000	2.452868000	1.804063000
C	3.332674000	-0.656075000	2.904178000
H	2.473824000	-1.305403000	2.742617000
H	3.350381000	-0.371053000	3.959952000
H	4.238000000	-1.233349000	2.709171000
C	1.906814000	-2.778497000	0.576724000
C	-0.666638000	-2.484377000	-0.290025000
C	1.379042000	-1.815762000	-1.900181000
N	0.173053000	-0.517159000	1.843263000
C	-0.286879000	-0.075039000	2.796318000
C	-0.870841000	0.494846000	3.984077000
H	-0.120321000	1.065062000	4.533872000
H	-1.689954000	1.155010000	3.692594000
H	-1.261371000	-0.297247000	4.624686000

14 (PBE1PBE/Def2-TZVP-GD3, PCM for MeCN)

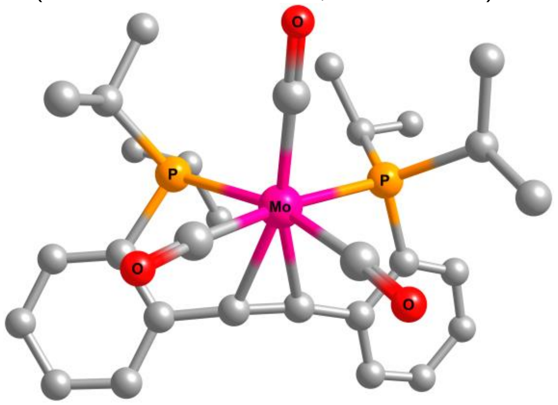

Mo	0.007239000	-0.465370000	-1.039155000
P	-1.888030000	-0.557972000	0.720172000
P	2.145218000	-0.417589000	0.361311000
O	0.222781000	-3.549217000	-1.480476000
O	-2.205495000	-0.412297000	-3.233603000
O	1.535243000	0.074240000	-3.691107000
C	-2.929751000	0.860109000	0.206764000
C	-2.225076000	1.902802000	-0.425101000
C	-2.888935000	3.063458000	-0.818199000
H	-2.335432000	3.859959000	-1.300834000
C	-4.253581000	3.180680000	-0.601041000
H	-4.772921000	4.075945000	-0.922581000
C	-4.954441000	2.155496000	0.018593000
H	-6.022711000	2.246135000	0.176841000
C	-4.290315000	1.006073000	0.433460000
H	-4.852810000	0.215267000	0.915762000
C	2.641957000	1.348325000	0.429734000
C	1.686392000	2.266153000	-0.035009000
C	1.966921000	3.636211000	-0.034936000
H	1.218365000	4.331592000	-0.395524000
C	3.193995000	4.089715000	0.414037000
H	3.412781000	5.151150000	0.402708000
C	4.146515000	3.186052000	0.870085000
H	5.113609000	3.539524000	1.208110000
C	3.867539000	1.826997000	0.882014000
H	4.631999000	1.140041000	1.223188000
C	-0.818211000	1.720384000	-0.607812000
C	0.412658000	1.783432000	-0.468359000
C	-3.029958000	-2.010981000	0.714404000
H	-3.886042000	-1.735344000	1.337540000
C	-2.342104000	-3.220049000	1.337901000
H	-1.474364000	-3.521729000	0.746362000
H	-3.035964000	-4.064410000	1.363351000
H	-2.008485000	-3.034667000	2.360748000
C	-3.536458000	-2.332650000	-0.683228000
H	-4.015095000	-1.473263000	-1.156106000
H	-4.269930000	-3.141651000	-0.626808000
H	-2.720471000	-2.662443000	-1.329038000
C	-1.651801000	-0.205566000	2.540199000
H	-0.926684000	-0.961663000	2.856520000
C	-1.028263000	1.171994000	2.738705000
H	-0.154364000	1.333880000	2.107344000
H	-0.723017000	1.290965000	3.781550000
H	-1.749916000	1.960380000	2.510285000
C	-2.901888000	-0.340176000	3.398058000
H	-3.678890000	0.359109000	3.078754000
H	-2.653732000	-0.099062000	4.435819000
H	-3.319124000	-1.347678000	3.388228000
C	3.710580000	-1.210107000	-0.249503000
H	4.475691000	-0.914663000	0.476115000
C	4.120791000	-0.680153000	-1.616335000
H	3.469156000	-1.071390000	-2.397637000
H	5.140313000	-1.007946000	-1.837226000
H	4.098914000	0.409865000	-1.668814000
C	3.635907000	-2.730973000	-0.272518000
H	3.532635000	-3.161874000	0.722664000
H	4.556412000	-3.129775000	-0.707879000
H	2.801623000	-3.078513000	-0.885157000
C	2.024351000	-0.927797000	2.152338000
H	1.339176000	-0.168466000	2.543087000
C	3.307271000	-0.869161000	2.967830000
H	4.049550000	-1.585370000	2.607816000
H	3.085420000	-1.128247000	4.007164000
H	3.755484000	0.124567000	2.968613000
C	1.342661000	-2.284933000	2.301136000
H	0.480920000	-2.380366000	1.638860000
H	1.002019000	-2.415087000	3.332099000
H	2.023903000	-3.107289000	2.080091000
C	0.155869000	-2.407391000	-1.300140000
C	-1.442722000	-0.438384000	-2.364712000
C	1.051745000	-0.134619000	-2.658074000

Alternative reaction pathways

As mentioned in the article, DFT calculations for two alternative reaction pathways (**13**→**14**) were conducted as well, but higher barriers were found for these alternative mechanistic scenarios. In these alternative scenarios, a coordinatively unsaturated five-coordinated molybdenum complex (**INT4**) and a thf-adduct of this complex (**INT5**) were examined. From both these intermediates, C-P bond cleavage is possible via **TS4** and **TS5**, respectively. The associated energies of these two transition states, however, are significantly higher in energy as **TS1**, which is considered the most plausible transition state (see article text). In the energy diagrams shown below, both alternative transition states **TS4** and **TS5** are shown in comparison to **TS1**. The difference in energies between **TS1** and both alternative transition states is independent of the employed corrections (for solvent and dispersion). Optimised coordinates for the excluded structures **INT4**, **INT5**, **TS4** and **TS5** are provided on the following pages.

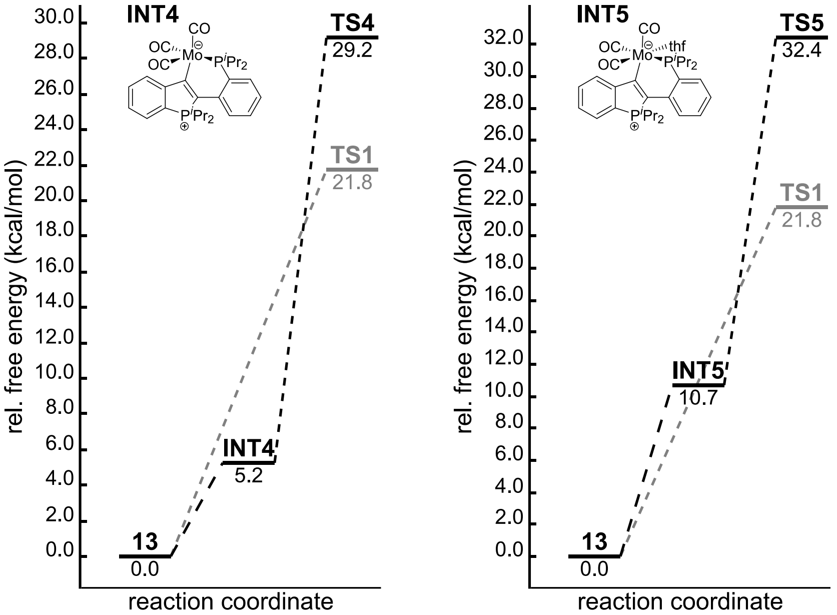

Figure S67: Energy diagrams for the conversion of **13** to **TS4** (left side) and **TS5** (right side) via the corresponding intermediates **INT4** and **INT5**. (PBE1PBE/Def2-TZVP, gas phase, without dispersion or solvent corrections). Compound **13** was set to 0.0 kcal/mol.

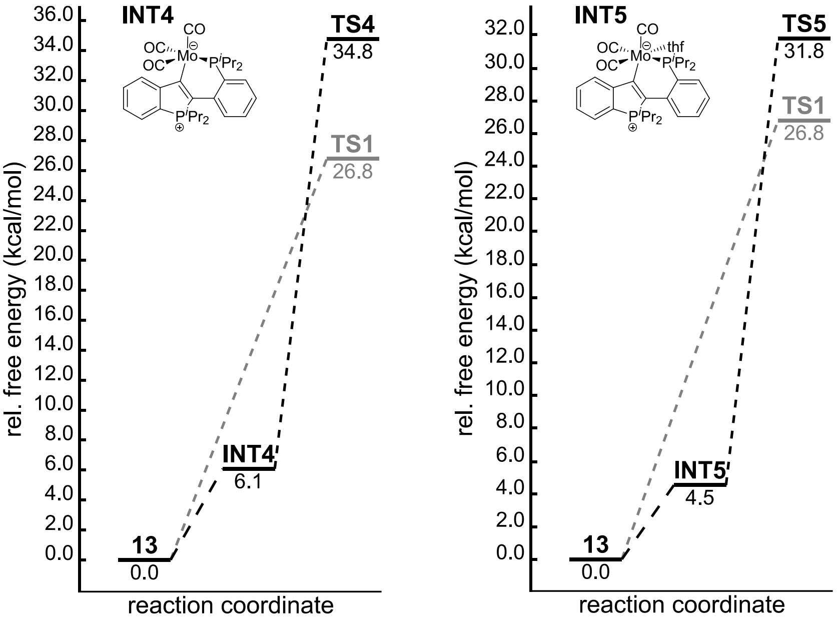

Figure S68: Energy diagrams for the conversion of **13** to **TS4** (left side) and **TS5** (right side) via the corresponding intermediates **INT4** and **INT5**. (PBE1PBE/Def2-TZVP-GD3, PCM solvent correction for thf). Compound **13** was set to 0.0 kcal/mol.

INT4 (PBE1PBE/Def2-TZVP)

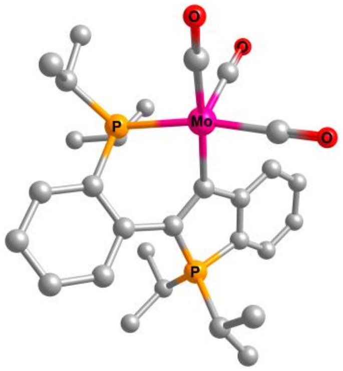

Mo	-1.536155000	-1.220176000	-0.688518000
P	-2.002286000	0.913319000	0.523658000
P	2.813725000	0.432043000	-0.078945000
O	-0.404969000	-3.689470000	-2.240707000
O	-4.175559000	-1.656896000	-2.345152000
O	-2.596393000	-3.285381000	1.309602000
C	-0.937860000	2.116751000	-0.382672000
C	-1.424948000	3.371741000	-0.738860000
H	-2.421488000	3.659580000	-0.430911000
C	-0.684349000	4.280113000	-1.481202000
H	-1.108336000	5.241919000	-1.744540000
C	0.589280000	3.928970000	-1.888243000
H	1.183922000	4.605841000	-2.491501000
C	1.104059000	2.697883000	-1.522681000
H	2.100460000	2.439839000	-1.861554000
C	0.387682000	1.768569000	-0.758819000
C	1.054003000	0.530118000	-0.327029000
C	0.469731000	-0.693511000	-0.023552000
C	1.446407000	-1.636686000	0.567810000
C	1.157231000	-2.903329000	1.064533000
H	0.142362000	-3.274851000	1.003713000
C	2.162233000	-3.671108000	1.636876000
H	1.921278000	-4.653363000	2.027370000
C	3.467054000	-3.201296000	1.722675000
H	4.237246000	-3.811313000	2.179858000
C	3.783790000	-1.945607000	1.220994000
H	4.801116000	-1.575944000	1.289456000
C	2.774575000	-1.185744000	0.652374000
C	-1.323250000	1.101700000	2.253405000
C	-2.207399000	-2.492348000	0.549632000
H	-0.248442000	1.045937000	2.050116000
C	-1.663951000	-0.067577000	3.167998000
H	-2.719841000	-0.087027000	3.442368000
H	-1.087498000	0.011336000	4.095093000
H	-1.426332000	-1.021448000	2.696154000
C	-1.619009000	2.448350000	2.897057000
H	-1.351642000	3.284095000	2.245585000
H	-1.045745000	2.552842000	3.823647000
H	-2.674683000	2.548088000	3.161510000
C	-3.687313000	1.692587000	0.555415000
H	-3.590604000	2.709901000	0.950603000
C	-4.600712000	0.899313000	1.486734000
H	-4.310940000	0.986104000	2.534103000
H	-5.625612000	1.269887000	1.395223000
H	-4.604819000	-0.161051000	1.219966000
C	-4.304243000	1.740695000	-0.838705000
H	-3.659866000	2.212974000	-1.581359000
H	-5.244337000	2.299496000	-0.802513000
H	-4.529128000	0.734089000	-1.192240000
C	3.603795000	1.601629000	1.099298000
H	4.528156000	1.078310000	1.377121000
C	2.735413000	1.740668000	2.344319000
H	1.816175000	2.284033000	2.116602000
H	3.278804000	2.302832000	3.107826000
H	2.464953000	0.770989000	2.766325000
C	3.971653000	2.958096000	0.510170000
H	4.621883000	2.876120000	-0.362945000
H	4.508138000	3.540624000	1.263544000
H	3.082577000	3.522634000	0.225444000
C	3.836198000	0.383168000	-1.612564000
H	3.851284000	1.413436000	-1.983564000
C	5.272551000	-0.048685000	-1.337247000
H	5.771064000	0.578963000	-0.595254000
H	5.850756000	0.016072000	-2.262359000
H	5.312256000	-1.085649000	-0.998757000
C	3.165437000	-0.506560000	-2.652453000
H	3.089674000	-1.538012000	-2.300924000
H	3.761209000	-0.507048000	-3.568471000
H	2.159706000	-0.163028000	-2.896912000
C	-0.815179000	-2.762846000	-1.688851000
C	-3.224782000	-1.476101000	-1.716528000

TS4 (PBE1PBE/Def2-TZVP)

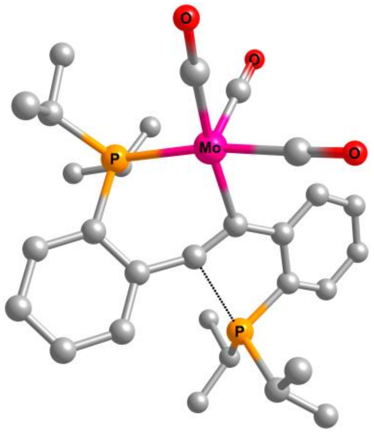

Mo	-1.556338000	-1.225284000	-0.665440000
P	-2.302307000	0.882500000	0.493349000
P	3.089764000	0.672048000	-0.068523000
O	-0.238804000	-3.617857000	-2.184323000
O	-4.202514000	-2.173451000	-2.002652000
O	-2.324448000	-3.232249000	1.509907000
C	-1.282291000	2.149074000	-0.356643000
C	-1.752745000	3.408306000	-0.708972000
H	-2.759875000	3.695188000	-0.432282000
C	-0.974087000	4.307390000	-1.422372000
H	-1.374546000	5.276742000	-1.695104000
C	0.312205000	3.946460000	-1.787858000
H	0.932940000	4.629310000	-2.356513000
C	0.815241000	2.712338000	-1.421411000
H	1.829946000	2.446125000	-1.684813000
C	0.046577000	1.792353000	-0.699880000
C	0.604172000	0.523116000	-0.298901000
C	0.575415000	-0.710479000	0.028821000
C	1.637811000	-1.551104000	0.550721000
C	1.416635000	-2.837043000	1.032307000
H	0.416199000	-3.247397000	0.986622000
C	2.462163000	-3.571549000	1.568509000
H	2.276393000	-4.570166000	1.946623000
C	3.738454000	-3.032509000	1.625691000
H	4.555194000	-3.606043000	2.049060000
C	3.973070000	-1.754416000	1.135787000
H	4.976016000	-1.345325000	1.187316000
C	2.937015000	-1.004575000	0.593001000
C	-1.763184000	1.171570000	2.259454000
H	-0.673714000	1.202229000	2.136256000
C	-2.078098000	0.010861000	3.193763000
H	-3.149128000	-0.099697000	3.371352000
H	-1.603222000	0.186788000	4.163679000
H	-1.707958000	-0.935498000	2.801026000
C	-2.217585000	2.506597000	2.831410000
H	-1.969865000	3.344656000	2.175881000
H	-1.726398000	2.683646000	3.792839000
H	-3.295086000	2.520254000	3.013793000
C	-4.042058000	1.504816000	0.359200000
H	-4.069269000	2.524581000	0.759100000
C	-4.972201000	0.630415000	1.195637000
H	-4.779170000	0.717720000	2.265254000
H	-6.009521000	0.928928000	1.020862000
H	-4.876140000	-0.421908000	0.915881000
C	-4.505737000	1.517097000	-1.093376000
H	-3.852635000	2.099450000	-1.745006000
H	-5.510036000	1.946861000	-1.151794000
H	-4.554603000	0.502025000	-1.490360000
C	4.269258000	1.525110000	1.079365000
H	5.166677000	0.902744000	1.175164000
C	3.619680000	1.664347000	2.451111000
H	2.719852000	2.282990000	2.394775000
H	4.312562000	2.144371000	3.147839000
H	3.338149000	0.696180000	2.869386000
C	4.668330000	2.884156000	0.517509000
H	5.207399000	2.803086000	-0.428611000
H	5.320836000	3.403619000	1.224678000
H	3.789764000	3.515201000	0.354983000
C	4.014285000	0.468942000	-1.673730000
H	4.110032000	1.498277000	-2.042715000
C	5.406464000	-0.134673000	-1.558401000
H	6.053671000	0.423446000	-0.878421000
H	5.888862000	-0.139599000	-2.540537000
H	5.356812000	-1.169999000	-1.214499000
C	3.156503000	-0.312462000	-2.663264000
H	2.996275000	-1.338307000	-2.322736000
H	3.658203000	-0.359803000	-3.633506000
H	2.176276000	0.144378000	-2.811472000
C	-0.715431000	-2.721750000	-1.639644000
C	-3.228604000	-1.792339000	-1.509874000
C	-2.036331000	-2.458862000	0.690143000

INT5 (PBE1PBE/Def2-TZVP)

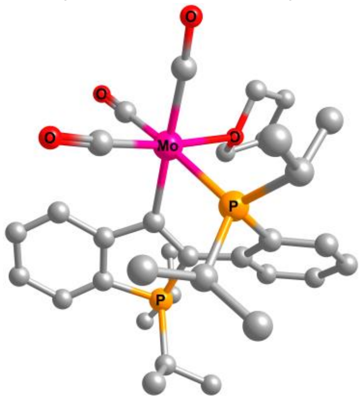

Mo	1.513869000	1.235007000	-0.153326000
P	1.575777000	-0.835815000	1.271596000
P	-3.018692000	-0.379565000	-0.049959000
O	1.147398000	3.737114000	-1.993588000
O	4.588540000	1.881565000	-0.110264000
O	1.528039000	3.333614000	2.101044000
C	0.660839000	-2.127343000	0.326902000
C	1.166582000	-3.416569000	0.181657000
H	2.097972000	-3.679886000	0.665364000
C	0.521106000	-4.395317000	-0.560235000
H	0.954966000	-5.384454000	-0.650497000
C	-0.674444000	-4.084548000	-1.181234000
H	-1.195463000	-4.821943000	-1.781827000
C	-1.203558000	-2.814224000	-1.034302000
H	-2.131746000	-2.585621000	-1.544196000
C	-0.577448000	-1.809794000	-0.285802000
C	-1.244158000	-0.504879000	-0.139547000
C	-0.656554000	0.755202000	-0.035651000
C	-1.694843000	1.800041000	0.208048000
C	-1.471943000	3.163163000	0.382835000
H	-0.466205000	3.548980000	0.310893000
C	-2.526755000	4.020493000	0.664273000
H	-2.323239000	5.075879000	0.806152000
C	-3.828299000	3.550943000	0.780865000
H	-4.637556000	4.231270000	1.018965000
C	-4.086685000	2.201116000	0.589296000
H	-5.099821000	1.825195000	0.680807000
C	-3.027444000	1.355835000	0.299250000
C	0.584772000	-0.885076000	2.856548000
H	-0.425170000	-0.783333000	2.447608000
C	0.800828000	0.298229000	3.790139000
H	1.788619000	0.290206000	4.253030000
H	0.064719000	0.256181000	4.599734000
H	0.683522000	1.249640000	3.275401000
C	0.670908000	-2.210526000	3.600657000
H	0.494182000	-3.067942000	2.947172000
H	-0.078431000	-2.238782000	4.398448000
H	1.645842000	-2.341181000	4.077312000
C	3.195173000	-1.645203000	1.699397000
H	2.980940000	-2.625515000	2.138664000
C	3.945044000	-0.812766000	2.736010000
H	3.460573000	-0.827585000	3.712201000
H	4.955585000	-1.212121000	2.862750000
H	4.037823000	0.227845000	2.415543000
C	4.070794000	-1.825570000	0.463900000
H	3.557390000	-2.332738000	-0.354673000
H	4.957784000	-2.411722000	0.723415000
H	4.410406000	-0.854599000	0.100473000
C	-3.918995000	-1.289628000	1.271289000
H	-4.884038000	-0.767638000	1.306955000
C	-3.231480000	-1.112241000	2.618378000
H	-2.314611000	-1.701684000	2.664686000
H	-3.893810000	-1.463308000	3.413724000
H	-2.981434000	-0.069316000	2.821590000
C	-4.169167000	-2.760250000	0.957259000
H	-4.731411000	-2.904805000	0.032787000
H	-4.751653000	-3.206042000	1.767751000
H	-3.231958000	-3.313793000	0.877532000
C	-3.968012000	-0.672072000	-1.611404000
H	-3.927080000	-1.753176000	-1.778373000
C	-5.433847000	-0.267203000	-1.491224000
H	-5.952095000	-0.774052000	-0.675125000
H	-5.952282000	-0.526714000	-2.417906000
H	-5.534804000	0.810607000	-1.352506000
C	-3.294741000	0.035451000	-2.779332000
H	-3.265615000	1.115971000	-2.622418000
H	-3.857472000	-0.157204000	-3.696212000
H	-2.271908000	-0.308752000	-2.929283000
C	1.270557000	2.787631000	-1.342689000
C	3.465805000	1.603282000	-0.157142000
C	1.498326000	2.509047000	1.278750000
O	1.750776000	-0.178415000	-2.100018000
C	0.754926000	-0.145184000	-3.116060000
C	2.993152000	-0.131582000	-2.801925000
C	1.270060000	-1.086030000	-4.196731000
H	-0.182578000	-0.450352000	-2.659193000
H	0.654247000	0.883525000	-3.485279000
C	2.799767000	-1.034669000	-4.017762000
H	3.194328000	0.905368000	-3.095290000
H	3.773856000	-0.457439000	-2.118723000
H	0.950600000	-0.769616000	-5.190734000
H	0.896785000	-2.097714000	-4.029982000
H	3.309826000	-0.629267000	-4.892501000
H	3.202014000	-2.031538000	-3.832951000

TS5 (PBE1PBE/Def2-TZVP)

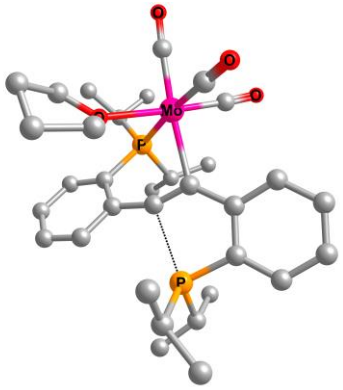

Mo	1.580655000	-1.244787000	0.162494000
P	1.835352000	0.850242000	-1.246197000
P	-3.247898000	0.604300000	0.009654000
O	0.981316000	-3.832506000	1.802621000
O	4.549781000	-2.141352000	0.351321000
O	1.603854000	-3.181167000	-2.231134000
C	0.998477000	2.152639000	-0.260684000
C	1.484299000	3.441340000	-0.075887000
H	2.404610000	3.739589000	-0.563931000
C	0.821714000	4.361707000	0.724708000
H	1.229311000	5.357103000	0.859183000
C	-0.363425000	3.996885000	1.342783000
H	-0.893596000	4.702767000	1.971978000
C	-0.879766000	2.727663000	1.150579000
H	-1.818667000	2.452005000	1.613548000
C	-0.218014000	1.783799000	0.361242000
C	-0.772149000	0.464736000	0.171566000
C	-0.725627000	-0.795693000	-0.019135000
C	-1.822937000	-1.702581000	-0.321934000
C	-1.638062000	-3.052505000	-0.602198000
H	-0.637869000	-3.460983000	-0.562481000
C	-2.715475000	-3.854238000	-0.944479000
H	-2.551727000	-4.902784000	-1.164849000
C	-3.991515000	-3.318262000	-1.019331000
H	-4.832207000	-3.942163000	-1.300945000
C	-4.192012000	-1.974118000	-0.734467000
H	-5.192892000	-1.563703000	-0.807455000
C	-3.125433000	-1.159223000	-0.374690000
C	0.817131000	1.028109000	-2.813242000
H	-0.195702000	1.035130000	-2.394416000
C	0.893325000	-0.154340000	-3.768686000
H	1.883113000	-0.272266000	-4.212267000
H	0.186119000	0.002721000	-4.589324000
H	0.638383000	-1.091001000	-3.277804000
C	1.038603000	2.350008000	-3.535820000
H	0.959906000	3.209720000	-2.866500000
H	0.284685000	2.471778000	-4.319538000
H	2.015714000	2.383962000	-4.024425000
C	3.501000000	1.550774000	-1.670513000
H	3.348971000	2.543699000	-2.107929000
C	4.183562000	0.660773000	-2.706349000
H	3.674659000	0.674689000	-3.670160000
H	5.208124000	1.007536000	-2.867956000
H	4.232521000	-0.375157000	-2.361155000
C	4.388977000	1.675506000	-0.439063000
H	3.954362000	2.309046000	0.335024000
H	5.351767000	2.109065000	-0.725712000
H	4.582381000	0.692854000	-0.004665000
C	-4.349654000	1.291043000	-1.314723000
H	-5.260202000	0.681412000	-1.351069000
C	-3.640463000	1.188917000	-2.659845000
H	-2.733181000	1.799188000	-2.668800000
H	-4.294630000	1.552211000	-3.457400000
H	-3.359494000	0.160941000	-2.896506000
C	-4.727687000	2.732930000	-0.999065000
H	-5.313191000	2.820287000	-0.081477000
H	-5.327553000	3.149058000	-1.813244000
H	-3.836086000	3.357609000	-0.892658000
C	-4.251433000	0.700418000	1.578939000
H	-4.301366000	1.778989000	1.775763000
C	-5.671785000	0.158535000	1.506181000
H	-6.256360000	0.611789000	0.702981000
H	-6.192937000	0.363060000	2.446587000
H	-5.671473000	-0.923862000	1.363612000
C	-3.477182000	0.045404000	2.717026000
H	-3.356210000	-1.026689000	2.542725000
H	-4.016835000	0.171331000	3.659722000
H	-2.482007000	0.478140000	2.837483000
C	1.184888000	-2.846759000	1.233783000
C	3.449389000	-1.778993000	0.296874000
C	1.574718000	-2.414090000	-1.357131000
C	2.717515000	0.744485000	2.678987000
O	1.581058000	0.026093000	2.176390000
C	0.754398000	-0.392525000	3.265627000
C	1.061900000	0.585262000	4.377910000
C	2.562416000	0.780759000	4.192964000
H	3.625950000	0.235590000	2.352386000
H	2.692489000	1.745471000	2.240004000
H	-0.278189000	-0.381105000	2.917775000
H	1.017783000	-1.418055000	3.547801000
H	0.525972000	1.525078000	4.218277000
H	0.795924000	0.196225000	5.361633000
H	2.936005000	1.711067000	4.622246000
H	3.108097000	-0.047143000	4.652161000

INT4 (PBE1PBE/Def2-TZVP-GD3, PCM for thf)

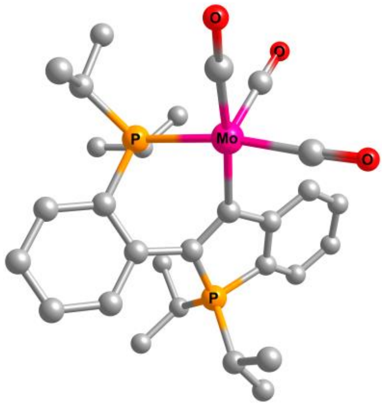

Mo	-1.635030000	-1.160206000	-0.748871000
P	-1.893495000	0.993834000	0.516797000
P	2.837093000	0.336271000	-0.081647000
O	-0.760758000	-3.790662000	-2.180996000
O	-4.445610000	-1.511465000	-2.089173000
O	-2.735362000	-3.092163000	1.354347000
C	-0.814579000	2.163111000	-0.405059000
C	-1.248026000	3.445397000	-0.730373000
H	-2.214178000	3.788228000	-0.383981000
C	-0.478462000	4.317171000	-1.488657000
H	-0.853704000	5.305915000	-1.725028000
C	0.760806000	3.902070000	-1.941903000
H	1.373539000	4.554186000	-2.553596000
C	1.222340000	2.641201000	-1.603099000
H	2.195437000	2.336370000	-1.968600000
C	0.476073000	1.751758000	-0.825042000
C	1.081661000	0.478617000	-0.399632000
C	0.452337000	-0.714472000	-0.120689000
C	1.378111000	-1.696258000	0.492619000
C	1.035434000	-2.958104000	0.963745000
H	0.014823000	-3.302412000	0.858774000
C	1.995661000	-3.758145000	1.571920000
H	1.713849000	-4.736973000	1.943225000
C	3.306231000	-3.322182000	1.718779000
H	4.040134000	-3.954562000	2.203942000
C	3.676784000	-2.069023000	1.243606000
H	4.697362000	-1.722854000	1.359975000
C	2.712961000	-1.281257000	0.640243000
C	-1.120669000	1.092639000	2.207774000
H	-0.062515000	1.005237000	1.947326000
C	-1.451068000	-0.091500000	3.105183000
H	-2.500646000	-0.111790000	3.402672000
H	-0.850547000	-0.033401000	4.018013000
H	-1.225796000	-1.034652000	2.606255000
C	-1.331038000	2.431486000	2.897808000
H	-1.074047000	3.270754000	2.246676000
H	-0.695300000	2.492624000	3.786099000
H	-2.364363000	2.560731000	3.228312000
C	-3.531978000	1.844035000	0.653174000
H	-3.367026000	2.846844000	1.060952000
C	-4.423209000	1.066751000	1.617716000
H	-4.068055000	1.114584000	2.647084000
H	-5.434701000	1.481414000	1.596045000
H	-4.485618000	0.014593000	1.325056000
C	-4.216290000	1.947458000	-0.705570000
H	-3.590733000	2.412558000	-1.468755000
H	-5.129878000	2.541210000	-0.609570000
H	-4.496310000	0.956683000	-1.065546000
C	3.591095000	1.496485000	1.114225000
H	4.478229000	0.948755000	1.456471000
C	2.652584000	1.697027000	2.298147000
H	1.783129000	2.288659000	2.004174000
H	3.177156000	2.240145000	3.086953000
H	2.304219000	0.749280000	2.714041000
C	4.035746000	2.821257000	0.506934000
H	4.736810000	2.690673000	-0.318969000
H	4.540411000	3.408059000	1.277751000
H	3.183277000	3.401674000	0.150786000
C	3.884248000	0.248534000	-1.584838000
H	3.944819000	1.276261000	-1.956234000
C	5.293419000	-0.231124000	-1.254841000
H	5.780875000	0.379136000	-0.491987000
H	5.905038000	-0.180726000	-2.158228000
H	5.284804000	-1.269442000	-0.918533000
C	3.211446000	-0.625444000	-2.635706000
H	3.087860000	-1.649405000	-2.275088000
H	3.837134000	-0.656638000	-3.530197000
H	2.228745000	-0.243657000	-2.915858000
C	-1.071034000	-2.784097000	-1.691552000
C	-3.406758000	-1.346826000	-1.593079000
C	-2.314344000	-2.339340000	0.560077000

TS4 (PBE1PBE/Def2-TZVP-GD3, PCM for thf)

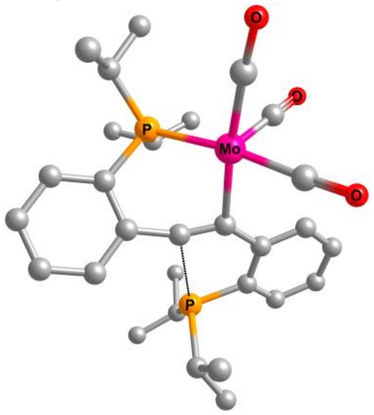

Mo	-1.674303000	-1.337161000	-0.713764000
P	-2.256856000	0.841316000	0.451556000
P	3.054474000	0.603460000	-0.010171000
O	-0.617506000	-3.893724000	-2.155880000
O	-4.496514000	-2.329417000	-1.538478000
O	-2.225294000	-3.215371000	1.633420000
C	-1.284479000	2.064602000	-0.500293000
C	-1.737902000	3.327652000	-0.860016000
H	-2.724382000	3.653301000	-0.553614000
C	-0.951685000	4.187715000	-1.613627000
H	-1.330433000	5.164940000	-1.889051000
C	0.315811000	3.788066000	-2.009859000
H	0.937895000	4.448248000	-2.602688000
C	0.798083000	2.545683000	-1.640048000
H	1.799037000	2.248336000	-1.922110000
C	0.017064000	1.668295000	-0.883683000
C	0.532015000	0.387469000	-0.474693000
C	0.549768000	-0.837403000	-0.159153000
C	1.589791000	-1.682743000	0.392951000
C	1.367571000	-2.995721000	0.795613000
H	0.384539000	-3.426070000	0.660958000
C	2.388499000	-3.735421000	1.371644000
H	2.200906000	-4.755831000	1.685122000
C	3.641208000	-3.169189000	1.554502000
H	4.438821000	-3.742814000	2.012477000
C	3.876305000	-1.861856000	1.147597000
H	4.859875000	-1.432638000	1.300183000
C	2.868231000	-1.107954000	0.559102000
C	-1.551104000	1.151950000	2.149281000
H	-0.477804000	1.179343000	1.926770000
C	-1.779901000	0.009765000	3.128877000
H	-2.835677000	-0.127422000	3.368703000
H	-1.255611000	0.226813000	4.063948000
H	-1.399726000	-0.933386000	2.738038000
C	-1.955251000	2.499817000	2.728015000
H	-1.774305000	3.319310000	2.028633000
H	-1.373491000	2.700281000	3.632004000
H	-3.010943000	2.516258000	3.009513000
C	-3.984166000	1.489279000	0.451249000
H	-3.955795000	2.520133000	0.819134000
C	-4.847590000	0.655414000	1.392452000
H	-4.554242000	0.768297000	2.436453000
H	-5.891253000	0.968615000	1.305286000
H	-4.792562000	-0.405643000	1.133910000
C	-4.564515000	1.468711000	-0.958386000
H	-3.956741000	2.022255000	-1.675746000
H	-5.563128000	1.913954000	-0.947851000
H	-4.658370000	0.442939000	-1.319564000
C	4.126085000	1.395563000	1.270242000
H	5.018466000	0.776470000	1.413702000
C	3.353605000	1.462667000	2.582582000
H	2.452070000	2.072502000	2.472781000
H	3.974028000	1.916378000	3.360154000
H	3.051021000	0.471588000	2.927415000
C	4.552772000	2.781295000	0.802819000
H	5.165360000	2.743567000	-0.100279000
H	5.141118000	3.272215000	1.582642000
H	3.681908000	3.410548000	0.596435000
C	4.106544000	0.452012000	-1.537148000
H	4.199939000	1.488576000	-1.884097000
C	5.500483000	-0.117868000	-1.322644000
H	6.075287000	0.444260000	-0.584035000
H	6.057245000	-0.091637000	-2.264075000
H	5.451145000	-1.160239000	-1.000158000
C	3.345557000	-0.341197000	-2.594266000
H	3.206653000	-1.378297000	-2.277688000
H	3.909851000	-0.350745000	-3.530341000
H	2.358232000	0.078833000	-2.797727000
C	-0.990966000	-2.919487000	-1.654280000
C	-3.447445000	-1.916806000	-1.253048000
C	-2.001606000	-2.480758000	0.753058000

INT5 (PBE1PBE/Def2-TZVP-GD3, PCM for thf)

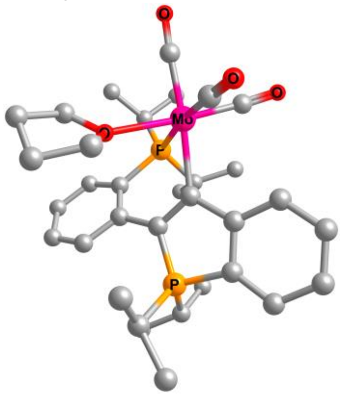

Mo	-1.584894000	1.176875000	0.429383000
P	-1.651299000	-0.675033000	-1.274580000
P	2.967558000	-0.318110000	-0.120046000
O	-1.100085000	3.569489000	2.371031000
O	-4.641856000	1.505843000	1.028103000
O	-2.146737000	3.491892000	-1.523401000
C	-0.677656000	-2.066011000	-0.574689000
C	-1.136031000	-3.379530000	-0.624162000
H	-2.052546000	-3.607010000	-1.153280000
C	-0.457189000	-4.423750000	-0.010649000
H	-0.849868000	-5.432456000	-0.063796000
C	0.715885000	-4.153981000	0.671986000
H	1.255638000	-4.945433000	1.179355000
C	1.207599000	-2.859366000	0.696578000
H	2.128425000	-2.671245000	1.235109000
C	0.550834000	-1.793515000	0.075039000
C	1.190225000	-0.466636000	0.046972000
C	0.595350000	0.779727000	0.048034000
C	1.598459000	1.853900000	-0.182262000
C	1.329355000	3.213056000	-0.301552000
H	0.310874000	3.559535000	-0.193944000
C	2.355595000	4.110109000	-0.572557000
H	2.125469000	5.165220000	-0.669571000
C	3.665620000	3.677678000	-0.732002000
H	4.452682000	4.388024000	-0.955520000
C	3.964719000	2.326017000	-0.608261000
H	4.983446000	1.980790000	-0.741051000
C	2.935637000	1.442984000	-0.331519000
C	-0.697248000	-0.430722000	-2.858859000
H	0.321206000	-0.428919000	-2.463786000
C	-0.898336000	0.914322000	-3.539605000
H	-1.908686000	1.041945000	-3.931281000
H	-0.206074000	0.995070000	-4.383672000
H	-0.698468000	1.736807000	-2.854924000
C	-0.829229000	-1.591978000	-3.832823000
H	-0.664146000	-2.557001000	-3.347744000
H	-0.088440000	-1.489741000	-4.631743000
H	-1.814162000	-1.612282000	-4.306075000
C	-3.268133000	-1.420940000	-1.792327000
H	-3.061341000	-2.292202000	-2.422468000
C	-4.042364000	-0.396972000	-2.617856000
H	-3.568681000	-0.191376000	-3.577758000
H	-5.049393000	-0.772988000	-2.818530000
H	-4.137516000	0.547482000	-2.074675000
C	-4.103768000	-1.849425000	-0.592449000
H	-3.603886000	-2.587875000	0.034498000
H	-5.044499000	-2.286091000	-0.940319000
H	-4.343980000	-0.988336000	0.033017000
C	3.773234000	-1.100374000	-1.567066000
H	4.725979000	-0.559794000	-1.630562000
C	2.978916000	-0.832766000	-2.838238000
H	2.078136000	-1.448119000	-2.863589000
H	3.587348000	-1.098311000	-3.705353000
H	2.688952000	0.215332000	-2.936049000
C	4.059691000	-2.586228000	-1.385277000
H	4.697631000	-2.790594000	-0.524212000
H	4.578420000	-2.954869000	-2.273235000
H	3.136007000	-3.157301000	-1.276024000
C	3.963138000	-0.752335000	1.362289000
H	3.997951000	-1.846107000	1.372419000
C	5.391538000	-0.228263000	1.255858000
H	5.898664000	-0.554105000	0.345846000
H	5.967690000	-0.602904000	2.104761000
H	5.413369000	0.862048000	1.297178000
C	3.273219000	-0.264983000	2.627665000
H	3.120802000	0.816887000	2.605383000
H	3.896909000	-0.499346000	3.493070000
H	2.304820000	-0.744909000	2.767062000
C	-1.262253000	2.637049000	1.693004000
C	-3.507576000	1.342646000	0.818583000
C	-1.906703000	2.582808000	-0.822412000
O	-1.269592000	-0.421370000	2.115005000
C	-0.489500000	-0.062464000	3.254664000
C	-2.174802000	-1.477067000	2.469786000
C	-0.393253000	-1.341560000	4.052456000
H	0.455731000	0.332101000	2.886918000
H	-1.003589000	0.725498000	3.818764000
C	-1.805519000	-1.895825000	3.889109000
H	-3.199391000	-1.108520000	2.385113000
H	-2.026838000	-2.282679000	1.748710000
H	-0.114905000	-1.170895000	5.092778000
H	0.339970000	-2.013387000	3.597405000
H	-2.476224000	-1.427763000	4.613220000
H	-1.862388000	-2.976223000	4.023198000

TS5 (PBE1PBE/Def2-TZVP-GD3, PCM for thf)

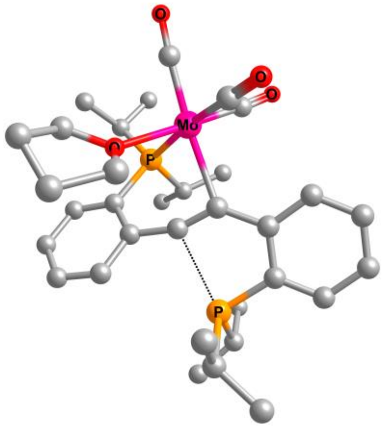

Mo	1.624770000	-1.217683000	0.360775000
P	1.774356000	0.650006000	-1.366574000
P	-3.242021000	0.610460000	-0.066456000
O	1.116689000	-3.532682000	2.384460000
O	4.601220000	-2.061895000	0.539732000
O	1.611697000	-3.498903000	-1.706805000
C	1.008802000	2.087117000	-0.537253000
C	1.486464000	3.390514000	-0.565321000
H	2.369503000	3.629880000	-1.145750000
C	0.853986000	4.400352000	0.148190000
H	1.248982000	5.409231000	0.122297000
C	-0.282921000	4.111317000	0.888369000
H	-0.782333000	4.891523000	1.450744000
C	-0.792623000	2.824260000	0.902644000
H	-1.697717000	2.605535000	1.454135000
C	-0.159148000	1.798160000	0.201499000
C	-0.686764000	0.460003000	0.203114000
C	-0.717145000	-0.802942000	0.193741000
C	-1.809858000	-1.736908000	0.001146000
C	-1.628392000	-3.114904000	-0.060296000
H	-0.634156000	-3.517124000	0.077820000
C	-2.701554000	-3.955362000	-0.312684000
H	-2.544097000	-5.026647000	-0.361703000
C	-3.967377000	-3.427006000	-0.516493000
H	-4.805696000	-4.080683000	-0.728669000
C	-4.161860000	-2.053095000	-0.449457000
H	-5.155155000	-1.654231000	-0.619376000
C	-3.102041000	-1.195517000	-0.179810000
C	0.634719000	0.581978000	-2.849729000
H	-0.339596000	0.718810000	-2.368319000
C	0.590518000	-0.762761000	-3.558795000
H	1.549095000	-1.036159000	-4.003164000
H	-0.146935000	-0.717817000	-4.365878000
H	0.298349000	-1.560180000	-2.877607000
C	0.846420000	1.736916000	-3.817963000
H	0.860735000	2.703843000	-3.309562000
H	0.030208000	1.757405000	-4.545790000
H	1.778560000	1.630321000	-4.378036000
C	3.405592000	1.236354000	-2.004350000
H	3.233504000	2.147054000	-2.587228000
C	3.998095000	0.168680000	-2.919619000
H	3.408243000	0.021291000	-3.824558000
H	5.005567000	0.464364000	-3.224060000
H	4.071322000	-0.790821000	-2.400341000
C	4.367972000	1.542066000	-0.864011000
H	3.994766000	2.314963000	-0.191635000
H	5.321458000	1.886169000	-1.274301000
H	4.560694000	0.645076000	-0.272188000
C	-4.348669000	1.062610000	-1.476968000
H	-5.247711000	0.437918000	-1.433464000
C	-3.614227000	0.781630000	-2.783042000
H	-2.720845000	1.407297000	-2.868159000
H	-4.262567000	1.006010000	-3.634370000
H	-3.304181000	-0.262877000	-2.860233000
C	-4.753148000	2.527593000	-1.372528000
H	-5.345202000	2.732273000	-0.478214000
H	-5.354501000	2.810882000	-2.240606000
H	-3.871949000	3.175822000	-1.352067000
C	-4.238716000	0.898866000	1.477364000
H	-4.293304000	1.993021000	1.535437000
C	-5.653598000	0.339801000	1.478018000
H	-6.240336000	0.678516000	0.621883000
H	-6.175395000	0.662053000	2.384146000
H	-5.643459000	-0.752221000	1.478125000
C	-3.451329000	0.394728000	2.681969000
H	-3.331777000	-0.691278000	2.643450000
H	-3.981787000	0.640210000	3.605692000
H	-2.454717000	0.837592000	2.738468000
C	1.289048000	-2.633256000	1.671337000
C	3.492829000	-1.705278000	0.486480000
C	1.585512000	-2.602543000	-0.958075000
C	2.733241000	1.258119000	2.370435000
O	1.656584000	0.352590000	2.078836000
C	0.818679000	0.198707000	3.230439000
C	1.062928000	1.447104000	4.046408000
C	2.555649000	1.663155000	3.824454000
H	3.678438000	0.749767000	2.172045000
H	2.637447000	2.109932000	1.692903000
H	-0.205381000	0.076401000	2.879041000
H	1.118069000	-0.705843000	3.771846000
H	0.487121000	2.281516000	3.637219000
H	0.796454000	1.317881000	5.095599000
H	2.876738000	2.689299000	4.004248000
H	3.132174000	1.004103000	4.478005000

DFT-Based Comparison of the Molybdenum Complexes 13-16

The Gibbs free energies of complexes **14**, **15** and **16** were calculated relative to **13** employing the methodologies described at the beginning of this section, i.e. at the PBE1PBE/Def2-TZVP[14,15] level of theory either with or without dispersion[16] and solvent correction.[17,18] Experimentally, it was shown that **13** and **14** may be interconverted reversibly, indicative of a nearly thermoneutral reaction. This is only well reproduced *in silico* with solvent and dispersion corrected energies values. Compound **16** was found to be thermodynamically favoured in comparison to **13** by approximately 18 kcal/mol. Optimised coordinates for complexes **13** and **14** are provided in the preceding sub-section (*vide supra*); optimised coordinates for complexes **15** and **16** are provided on the following pages.

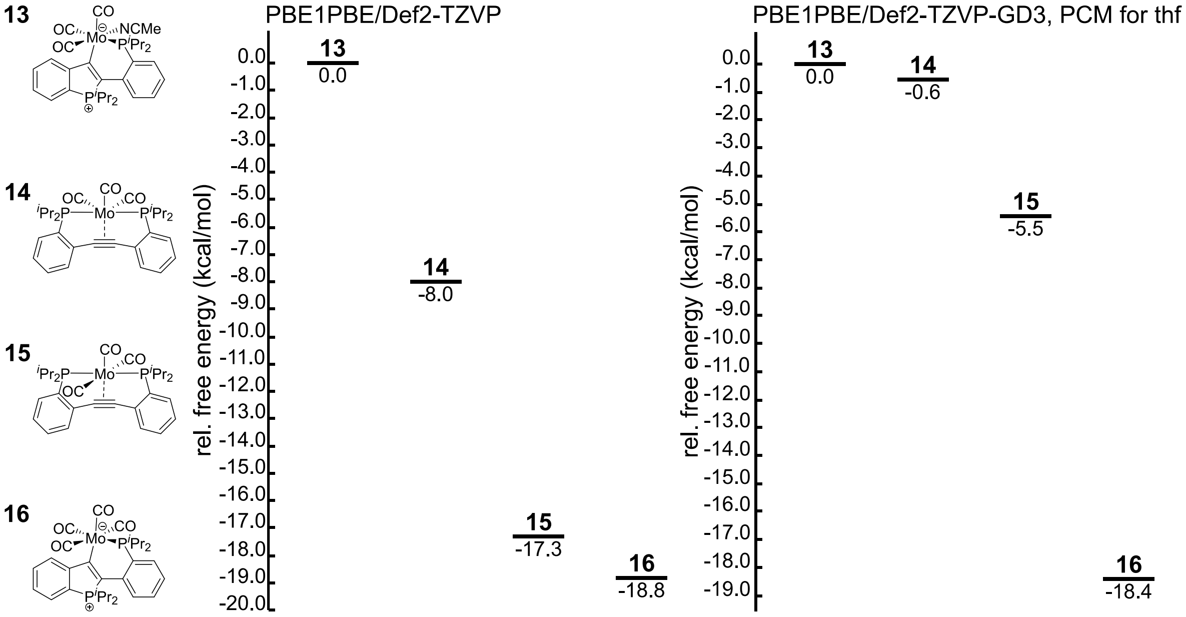

Figure S69: Gibbs free energy diagrams (PBE1PBE/Def2-TZVP) for complexes **13** - **16** with **13** set to 0.0 kcal/mol. Left diagram: no dispersion or solvent corrections; right diagram: energy values for structures optimised with Grimme's dispersion correction GD3 and solvent correction (PCM for thf).

15 (PBE1PBE/Def2-TZVP)

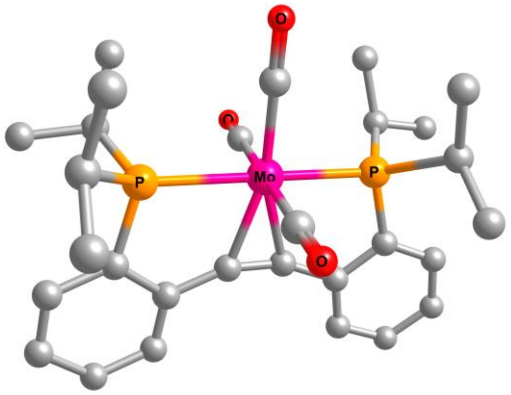

Mo	-0.000012000	-0.484243000	0.038461000
P	2.493118000	-0.455948000	-0.026660000
P	-2.493172000	-0.455925000	-0.026873000
C	3.019732000	1.302447000	-0.052342000
C	1.956896000	2.206352000	-0.185643000
C	2.218451000	3.580007000	-0.261475000
H	1.392729000	4.272509000	-0.374035000
C	3.516567000	4.044135000	-0.174208000
H	3.713294000	5.109109000	-0.225175000
C	4.568748000	3.150845000	-0.002401000
H	5.584640000	3.518276000	0.086591000
C	4.318757000	1.788443000	0.057255000
H	5.147010000	1.105532000	0.204856000
C	-3.019677000	1.302519000	-0.052406000
C	-1.956811000	2.206416000	-0.185492000
C	-2.218316000	3.580111000	-0.260904000
H	-1.392556000	4.272604000	-0.373242000
C	-3.516398000	4.044290000	-0.173452000
H	-3.713066000	5.109290000	-0.224086000
C	-4.568616000	3.150993000	-0.001916000
H	-5.584490000	3.518434000	0.087240000
C	-4.318675000	1.788566000	0.057340000
H	-5.146963000	1.105667000	0.204785000
C	0.629378000	1.677438000	-0.197710000
C	-0.629282000	1.677544000	-0.197852000
C	3.228747000	-1.102596000	-1.626387000
H	2.687211000	-0.490043000	-2.356617000
C	2.876340000	-2.561922000	-1.896525000
H	3.502182000	-3.237490000	-1.309263000
H	3.058840000	-2.789016000	-2.950515000
H	1.832619000	-2.789097000	-1.681686000
C	4.718730000	-0.878506000	-1.844324000
H	4.991903000	0.175751000	-1.821537000
H	4.997876000	-1.266775000	-2.828574000
H	5.326456000	-1.413594000	-1.109462000
C	3.550102000	-1.188108000	1.323856000
H	4.573256000	-1.195183000	0.929844000
C	3.139691000	-2.620972000	1.644209000
H	2.137744000	-2.645273000	2.076938000
H	3.830529000	-3.044039000	2.379365000
H	3.137695000	-3.274846000	0.772838000
C	3.530904000	-0.351227000	2.598260000
H	3.867110000	0.672919000	2.436751000
H	4.192535000	-0.810039000	3.339163000
H	2.531484000	-0.313439000	3.033194000
C	-3.228690000	-1.102527000	-1.626656000
H	-2.687102000	-0.489967000	-2.356851000
C	-2.876214000	-2.561827000	-1.896813000
H	-1.832458000	-2.788923000	-1.682058000
H	-3.058798000	-2.788953000	-2.950786000
H	-3.501937000	-3.237447000	-1.309500000
C	-4.718655000	-0.878465000	-1.844694000
H	-5.326384000	-1.413781000	-1.110002000
H	-4.997689000	-1.266516000	-2.829061000
H	-4.991908000	0.175759000	-1.821699000
C	-3.550337000	-1.188039000	1.323585000
H	-4.573460000	-1.194983000	0.929479000
C	-3.140152000	-2.620967000	1.643943000
H	-3.138429000	-3.274892000	0.772610000
H	-3.830980000	-3.043840000	2.379217000
H	-2.138160000	-2.645461000	2.076536000
C	-3.531137000	-0.351211000	2.598018000
H	-2.531643000	-0.313086000	3.032743000
H	-4.192435000	-0.810310000	3.339042000
H	-3.867739000	0.672820000	2.436641000
C	0.000036000	-0.283474000	2.054493000
C	-0.000063000	-2.465075000	0.242681000
C	0.000009000	-0.679758000	-1.975518000
O	0.000252000	-0.213613000	3.201557000
O	0.000030000	-3.613381000	0.347365000
O	-0.000029000	-0.816502000	-3.116120000

16 (PBE1PBE/Def2-TZVP)

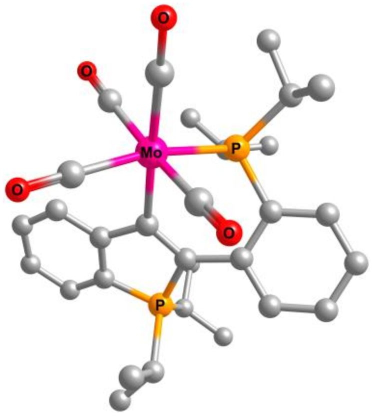

Mo	1.584672000	-1.208918000	-0.538543000
P	1.868392000	0.988064000	0.620056000
P	-2.913305000	0.368063000	-0.007076000
O	4.550369000	-1.848821000	-1.370164000
O	1.435213000	0.293260000	-3.331737000
O	2.392360000	-3.110601000	1.863201000
O	0.624915000	-3.766004000	-2.079203000
C	0.773527000	2.183028000	-0.248724000
C	1.219493000	3.460281000	-0.577896000
H	2.215165000	3.767345000	-0.286736000
C	0.437271000	4.366752000	-1.277397000
H	0.829099000	5.346459000	-1.524419000
C	-0.837269000	3.994084000	-1.661026000
H	-1.465667000	4.671310000	-2.228379000
C	-1.306908000	2.738515000	-1.320427000
H	-2.303161000	2.465551000	-1.645124000
C	-0.542999000	1.805721000	-0.610014000
C	-1.150147000	0.521388000	-0.230532000
C	-0.535895000	-0.702744000	-0.026008000
C	-1.508253000	-1.715449000	0.477675000
C	-2.844899000	-1.296916000	0.596051000
C	-3.846317000	-2.108976000	1.100015000
H	-4.866460000	-1.753671000	1.196344000
C	-3.519899000	-3.398537000	1.499035000
H	-4.283676000	-4.051787000	1.904110000
C	-2.211139000	-3.842977000	1.373785000
H	-1.956952000	-4.851757000	1.678969000
C	-1.214831000	-3.017457000	0.869223000
H	-0.201004000	-3.381080000	0.782190000
C	3.539062000	1.801044000	0.613686000
H	3.424754000	2.815257000	1.010899000
C	4.119001000	1.870518000	-0.795393000
H	4.345596000	0.870758000	-1.166797000
H	5.053646000	2.439212000	-0.776933000
H	3.449243000	2.344365000	-1.514085000
C	4.493794000	1.037836000	1.527081000
H	4.215678000	1.110192000	2.579054000
H	5.503137000	1.445926000	1.422968000
H	4.534555000	-0.020069000	1.255921000
C	1.212053000	1.221889000	2.359842000
H	0.133785000	1.159952000	2.175660000
C	1.561043000	0.099543000	3.326435000
H	1.236930000	-0.868385000	2.947480000
H	1.058455000	0.273358000	4.283277000
H	2.631690000	0.036964000	3.527926000
C	1.513791000	2.592032000	2.951652000
H	2.571330000	2.697640000	3.206517000
H	0.946357000	2.730035000	3.877350000
H	1.244030000	3.404745000	2.273536000
C	-3.748844000	1.423587000	1.244068000
H	-4.661197000	0.856446000	1.470604000
C	-2.897883000	1.494010000	2.506728000
H	-2.592699000	0.504840000	2.853087000
H	-3.469294000	1.971912000	3.306240000
H	-1.999181000	2.089198000	2.333547000
C	-4.147412000	2.809222000	0.751034000
H	-3.271275000	3.413963000	0.512303000
H	-4.701468000	3.322933000	1.540830000
H	-4.791728000	2.773527000	-0.129555000
C	-3.913360000	0.397541000	-1.556696000
H	-3.979317000	1.451837000	-1.843254000
C	-3.187084000	-0.369762000	-2.655236000
H	-2.199665000	0.044597000	-2.861732000
H	-3.776297000	-0.329669000	-3.574621000
H	-3.059471000	-1.420314000	-2.384456000
C	-5.329967000	-0.123921000	-1.339194000
H	-5.324878000	-1.187819000	-1.095940000
H	-5.901270000	-0.001288000	-2.262691000
H	-5.864449000	0.411178000	-0.550902000
C	3.469266000	-1.598683000	-1.058902000
C	1.447001000	-0.207487000	-2.300608000
C	2.035551000	-2.379230000	1.050259000
C	0.983966000	-2.832133000	-1.509488000

15 (PBE1PBE/Def2-TZVP-GD3, PCM for thf)

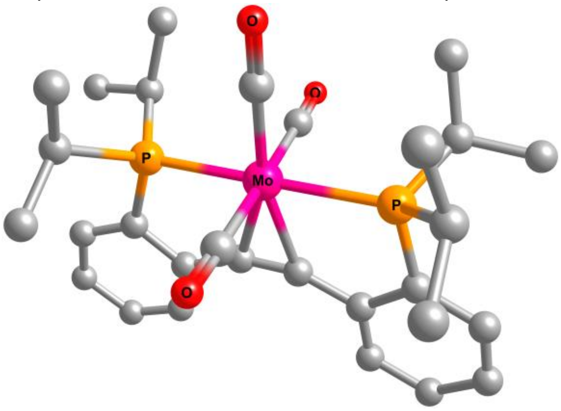

Mo	-0.000037000	-0.485267000	0.025061000
P	2.487366000	-0.450514000	-0.041288000
P	-2.487429000	-0.450423000	-0.041350000
C	3.017683000	1.303084000	-0.058163000
C	1.957294000	2.210811000	-0.178965000
C	2.218001000	3.585186000	-0.240584000
H	1.392388000	4.280071000	-0.341603000
C	3.519072000	4.044213000	-0.153581000
H	3.718728000	5.108994000	-0.193723000
C	4.570475000	3.145658000	0.003756000
H	5.587401000	3.510044000	0.091872000
C	4.319117000	1.782564000	0.051158000
H	5.145608000	1.095739000	0.189154000
C	-3.017683000	1.303196000	-0.058296000
C	-1.957258000	2.210878000	-0.179109000
C	-2.217914000	3.585259000	-0.240834000
H	-1.392273000	4.280106000	-0.341892000
C	-3.518971000	4.044339000	-0.153901000
H	-3.718587000	5.109124000	-0.194125000
C	-4.570408000	3.145832000	0.003474000
H	-5.587324000	3.510259000	0.091540000
C	-4.319102000	1.782732000	0.050965000
H	-5.145623000	1.095948000	0.188984000
C	0.628285000	1.686312000	-0.193304000
C	-0.628266000	1.686335000	-0.193351000
C	3.220475000	-1.101227000	-1.633809000
H	2.671898000	-0.501566000	-2.369381000
C	2.880155000	-2.567464000	-1.878975000
H	3.503811000	-3.224895000	-1.269407000
H	3.077607000	-2.813781000	-2.925707000
H	1.834976000	-2.796052000	-1.671450000
C	4.708110000	-0.865505000	-1.853040000
H	4.966998000	0.192434000	-1.856182000
H	4.993600000	-1.275277000	-2.826365000
H	5.318951000	-1.372451000	-1.101508000
C	3.517479000	-1.197005000	1.316158000
H	4.539509000	-1.252327000	0.925199000
C	3.042669000	-2.604883000	1.657290000
H	2.045286000	-2.573148000	2.100984000
H	3.721753000	-3.053053000	2.387974000
H	2.997925000	-3.265170000	0.791996000
C	3.526222000	-0.337192000	2.575179000
H	3.941044000	0.655896000	2.404467000
H	4.132714000	-0.828738000	3.341329000
H	2.520117000	-0.218263000	2.979675000
C	-3.220517000	-1.101179000	-1.633861000
H	-2.671939000	-0.501534000	-2.369445000
C	-2.880169000	-2.567417000	-1.878978000
H	-1.834977000	-2.795969000	-1.671480000
H	-3.077651000	-2.813783000	-2.925693000
H	-3.503786000	-3.224839000	-1.269359000
C	-4.708153000	-0.865489000	-1.853120000
H	-5.318997000	-1.372408000	-1.101571000
H	-4.993626000	-1.275313000	-2.826428000
H	-4.967059000	0.192445000	-1.856319000
C	-3.517563000	-1.196867000	1.316106000
H	-4.539625000	-1.252024000	0.925209000
C	-3.042921000	-2.604837000	1.657088000
H	-2.998340000	-3.265058000	0.791734000
H	-3.722007000	-3.052975000	2.387790000
H	-2.045497000	-2.573277000	2.100703000
C	-3.526094000	-0.337153000	2.575196000
H	-2.519938000	-0.218382000	2.979614000
H	-4.132580000	-0.828686000	3.341360000
H	-3.940809000	0.655998000	2.404601000
C	-0.000067000	-0.346000000	2.040842000
C	-0.000080000	-2.459364000	0.206101000
C	-0.000021000	-0.698897000	-1.985015000
O	-0.000085000	-0.336407000	3.192557000
O	-0.000116000	-3.611670000	0.296513000
O	-0.000056000	-0.872232000	-3.122133000

16 (PBE1PBE/Def2-TZVP-GD3, PCM for thf)

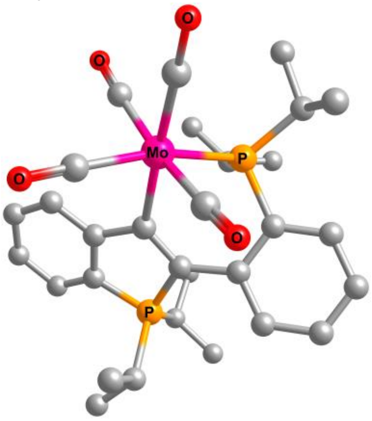

Mo	1.614857000	-1.187871000	-0.622222000
P	1.830738000	0.985471000	0.598459000
P	-2.899171000	0.379077000	0.008696000
O	4.584890000	-1.770733000	-1.443912000
O	1.514271000	0.421769000	-3.354672000
O	2.500283000	-3.055170000	1.775785000
O	0.625793000	-3.711933000	-2.192147000
C	0.763383000	2.187373000	-0.284230000
C	1.209276000	3.468275000	-0.595350000
H	2.189918000	3.789723000	-0.270063000
C	0.434417000	4.363316000	-1.319553000
H	0.819097000	5.349655000	-1.550858000
C	-0.823314000	3.975256000	-1.745349000
H	-1.441935000	4.646611000	-2.329568000
C	-1.294264000	2.715881000	-1.416230000
H	-2.280166000	2.434074000	-1.763639000
C	-0.538993000	1.799786000	-0.678767000
C	-1.138921000	0.516896000	-0.284375000
C	-0.522304000	-0.696254000	-0.076765000
C	-1.473310000	-1.702272000	0.472372000
C	-2.801953000	-1.274252000	0.641378000
C	-3.784890000	-2.073447000	1.195666000
H	-4.797151000	-1.709978000	1.329983000
C	-3.446928000	-3.361253000	1.595772000
H	-4.196294000	-4.004656000	2.040992000
C	-2.147342000	-3.815840000	1.420672000
H	-1.886904000	-4.822535000	1.727446000
C	-1.168267000	-3.000472000	0.864745000
H	-0.160570000	-3.371499000	0.740495000
C	3.495587000	1.791355000	0.678509000
H	3.366941000	2.795927000	1.094166000
C	4.130440000	1.890170000	-0.704428000
H	4.359877000	0.896799000	-1.091387000
H	5.067744000	2.449625000	-0.635694000
H	3.491676000	2.388910000	-1.434505000
C	4.400985000	0.994200000	1.611877000
H	4.068139000	1.031712000	2.649505000
H	5.415471000	1.400182000	1.573475000
H	4.448059000	-0.054483000	1.305309000
C	1.099932000	1.153350000	2.306840000
H	0.032603000	1.116590000	2.070992000
C	1.383035000	-0.014535000	3.238972000
H	1.023178000	-0.951244000	2.814574000
H	0.863622000	0.146543000	4.188455000
H	2.445456000	-0.130203000	3.460130000
C	1.393850000	2.497332000	2.957673000
H	2.436970000	2.575480000	3.273294000
H	0.774182000	2.616671000	3.851224000
H	1.175997000	3.333164000	2.288741000
C	-3.662572000	1.479210000	1.253592000
H	-4.559399000	0.921823000	1.552200000
C	-2.739826000	1.607286000	2.459811000
H	-2.389067000	0.637016000	2.817828000
H	-3.276996000	2.095603000	3.275444000
H	-1.871432000	2.222168000	2.214565000
C	-4.088593000	2.838501000	0.713488000
H	-3.227774000	3.427654000	0.393620000
H	-4.592558000	3.389530000	1.510719000
H	-4.785357000	2.758582000	-0.122349000
C	-3.923631000	0.373205000	-1.513694000
H	-3.995577000	1.420642000	-1.821562000
C	-3.222948000	-0.424053000	-2.606547000
H	-2.244658000	-0.008668000	-2.852566000
H	-3.838949000	-0.413325000	-3.508250000
H	-3.083625000	-1.465239000	-2.305957000
C	-5.330988000	-0.143005000	-1.235111000
H	-5.315835000	-1.202707000	-0.974592000
H	-5.933334000	-0.032301000	-2.139315000
H	-5.832312000	0.406097000	-0.435452000
C	3.492212000	-1.538845000	-1.134866000
C	1.492699000	-0.128151000	-2.346287000
C	2.099688000	-2.345097000	0.961175000
C	1.001513000	-2.787046000	-1.609626000

X-Ray Crystal Structure Determinations

Crystal data and details of the structure determinations are compiled in Tables S2 - S5. Full shells of intensity data were collected at low temperature with a Bruker AXS Smart 1000 CCD diffractometer (Mo-$K\alpha$ radiation, sealed X-ray tube, graphite monochromator; structures **4**, **7**·1.5CH_2Cl_2, **12**·3thf, **13**·CH_3CN and **13-W**·CH_3CN) or an Agilent Technologies Supernova-E CCD diffractometer (Mo- or Cu-K_α radiation, microfocus X-ray tubes, multilayer mirror optics; all other structures).

Detector frames (typically ω-, occasionally φ-scans, scan width 0.4...1°) were integrated by profile fitting.[28-30] Data were corrected for air and detector absorption, Lorentz and polarisation effects[29,30] and scaled essentially by application of appropriate spherical harmonic functions.[31-33] Absorption by the crystal was treated with a semiempirical multiscan method (as part of the scaling procedure), and augmented by a spherical correction,[31-33] or numerically (Gaussian grid).[33-34] For datasets collected with the microfocus tubes an illumination correction was performed as part of the numerical absorption correction.[33]

The structures were solved by "modern" direct methods with dual-space recycling (compound **10**·2thf),[35-37] the Patterson method (compound **11**·2C_6H_5F·Et_2O),[37] by the heavy atom method combined with structure expansion by direct methods applied to difference structure factors (compound **13-W**· CH_3CN),[38] by ab initio dual space methods involving difference Fourier syntheses (VLD procedure, compounds **15** and **15-W**)[35-37,39] or by the charge flip procedure (all other structures).[40]

Refinement was carried out by full-matrix least squares methods based on F^2 against all unique reflections.[41] All non-hydrogen atoms were given anisotropic displacement parameters. Hydrogen atoms were generally input at calculated positions and refined with a riding model.[42] When justified by the quality of the data the positions of some relevant hydrogen atoms (H14 of **3** and those on the arene ligand of **8**) were taken from difference Fourier syntheses and refined. Split atom models were used to refine disordered groups and/or solvent molecules. When found necessary, suitable geometry and adp restraints were applied.[42,43]

Due to severe disorder and fractional occupancy, electron density attributed to all or part of the solvent(s) of crystallisation was removed from the structures of [**8**]SbF_6·0.5C_5H_{12}·0.5CH_2Cl_2 (CH_2Cl_2), [**9**]SbF_6·2CH_2Cl_2 (CH_2Cl_2), **10**·2thf (thf), **11**·2C_6H_5F·Et_2O (C_6H_5F and Et_2O), 12·3thf (thf) with the BYPASS procedure,[44] as implemented in PLATON (squeeze/hybrid).[45] Partial structure factors from the solvent masks were included in the refinement as separate contributions to F_{calc}.

CCDC 2036100 - 2036115 contains the supplementary crystallographic data for this paper. These data can be obtained free of charge from the Cambridge Crystallographic Data Centre's and FIZ Karlsruhe's joint Access Service via https://www.ccdc.cam.ac.uk/structures/?.

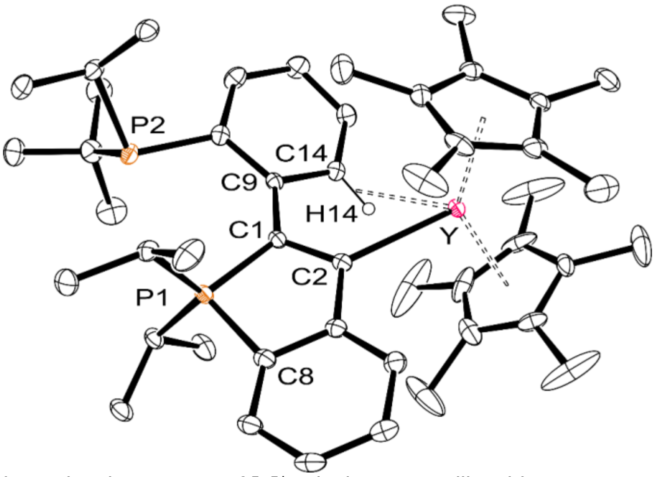

Figure S70: ORTEP plot of the molecular stucture of **[3]**$^+$. Displacement ellipsoids set to 30% probability, hydrogen atoms except H14 ommitted for clarity. Selected bond lenghts (Å) and angles (°): Y-Cp*-plane(C31-C35) 2.3407(14), Y-Cp*-plane(C41-C45) 2.3777(14), Y-Centroid(H14,C14) 2.566, Y-C2 2.471(3), C2-C1 1.371(4), C1-P1 1.825(3), C2-C1-P1 109.6(2), C8-P1-C1 93.39(13), C2-C1-C9 120.7(2).

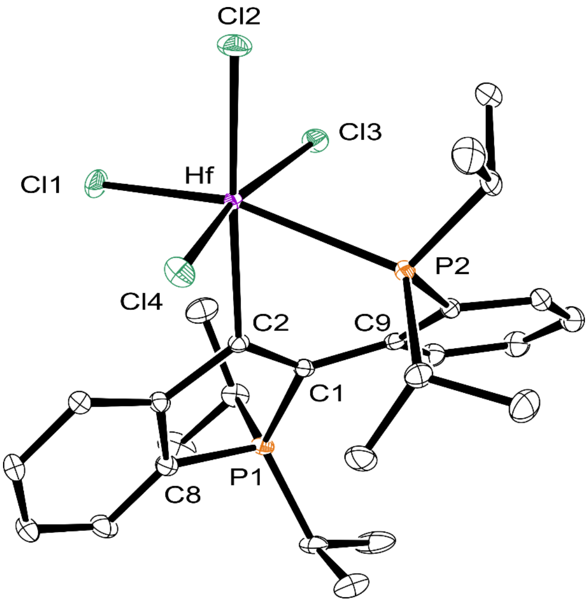

Figure S71: ORTEP plot of the molecular stucture of **4**. Displacement ellipsoids set to 30% probability, hydrogen atoms ommitted for clarity. Selected bond lenghts (Å) and angles (°): Hf-Cl1 2.4493(13), Hf-Cl2 2.3752(10), Hf-Cl3 2.4222(10), Hf-Cl(4) 2.4227(11), Hf-P2 2.7528(12), Hf-C2 2.356(2), C2-C1 1.365(3), C1-P1 1.819(2), C2-C1-P1 109.24(14), C8-P1-C1 93.83(9), C2-C1-C9 131.85(18).

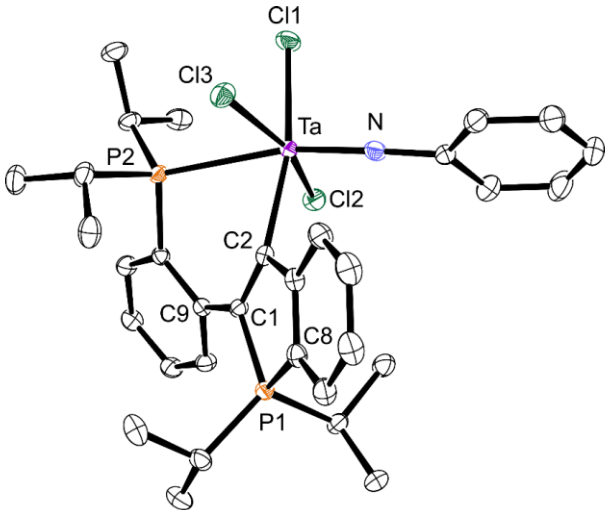

Figure S72: ORTEP plot of the molecular stucture of **5**. Displacement ellipsoids set to 30% probability, hydrogen atoms ommitted for clarity. Selected bond lenghts (Å) and angles (°): Ta-Cl1 2.4374(4), Ta-Cl2 2.4018(4), Ta-Cl3 2.4200(4), Ta-P2 2.7960(4), Ta-N 1.8010(15), Ta-C2 2.2623(16), C2-C1 1.374(2), C1-P1 1.8151(16), C2-C1-P1 108.87(12), C1-P1-C8 94.36(8), C(2)-C(1)-C(9) 131.49(15).

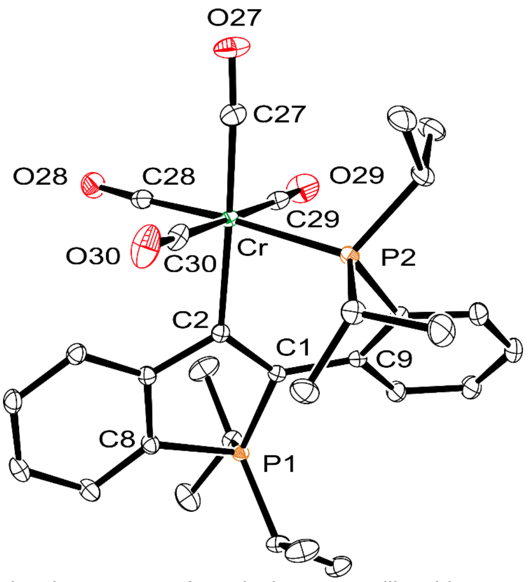

Figure S73: ORTEP plot of the molecular stucture of **6**. Displacement ellipsoids set to 30% probability, hydrogen atoms ommitted for clarity. Selected bond lenghts (Å) and angles (°):Cr-C27 1.8473(14), C27-O27 1.1647(17), Cr-C28 1.8474(14), C28-O28 1.1574(17), Cr-C29 1.8675(14), C29-O29 1.1519(17), Cr-C30 1.8864(14), C30-O30 1.1497(17), Cr-P2 2.3374(4), Cr-C2 2.1108(13), C2-C1 1.3793(17), C1-P1 1.7914(13), C2-C1-P1 110.50(9), C1-P1-C8 94.42(6), C2-C1-C9 129.64(11).

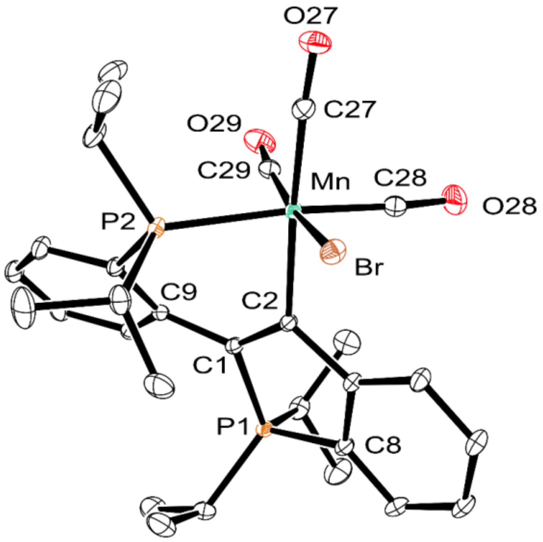

Figure S74: ORTEP plot of the molecular stucture of **7**. Displacement ellipsoids set to 30% probability, hydrogen atoms ommitted for clarity. Selected bond lenghts (Å) and angles (°): Mn-Br 2.6205(9), Mn-C27 1.8226(15), C27-O27 1.1446(18), Mn-C28 1.8195(16), C28-O28 1.1449(19), Mn-C29 1.7831(17), C29-O29 1.117(2), Mn-P2 2.3134(7), Mn-C2 2.0758(14), C2-C1 1.3747(18), C1-P1 1.8032(14), C2-C1-P1 110.72(9), C1-P1-C8 93.78(7), C2-C1-C9 129.34(11).

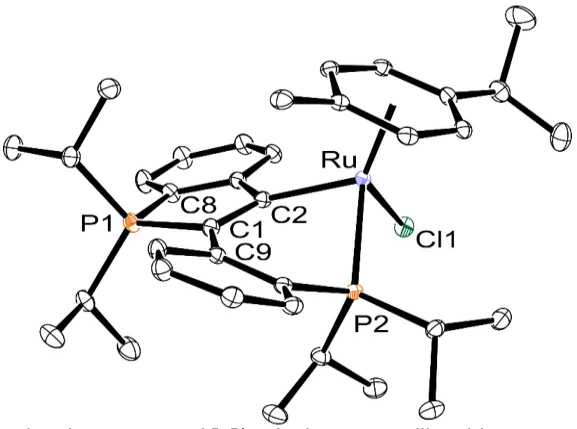

Figure S75: ORTEP plot of the molecular stucture of **[8]**$^{+}$. Displacement ellipsoids set to 30% probability, hydrogen atoms ommitted for clarity. Selected bond lenghts (Å) and angles (°): Ru-Cymene-plane(C27-C32) 1.7599(8), Ru-Cl1 2.4084(5), Ru-P2 2.3108(5), Ru-C2 2.0669(18), C2-C1 1.387(3), C1-P1 1.7946(19), C2-C1-P1 110.25(14), C1-P1-C8 94.12(9), C2-C1-C9 126.48(17).

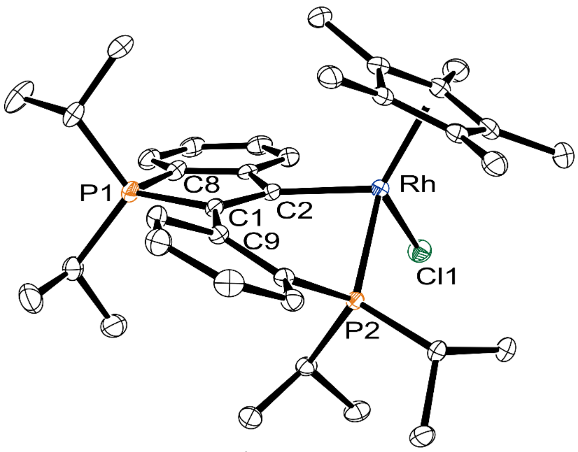

Figure S76: ORTEP plot of the molecular stucture of **[9]**$^{+}$. Displacement ellipsoids set to 30% probability, hydrogen atoms ommitted for clarity. Selected bond lenghts (Å) and angles (°): Rh-Cp*-plane(C27-C31) 1.8737(19), Rh-Cl1 2.4288(12), Rh-P2 2.2831(11), Rh-C2 2.046(4), C2-C1 1.369(6), C1-P1 1.807(4), C2-C1-P1 109.6(3), C1-P1-C8 94.0(2), C2-C1-C9 127.0(4).

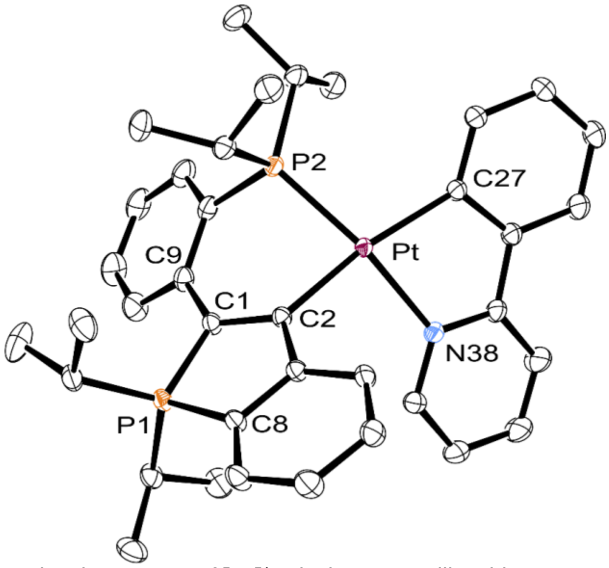

Figure S77: ORTEP plot of the molecular stucture of **[10]**$^{+}$. Displacement ellipsoids set to 30% probability, hydrogen atoms ommitted for clarity. Selected bond lenghts (Å) and angles (°): Pt-C27 2.070(2), Pt-N38 2.1078(17), Pt-P2 2.2260(5), Pt-C2 2.0591(19), C2-C1 1.369(3), C1-P1 1.801(2), C2-C1-P1 110.52(15), C1-P1-C8 93.66(9), C2-C1-C9 124.72(18).

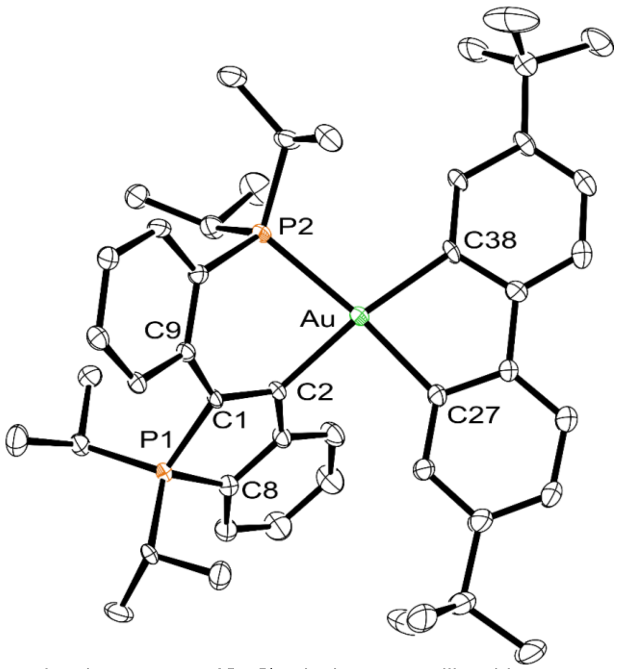

Figure S78: ORTEP plot of the molecular stucture of **[11]**$^+$. Displacement ellipsoids set to 30% probability, hydrogen atoms ommitted for clarity. Only one of the two disordered positions of the biphenyl-coligand is shown; values in square brackets refer to the second set. Selected bond lenghts (Å) and angles (°): Au-C27 2.076(5) [2.076(5)], Au-C38 2.097(4) [2.097(4)], Au-P2 2.3818(12), Au-C2 2.084(4), C2-C1 1.363(6), C1-P1 1.820(4), C2-C1-P1 109.0(3), C1-P1-C8 93.7(2), C2-C1-C9 128.1(4).

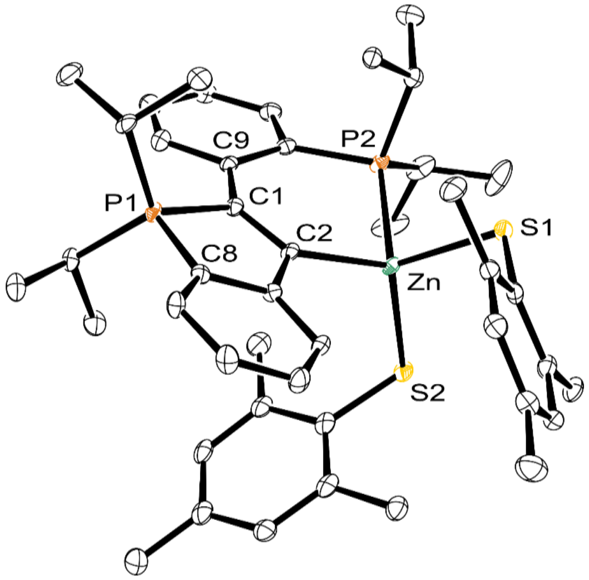

Figure S79: ORTEP plot of the molecular stucture of **12**. Displacement ellipsoids set to 30% probability, hydrogen atoms ommitted for clarity. Only one of the four independent molecules is shown, values in square brackets refer to the other independent molecules. Selected bond lenghts (Å) and angles (°): Zn1-S1 2.3550(16) [2.3342(14), 2.3276(14), 2.3602(16)], Zn1-S2 2.3220(15) [2.3237(16), 2.3349(16), 2.3161(15)], Zn1-P2 2.3957(18) [2.4306(16), 2.4213(16), 2.4049(18)], Zn1-C2 2.036(4) [2.044(4), 2.043(4), 2.037(4)], C2-C1 1.372(5) [1.355(6), 1.358(5), 1.362(5)], C1-P1 1.843(4) [1.828(4), 1.832(4), 1.836(4)], C2-C1-P1 108.4(3) [109.4(3), 109.9(3), 109.2(3)], C1-P1-C8 94.09(19) [93.71(18), 93.13(19), 93.79(19)], C2-C1-C9 130.9(4) [130.5(4), 130.2(4), 130.9(4)].

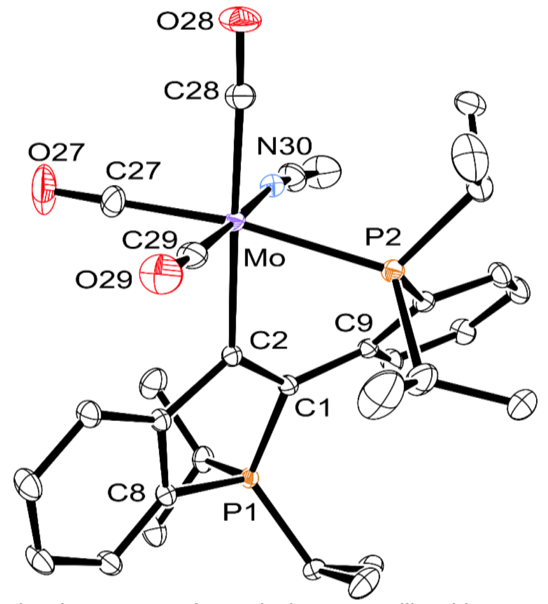

Figure S80: ORTEP plot of the molecular stucture of **13**. Displacement ellipsoids set to 30% probability, hydrogen atoms ommitted for clarity. Selected bond lenghts (Å) and angles (°): Mo-N30 2.2140(16), N30-C30 1.142(2), Mo-C27 1.9662(19), C27-O27 1.157(2), Mo-C28 1.9777(17), C28-O28 1.170(2), Mo-C29 1.9506(18), C29-O29 1.177(2), Mo-P2 2.5087(8), Mo-C2 2.2512(15), C2-C1 1.382(2), C1-P1 1.7981(15), C2-C1-P1 110.61(10), C1-P1-C8 93.92(7), C2-C1-C9 128.39(13).

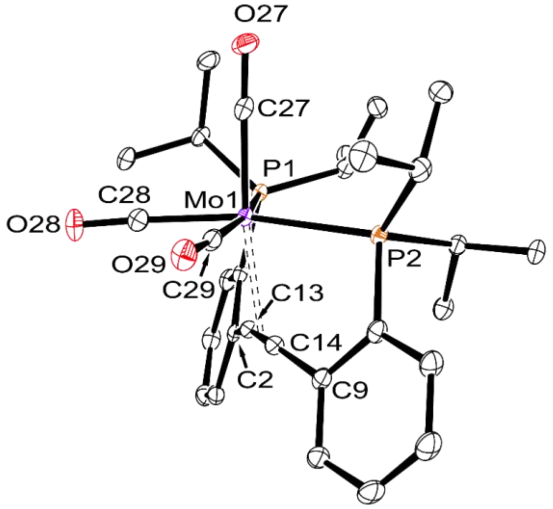

Figure S81: ORTEP plot of the molecular stucture of **14**. Displacement ellipsoids set to 30% probability, hydrogen atoms ommitted for clarity. Only one of the two independent molecules is shown, values in square brackets refer to the other independent molecule. Selected bond lenghts (Å) and angles (°): Mo1-C27 1.978(3) [1.982(4)], C27-O27 1.159(4) [1.153(4)], Mo1-C28 1.974(3) [1.963(3)], C28-O28 1.160(4) [1.158(4)], Mo1-C29 1.971(3) [1.994(4)], C29-O29 1.164(4) [1.150(4)], Mo1-P1 2.5671(8) [2.5393(8)], Mo1-P2 2.5560(8) [2.6011(8)], Mo1-C13 2.395(3) [2.380(3)], Mo1-C14 2.371(3) [2.402(3)], C13-C14 1.231(5) [1.238(4)], P1-Mo1-P2 105.60(3) [106.04(2)], C2-C13-C14 162.0(3) [159.2(3)], C13-C14-C8 159.9(3) [164.1(3)].

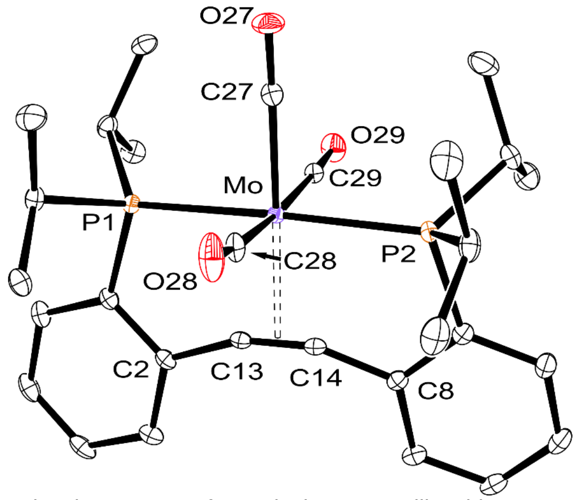

Figure S82: ORTEP plot of the molecular stucture of **15**. Displacement ellipsoids set to 30% probability, hydrogen atoms ommitted for clarity. Selected bond lenghts (Å) and angles (°): Mo-C27 1.9967(16), C27-O27 1.1545(19), Mo-C28 2.0205(16), C28-O28 1.150(2), Mo-C29 2.0325(16), C29-O29 1.1522(19), Mo-P1 2.4974(4), Mo-P2 2.4952(4), Mo-C13 2.3001(14), Mo-C14 2.2961(14), C13-C14 1.250(2), P1-Mo-P2 176.641(13), C2-C13-C14 158.66(15), C8-C14-C13 158.50(15).

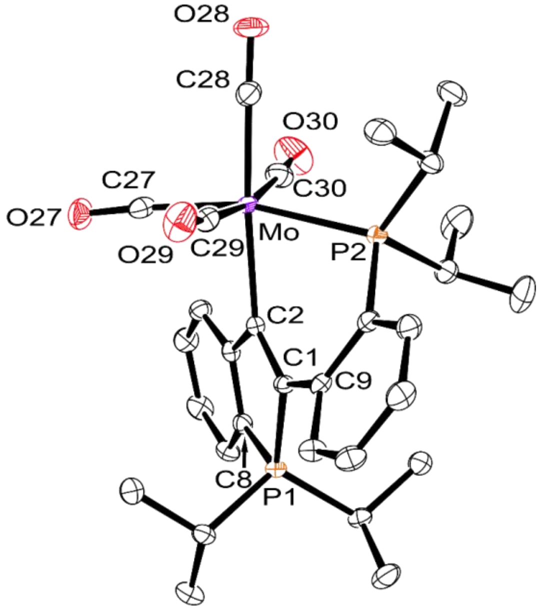

Figure S83: ORTEP plot of the molecular stucture of **16**. Displacement ellipsoids set to 30% probability, hydrogen atoms ommitted for clarity. Selected bond lenghts (Å) and angles (°): Mo-C27 1.978(5), C27-O27 1.162(5), Mo-C28 1.983(4), C28-O28 1.165(5), Mo-C29 2.050(4), C29-O29 1.138(5), Mo-C30 2.018(4), C30-O30 1.154(5), Mo-P2 2.4910(11), Mo-C2 2.253(4), C2-C1 1.376(5), C1-P1 1.807(4), C2-C1-P1 110.0(3), C1-P1-C8 94.31(18), C2-C1-C9 129.3(3).

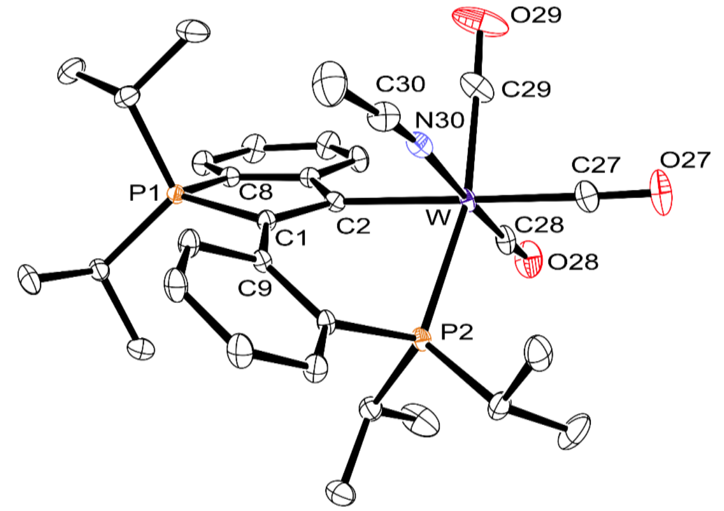

Figure S84: ORTEP plot of the molecular stucture of **13-W**. Displacement ellipsoids set to 30% probability, hydrogen atoms ommitted for clarity. Selected bond lengths (Å) and angles (°): W-N30 2.1952(18), N30-C30 1.142(2), W-C27 1.9790(19), C27-O27 1.174(2), W-C28 1.954(2), C28-O28 1.178(2), W-C29 1.965(2), C29-O29 1.161(3), W-P2 2.5019(9), W-C2 2.2410(18), C2-C1 1.387(2), C1-P1 1.7955(18), C2-C1-P1 110.65(12), C1-P1-C8 94.01(8), C2-C1-C9 128.11(15).

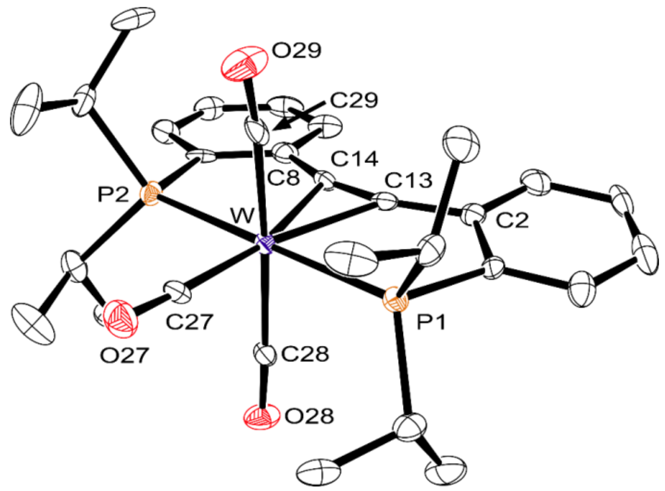

Figure S85: ORTEP plot of the molecular stucture of **15-W**. Displacement ellipsoids set to 30% probability, hydrogen atoms omitted for clarity. Selected bond lenghts (Å) and angles (°): W-C27 1.977(10), C27-O27 1.177(11), W-C28 2.007(10), C28-O28 1.170(11), W-C29 2.006(12), C29-O29 1.167(13), W-P1 2.500(3), W-P2 2.491(3), W-C13 2.267(10), W-C14 2.279(9), C13-C14 1.249(13), P1-W-P2 176.74(9), C2-C13-C14 157.3(11), C13-C14-C8 158.4(10).

Table S2: Crystal data and details of the structure determinations for **3**, **4**, **5**·CH_2Cl_2 and **6**.

Compound	**3**	**4**	**5**·CH_2Cl_2	**6**
Empirical Formula	$C_{70}H_{86}BP_2Y$	$C_{26}H_{36}Cl_4HfP_2$	$C_{33}H_{43}Cl_5NP_2Ta$	$C_{30}H_{36}CrO_4P_2$
Formula Weight	1089.04	730.78	873.82	574.53
Crystal system	monoclinic	monoclinic	orthorhombic	monoclinic
Space group	$P\ 2_1/c$	$P\ 2_1/c$	$P\ b\ c\ a$	$P\ 2_1/n$
a /Å	19.84621(17)	13.847(6) [b]	20.20265(10)	11.87864(15)
b /Å	15.33735(10)	11.756(6) [b]	16.84883(9)	15.68278(18)
c /Å	19.6362(2)	17.937(8) [b]	20.75660(9)	15.58857(18)
β /°	91.1824(8)	95.395(9) [b]		102.7735(12)
V /Å^3	5975.76(9)	2907(2) [b]	7065.36(6)	2832.13(6)
Z	4	4	8	4
F_{000}	2320	1448	3488	1208
d_c /Mg·m^{-3}	1.210	1.670	1.643	1.347
μ /mm^{-1}	2.172	4.080	3.605	0.550
max, min transmission factors	1.000, 0.664 [a]	0.649, 0.556 [c]	1.000, 0.651 [a]	1.000, 0.944 [c]
X-ray radiation, λ /Å	Cu $K\alpha$, 1.54184	Mo-$K\alpha$, 0.71073	Mo-$K\alpha$, 0.71073	Mo-$K\alpha$, 0.71073
data collect. temperature /K	120(1)	100(1)	120(1)	120(1)
θ range /°	2.2 - 71.2	2.1 - 32.5	2.2 – 30.5	2.2 - 32.4
index ranges h, k, l	±24, ±18, -22 ... 24	±20, ±17, ±26	±28, -23 ... 24, ±29	±17, -23 ... 22, ±23
reflections measured	223988	73827	191149	61874
unique [R_{int}]	11524 [0.08]	10012 [0.047]	10780 [0.036]	9755 [0.044]
observed [$I > 2\sigma(I)$]	9272	8570	9772	8822
parameters refined [restraints]	700 [79]	306 [0]	387 [0]	342 [0]
GooF on F^2	1.030	1.073	1.027	1.090
R indices [$F_0 > 4\sigma(F_o)$] $R(F)$, $wR(F^2)$	0.0474, 0.1042	0.0231, 0.0475	0.0196, 0.0461	0.0399, 0.0852
R indices (all data) R(F), $wR(F^2)$	0.0645, 0.1120	0.0321, 0.0508	0.0231, 0.0476	0.0461, 0.0862
difference density: max, min /e·Å^{-3}	1.167, -1.044	1.575, -0.686	2.381, -0.626	0.523, -0.457
deposition number CCDC	2036100	2036101	2036102	2036103

[a] numerical absorption correction.
[b] s.u. includes systematic error contributions from Monte Carlo simulations.
[c] semi-empirical absorption correction.

Table S3: Crystal data and details of the structure determinations for **7**·$1.5CH_2Cl_2$, [**8**]$SbF_6\cdot0.5C_5H_{12}\cdot0.5CH_2Cl_2$, [**9**]$SbF_6\cdot2CH_2Cl_2$, **10**·2thf.

Compound	**7**·$1.5CH_2Cl_2$	[**8**]$SbF_6\cdot0.5C_5H_{12}$ ·$0.5CH_2Cl_2$	[**9**]$SbF_6\cdot2CH_2Cl_2$	**10**·2thf
Empirical Formula	$C_{30.5}H_{39}BrCl_3MnO_3P_2$	$C_{39}H_{57}Cl_2F_6P_2RuSb$	$C_{38}H_{55}Cl_5F_6P_2RhSb$	$C_{45}H_{60}ClNO_2P_2Pt$
Formula Weight	756.75	995.50	1089.67	939.42
Crystal system	monoclinic	monoclinic	monoclinic	monoclinic
Space group	$P\,2_1/c$	$P\,2_1/n$	$P\,2_1/c$	$P\,2_1/c$
a /Å	12.120(5) [a]	14.68263(18)	8.68119(10)	18.17660(19)
b /Å	18.798(8) [a]	10.27641(16)	24.5831(3)	11.59885(8)
c /Å	15.683(6) [a]	28.8561(4)	20.7842(3)	21.4296(2)
β /°	111.303(6) [a]	94.9221(12)	96.7728(11)	114.9474(12)
V /Å^3	3329(2) [a]	4337.89(10)	4404.60(9)	4096.41(7)
Z	4	4	4	4
F_{000}	1548	2016	2192	1912
d_c /Mg·m^{-3}	1.510	1.524	1.643	1.523
μ /mm^{-1}	1.964	1.220	1.417	3.607
max, min transmission factors	0.495, 0.431 [b]	1.000, 0.902 [c]	1.000, 0.804 [c]	1.000, 0.629 [c]
X-ray radiation, λ /Å	Mo-$K\alpha$, 0.71073	Mo-$K\alpha$, 0.71073	Mo-$K\alpha$, 0.71073	Mo-$K\alpha$, 0.71073
data collect. temperature /K	100(1)	120(1)	120(1)	120(1)
θ range /°	1.8 - 32.5	2.4 – 34.8	2.1 – 28.9	2.1 - 34.8
index ranges *h*, *k*, *l*	±18, -27 ... 28, ±23	±23, ±16, -46 ... 45	±11, -31 ... 33, -28 ... 27	±29, ±18, ±34
reflections measured	84598	135520	128969	210353
unique [R_{int}]	11356 [0.031]	18168 [0.069]	11091 [0.068]	17347 [0.059]
observed [$I > 2\sigma(I)$]	9967	13473	8702	14940
parameters refined [restraints]	387 [0]	447 [49]	464 [138]	432 [30]
GooF on F^2	1.031	1.024	1.052	1.104
R indices [$F_0 > 4\sigma(F_o)$] $R(F)$, $wR(F^2)$	0.0285, 0.0754	0.0395, 0.0771	0.0562, 0.1219	0.0313, 0.0592
R indices (all data) R(F), $wR(F^2)$	0.0355, 0.0790	0.0632, 0.0844	0.0773, 0.1309	0.0408, 0.0617
difference density: max, min /e·Å^{-3}	1.062, -0.772	1.766, -1.095	1.970, -1.355	2.467, -2.656
deposition number CCDC	2036104	2036105	2036106	2036107

[a] s.u. includes systematic error contributions from Monte Carlo simulations.
[b] semi-empirical absorption correction.
[c] numerical absorption correction.

Table S4: Crystal data and details of the structure determinations for **11**·C_6H_5F·Et_2O, **12**·3thf, **13**·CH_3CN, **14**·1.5thf.

Compound	**11**·C_6H_5F·Et_2O	**12**·3thf	**13**·CH_3CN	**14**·1.5thf
Empirical Formula	$C_{86}H_{100}AuBF_2OP_2$	$C_{56}H_{82}O_3P_2S_2Zn$	$C_{33}H_{42}MoN_2O_3P_2$	$C_{35}H_{48}MoO_{4.5}P_2$
Formula Weight	1457.37	994.64	672.56	698.61
Crystal system	monoclinic	monoclinic	monoclinic	monoclinic
Space group	$P\ 2_1/c$	$P\ 2_1/c$	$P\ 2_1/n$	$C\ 2/c$
a /Å	11.28458(15)	20.688(11) [b]	10.771(4)	32.5140(4)
b /Å	13.79053(15)	26.218(15) [b]	18.080(7)	11.57882(9)
c /Å	47.7699(4)	40.07(2) [b]	16.992(7)	39.5477(4)
β /°	90.5291(10)	95.990(10) [b]	97.839(10)	113.9169(13)
V /Å^3	7433.66(14)	21614(20) [b]	3278(2)	13610.3(3)
Z	4	16	4	16
F_{000}	3024	8544	1400	5856
d_c /Mg·m^{-3}	1.302	1.223	1.363	1.364
μ /mm^{-1}	2.072	0.632	0.532	4.339
max, min transmission factors	0.993, 0.884 [a]	0.745, 0.651 [c]	0.746, 0.699 [c]	0.910, 0.761 [a]
X-ray radiation, λ /Å	Mo-$K\alpha$, 0.71073	Mo-$K\alpha$, 0.71073	Mo-$K\alpha$, 0.71073	Cu $K\alpha$, 1.54184
data collect. temperature /K	120(1)	100(1)	100(1)	120(1)
θ range /°	2.3 - 33.2	1.3 - 26.4	2.1 - 32.4	2.4 - 71.6
index ranges h, k, l	±17, ±21, ±73	±25, ±32, ±50	±16, -26 ... 27, ±25	-39 ... 37, ±14, ±48
reflections measured	399120	403063	83887	223964
unique [R_{int}]	28406 [0.159]	44237 [0.167]	11251 [0.042]	13157 [0.057]
observed [$I > 2\sigma(I)$]	18607	24856	9509	12071
parameters refined [restraints]	811 [678]	2166 [662]	379 [0]	782 [120]
GooF on F^2	1.127	1.006	1.031	1.026
R indices [$F_o > 4\sigma(F_o)$] $R(F)$, $wR(F^2)$	0.0764, 0.1264	0.0627, 0.1318	0.0316, 0.0746	0.0417, 0.0976
R indices (all data) R(F), $wR(F^2)$	0.1206, 0.1384	0.1325, 0.1603	0.0431, 0.0802	0.0457, 0.0998
difference density: max, min /e·Å^{-3}	1.117, -1.322	1.161, -1.011	2.019, -0.593	2.260, -1.295
deposition number CCDC	2036108	2036109	2036110	2036111

[a] numerical absorption correction.
[b] s.u. includes systematic error contributions from Monte Carlo simulations.
[c] semi-empirical absorption correction.

Table S5: Crystal data and details of the structure determinations for **15**, **16**·2thf, **13-W**·CH_3CN, **15-W**.

Compound	**15**	**16**·2thf	**13-W**·CH_3CN	**15-W**
Empirical Formula	$C_{29}H_{36}MoO_3P_2$	$C_{32}H_{52}MoO_6P_2$	$C_{33}H_{42}N_2O_3P_2W$	$C_{29}H_{36}O_3P_2W$
Formula Weight	590.46	762.67	760.47	678.37
Crystal system	orthorhombic	monoclinic	monoclinic	orthorhombic
Space group	*P b c a*	$P\,2_1/c$	$P\,2_1/n$	*P b c a*
a /Å	15.65317(6)	10.8424(3)	10.771(5) [b]	15.6520(7)
b /Å	16.11165(8)	15.9246(5)	18.020(9) [b]	16.0720(7)
c /Å	22.26048(9)	22.3877(5)	16.983(8) [b]	22.3038(12)
β /°		101.630(3)	97.989(12) [b]	
V /Å^3	5614.06(4)	3786.12(19)	3264(3) [b]	5610.7(5)
Z	8	4	4	8
F_{000}	2448	1600	1528	2704
d_c /Mg·m^{-3}	1.397	1.338	1.547	1.606
μ /mm^{-1}	5.116	0.474	3.672	4.259
max, min transmission factors	0.964, 0.636 [a]	1.000, 0.672 [a]	0.746, 0.672 [c]	1.000, 0.864 [a]
X-ray radiation, λ /Å	Cu $K\alpha$, 1.54184	Mo-$K\alpha$, 0.71073	Mo $K\alpha$, 0.71073	Mo $K\alpha$, 0.71073
data collect. temperature /K	120(1)	120(1)	100(1)	120(1)
θ range /°	4.0 - 71.1	2.3 - 30.6	2.1 - 32.5	2.2 – 26.0
index ranges *h*, *k*, *l*	±19, -19 ... 17, ±27	±15, ±22, ±31	-15 ... 16, -26 ... 27, ±25	-18 ... 10, -19 ... 9, -26 ... 9
reflections measured	201982	69079	83195	7849 [d]
unique [R_{int}]	5421 [0.036]	11568 [0.112]	11202 [0.036]	5205 [0.042]
observed [$I > 2\sigma(I)$]	5263	8007	9786	3581
parameters refined [restraints]	324 [0]	432 [0]	400 [31]	324 [0]
GooF on F^2	1.090	1.074	1.070	1.076
R indices [$F_0 > 4\sigma(F_o)$] $R(F)$, $wR(F^2)$	0.0192, 0.0447	0.0715, 0.1633	0.0206, 0.0439	0.0678, 0.1097
R indices (all data) R(F), $wR(F^2)$	0.0201, 0.0451	0.1090, 0.1833	0.0282, 0.0467	0.1058, 0.1252
difference density: max, min /e·Å^{-3}	0.330, -0.327	2.391, -1.146	1.251, -0.593	1.409, -1.000
deposition number CCDC	2036112	2036113	2036114	2036115

[a] numerical absorption correction.
[b] s.u. includes systematic error contributions from Monte Carlo simulations.
[c] semi-empirical absorption correction.
[d] Partial data shell only.

References

1 P. Federmann, H. K. Wagner, P. W. Antoni, J. Mörsdorf, J. L. Pérez Lustres, H. Wadepohl, M. Motzkus and J. Ballmann, *Org. Lett.*, 2019, **21**, 2033.
2 G. R. Fulmer, A. J. M. Miller, N. H. Sherden, H. E. Gottlieb, A. Nudelman, B. M. Stoltz, J. E. Bercaw and K. I. Goldberg, *Organometallics*, 2010, **29**, 2176.
3 W. J. Evans, B. L. Davis, T. M. Champagne and J. W. Ziller, *Proc. Natl. Acad. Sci. U.S.A.,* 2006, **103**, 12678-12683.
4 J. A. M.Canich, F. A. Cotton, S. A. Duraj and W. J. Roth, *Polyhedron*, 1986, **5**, 895.
5 G. T. Burns, E. Colomer, R. J. P. Corriu, M. Lheureux, J. Dubac, A. Laporterie and H. Iloughmane, *Organometallics*, 1987, **6**, 1398.
6 M. Pons, G. E. Herberich, P. N. Kirk, M. P. Castellani, M. J. Rizzo and J. D. Atwood, *Inorg. Synth*., 2014, **36**, 152.
7 J. Markham, J. Liang, A. Levina, R. Mak, B. Johannessen, P. Kappen, C. J. Glover, B. Lai, S. Vogt and P. A. Lay, *Eur. J. Inorg. Chem*., 2017, 1812.
8 N. Godbert, T. Pugliese, I. Aiello, A. Bellusci, A. Crispini and M. Ghedini, *Eur. J. Inorg. Chem*., 2007, 5105.
9 B. David, U. Monkowius, J. Rust, C. W. Lehmann, L. Hyzak and F. Mohr, *Dalton Trans*. 2014, **43**, 11059.
10 M. Bochmann, K. J. Webb, M. B. Hursthouse and M. Mazid, *J. Chem. Soc., Dalton Trans*. 1991, 2317.
11 F. Edelmann, P. Behrens, S. Behrens and U. Behrens, *J. Organomet. Chem*. 1986, **310**, 333.
12 D. J. Darensbourg and R. L. Kump, *Inorg. Chem*. 1978, **17**, 2680.
13 M. J. Frisch, G. W. Trucks, H. B. Schlegel, G. E. Scuseria, M. A. Robb, J. R. Cheeseman, G. Scalmani, V. Barone, G. A. Petersson, H. Nakatsuji, X. Li, M. Caricato, A. V. Marenich, J. Bloino, B. G. Janesko, R. Gomperts, B. Mennucci, H. P. Hratchian, J. V. Ortiz, A. F. Izmaylov, J. L. Sonnenberg, D. Williams-Young, F. Ding, F. Lipparini, F. Egidi, J. Goings, B. Peng, A. Petrone, T. Henderson, D. Ranasinghe, V. G. Zakrzewski, J. Gao, N. Rega, G. Zheng, W. Liang, M. Hada, M. Ehara, K. Toyota, R. Fukuda, J. Hasegawa, M. Ishida, T. Nakajima, Y. Honda, O. Kitao, H. Nakai, T. Vreven, K. Throssell, J. A. Montgomery, Jr., J. E. Peralta, F. Ogliaro, M. J. Bearpark, J. J. Heyd, E. N. Brothers, K. N. Kudin, V. N. Staroverov, T. A. Keith, R. Kobayashi, J. Normand, K. Raghavachari, A. P. Rendell, J. C. Burant, S. S. Iyengar, J. Tomasi, M. Cossi, J. M. Millam, M. Klene, C. Adamo, R. Cammi, J. W. Ochterski, R. L. Martin, K. Morokuma, O. Farkas, J. B. Foresman, and D. J. Fox, Gaussian 16, Revision B.01, *Gaussian, Inc., Wallingford CT*, 2016.
14 J. P. Perdew, K. Burke and M. Ernzerhof, *Phys. Rev. Lett.* 1997, **78**, 1396.
15 F. Weigend and R. Ahlrichs, *Phys. Chem. Chem. Phys.* 2005, **7**, 3297.
16 S. Grimme, J. Antony, S. Ehrlich and H. Krieg, *J. Chem. Phys.* 2010, **132**, Online Article No. 154104 https://doi.org/10.1063/1.3382344.
17 J. Tomasi, B. Mennucci and R. Cammi, *Chem. Rev.* 2005, **105**, 2999.
18 A. V. Marenich, C. J. Cramer and D. G. Truhlar, *J. Phys. Chem. B* 2009, **113**, 6378.
19 Lu, T. *Multiwfn*, University of Science and Technology Beijing, China, 2011.
20 G. Knizia, *J. Chem. Theory Comput.* 2013, **9** 4834.
21 M. Mitoraj, A. Michalak and T. Ziegler, *J. Chem. Theory Comput.* 2009 **5**, 962.
22 A. D. Becke, *Phys. Rev. A* 1988, **38**, 3098.
23 J. P. Perdew, *Phys. Rev. B* 1986, **33**, 8822.
24 G. Te Velde, F.M. Bickelhaupt, E. J. Baerends, C. Fonseca Guerra, S. J. A. Van Gisbergen, J. G. Snijders and T. Ziegler, *J. Comput. Chem.* 2001, **22**, 931.
25 E.J. Baerends, T. Ziegler, A.J. Atkins, J. Autschbach, O. Baseggio, D. Bashford, A. Bérces, F.M. Bickelhaupt, C. Bo, P.M. Boerrigter, L. Cavallo, C. Daul, D.P. Chong, D.V. Chulhai, L. Deng, R.M. Dickson, J.M. Dieterich, D.E. Ellis, M. van Faassen, L. Fan, T.H. Fischer, C. Fonseca Guerra, M. Franchini, A. Ghysels, A. Giammona, S.J.A. van Gisbergen, A. Goez, A.W. Götz, J.A. Groeneveld, O.V. Gritsenko, M. Grüning, S. Gusarov, F.E. Harris, P. van den Hoek, Z. Hu, C.R. Jacob, H. Jacobsen, L. Jensen, L. Joubert, J.W. Kaminski, G. van Kessel, C. König, F. Kootstra, A. Kovalenko, M.V. Krykunov, E. van Lenthe, D.A. McCormack, A. Michalak, M. Mitoraj, S.M. Morton, J. Neugebauer, V.P. Nicu, L. Noodleman, V.P. Osinga, S. Patchkovskii, M. Pavanello, C.A. Peeples, P.H.T. Philipsen, D. Post, C.C. Pye, H. Ramanantoanina, P. Ramos, W. Ravenek, J.I. Rodríguez, P. Ros, R. Rüger, P.R.T. Schipper, D. Schlüns, H. van Schoot, G. Schreckenbach, J.S. Seldenthuis, M. Seth, J.G. Snijders, M. Solà, M. Stener, M. Swart, D. Swerhone, V. Tognetti, G. te Velde, P. Vernooijs, L. Versluis, L. Visscher, O. Visser, F. Wang, T.A. Wesolowski, E.M. van Wezenbeek, G. Wiesenekker, S.K. Wolff, T.K. Woo and A.L. Yakovlev, *ADF*, Vrije Universiteit, Amsterdam, The Netherlands, 2017.
26 a) E. Van Lenthe, E. J. Baerends and J. G. Snijders, *J. Chem. Phys.* 1993, **99**, 4597; b) E. Van Lenthe, E. J. Baerends and J. G. Snijders, *J. Chem. Phys.* 1994, **101**, 9783; c) E. Van Lenthe, A. Ehlers and E. J. Baerends, *J. Chem. Phys.* 1999, **110**, 8943.
27 Chemcraft - graphical software for visualization of quantum chemistry computations (www.chemcraftprog.com).
28 K. Kabsch, in: M. G. Rossmann, E. Arnold (eds.), "*International Tables for Crystallography*" Vol. F, Ch. 11.3, Kluwer Academic Publishers, Dordrecht, The Netherlands, 2001.
29 *SAINT*, Bruker AXS GmbH, Karlsruhe, Germany 1997-2019.
30 *CrysAlisPro*, Agilent Technologies UK Ltd., Oxford, UK 2011-2014 and Rigaku Oxford Diffraction, Rigaku Polska Sp.z o.o., Wrocław, Poland 2015-2020.
31 R. H. Blessing, *Acta Cryst*. 1995, **A51**, 33.
32 (a) G. M. Sheldrick, *SADABS*, Bruker AXS GmbH, Karlsruhe, Germany 2004-2016; (b) L. Krause, R. Herbst-Irmer, G. M. Sheldrick, D. Stalke, *J. Appl. Cryst*. 2015, **48**, 3.
33 *SCALE3 ABSPACK*, *CrysAlisPro*, Agilent Technologies UK Ltd., Oxford, UK 2011-2014 and Rigaku Oxford Diffraction, Rigaku Polska Sp.z o.o., Wrocław, Poland 2015-2020.

34 W. R. Busing, H. A. Levy, *Acta Cryst*. 1957, **10**, 180.
35 C. Giacovazzo "*Phasing in Crystallography*", IUCr and Oxford Science Publications, Oxford, UK, 2013.
36 M. C. Burla, R. Caliandro, B. Carrozzini, G. L. Cascarano, C. Cuocci, C. Giacovazzo, M. Mallamo, A. Mazzone, G. Polidori, *J. Appl. Cryst*. 2015, **48**, 306.
37 M. C. Burla, R. Caliandro, B. Carrozzini, G. L. Cascarano, C. Cuocci, C. Giacovazzo, M. Mallamo, A. Mazzone, G. Polidori, D. Siliqi *SIR2019*, CNR IC, Bari, Italy, 2019.
38 (a) P. T. Beurskens, G. Beurskens, R. de Gelder, J. M. M. Smits, S. Garcia-Granda, R. O. Gould, *DIRDIF-2008*, Radboud University Nijmegen, The Netherlands, 2008; (b) P. T. Beurskens, in: G. M. Sheldrick, C. Krüger, R. Goddard (eds.), „*Crystallographic Computing 3*", Clarendon Press, Oxford, UK, 1985, p. 216.
39 M. C. Burla, R. Caliandro, B. Carrozzini, G. L. Cascarano, C. Cuocci, C. Giacovazzo, M. Mallamo, A. Mazzone, G. Polidori, *SIR2014*, CNR IC, Bari, Italy, 2014.
40 (a) L. Palatinus, *SUPERFLIP*, EPF Lausanne, Switzerland and Fyzikální ústav AV ČR, v. v. i., Prague, Czech Republic, 2007-2014; (b) L. Palatinus, G. Chapuis, *J. Appl. Cryst*. 2007, **40**, 786; (c) L. Palatinus, *Acta. Cryst*. 2013, **B69**, 1.
41 (a) G. M. Sheldrick, *SHELXL-20xx*, University of Göttingen and Bruker AXS GmbH, Karlsruhe, Germany 2012-2018; (b) W. Robinson, G. M. Sheldrick in: N. W. Isaaks, M. R. Taylor (eds.) „*Crystallographic Computing 4*", Ch. 22, IUCr and Oxford University Press, Oxford, UK, 1988; (c) G. M. Sheldrick, *Acta Cryst*. 2008, **A64**, 112; (d) G. M. Sheldrick, *Acta Cryst*. 2015, **C71**, 3.
42 (a) J. S. Rollett in: F. R. Ahmed, S. R. Hall, C. P. Huber (eds.) „*Crystallographic Computing*" p. 167, Munksgaard, Copenhagen, Denmark, 1970; (b) D. Watkin in: N. W. Isaaks, M. R. Taylor (eds.) „*Crystallographic Computing 4*", Ch. 8, IUCr and Oxford University Press, Oxford, UK, 1988; (c) P. Müller, R. Herbst-Irmer, A. L. Spek, T. R. Schneider, M. R. Sawaya in: P. Müller (ed.) "*Crystal Structure Refinement*", Ch. 5, Oxford University Press, Oxford, UK, 2006; (d) D. Watkin, *J. Appl. Cryst*. 2008, **41**, 491.
43 A. Thorn, B. Dittrich, G. M. Sheldrick, *Acta Cryst*. 2012, **A68**, 448.
44 P. v. d. Sluis, A. L. Spek, *Acta Cryst*. 1990, **A46**, 194; (b) A. L. Spek, *Acta Cryst*. 2015, **C71**, 9.
45 A. L. Spek, *PLATON*, Utrecht University, The Netherlands; (b) A. L. Spek, *J. Appl. Cryst*. 2003, **36**, 7.
